# Multivalency influences virus dynamics at the glycocalyx: A study of glycosaminoglycan binding in HPV16 entry

**DOI:** 10.1016/j.bpj.2026.05.014

**Published:** 2026-05-12

**Authors:** Dario Valter Conca, Fouzia Bano, Yara Abidine, Laura Soria Martinez, Kerstin Seier, Justas Svirelis, Andreas Dahlin, Mario Schelhaas, Marta Bally

**Affiliations:** 1 Department of Clinical Microbiology, Wallenberg Centre for Molecular Medicine and Umeå Centre for Microbial Research, Umeå University, Umeå, Sweden; 2 Institute of Cellular Virology, ZMBE, University of Münster, Münster, Germany; 3 Department of Chemistry and Chemical Engineering, Chalmers University of Technology, 412 96 Gothenburg, Sweden; 4 Science for Life Laboratory (SciLifeLab), Umeå University, Umeå, Sweden

## Abstract

Virus attachment at the cell surface often involves the establishment of multiple ligand-receptor interactions between viral capsid proteins and glycans in the glycocalyx. Multivalency likely contributes to strengthening and fine-tuning virus dynamics at the cell surface to optimize entry. Here, we present experimental and theoretical frameworks to describe how virus attachment, detachment, and diffusion at the cell surface are modulated by multivalency. We focus on human papillomavirus 16 (HPV16), a leading cause of cervical cancer and its multivalent interactions with heparan sulfate (HS), a ubiquitous cell-surface glycan. Using single-particle tracking microscopy, we investigate the dynamic behavior of HPV16 particles interacting with multiple HS chains through a glycocalyx mimic consisting of end-tethered heparin chains that were selectively desulfated. We further establish a theoretical framework to predict the dynamic behavior of individual virus particles interacting with multiple cellular receptors and apply it to the HPV16-HS binding system using previously published association and dissociation rates of the individual capsomer-glycan bonds. Our experiments reveal that N-sulfation is essential to ensure HPV16 association and validate our theoretical prediction that at biologically relevant timescales, the lifetime of monovalent interaction primarily influences the apparent particle attachment behavior. Conversely, the nature of the sulfate group had a marginal effect on particle dissociation in the experiments, due to the great influence of multivalency on the particle’s interaction lifetime. Experiments and simulations also indicate that the mobility of HPV16 particles on heparin surfaces is highly restricted due to the relatively high affinity of the monovalent interactions, suggesting that particle motion due to the creation and rupturing of individual bonds is unlikely key in HPV16 recruitment. Together, these findings provide quantitative mechanistic insights into how virus-glycan interactions are modulated. They highlight the importance of HS chemistry in modulating early HPV16 engagement and suggest new targets against viral binding.

## Significance

To optimize entry, viruses modulate their dynamic behavior at the cell surface through multivalent interactions with glycans. In this work, we establish a theoretical framework describing how individual ligand-receptor interactions culminated into a fine-tuned virus attachment, detachment, and diffusion behavior in the glycocalyx. Theory and simulations are validated by experimental investigations of the multivalent interaction of individual HPV16 particles with heparin, which complement previous investigations of individual capsomer-heparin bonds. The presented theoretical frameworks represent a mechanistic basis for molecular-level understanding of the early stages of viral attachment and can be readily applied to other viruses. Our experimental findings contribute to our understanding of HPV16’s biology and will facilitate the design of therapeutic strategies targeting HS sulfation to block HPV16 infection.

## Introduction

The first step of viral infection requires the attachment of a virus to the cell surface, a process eventually triggering its uptake by the host cell. The viral attachment process is mediated by specific biomolecular recognition events occurring between viral proteins and cellular biomolecules. Given that viruses are often highly symmetric and carry multiple copies of a binding protein, they interact by establishing multiple bonds with molecular species at the cell surface. In this context, the term bond is intended as the ensemble of atomic-scale interactions that results in a transient association between individual viral binding proteins and individual viral receptors on the cell surface. Here, multivalency is likely to offer exquisite possibilities to fine-tune the dynamic behavior of a virus at the cell surface and to optimize the entry process. Many viruses initially bind carbohydrates in the glycocalyx, the sugar coat covering the cell surface, before transferring to more specific entry-receptors that trigger virus uptake.^1^^,^^2^ Such protein-carbohydrate interactions are often relatively weak but can be significantly strengthened through multivalency, thereby modulating the dynamic characteristics of the initial contact of a virus approaching a target cell.^3^^,^^4^^,^^5^ While the importance of multivalency in tuning viral interactions at the cell surface is being increasingly acknowledged,^5^^,^^6^^,^^7^ theoretical and experimental frameworks quantitatively relating the characteristics of individual ligand-receptor interactions to the dynamic behavior of a virus particle interacting through multivalent interactions have remained limited. In this context, *in vitro* glycocalyx models such as surface-immobilized glycosaminoglycan (GAG)^8^^,^^9^^,^^10^ and synthetic glycopolymers^11^^,^^12^ have shown a great potential; they have been previously used to study how glycan density and spatial organization can modulate viral binding kinetics.

**Figure 1:**
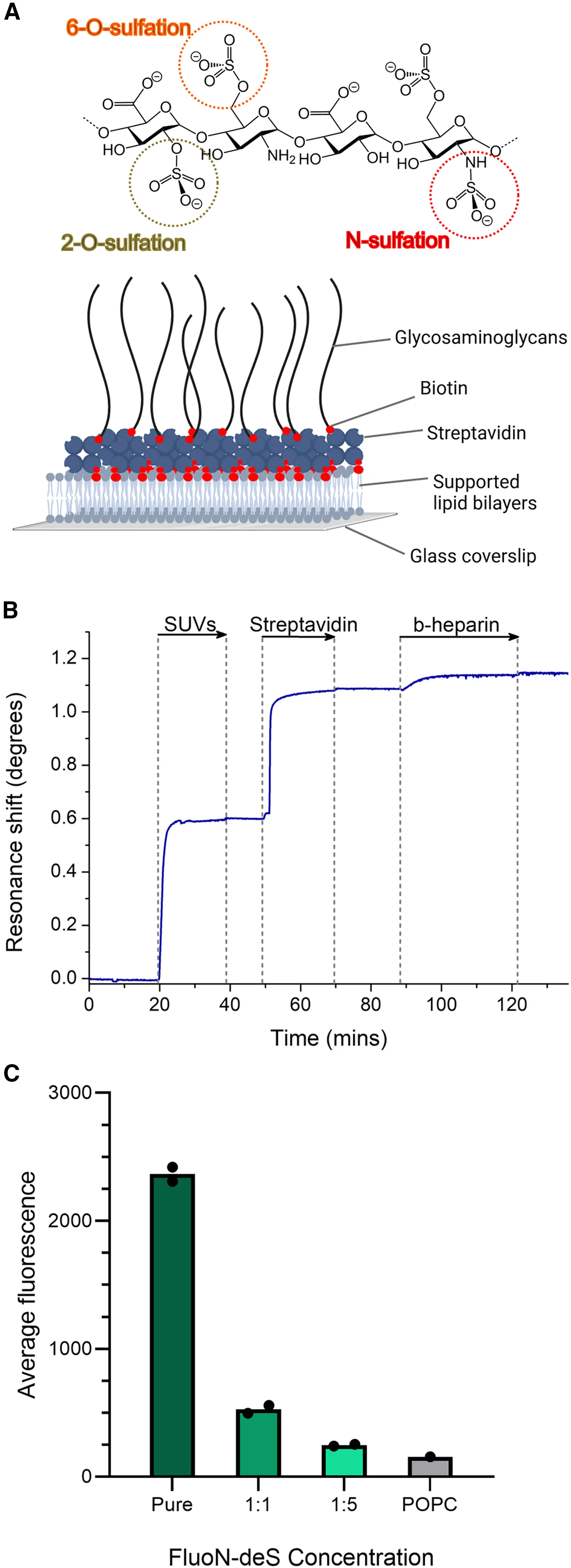
Characterization of GAG platform. (A) Chemical structure of two disaccharide units of heparin with sulfation groups (top) and illustration of the design of a surface displaying end-grafting immobilization of glycosaminoglycans via biotin on a monolayer of streptavidin formed on a supported lipid bilayer (not to scale, bottom). (B) A representative SPR sensogram (670 nm) for the step-by-step assembly of heparin films. Stability of each immobilized biomolecule is demonstrated by no further shift during rinsing with PBS buffer. (C) Mean of the fluorescence images (Figure S3B) from 2 identically prepared wells for each condition except POPC (black dots). Conditions from left to right: full layer of FluoN-deS heparin (pure); 1:1 (molar ratio) mixture of biotinylated FluoN-deS and biotinylated PEG (1:1); 1:5 (molar ratio) mixture of biotinylated FluoN-deS and biotinylated PEG (1:5); POPC surfaces exposed to biotinylated-fluorescent N-deS heparin (POPC) as a negative control.

Heparan sulfate (HS), a ubiquitous GAG found in the glycocalyx and extracellular matrix, mediates a variety of cellular processes involving multivalent interactions with proteins at the cell surface, for example cell signaling, cell adhesion, or the regulation of immune responses.^13^^,^^14^ Conversely, many viral pathogens exploit them to initiate infection. Viruses binding to HS include human papillomaviruses (HPVs), herpes simplex virus (HSV), human immunodeficiency virus, SARS-COV-2, and many more.^8^^,^^15^^,^^16^ HS is a linear polysaccharide composed of a repeating disaccharide backbone, made of glucuronic or iduronic (IdoA) acid and N-acetylglycosamine (GlcNAc) units. This periodic structure is often modified by the addition of sulfation groups in well-defined positions, such as N-sulfated glucosamine, and sulfation of the C6 and C3 positions of GlcNAc and the C2 position of IdoA (6-O, 3-O, and 2-O sulfation, respectively) (Figure 1A).^17^ HS is synthesized and sulfated enzymatically, resulting in highly heterogeneous glycan chains and sulfation patterns in terms of the level and type of modifications, whose nature and distribution are likely cell and tissue specific. These structural and spatial heterogeneities of HS chains on mammalian cells are likely key in defining the host specificity, tissue tropism, and severity of disease caused by many GAG-binding viruses.^18^^,^^19^

HPVs, a large family of small (∼50 nm in diameter) nonenveloped DNA viruses, use HS for initial attachment at the cell surface.^20^^,^^21^^,^^22^^,^^23^ These viruses are of major public health concern, as they can be etiological agents for several anogenital and oropharyngeal cancers, with HPVs detected in more than 90% of invasive cervical cancer cases.^24^^,^^25^ HPV type 16 (HPV16), in particular, represents the highest-risk genotype and is responsible for approximately 60% of cervical cancer cases worldwide, across cancer subtypes and geographic regions.^26^ HPV has an icosahedral capsid composed of 72 pentamers of the major capsid proteins L1 and up to an equal number of minor capsid protein L2.^27^^,^^28^ Binding to HS occurs through the engagement of several binding pockets located on the HPV16 capsomer.^20^^,^^27^ This interaction is of particular importance to the infection process as it triggers a conformational change in the capsid, thereby structurally activating it for further steps of infections.^29^^,^^30^ Specifically, structural activation promotes a cascade of enzymatic reactions that further modify the capsid, leading to a loss of the virus’ ability to bind to HS and its transfer to an entry receptor whose identity remains elusive.^31^ In this process, specific sulfation groups and patterns of HS have been suggested to play a key role in mediating functional binding and structural activation of HPV16.^23^^,^^29^^,^^30^ The entry process of HPV16 is uniquely slow. While enzymatic processing of the capsid likely starts within minutes after attachment, the whole entry process is known to take several hours.^31^^,^^32^

In a recent study, we have used atomic force microscopy (AFM)-based single-molecule force spectroscopy (SMFS) to quantify the association (k_on_) and dissociation rate constants (k_off_) of individual ligand-receptor bonds occurring between a single HPV16 virus particle and single heparin chains. Using either heparin, a secreted highly sulfated structural analog of HS, or various desulfated heparin derivatives, we showed that all types of sulfation contribute similarly to the initial engagement of the capsomer with a heparin chain, while N-sulfation is crucially needed to increase the interaction’s lifetime.^30^ Here, we focus on the binding kinetics and diffusion of HPV16 particles in the context of their multivalent interaction with heparin. Using a glycocalyx-mimetic platform, produced by end-grafting GAG chains on a surface, we quantify HPV16 particle interaction kinetics and diffusion on this surface using single-particle tracking (SPT) of the particles bound to the surface by total internal reflection fluorescence (TIRF) microscopy. Additionally, we present a theoretical framework that links the properties of single capsomer-glycan interactions to the characteristics of a multivalent particle-glycan interaction. We confirm that N-sulfates play a key role in mediating the interaction between HPV16 and heparin. However, at timescales of relevance to the biological processes under investigations, i.e., minutes to hours, the lifetime of the monovalent interaction primarily influences the apparent attachment behavior of the multivalent particle, resulting in a nearly 1 order of magnitude increase in particle binding rates for HPV16 when N-sulfation is present. Conversely, the nature of the sulfate group had a minimal effect on the dissociation, with particle dissociation rate constants in the same order of magnitude for all cases. In addition to this, theoretical modeling and experimental investigations of virus motion on surfaces presenting immobile heparin ligands reveal that in all cases investigated here, the monovalent capsomer-heparin interaction is too strong to permit multivalency-assisted diffusion of the particle on the surface, i.e., motion of the particles on the surface presenting immobile receptors due to the creation and rupturing of individual capsomer-heparin bonds.

## Materials and methods

### Buffer and proteins

All experiments were performed in phosphate-buffered saline (PBS) buffer at pH 7.4, which was prepared by dissolving 1 PBS tablet (Medicago AB, Uppsala, Sweden) in 1 L Milli-Q water (Millipore integral system, Molsheim, France) and filtering with 0.2 μm filters (Sarstedt, Germany) before use.

Lyophilized streptavidin (Sigma-Aldrich, St. Louis, MO) was dissolved in Milli-Q water at 5 mg/mL and stored at −80°C. Thawed aliquots were used within a few days. Alexa Fluor 488 succinimidylester was from Thermo Fisher Scientific.

### Virus production and labeling

HPV16 pseudoviruses (PsVs) containing a GFP reporter plasmid were produced using 293TT cells as previously described.^30^ In brief, 293TT cells were transfected with p16SheLL and pCIneo. After 48 h, cells were harvested and lysed. For optimal maturation, lysates were incubated for a further 24 h with 25 mM ammonium sulfate (pH 9.0) at 37°C. PsVs were purified on a 25%–39% linear Optiprep gradient (Sigma-Aldrich).

HPV16 PsVs were covalently labeled with Alexa Fluor dyes as previously described.^30^^,^^33^ In short, HPV16 PsVs were incubated for 1 h with NHS ester of the fluorescent dye Alexa Fluor 488 at a 1:8 molar ratio (L1/dye). Labeled HPV16 PsVs were separated from free dye by an Optiprep step gradient (5%/39%). Stock concentration of WT HPV16 was 130 μg/mL, which was determined after SDS-PAGE and Coomassie staining by densitometry relative to a standard of BSA. It should be noted that throughout this study, all mentions of particles or viruses specifically refer to HPV16 PsVs.

### GAGs

All GAGs used in this study are listed in Table S1 together with their biochemical characteristics. Lyophilized heparin (HEP001) and 3 selectively desulfated heparins (N-desulfated, DSH003/N; 2-O-desulfated, DSH001/2; 6-O-desulfated, DSH002/6) were purchased from Iduron, UK. According to the supplier’s product data sheet, there was ∼92% reduction in N-sulfates for DSH003/N, ∼90% reduction in 6-O sulfates with 25% loss of 2-O sulfates for DSH002/6, and ∼95% reduction in 2-O sulfates for DSH001/2 as compared with parental heparin. Lyophilized end-biotinylated hyaluronan (HA) was purchased from INNOVENT e.V. Technologieentwicklung, Jena, Germany (KS855). All GAGs were dissolved in Milli-Q water at the desired concentration and gently shaken overnight at 4°C before aliquoting. Heparin and the selectively desulfated variants were biotinylated via oxime ligation at their reducing end as described previously.^30^^,^^34^ Stock solutions of all GAGs were aliquoted and stored at −20°C after biotinylation. Thawed aliquots were used within a few weeks.

### Lipid vesicle preparation

1-palmitoyl-2-oleoyl-sn-glycero-3-phosphocholine (POPC), cat. no. 850457P, and 18:1 Biotinyl Cap PE (DOPE-Cap-B), cat. no. 870273P, were purchased from Avanti Polar Lipids (Alabaster, AL, USA). Small unilamellar vesicles (SUVs) were prepared from a mixture of POPC:DOPE-Cap-B (90:10, molar ratio) in PBS at a stock concentration of 2 mg/mL by extrusion through a 50 nm polycarbonate membrane at least 21 times using a mini extruder equipped with a 1 mL syringe (Avanti Polar Lipids; 610020 and 610017) as previously described.^9^ Stocks were stored at 4°C under N_2_ and used within a few weeks.

### Surface plasmon resonance

Surface plasmon resonance (SPR) chips with ∼2 nm Cr and 50 nm Au were prepared by electron beam heated physical vapor deposition (Lesker PVD 225) on glass substrates (20 × 12 mm; Bionavis), which were cleaned beforehand with RCA-2 (1:1:5 volume of conc. HCl:H_2_O_2_ [30%]:H_2_O [ultrafiltered at 18.6 MΩs] at 80°C) and 50 W O_2_ plasma at 250 mTorr. Moreover, a thin silica layer (∼11 nm) was deposited on top of the gold layer with atomic layer deposition (Oxford FlexAL) at 300°C by using a bis(t-butylamino)silane (BTBAS) as a precursor and oxygen as processing gas. Sensor surfaces were incubated in a UV O_3_ chamber for at least 30 min before use.

SPR Navi 220 A instrument (BioNavis) is equipped with three lasers (wavelengths of 785 nm, 670 nm, and 980 nm), which induce different decay lengths of sensing fields. The SPR spectra can be acquired in parallel from 2 flow channels in a polyether ether ketone flow cell. The running buffer was PBS (Merck), which was degassed using a combined setup of vacuum pump (DIVAC 1.4HV3C) and sonicator bath (35 kHz). The flow rate was between 5 and 20 μL/min, and the temperature was kept at 25°C. The quantification of mass coverage on silica-coated gold sensors was done in a similar manner as reported before^35^ (see supplemental information, Section S2). In brief, the SPR signals were used to calculate the surface molecular grafting coverage, i.e., the number of GAG chains per unit area, by employing independently determined parameters (molar refractivity, bulk sensitivity, and plasmonic field decay length). The grafting coverage was calculated based on the mass density and the molecular weight of biomolecules. The average spacing between GAG chains was calculated as the inverse of the square root of the grafting coverage, thus assuming a square arrangement.

Condition for self-assembling GAGs films in SPR experiments was as follows: (1) the formation of supported lipid bilayer (SLB) was done by the injection of SUVs containing 10 mol % biotin functionalized lipids at concentration of 100 μg/mL with flow rate of 20 μL/min; (2) next the streptavidin monolayer was formed by exposing SLBs at concentration of 50 μg/mL with flow rate of 12.5 μL/min; and (3) finally the binding of biotinylated GAGs or biotinylated polyethylene glycol (PEG) was performed at concentration of 10 μg/mL with flow rate of 5 μL/min.

### Fluorescently labeled biotinylated GAG film

In our system, we control the grafting coverage of GAGs by premixing them with biotinylated PEG (biotin-PEG-SH; biotinylated PEG with thiol end group; molecular weight of 3.4 kDa; RAPP polymere, Germany). The surface coverage of GAG in the GAG-PEG mixture was deduced from SPR measurements (Table S3) and the fluorescence intensity of surface-immobilized fluorescently labeled N-desulfated (FluoN-deS). For details on how the N-desulfated heparin was fluorescently labeled with an Alexa Fluor 488 derivative (FluoN-deS), see supplemental information, Section S6. To prepare pure FluoN-deS and mixed FluoN-deS:PEG layers, 8 wells were created on cleaned glass coverslips and prepared with a streptavidin layer as described below in TIRF microscopy. The biotinylated heparin derivatives, either alone or mixed with biotin-PEG-SH, were immobilized on the surface by incubating them with the solution for 30 min at the following concentrations: biotinylated FluoN-deS at 10 μg/mL (FluoN-deS in Figure 1C) or biotinylated FluoN-deS (1 μg/mL) mixed with biotin-PEG-SH at the concentration of either 0.227 μg/mL or 1.13 μg/mL to have the molar ratios of 1:1 and 1:5, respectively. All experiments were performed in duplicate. Pure POPC SLBs with and without incubation with biotinylated FluoN-deS (10 μg/mL for 30 min) were used as negative controls.

Each sample was imaged at 12 random positions on the surface using an inverted Nikon Eclipse Ti2-E microscope. Assuming linearity between the GAG density at the surface and the fluorescent signal, we can calculate the surface coverage (SC) of the GAG-PEG mixture from the SC of immobilized pure GAG, as measured with SPR, using the following formula: ${SC}_{\text{FluoN}-\text{deS}:\text{PEG}::1:x}=\frac{I_{\text{FluoN}-\text{deS}}-I_{\text{POPC}}}{I_{\text{FluoN}-\text{deS}:\text{PEG}::1:x}-I_{\text{POPC}}}{SC}_{\text{FluoN}-\text{deS}}$, where *SC*_surface_ and *I*_surface_ are the surface coverage and intensity of the corresponding surface, respectively, and X represents either 1 or 5.

### TIRF microscopy

The surfaces for TIRF microscopy were prepared using a mixture of biotinylated GAGs and PEG. For TIRF microscopy-based kinetic and diffusion experiments, glass coverslips were cleaned and UV treated as described previously.^30^ Briefly, round microscope glass coverslips (24 mm diameter, Deckglaser, Germany) were clened with 1:6 (v/v) solution of 7x detergent (MP Biomedicals, CA) and Milli-Q water at near boiling temperature for 2 h. The slides were then thoroughly rinsed and stored in Milli-Q water. Immediately prior to use, the slides were rinsed again with Milli-Q water, dried with N_2_, and treated with UV (UV Ozone Cleaner - ProCleaner Plus, BioForce Nanosciences, IL) for 30 min. Then, two slides were glued to a custom-designed Teflon holder using Twinsil glue (Picodent, Germany) to create 6 separated wells that can hold a maximum volume of 250 μL. SLBs were formed by spontaneous rupture of liposomes composed of 90:10 (molar ratio) POPC and DOPE-Cap-B at a concentration of 100 μg/mL. SLBs were then rinsed extensively and incubated for 30 min with POPC vesicles at 50 μg/mL to fill up any potential gaps in the SLBs. After removing excess unbound vesicles, each well was incubated with streptavidin in PBS at a final concentration of 50 μg/mL for 30 min. After rinsing, the wells were incubated with 2% v/w paraformaldehyde (PFA) from VWR for 15 min. Following a rinsing step with PBS, a solution mixture of desired biotinylated GAGs and biotin-PEG-SH was added to each well at molar ratio of 1:5 for 30 min. One well was incubated with biotin-PEG-SH only at final concentration of 25 μg/mL for 30 min. This well was used as negative control. After rinsing with PBS, all wells were incubated with 50 μg/mL pure POPC vesicles for ∼1 h. The HPV16 particles were diluted (∼5,714× stock) and added to the wells in 50 μg/mL pure POPC vesicles in PBS, to further minimize nonspecific adsorption. Rinsing was performed by dispensing 150 μL of PBS into each well while maintaining a residual volume of 50 μL to prevent the wells from drying out. This procedure was repeated 14 times for each rinsing step. All reagents were prepared at twice the working concentration, and 50 μL of reagent was added to the 50 μL residual volume already present in each well. For N-deS and HA films, we used 3 times higher virus concentration than the rest of the conditions. A high density of biotin within the SLBs was used to ensure the formation of a densely packed and immobile layer of streptavidin molecules after PFA fixation. This prevents clustering of HS around the particles as well as particle motion arising from HS diffusion.

TIRF imaging of single virus particles was as follows: an inverted Nikon Eclipse Ti2-E microscope (Nikon, Japan) equipped with an Apo TIRF 60× oil-immersion objective (numerical aperture: 1.49) together with a Lumencor SPECTRA III light source, a multi-band filter cube 86012v2 DAPI/FITC/TxRed/Cy5 (Nikon), a Ti2-LAPP laser system, and a Prime 95 B sCMOS camera (Photometrics Teledyne, USA) was used to perform all TIRF experiments.

Single-particle kinetics and equilibrium fluctuation analysis (EFA) was as follows: for binding kinetic data, time-lapse videos (129 × 129 μm^2^ field of view) at a frame every 15 s for 1 h were recorded with labeled HPV16 particles after 75 min preincubation to reach equilibrium unless otherwise stated. This experimental time frame was deemed to be a good compromise between time resolution and photobleaching of the particles, while allowing observation of virus binding events at timescales of relevance to the infection process.

The time-lapse movies were automatically analyzed using an in-house MATLAB script.^8^ Briefly, particles were detected in all frames using a 2D peak detection algorithm. Only particles exceeding a user-defined prominence threshold were considered. Particles were then linked between frames using a nearest-neighbor approach with a maximum linking distance of 2 pixels. According to their fluorescence intensity and permanence on the surface, particles were categorized as either “firmly bound” (residence time more than 3 frames), “loosely bound” (residence time less than 3 frames), or “bleached” (slow and steady decrease in intensity below the detection threshold with no sudden drop suggesting detachment).

Loosely bound and bleached particles were disregarded in the analysis, and only detachment of particles that landed onto the surface in the first half of each video was considered. To improve statistics, we measured the cumulative value of 5 individual movies corresponding to 5 randomized locations.

The particle association kinetics to the GAG surface can be determined by considering the cumulative sum of the detected arrival events over time. In particular, under equilibrium conditions, i.e., in absence of global diffusion limitations, the slope of the cumulative sum can be related to the particles’ association rate constant (*k*_on_) using the following expression:

(1) $$\frac{dN^{+}}{\mathrm{dt}}=k_{\text{on}}C_{b}\theta$$

Here, *N*^+^[cm^−2^] is the cumulative number of particles landed since the beginning of the observation period, *C*_*b*_ [M] is the virus bulk concentration, and *θ*[cm^−2^] is the surface density of free receptors on the surface. It is important to note that determination of a value for *k*_on_ in the complex case of an HPV16 particle binding to GAGs is complicated by the fact that the receptor cannot be unambiguously identified. Here, we assume that each GAG chain is a single receptor. Additionally, in our experiments, the number of grafted heparin chains exceeds bound particles by >10^4^ (>2 × 10^7^ heparin chains and <10^3^ bound particles per field of view). The number of occupied receptors is thus negligible, and we can ignore saturation effects and approximate *θ* to the total GAG grafting coverage (Table 1). Furthermore, because of a well-recognized artifact associated with difficulties in accurately identifying all particles initially present on a crowded surface, the first 150 s were discarded from the linear fit.

**Table 1:** Surface grafting densities and average spacing between GAG chains for different GAG surfaces used in the study

GAG:PEG surface molar ratio	Mass density^a^ (ng/cm^2^)	Grafting coverage (10^11^/cm^2^)	Spacing between chains (nm)
Heparin:PEG:1:5	2.8 ± 0.7	1.1 ± 0.3	29.9 ± 3.7
2-O-deS:PEG:1:5	3.2 ± 0.7	1.3 ± 0.3	27.7 ± 3.2
6-O-deS:PEG:1:5	3.8 ± 2.2	1.5 ± 0.9	25.6 ± 7.4
N-deS:PEG:1:5	3.9 ± 1.0	1.6 ± 0.4	25.2 ± 3.3
Heparin:PEG:1:1	11.3 ± 3.1	4.5 ± 1.2	14.8 ± 2.0
N-deS:PEG:1:1	16.0 ± 4.5	6.4 ± 1.8	12.5 ± 1.8
N-deS	94.9 ± 23.4	38.1 ± 9.0	5.1 ± 0.6

Values ± standard deviation were obtained from 2 independent experiments except HA.

a Grafting density values were calculated from SPR data (Table S3) with the factor of 24 ± 3 for 1:5 and 6 ± 1 for 1:1 obtained from fluorescently labeled biotinylated GAG assay.

In addition, the particle residence time distribution was used to calculate the cumulative survival probability of the bond to the surface, i.e., the percentage of particles still bound to the surface at any time from attachment. This was then fitted to a single exponential function to determine the particle’s dissociation rate constant (*k*_off_):

(2) $$f\left(t\right)=A\left[{\left(1-B\right)e}^{-k_{\text{off}}t}+B\right]$$

where A is the initial value, and B represents the fraction of particles that appeared to be irreversibly bound to the substrate in the time frame of the experiment**.** Both A and B are fitted parameters.

### Single-particle tracking of virus particles

To image and track landing HPV16, diluted HPV16 particles were added at concentration of 1.14 pM (or 0.0228 μg/mL) on the GAG surfaces. Timelapses of the landing particles were recorded at room temperature without rinsing and immediately after virus addition to capture the possible motion of the particle while still in the process of establishing bonds. Movies were recorded either at an acquisition rate of 1 s per frame for a total duration of 20 min, or at 10 s per frame for a total duration of 25 min. Recorded movies were processed to remove spatial drift and analyzed using Trackmate^36^ and MATLAB DC-MSS (Divide-and-Conquer Moment Scaling Spectrum)^37^ transient diffusion analysis as described in Abidine et al., 2022.^38^ First, sample drift was removed using an in-house MATLAB script that performs fast Fourier transform-based frame registration^39^ and generates drift-corrected movies. The resulting movies were then analyzed using TrackMate. The virus particles were detected with sub-pixel localization in each frame, and tracks were reconstructed using a maximum frame-to-frame displacement of 0.8 μm and maximum gap of 0.5 μm and 3 frames. The resulting tracks were further processed in MATLAB to correct for residual sample drift by identifying and removing collective motions common to all tracks, discarding trajectories that switched between two particles, and filtering out single-point outliers within individual tracks.

To have a better understanding of the transient diffusion of HPV16 on GAG surfaces, the trajectories were further segmented and classified in MATLAB according to the DC-MSS algorithm, as detailed in Abidine et al., 2022.^38^ In brief, for each trajectory, a rolling-window of 21 frames was used to calculate the time-dependent moment scaling spectrum (MSS) and tracks were segmented according to the MSS slope: a slope <0 represented immobile particles, while a slope between 0 and 0.5 implied a confined motion, a slope of 0.5 indicating free diffusion, and a slope >0.5 directed motion. After the first classification, adjecent segments with the same motion type were pooled together, and classification was calculated once more on the new segments. Finally, diffusion properties, such as the diffusion coefficient (*D*) and confinement radius (*R*_*c*_), were calculated for each segment, as well as the time spent in one motion type. The signal to noise ratio (SNR) in all movies was calculated as follows. First, the background was isolated from the particle signal by manual thresholding in each frame. The resulting mask was then eroded by three pixels to remove possible remaining signal from the particles, and the background mean intensity (*I*_bkg_) and standard deviation (*σ*_bkg_) were calculated for each frame and averaged over the whole movie. The particle intensity (*I*_*p*_) was calculated by applying a Gaussian blur (*σ*_Gaussian_ = 1 pixel) to each frame and measuring the maximum value in a 5 × 5 pixel area around the detected position of each particle at each frame. The intensity was then averaged for each particle over the whole trajectory. The SNR was calculated for each particle as SNR=Ip−Ibkgσbkg.

### Estimation of the maximum number of bonds per virion

In our platform, the concentration of heparin on the surface is maintained at σ = 1.1 to 1.6 ×10^11^ molecules/cm^2^ for all heparin species (Table 1) and is ∼24 times lower than saturated coverage for experimental conditions tested here (Table S3). For this reason, heparin does not form a continuous layer; instead the chains are distributed randomly with an average distance of ∼27 nm. This results in areas of high concentration and locally depleted areas. The number of molecules available for binding for a single virion thus varies depending on the landing position. The probability distribution of the number *n* of available heparin chain in a contact area, *A*, follows a Poisson distribution:

(3) $$P\left(n,\sigma A\right)=\frac{{\left(\sigma A\right)}^{n}e^{-\sigma A}}{n!}$$

In particular, the probability that the area contains at most *n*_*max*_ molecules is

(4) $$P\left(n\leq n_{\max}\right)=\sum_{i=0}^{n_{\max}}P\left(i,\lambda A\right)$$

The contact area depends on the radius of the virion, but also on the height of heparin chain above the PEG layer and the length of the minimal binding motif, which, based on previous studies,^29^^,^^40^ we consider to be four disaccharides (Figure S7A). The Flory theory for real polymer chains was used to calculate the extension of heparin and PEG. The following parameters were used for heparin: Kuhn length (b): 9 nm, diameter exclusion volume (d): 0.5 nm, contour length (L_c_): 25 nm, and σ = 1.1–16 ×10^−3^ nm^−2^. For PEG, b = 1.4 nm, d: 0.3, L_c_ = 23 nm, and σ = 0.07 nm^−2^. A detailed description of the model and calculation performed is presented in Section S11 of the supplemental information. The resulting heparin height was calculated to be between 15.1 and 16.3 nm, depending on the sulfation. Approximating the virion to a sphere of radius 25 nm, this results in an interaction area between 1,650 nm^2^ and 1,730 nm^2^, respectively.

### Multivalency-aided diffusion simulations

We modeled the diffusion of particles on surface-grafted binders originating from the establishment of transient multivalent bonds using in-house MATLAB scripts. We consider randomly distributed binders with a defined concentration and initialize the system with a single particle bound to a single binder on the surface. The particle is then allowed to create new bonds with any of the binders within its interaction area and break existing ones with probability *p*_*r*_ = 1 − *e*^−^^*r*/*dt*^, where *r* is either the attachment or detachment rates (*r*_on_ and *r*_off_). The position of the particle is determined at each iteration as the center of mass of all the engaged binders. If the particle has no bonds with the surface, it is interpreted as detached from the surface, and the simulation is ended. We normalized the binder concentration to the number of binders in the interaction area and the *r*_*on*_ to the *r*_off_, in order to generalize the results to all particle-surface interactions. We considered 35 logarithmically distributed values of ${r_{\text{on}}/r}_{\text{off}}$ between 0.02 and 10,000 and 25 binder concentrations between 0.1 and 100. All conditions were simulated 100 times for 500 timesteps, with a time interval *dt* = 0.1/*r*_off_. Example tracks are shown in Figures S15C–S15F. We analyze the resulting tracks by considering the total path length, maximum displacement, average number of bonds, average track duration, average particle speed, hull area and radius of the trajectory, and estimated diffusion coefficient. Additionally, we segmented the tracks in 4 possible motion types (immobile, confined, free diffusion, and direct motion) as described for the analysis of the experimental tracks and shown in Figure S15A. The average motion time was determined by assigning a score from 0 (immobile) to 3 (directed) to each motion time and averaging the score over the track, weighted by the time spent in each mode, as described in Becker et al.^41^ Additional details on the track analysis and the incorporation of the particle Brownian motion are described in Section S15 of the supplemental information.

## Results

### Heparin and its derivatives have the same grafting density on the glycocalyx mimic platform

In this study, we implemented a glycocalyx mimic platform, which allowed us to study the interactions between viruses and GAGs in a well-controlled experimental environment. This platform is based on the immobilization of GAG chains by end-grafting through a biotin-tag at their reducing end, as illustrated in Figure 1A. Moreover, streptavidin was cross-linked using paraformaldehyde. This prevented GAG chain diffusion laterally on the bilayer (Figure S1; Section S3 in supplemental information) while preserving streptavidin’s ability to bind biotinylated GAGs (Figure S2; Section S4 in supplemental information).

In this study, we use heparin as a representative of the sulfated domain of HS, as it is commonly used instead of HS. Moreover, sulfation patterns of heparin are more predictable and lack the highly sulfated or nearly desulfated macrodomains characteristic of HS. This reduced structural heterogeneity, along with ease of production and high purity,^42^ facilitates clearer interpretation of the role of individual sulfation types, which is the focus of this work. More specifically, we used parental heparin as well as its chemically desulfated derivatives missing either 2-O, 6-O, or N-sulfations (2-O-deS, 6-O-deS, and N-deS, respectively). HA, a naturally occurring negatively charged nonsulfated GAG, was used as a negative control (Table S1). Where required, we used PEG as an inert spacer to control the GAG chains’ grafting coverage.

SPR was used to follow the step-by-step assembly of GAG films and to quantify the amount of surface-bound GAG molecules on the streptavidin monolayers. A representative time-resolved SPR sensogram for a heparin film is shown in Figure 1B, while Table S3 provides the shift in SPR angle at saturated binding for all 5 GAG films, together with the corresponding calculated grafting density and average spacing between adjacent GAGs. Further details on the calculation are provided in the supplemental information (Section S5). These data indicate that heparin and all its derivatives yield similar SCs on the streptavidin layer.

For TIRF experiments in this study, we used a mixture of GAGs and PEG at molar ratio of 1:1 or 1:5 to alter the grafting coverage of the GAG chains. However, the presence of two biotinylated species complicates the SPR analysis as only the total adsorbed mass is detected, not the relative amounts. We estimate the reduction in GAG density from the saturation values by measuring the fluorescent intensity of fluorescently labeled N-deS heparin immobilized on SLBs (FluoN-deS, Figure 1C; S3B; Section S6 of supplemental information). We determined a reduction of 6 ± 1 and 24 ± 3 for 1:1 and 1:5 FluoN-deS:PEG molar ratio, respectively. The estimated mass density, grafting coverage, and spacing between chains for all heparin derivatives and GAG:PEG mixtures employed in this work are summarized in Table 1. Overall, we observed a similar reduction by immunostaining of immobilized parental heparin (Figure S4), indicating that the grafting coverage is independent of the heparin sulfation.

### N-sulfation of heparin primarily mediates the attachment of HPV16 particles to the glycocalyx mimic

**Figure 2:**
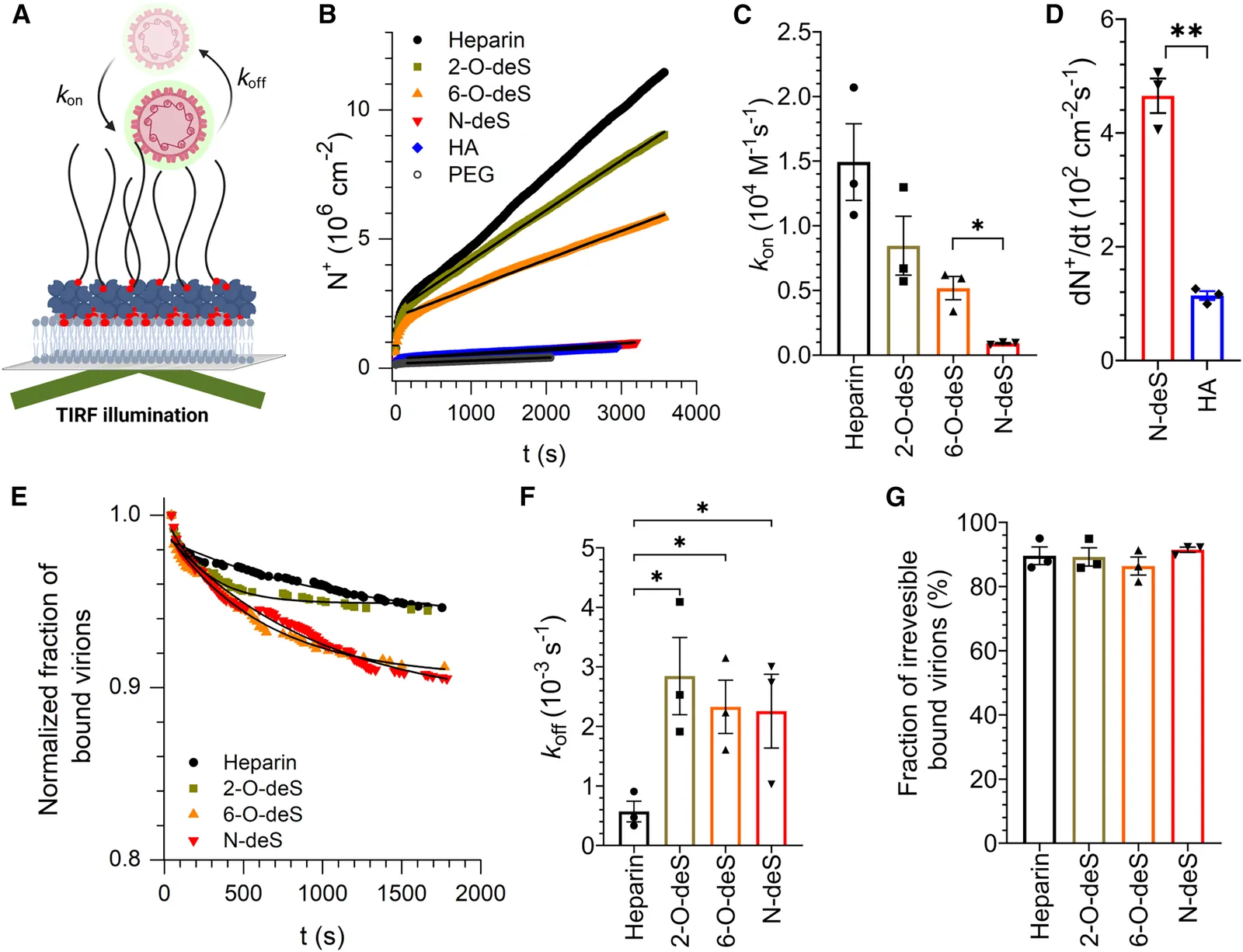
Kinetics of binding between HPV16 particles and GAG. (A) Schematic representation of TIRF microscopy assay to probe the interactions between HPV16 on immobilized GAG:PEG (1:5) surface. Surfaces consisting of a mixture of GAG and PEG were prepared on an SLB-bound streptavidin layer. The binding of HPV16 particles labeled with Alexa Fluor 488 was detected using TIRF microscopy. (B) Representative curves showing the cumulated number of newly bound (since t = 0) particles per unit area (N^+^) as a function of time (t). The black lines represent fits to a linear function, where the first 150 s are discarded from the fit. (C) Association rate constant (*k*_*on*_). (D) New particle arrival rate (*N*^+^/*dt*) for N-deS and HA showing significant increase in binding over HA control. (E) Fraction of HPV16 remaining bound as a function of time. The black lines are best fits to a single exponential decaying function with an offset. The curves are normalized to 1 at t = 45 s (F and G) Dissociation rate constants (*k*_*off*_,*F*) and fraction of irreversibly bound particles (G), obtained from the decaying function fit of the data in (C) for each condition. Column bars represent means, and error bars represent SEM. Each black symbol represents an independent experiment. Statistical significance is calculated using Welch’s *t* test. ^∗^ = 0.02, ^∗∗^ = 0.006, ns = nonsignificant.

To assess the influence of the different sulfate groups on the overall interaction kinetics of individual HPV16 particles with biomimetic heparin films, we used TIRF-based SPT and EFA.^8^^,^^9^^,^^43^^,^^44^ Here, time-lapse movies of fluorescently labeled HPV16 binding to and detaching from the model GAG surfaces are recorded, as schematically shown in Figure 2A. The selected experimental time window for the EFA experiment allowed the observation of particles with a residence time ranging from 45 s to 30 min, i.e., in the order of the expected timescale of the HPV16-HS interaction at the cell surface during binding and the initial stages of structural activation.^31^^,^^32^ Under steady-state conditions, i.e., in absence of global diffusion limitations, the arrival rate of new particles is proportional to the association rate constant of the virus particle to the GAG surface (*k*_on_) (Equation 1). Additionally, the residence time of the viruses on the surface yields the dissociation rate constant of the particle (*k*_off_) (Equation 2). The model GAG surfaces used for EFA were prepared with a mixture of GAG:PEG chains at a molar ratio of 1:5 as described earlier. In these conditions, the distance between two adjacent GAG chains was estimated to be between 25 and 30 nm (Table 1). This distance is about half the diameter of a single virus (∼50 nm), which allows, in principle, the formation of multivalent bonds with neighboring GAG chains (see supplemental information, Section S11).

Figure 2B shows the cumulative number of newly bound HPV16 particles per surface area (*N*^+^) over time on four differently sulfated HSs and the two negative control model surfaces (HA and PEG), while Figure S5A and Table 2 present the values of the slope obtained by linear fit. The linear increment of *N*^+^ versus time (Figure 2B) and the limited time-dependence of the virus SC (Figure S5B) are consistent with equilibrium conditions. It must be noted that in the case where the ligand of interest is immobilized on a larger particle, as in the case of the binding of HPV16 capsomers to heparin, the slow diffusion of the whole particle might limit the arrival rate of the ligand to the cell-surface receptor. In this case, even though an equilibrium without net mass transfer is established, the *k*_*on*_ of the ligand-receptor interaction is not experimentally probed. Here, it is thus important to compare the experimental arrival rate to what is expected by sole diffusion of the particles in solution to ascertain that we are indeed probing the characteristics of the capsomer-heparin interaction. In our case, we calculated that the arrival rate in all conditions is at least one order of magnitude lower than was expected by diffusion (supplemental information, Section S7), confirming that the experiment probes the HPV16-GAG bonds in reaction limit conditions.

**Table 2:** Summary of association rates and association rates per GAG chain on GAG:PEG model surfaces

GAG surfaces	Arrival rate (N^+^) (10^15^ cm^−2^M^−1^s^−1^)	***k***_on_ (10^4^ M^−1^s^−1^)	***k***_off_ (10^−3^ s^−1^)
Heparin:PEG	1.67 ± 0.60	1.49 ± 0.5	0.57 ± 0.3
2-O-deS:PEG	1.10 ± 0.51	0.85 ± 0.4	2.8 ± 1.1
6-O-deS:PEG	0.79 ± 0.24	0.52 ± 0.15	2.3 ± 0.77
N-deS:PEG^a^	0.14 ± 0.02	0.09 ± 0.01	2.3 ± 1.1
HA:PEG^a^	0.035 ± 0.004	/	/
PEG	0.07 ± 0.02	/	/

Arrival rate; k_on_= association rate constant, assuming that each GAG chain is an individual receptor; and k_off_ = dissociation rate constant.

a At 3 times more virus concentration in bulk solution.

The notably low arrival rate of particles to N-deS indicates that HPV16 mostly exploits N-sulfation to bind to heparin, although it appears that HPV16 is still able, to a limited extent, to interact with N-deS heparin (Figure 2D). This interaction is presumably mediated by other sulfate groups, or by the ∼8% residual N-sulfates, which are still present after the chemical de-sulfation process (see materials and methods and Section S16 of supplemental information for a discussion on the potential effect of residual sulfation on particle binding).

Under steady-state conditions, and when the number of occupied receptors is low, the arrival rate is proportional to *k*_on_ and to the number of receptors found on the cell surface (Equation 1). In the case of a virus binding to heparin, the estimation of *k*_on_ is complicated by the fact that the interaction is multivalent and that the receptor is not unambiguously identified. Since it has previously been reported that at least four disaccharide units are needed to establish binding,^32^^,^^40^ one GAG chain may in principle carry several virus-binding domains. Nevertheless, we estimate the association rate constant for the virus-GAG interaction (*k*_on_) from the measured arrival rate (Figure S5A), assuming that each GAG chain is acting as a single receptor (Figure 2C; Table 2). Our results show that removal of N-sulfates leads to a decrease in *k*_on_, by a factor ∼16 as compared with heparin. Removal of 6-O sulfates leads to a decrease in *k*_on_, by a factor ∼2, while removal of the 2-O sulfates leads to a decrease in *k*_on_, by a factor ∼1.5 as compared with heparin. Overall, these suggest that HPV16 favors the binding to sulfated GAGs, and that N-sulfates, in particular, play a key role in mediating a stable attachment of the virus to cell-surface HS, in agreement with our previous studies.^23^^,^^30^ This clear dependence of HPV16 binding on sulfate groups is also supported by data showing the surface coverage of particles bound to the respective surfaces (Figures S5C and S5D).

To gain insight into how the different sulfation groups influence the overall multivalent dissociation behavior of a single particle, we consider the residence time of individual HPV16 particles and report the fraction of particles still bound over time (Figure 2E). All plots showed a slow dissociation. Fitting the dissociation data with a single exponential decay with offset function (Equation 2; black solid lines in Figure 2E), as an approximation for the dissociation behavior, revealed that about 90% of the HPV16 particles were irreversibly bound (see materials and methods) in all cases (Figure 2G), meaning that they do not dissociate from the surface within the time frame of the experiment (30 min). For those particles that exhibited dissociation, we extracted the dissociation rate constants for the multivalent virus-GAG interaction (*k*_off_) from the fits. As presented in Figure 2F, no statistically significant differences in *k*_off_ values were found for the different desulfated heparins; however, parental heparin showed a significant lower *k*_off_ value in comparison to other desulfated heparin, indicating that all sulfates have a stabilizing effect on heparin binding. Comparing these results with our previous reports on monovalent capsomer-heparin bonds using AFM-SMFS,^30^ we observe much larger differences in *k*_on_ between heparin derivatives (a factor >15 instead of two to three for single bonds), while we fail to observe significant differences in the detachment rate, which instead spanned more than two orders of magnitude in the single-molecule case (see Table S4; Figure S6).

### Multivalent interactions stabilize the virus-receptor interaction: A theoretical framework

**Figure 3:**
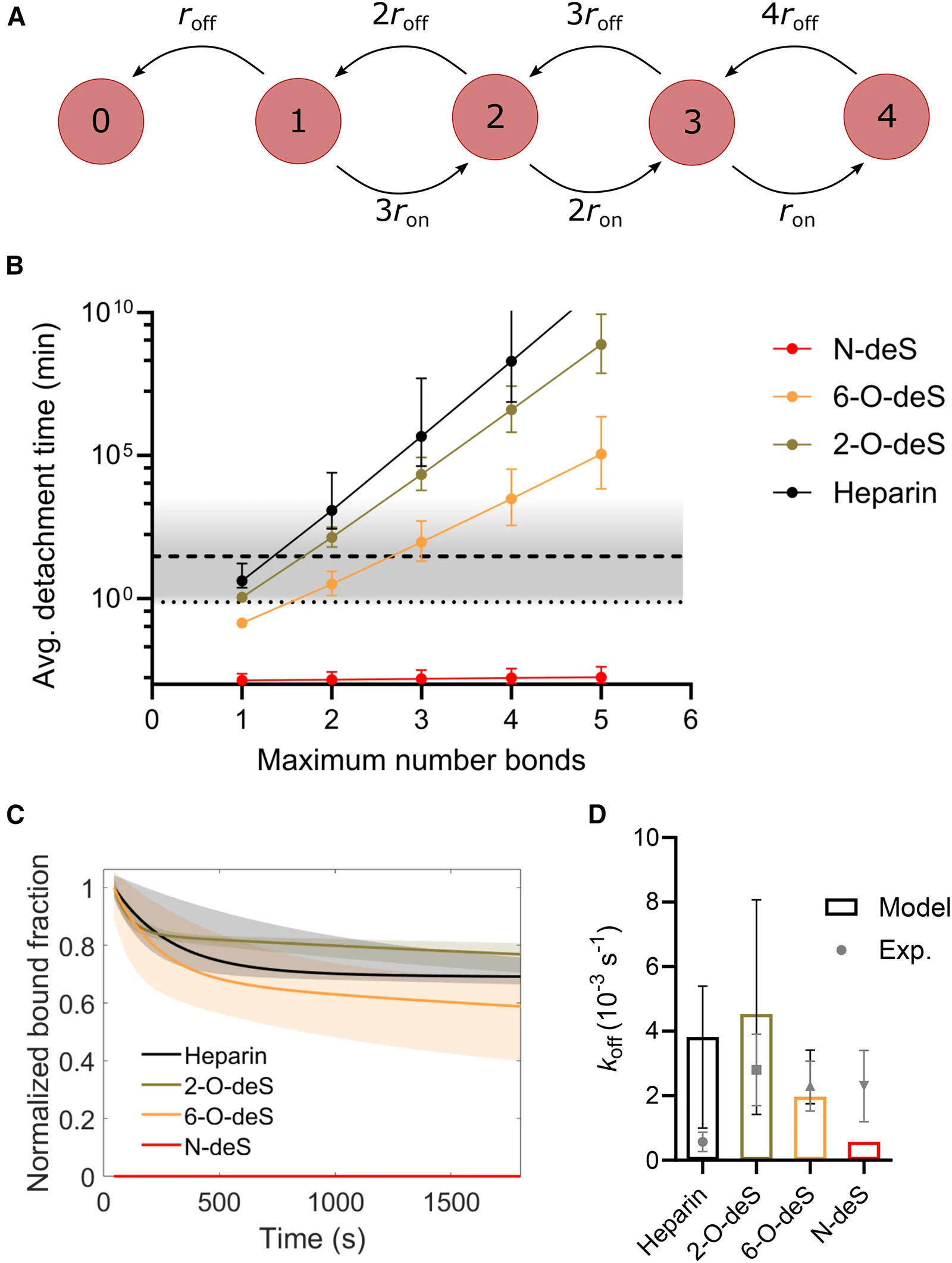
Markov theory to estimate the average dissociation rate. (A) Diagram of the continuous-time Markov chain that describes the multivalent binding of a particle considering possible creation of new bonds, at the example of a case with a maximum number of bonds of 4. The states are named after the number of bonds established with the surface, and the arrows indicate the possible transitions with their expected rate. (B) Average detachment time for HPV16 as a function of the maximum number of bonds they can establish with the surface-immobilized heparin. The dotted line indicates the minimum time a particle must stay on the surface to be detected as bound, and the dashed line indicates the duration of an experiment. The error bars indicate the values obtained using the confidence interval obtained in the AFM-SMFS measurements. The area between the two lines is the time frame in which our system can detect particle detachment, while the shaded gray area indicates the timescale of HPV16 internalization from first structural modifications upon HS binding (∼minutes) to entry (4–16 h). (C) Curves obtained by calculating the percentage of bonds remaining as a function of time using the values in (B) and the heparin grafting density. The solid line was obtained using the mean value and the shaded areas using the upper and lower limits of the bond lifetime. (D) Dissociation rate constants obtained by fitting the curves in (C) with the same function used for the experimental data. Error bars indicate the values obtained using the curves describing the edges of the shaded areas in (C). The gray symbols indicate the experimental results.

To investigate the origin of the dynamic behavior of individual particles establishing multiple interactions with a receptor-presenting surface, we propose a simplified model as a theoretical framework to relate the single-molecule and multivalent interaction kinetics. In this model, we assume that each heparin chain acts as a single binding site, i.e., a single chain can only bind one capsomer of the HPV16 particle. Similarly, an individual capsomer can only interact with a single heparin chain. Thus, the multivalent interaction between a particle and the substrate requires the binding of multiple heparin chains by an equal number of capsomers on the virus capsid (see Figure S7A). This assumption is reasonable, given that in the AFM-SMFS experiments published previously,^30^ the reported interactions were ascribed to a single HPV16 particle interacting with a single heparin chain, without observing, in most cases, multiple rupture events. Since we focus our interest on the binding behavior at the particle level, we abstract from the nature of the molecular interaction and refer to each interaction between a capsomer and a heparin chain as a single bond. Further, we consider that all heparin chains immobilized on the biomimetic surface are always accessible for binding. This aligns with the notion that the heparin chain moves much faster (∼100 ns) than the timescale for detachment, so the binding sites are accessible during the capsomer-heparin interaction (see supplemental information, Section S11) In this scenario, the number of possible bonds, i.e., the number of possible capsomer-heparin interactions, a single particle can form with the surface is thus limited by the number of heparin chains in its vicinity or the number of available capsomers. In the model, we further assume that all bonds are identical, that the individual bonds do not interact with each other, and that they can be independently formed and broken; i.e., they should be modeled as independent contributors rather than cooperatively producing a single energy barrier between the bound and unbound states. In such a case, the bonds are formed one at a time in a sequential manner. Consequentially, when a particle first attaches to the surface, only one bond is formed. Under such conditions, the interaction can be modeled as a continuous-time Markov process where states are solely defined by the number of bonds, and we only consider transitions between states that differ by a single bond, as depicted in Figure 3A. The association rate (*r*_on_), the rate at which a new single bond is created, can be readily calculated from the average binding time *τ*^SB^ previously measured for AFM-SMFS (Table S4). In the case of a particle bound to the surface, the interaction volume can be estimated as a cylinder having the height of the expected elongation of the grafted heparin during the particle residence time (h ∼16 nm, Table S5) and the radius of the base surface of a spherical cap of height h and radius of a virus particle (*R* ∼ 25 nm), *V*_*int*_ = *πh*^2^(2*R* − *h*) = 28 × 10^3^ nm^3^ (Figure S7A and supplemental information; Section S11). Note that in this case, the particle is not allowed to rotate freely, and the interaction volume only includes ∼23 capsomers (*n*_*c*_) on average. This contrasts to the AFM-SMFS case, where the particle is held in contact with the surface by the cantilever tip, and the estimated interaction volume includes the whole virus particle, with any of the 72 capsomers (*N*_*c*_) available for binding. To account for differences in number capsomers available for binding in the different experimental geometries, we adjusted the association behavior for the heparin derivatives as *r*_on_ = *n*_*c*_/*N*_*c*_*τ*^SB^ = 2.6 to 8.5 *s*^−1^ (Table S4). In addition, the association rate is proportional to the number of heparin chains available to the virus. For this reason, the transition between a state with M bonds to one with M + 1 bonds can be calculated as $r_{\text{on}}^{M\to M+1}=\left(M_{\max}-M\right)r_{\text{on}}$, where *M*_*max*_ is the maximum number of bonds that can be formed by a particle. In our experiments, given the heparin low grafting density, this corresponds to the number of heparin chains in the interaction volume (Figure S7).

As for the dissociation behavior, the transition rate between a state with M bonds and one with M − 1 bonds is proportional to M, since there are M bonds available for dissociation, and to the single-molecule dissociation rate ($k_{\text{off}}^{\text{SB}}=r_{\text{off}}=4\times 10^{-3}\;\text{to}\;12\;s^{-1}$) determined by AFM-SMFS (Table S4). Thus, $r_{\text{off}}^{M\to M-1}=Mr_{\text{off}}$. The rate of the possible transitions in the model system can be described with the following matrix:

**Figure:**
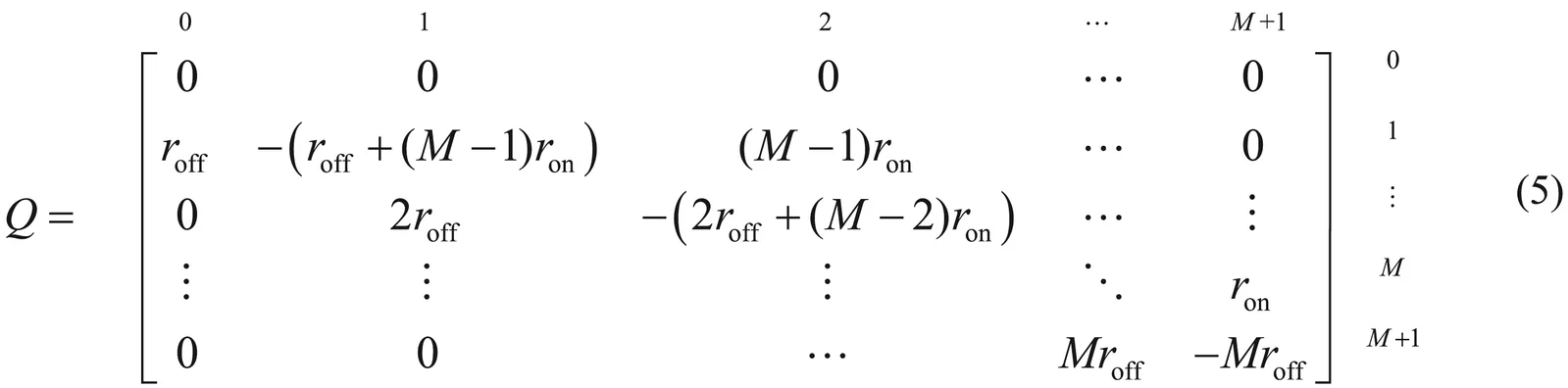

From this matrix, we can use Markov theory to estimate the average time needed to transition between the different states, as further detailed in the supplemental information (Section S10). In particular, we can calculate the time between attachment (1 bond) to particle release (0 bonds), using the following formula:

(6) $$t_{10}=-\frac{1}{q_{11}}+\sum_{j\neq 0}\frac{q_{1j}}{q_{11}}t_{j0}$$

where *q*_*ij*_ is the (i,j) element of the Q-matrix in Equation 5, and *t*_*j*0_ is the average time for the particle to transition from a state with j bonds to release.

In our AFM-SMFS experiments, the attachment rate was found to be between 1 and 3 orders of magnitude faster than the detachment rate for all heparin variants but N-deS^30^ (Figure S6). This means that after attaching, the particles will quickly occupy all the available binding sites and that all bonds must break almost simultaneously to prevent reattachment. The residence time of the particle thus strongly depends on the number of available binding sites for all heparin variants, as shown in Figure 3B. For parental and 2-O-deS heparin, the high affinity between heparin and HPV16 results in dissociation times for the particle longer than the duration of the experiment (dashed line in Figure 3B), suggesting that the detachment observed in the TIRF-based kinetic experiment is due to particles that only formed a single bond to the surface. In the case of 6-O-deS, instead, the average lifetime of a single bond is less than 10 s. This is more than 4 times shorter than the minimum residence time used in our experiment to define a bound particle (3 consecutive frames at 15 s per frame: 45 s, dotted line in Figure 3B) and indicates that particles bound through single bonds are typically not captured in our experimental setup. In contrast, particles interacting with two bonds exhibit an average residence time of ∼200 s compatible with experimental parameters and similar to what was observed for single bonds of parental and 2-O-deS heparin. This multivalent effect can explain the similar dissociation kinetics observed in TIRF experiments and provides an example of the stabilizing effect of multivalency in HPV16-cell interaction.

Combining the bond lifetime as a function of the number of bonds formed with the probability that a particle lands in a region where multiple bonds are possible, we calculated the survival probability of particles at the surface as a function of time and the dissociation rates predicted by the model (Figures 3C and 3D). The resulting curves qualitatively resemble those observed experimentally (Figure 2E), and the dissociation rate constants show good quantitative agreement with the experiments for both 2-O-deS and 6-O-deS. The model overestimates the dissociation rate for heparin, although the predicted value remains within the same order of magnitude as the experimental measurement. In addition, the predicted presence of a fast-dissociating population for 2-O-deS and 6-O-deS was tested by EFA at a higher time resolution of 1 fps, with the observation period limited to 15 min. As shown in Figure S9A, the experimental data are in very good agreement with the model predictions, with dissociation times comparable to those expected for the detachment of single bonds. However, the presence of short-lived, possibly nonspecific events limits the accuracy of the measurement (Figures S9B and S9C).

In the case of N-deS, much faster dissociation (*r*_on_/*r*_off_ ∼ 0.13) causes the lifetime of all particles, even those bound via multiple bonds, to be much shorter than what can be detected in our experiment. Indeed, the average lifetime of a particle bound through 5 attachment points is only ∼0.1 s, indicating that in our TIRF-based kinetic experiments, no particle binding should be detected. We speculate that the low, yet specific residual binding to N-deS (binding is still significantly higher than what observed for the nonsulfated HA) is due to residual N-sulfation in our sample, in which sulfation is reduced by 92% and not eliminated, as further discussed in Section S16 of the supplemental information.

In all cases, the model underestimates the irreversible fraction, i.e., the number of particles that remain on the surface for longer than the experimental observation time. The number of irreversibly bound particles is primarily determined by the number of available binding partners, as bond multiplicity rapidly stabilizes the interaction, as shown in Figure S10. We therefore consider the possibility that the model underestimates either the interaction area between the particles and GAGs or the GAG grafting density in the GAG:PEG complex mixture. Doubling the grafting density in the model indeed greatly improves the agreement between the model and the experimental data (Figure S10B).

### Diffusion of HPV16 on heparin surfaces is restricted

**Figure 4:**
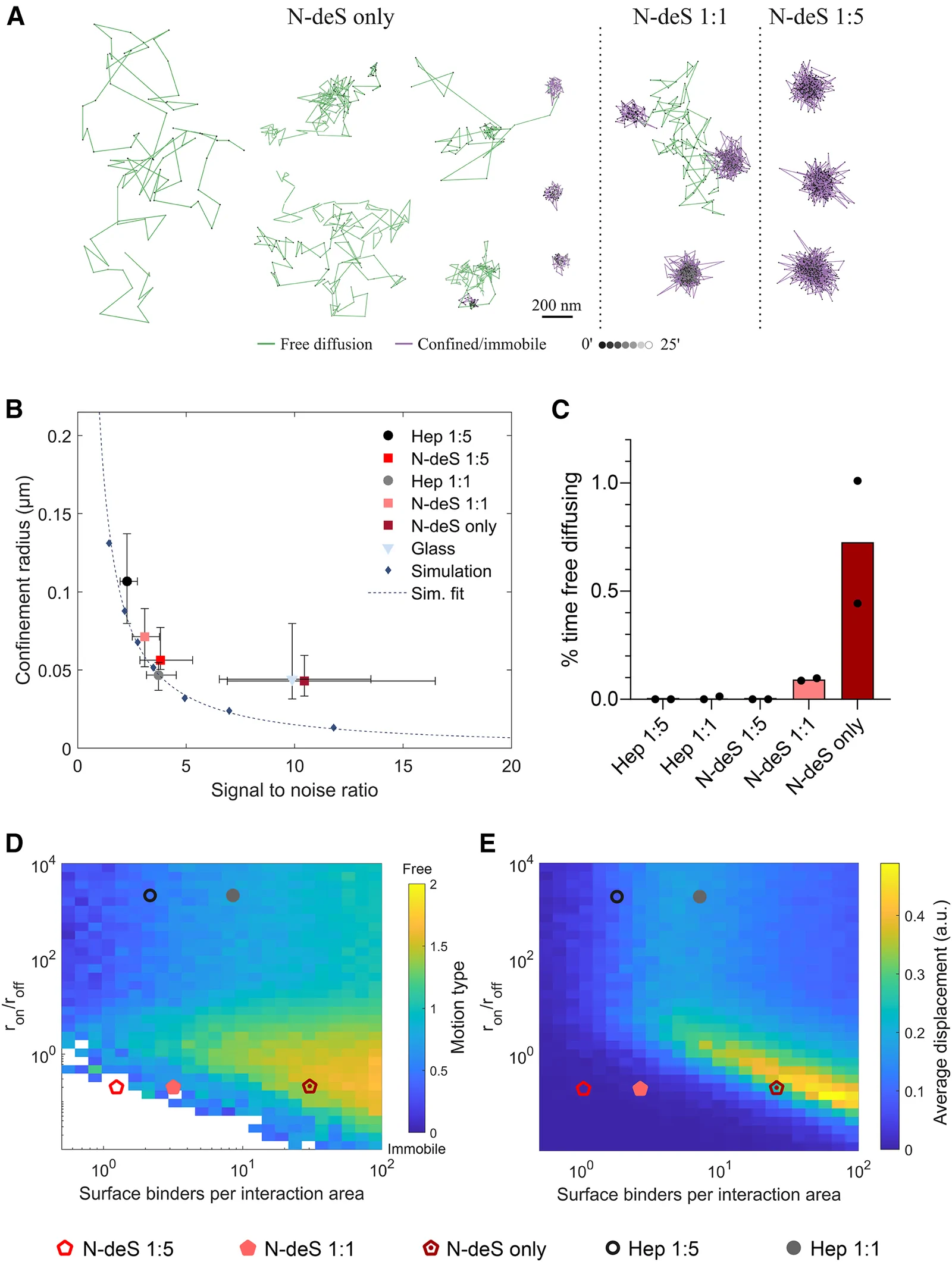
HPV16 diffusion on the GAG surface. (A) Example of experimental tracks of HPV16 particles diffusing on surfaces presenting different grafting coverage of N-deS heparin; from left to right: N-deS only: ∼38 × 10^11^ cm^−2^; N-deS:PEG 1:1: ∼6×10^11^ cm^−2^; N-deS:PEG 1:5: ∼1.6×10^11^ cm^−2^ (Table 1). Tracks are colored to distinguish between segments displaying free diffusion (green) and tightly confined/immobile segments (purple). Grayscale dots indicate the position of the particle at each frame and are colored to indicate the passage of time from black (0 min) to white (25 min). (B) Confinement radius of the detected particles as a function of the signal to noise ratio of the images used for single-particle tracking. The filled dots indicate the median value for each experimental condition (black: Hep:PEG1:5; gray: Hep:PEG1:1; red: N-deS:PEG1:5; pink: N-deS:PEG1:1; dark red: N-deS). Particles stuck on glass, in teal, were an immobile control. The error bars indicate the first and last quartile. The blue diamonds show the confinement radius obtained using our algorithm for synthetic timelapses of immobile particles. The dashed line is the best power fit: *R*_*C*_ = 0.215·SNR^−1.15^. (C) Percentage of time collectively spent by the HPV16 particles in free diffusion for parental heparin and N-deS heparin at different grafting densities. (D and E) Results of our multivalency-aided diffusion model of viruses on immobile randomly distributed surface binders. The heat maps, show (D) the average motion type (0: immobile, 1: confined, 2: free motion; see Becker et al.^41^) and (E) the average confinement radius as a function of the ratio between attachment and detachment rates (${r_{\text{on}}/r}_{\text{off}}$) and the number of binders available to the virus on average estimated through our simulations. White area indicates fewer than 10 tracks available for analysis. The circles and pentamers represent the various experimental conditions investigated in this work for N-deS and parental heparin, as indicated in the legend.

Diffusion of individual virus particles across GAG chains through exchange of individual ligand-receptor interactions^9^ may be an important mechanism by which viruses can move through the glycocalyx and on the cell surface, thereby playing an important role in virus accessibility to the cell surface for entry.^6^ Next, we thus asked whether the presence or absence of sulfate groups could influence the lateral diffusion of the HPV16 on the GAG layers, and in particular whether areas of low N-sulfation in the glycocalyx, while being ineffective at structurally activate the virus, could allow it to search the glycocalyx for areas of higher affinity, while maintaining contact with the cell surface. To address this question, we characterized the mobility of individual HPV16 particles using SPT on parental heparin and N-deS derivatives, the heparin derivatives that exhibit the highest and lowest affinity, respectively^30^ (Figure 2). Time-lapse movies were recorded immediately after adding fluorescently labeled HPV16 to the GAG surfaces, and single-particle tracks were extracted from the resulting movies^36^ (Figure 4A). The influence of the GAG concentration on the diffusive behavior was first assessed by measuring the diffusion of HPV16 particles on surfaces prepared at saturation coverage with pure GAGs as well as surfaces prepared from a mixture of GAG:PEG chains at molar ratios of 1:1 and 1:5. For these conditions, the spacing between individual GAG chains was estimated to be 5.1 ± 0.6, 13 ± 1, and 27 ± 2 nm, respectively. We analyzed the collected tracks using a segmentation algorithm^41^ to distinguish free diffusion from confined or immobile particles. In all conditions, we observed limited mobility with particles spending more than 99% of the total time tightly confined within an area comparable to a single pixel (Figures 4A and 4C and S12). The small differences in the values of the confinement radius (Figure 4B) and diffusion coefficient (Figure S13) observed between experimental conditions were found to be mostly a consequence of tracking noise, as shown by experiments tracking particles stuck on glass, and assumed to be immobile, as well as by simulations of immobile particles at SNRs comparable to the range observed in the experiments (see supplemental information, Sections S12 and S13). These controls confirm that, in our experiments, particle motion mostly falls below the resolution of our tracking techniques and is to be considered largely immobile. Similar results with low mobility were obtained with the other desulfated derivatives (data not shown).

In the absence of PEG dilution, i.e., in case of a saturated N-deS surface, we observed a notable increase in particles undergoing free diffusion compared with all other conditions with instances of free diffusion lasting for more than 10 min (Figure 4A). However, free diffusing events remain below 1% of the total recoded track duration (Figure 4C) and are too rare to confidently measure the diffusion coefficient or other mobility parameters. No free motion was observed for saturated parental heparin, probably due to the extremely stable bond established by HPV16, which prevents mobility.

In summary, HPV16 particles have limited capability to diffuse on heparin, even when most of the N-sulfates are removed, despite the profound effect of this modification on the association rate of the virus particle and on the dissociation rate constants of the single virus-heparin interactions.^30^

### Model of multivalency-aided diffusion

To further complement our experimental tracking results, we explored the condition needed for multivalency-assisted diffusion of the particle on the surface, i.e., motion of the particles on the surface presenting only immobile receptors, due to the creation and rupturing of bonds. We modeled the system considering that heparin chains are randomly distributed on the surface and immobile. In this simulation, the virus particle is initially bound to a single heparin chain and allowed to interact with neighboring chains within its interaction area. Upon the formation of new bonds, the particle moves to the center of mass of the bound chains, resulting in a particle displacement on the surface (see supplemental information, Section S15). We initially consider the bond as rigid and neglect the Brownian motion of the tethered particle. Since the motion of the particle only depends on how fast bonds are created and broken and on the number of binders available for binding, we generalize our model by normalizing for the ratio of the attachment and detachment rates (${r_{\text{on}}/r}_{\text{off}}$) and the number of chains within the interaction volume. In this case, time is also expressed as a function of the detachment rate, while space coordinates are rescaled into interaction area (*A*_*i*_) units, i.e., *t* = 1/*r*_off_ and $x=1/A_{i}^{2}$.

We varied the ratio between the attachment and detachment rate (${r_{\text{on}}/r}_{\text{off}}$) from 0.01 to 1,000 and the binder number per unit area between 0.5, for which the binding sites are so sparse that almost completely prevent multivalency, and 100 binders. The results of the simulations are shown as heat maps in Figures 4C and 4D, as well as in Figures S13 and S14. We observe the largest mobility in the presence of multiple weak bonds (bottom right corner in Figures 4C, 4D; and S13B), both in the type of motion (free diffusion versus confined or immobile particles), diffusion coefficient, and the area explored by the particle. This is expected as weak bonds allow the particle to move on the surface, while a large number of binders prevents detachment from the surface (Figure S15D). Strong bonds at low binder density (1–10 binders) results in long tracks and high average speeds, comparable to the high-multiplicity, low-affinity case; however, in this condition, the particle motion is strongly confined in a small region due to the inability to efficiently break already established bonds (Figure S15C). Mobility becomes extremely limited and largely insensitive to the bond affinity for ${r_{\text{on}}/r}_{\text{off}}$ larger than 10. This is due to the high binding affinity between individual ligand receptors, which encourages rebinding in case of bond rupture, effectively resulting in particle immobilization at the landing site (Figure S16).

The model does not make assumption on the particle size or absolute value of the binding and unbinding rates and can thus be easily applied to other systems and experimental conditions. However, it does not consider the Brownian motion of the particles tethered to the surface. For this reason, we tested if the addition of Brownian motion has a significant impact on the overall particle diffusion. Given that Brownian motion is much faster than the binding kinetics (ns to μs versus ms to minutes), we can assume that a particle tethered to the surface by one or multiple heparin chains will explore the space around the binding site. Effectively, the particle Brownian motion increases the interaction area beyond the particle radius. The average displacement of a particle is dependent on the number of tethering heparin chains, and it was estimated through simulations, as further detailed in the supplemental information, Section S15. This displacement was then added to the interaction radius to correct the interaction area used in the multivalency-aided diffusion simulation. The larger interaction area and thus availability of possible receptors results in an increased mobility at low binder concentration (Figure S19). This inclusion, however, did not modify the overall dependency of mobility on ${r_{\text{on}}/r}_{\text{off}}$ or receptor density, and, thus, any major conclusion is from the simpler, Brownian-free, model.

Our model indicates that surface diffusion on immobilized binders driven solely by the binding and unbinding of individual bonds in a multivalent interaction is only expected for low-affinity bonds, *r*_on_ of similar magnitude to *r*_off_, at high binder concentrations (bottom right corner in Figures 4D and 4E).

The model is in good agreement with our experimental results obtained with parental heparin. In this case, the highly stable single-molecule interaction (${r_{\text{on}}/r}_{\text{off}}$ ∼ 2 × 10^3^) strongly restricts the mobility of particles attached to the surface through multiple bonds. This results in a strongly confined motion around the initial binding site with a confinement radius of only few nanometers (considering an ∼1,700 nm^2^ interaction area) at any surface binder density. This motion is of the same order as the Brownian motion experienced by tethered particles and around an order of magnitude smaller than the localization precision in our experiments, explaining the absence of free diffusion observed for parental heparin. In the N-deS case, due to the lower affinity (${r_{\text{on}}/r}_{\text{off}}$ ∼ 0.13), the model predicts a sharp change in behavior at increasing binder concentrations (red symbols in Figures 4D and 4E) from short-lived confined motion to long-range mostly free diffusion. While an increase in mobility is observed in the experiments at high binder concentrations, and free diffusion is only present at GAG interchain distance of ∼5.1 nm, immobile or strongly confined particles remain the dominant population in all conditions (Figure S11). We hypothesize that this discrepancy is due to the imperfect chemical removal of N-sulfation from N-deS heparin. Indeed, the presence of low percentage of N-sulfates is statistically sufficient to lead to the formation of localized binding pockets capable of stably pinning the virus to the surface (see Figure S20, Table S6, and Section 17 in the supplemental information for further details).

## Discussion

In this study, we adopted an approach based on a glycocalyx mimic platform to study the interaction dynamics of individual HPV16 particles binding to multiple heparin chains, using TIRF-based SPT and EFA. The role of individual sulfate types was assessed using desulfated variants of heparin. The study, which tagetted multivalent interactions at the particle level, reveals that N-sulfates are essential to ensure initial association of an HPV16 virus particle to a layer of heparin molecules, with only marginal effects from other sulfate types. Our results further show that the type of sulfation has little influence on both the dissociation of particles from and their diffusion on the biomimetic heparin-presenting surfaces.

The kinetic studies of HPV16-heparin interactions on a single-particle level presented here expand and complement our previous study of HPV16-heparin interaction using AFM-SMFS. The AFM-SMFS study focused on probing the characteristics of “monovalent” heparin-HPV16 interactions, presumably involving the interaction between an individual capsomer and an individual heparin chain. Here, we employ TIRF microscopy and SPT to observe the binding behavior of whole virus and consider the effect of the creation of multiple bonds by the same virus.^30^ Together, both studies provide a more comprehensive understanding of the role of sulfation groups in modulating the multivalent binding of HPV16 to HS. Both studies agree that N-sulfation is crucial for HPV16 binding to cell-surface GAGs. However, for the case of a particle interacting through multivalent interactions, the type of sulfation primarily influences the arrival rate and *k*_on_, with a limited effect on *k*_off_. This stands in contrast with the single-molecule (monovalent interactions) investigations (Table S4) that reveal only a marginal effect of the type of sulfation on the association rate constant $(k_{\text{on}}^{\text{SB}}$) and rather point toward a key role of the N-sulfates in stabilizing the interaction by increasing the lifetime of the capsomer-heparin bond (decreasing $k_{\text{off}}^{\text{SB}}$). These differences reflect the formation of multiple bonds between the HPV16 particle and heparin chains while being also associated with the different experimental timescales of both techniques. Indeed, AFM is able to resolve transient and fast interactions, while TIRF-based kinetics focus on bonds lasting for minutes to hours, closer to what is observed during viral infection.^31^^,^^32^ Bridging this gap thus requires adequate modeling. To address this, we employed a continuous-time Markov model, in which bonds form and dissociate independently while the particle is in contact with the surface, and where the state of the particle is fully described by the number of bonds formed. The model reveals that for parental and 2-O-deS heparin, it is only possible to monitor the detachment of particles interacting with the surface through a single bond. Particles capable of forming multiple bonds with the surface have detachment times that are orders of magnitude larger than the experimental observation time and thus appear as irreversibly bound to the surface (Figure 3B). Accordingly, the relative difference in *k*_off_ between heparin and 2-O-deS is in very good qualitative agreement with the values of the $k_{\text{off}}^{SB}$ values reported by AFM-SMFS^30^ (Table S4). For 6-O-deS, the model predicts that particles forming only a single bond have a residence time around 5 times shorter than the experimental time resolution, making them undetectable in our experiments and suggesting that only particles that form at least two bonds can be observed attaching in the assay. Conversely, particles forming more than 3 bonds have an expected detachment rate lower than what can effectively measure in our experiments and will appear as irreversibly bound. Thus, the slow detachment observed for 6-O-deS in TIRF-based experiments can be attributed to the stabilization effect of multivalency, which makes the detachment rate comparable to those of monovalent bonds formed by HPV16 with the parental heparin. Additionally, our model predicts that the experimental window of the TIRF-based kinetic experiments does not allow us to visualize the binding of HPV16 to N-deS heparin, which aligns with the low attachment rates reported here. A second consequence of the high affinity of the HPV16-heparin interaction, except for the N-deS case, is that the particles, once the first bond is established, quickly form additional bonds to all heparin chains accessible. Thus, the number of bonds formed to the surface depends mainly on the number of heparin chains that can be reached by an already bound particle. While the heparin grafting coverage in our system guarantees an average of ∼2 heparins per particle, the random distribution of accessible surface-grafted heparin leads to areas of high heparin density and areas with one or no heparin chains available for particle binding (Figures S7B and S7C). By modeling the heparin chain as a semi-flexible polymer embedded in a PEG brush (see Section S11 in supplemental information for the full mathematical model), we determine that most particles reaching the surface (>70%) will land in vicinity of multiple heparin chains and are able to form more than one bond (Table S5). This contributes to the large portion (∼90%) of irreversibly bound particles observed for all heparin variants. This modeling also reveals that ∼30% of particles will form a single bond for the 6-O-deS case, which are undetectable in our TIRF-based kinetic experiments due to their short residence time (Figure 3B). We speculate that this could be the reason for a 65% ± 41% reduction in the *k*_on_ value observed between parental and 6-O-deS heparin (Figure 2C).

When it comes to the determination of the multivalent particle’s lifetime with our Markov model, it should be noted that this value is very sensitive to the input binding rate (*r*_on_) value. Indeed, *r*_on_ determines the rebinding probability of individual bonds while the particle is still attached to the surface, i.e., the time frame in which all bonds need to break for the particle to be released. An accurate determination of *r*_on_ is particularly challenging here, as it requires knowledge of the interaction volume, which is hard to predict.^45^^,^^46^ For example, the extension of the heparin chains in solution, the temporal availability of binding sites, as well as the mobility of the tethered particle all influence *r*_on_. Additionally, these parameters could slightly vary between the sulfation level and grafting densities of the different heparin derivatives. While such inaccuracies potentially limit our ability to resolve small differences, expected for example when comparing 2-O-desulfation to heparin, our analysis remains very helpful in the interpretation of the interaction and resolving the apparent mismatch between single and multiple bond behavior observed experimentally. Although the model reproduces our experimental data well at both short (1 fps for 15 min) and long (15 s per frame for 1 h) timescales (Figures 3C, 3D, and S9), its validity cannot be fully tested in the system investigated here. This is primarily due to the high affinity of the HPV16-heparin interaction, which remains highly stable even at a low number of bonds. For example, the model predicts an average detachment time of ∼7,000 h for a triple bond, which is experimentally inaccessible and of limited biological relevance. In addition, the strong dependence on N-sulfation, where the monovalent affinity of N-deS is more than three orders of magnitude lower than that of parental heparin, makes the system highly sensitive to residual N-sulfates in the N-deS sample. As a result, even small amounts of remaining N-sulfation can dominate binding and prevent reliable determination of multivalent kinetic parameters. Accurate validation of the model will require its application to systems with lower affinity and higher multivalency, which is beyond the scope of this work.

The kinetic investigations presented here further revealed that the *k*_on_ values determined in TIRF-based experiments are two orders of magnitude lower, as compared with the ones determined by AFM-SMFS, $k_{\text{on}}^{\text{SB}}$. This discrepancy is particularly surprising considering that, in most cases, we determined that the detachment events observed in TIRF experiments are from particles monovalently bound to the surface, and thus the same bond probed with AFM-SMFS. The determination of *k*_on_ via AFM-SMFS is strongly dependent on the determination of the interaction volume, which, as discussed above, is difficult to estimate as it depends on the specific configuration of the particle immobilized on the tip.^45^^,^^46^ Conventionally, this is estimated as the half sphere having the sum of the particle diameter and the polymer chain linking it to the AFM tip as a radius. However, this can lead to an overestimation of the volume, particularly in the case of a short linker, such as the one used in our previous AFM-SMFS study where the linker is estimated to be ∼2 nm in length (unstretched). Indeed, such a short linker will most likely greatly restrict diffusion and rotational freedom to the particle. Accordingly, a contact volume between the particle and the heparin layer, as the one used in the Markov analysis (Figure S7A), may lead to a more realistic estimation. In such a more conservative representation, the interaction volume is decreased by a factor ∼24, with an equivalent reduction of the measured $k_{\text{on}}^{\text{SB}}$. This illustrates that the discrepancies in experimental *k*_on_ values reported for the TIRF and AFM-SMFS assays may be explained by assumptions and limitations of the used models. Altogether, the theoretical framework developed in this paper emphasizes how the time window and multiplicity of virus-receptor bonds can drastically influence their binding kinetic characteristics. For example, we show that a substantial change in bond stability (i.e., bond lifetime) can manifest as an apparent variation in association kinetics when examined at the spatial and temporal scales of the virus attachment, rather than at the level of individual bond formation.

A second aspect of this study focuses on the two-dimensional diffusive properties of heparin-bound HPV16 by tracking the movement of fluorescently labeled particles after they landed on the heparin film, using TIRF-based SPT. We previously reported that HSV1 viruses bound to GAG films similar to those used here undergo lateral diffusion once recruited to the surface, presumably through exchange of individual bonds between GAG chains and viral glycoproteins.^9^ This observation inspired us to investigate whether similar behavior exists for HPV16. In this study, however, HPV16 diffusion was extremely limited. For parental heparin, only immobile particles were observed, independently of the heparin grafting density. On N-deS heparin, particle mobility was partially recovered with a small percentage of particles displaying slow but free diffusion at high N-deS grafting coverage (Figure 4). This trend agrees with the numerical model we propose to describe multivalency-assisted mobility on surface-immobilized binders. The model, however, predicts nearly free diffusion for most particles in the case of high SC of N-deS heparin. This discrepancy is probably due, as in the kinetic assay, to residual N-sulfation present in our samples. Altogether, we conclude that passive diffusion of HPV16 aided by multivalent bond is unlikely at the cell surface, due to the long bond lifetimes (small $k_{\text{off}}^{\text{SB}}$ values) reported for the interaction with parental heparin.^30^ Previous studies showed that HPV16 binds quickly and mostly irreversibly to cells via interaction with HSPGs in line with our model.^33^^,^^47^ HPV16 exhibits lateral mobility on the plasma membrane predominantly by constricted diffusion and to a minor extent by ballistic motion types.^33^ The relatively fast diffusive motion is likely the result of lateral diffusion of HSPGs or clusters thereof within the membrane, whereas ballistic motion is driven by actin-retrograde flow. Hence, this is in line with the conclusion of a lack of passive diffusion by binding and unbinding events. The current model of structural activation by glycan engagement indicates a preference for certain sulfation types on HS.^29^^,^^48^ On cells, HS instead of heparin is present. The highly dispersed HS is variably sulfated and exhibits sulfated domains interspersed with unsulfated domains. Hence, it is possible that while not contributing to passive diffusion, binding and unbinding events of single HS chains may help sampling of the sulfated domains for a best fit to achieve structural activation. Finally, it has been reported that structural rearrangements and further enzymatic processing of the capsid weaken the affinity with cellular HS.^49^^,^^50^^,^^51^ We hypothesized that these processes could, in a second phase, improve the local mobility of the virus at the cell surface and thereby contribute viral entry.

In conclusion, the theoretical framework presented here allows us to predict the dynamic behavior of individual virus particles interacting with multiple cellular receptors from the association and dissociation rates of the individual ligand-receptor bonds involved in the interaction. In this study, this framework was used to convey fundamental understanding of the attachment, detachment, and diffusion behavior of HPV16 particles in an HS-rich glycocalyx and of its dependence on the presence of specific sulfation types. Beyond confirming the key role of N-sulfation in mediating the interaction of HPV16 particle with HS, we were able to show that at timescales of relevance to the HPV16 entry, the attachment rate of the virus particle is primarily determined by the lifetime of the individual virus-glycan bond; the influence of the association rate constant of the single bond ($k_{\text{on}}^{\text{SB}}$) becomes negligible. We further explored the possibility that areas of low sulfation and high receptor density could allow for motion of particles on the surface presenting immobile cellular receptors, due to the creation and rupturing of individual bonds with cellular carbohydrates. We concluded that while such behavior is possible in the presence of abundant and weakly interacting binders, it could not be observed experimentally for the N-deS case, presumably due to the presence of residual N-sulfates on the N-deS heparin. This indicates that multivalency-aided diffusive behavior is unlikely to play an important role in initiating the recruitment of HPV16 to the cell surface.

## Data availability

All the data are available upon request to authors. Codes are available at https://github.com/darioconcaUMU/HPV_HSsulfation.

## Acknowledgments

We acknowledge the Biochemical Imaging Center at 10.13039/501100004885Umeå University and the National Microscopy Infrastructure, NMI (VR-*RFI* 2019-00217) for providing access to AFM and QCM-D. D.C. thanks the 10.13039/501100007601European Union’s Horizon 2020 research and innovation program under the Marie Skłodowska-Curie grant agreement no. 101027987 for the funding received. M.B. thanks the 10.13039/501100004063Knut and Alice Wallenberg Foundation, the 10.13039/501100004359Swedish Research Council (VR grants 2017-04029; 2020-06242; 2024-04375; 2024-02928), the 10.13039/501100007067Kempe Foundations, and the 10.13039/100014437Wenner-Gren Foundations (UPD2018-0193) for financial support. M.S. acknowledges the 10.13039/501100001659German Research Foundation (DFG, grants SCHE 1552/6-1, SCHE 1552/3-2, INST 211/1029-1, SFB1348/2 A09 to M.S.) for funding. Hudson Pace is acknowledged for the help with fluorescent labeling of GAG for the quantification of the adlayer’s GAG surface density.

## Author contributions

M.B. and M.S. conceived and supervised the project. F.B. performed the experiments and analyzed the data. D.V.C. developed the theoretical framework and wrote MATLAB code for analysis. Y.A., K.S., and D.V.C. analyzed the SPT data. L.S.M. prepared and labeled the virus samples. J.S. and A.D. assisted with SPR data acquisition and analysis. F.B., D.V.C., and M.B. wrote the original draft. All the authors reviewed and edited the manuscript.

## Declaration of interests

The authors declare that they do not have any competing interests.

## Supplemental information

Document S1. Figures S1–S20, Tables S1–S6, and Sections S1–S15

Document S2. Article plus supplemental information
