# Supplementary material for: Multivalency influences virus dynamics at the glycocalyx: A study of glycosaminoglycan binding in HPV16 entry
Source: Biophys J. 2026 May 12;125(14):3498–515. doi: 10.1016/j.bpj.2026.05.014 (PMC13507399; doi:10.1016/j.bpj.2026.05.014)
Supplement: Supplementary file 1 [file mmc1.pdf]

Biophysical Journal, Volume *125*

Supplemental information

Multivalency influences virus dynamics at the glycocalyx: A study of glycosaminoglycan binding in HPV16 entry

Dario Valter Conca, Fouzia Bano, Yara Abidine, Laura Soria Martinez, Kerstin Seier, Justas Svirelis, Andreas Dahlin, Mario Schelhaas, and Marta Bally

Supporting material

Multivalency influences virus dynamics at the glycocalyx: a study of glycosaminoglycan binding in HPV16 entry

Dario Valter Conca[a*], Fouzia Bano[a*], Yara Abidine[a], Laura Soria Martinez[b], Kerstin Seier[a], Justas Svirelis[c], Andreas Dahlin[c], Mario Schelhaas[b], Marta Bally[a,d†]

[a]*Department of Clinical Microbiology, Wallenberg Centre for Molecular Medicine and Umeå Centre for Microbial Research, Umeå University, Umeå, Sweden*
[b]*Institute of Cellular Virology, ZMBE, University of Münster, Münster, Germany*
[c]*Department of Chemistry and Chemical Engineering, Chalmers University of Technology, Gothenburg 412 96, Sweden*
[d]*Science for Life Laboratory (SciLifeLab), Umeå University, Sweden*

*Contributed equally to this work
[†]Corresponding author

Contents

1. Supplementary reagents
2. Biotinylation of heparin and its derivatives
3. Measuring the lateral mobility of cross-linked streptavidin by FRAP
4. Characterization of the glycosaminoglycan (GAG) films by Quartz Crystal Microbalance with Dissipation (QCM-D)
5. Quantification of the optical mass of heparin by SPR
6. Estimation of the grafting coverage and chain spacing of a mixed layer of heparin and PEG
7. Additional Equilibrium Fluctuation Analysis data
8. Diffusion-controlled particle flux calculation
9. Summary of the AFM-based SMFS data
10. Markov-Model for multivalent binding
11. Probability of establishing multiple bonds with end-grafted heparin
12. Comparison between the Markov-multivalency model and experimental results
13. HPV16 diffusion on heparin film
14. Simulation of TIRF images of immobile particles

15. Model of multivalency aided diffusion
16. Effects of residual sulfation on particle binding and mobility
17. Discrete Markov model for heparin sulfation

1. Supplementary reagents

Reagents, proteins, lipids and buffer: Lyophilized fluorescently streptavidin with atto-488 (SAv-488; Sigma Aldrich, St. Louis, MO) was dissolved in milli-Q water at 1 mg/mL and stored at -20 ºC. Labelled lipids (1,2-dioleoyl-sn-glycero-3-phosphoethanolamine-N-(lissamine rhodamine B sulfonyl, 18:1 Liss Rhod PE; 810150, avanti polar lipids) were purchased from Sigma Aldrich. Lyophilized Bovine Serum Albumin (BSA) Fraction V was obtained from Roche, Germany, dissolved in milli-Q water at concentration of 10% (weight/volume, w/v) and stored at -20ºC. Anti-Heparan Sulphate (antiHS, clone F58-10E4) was obtained from AMSBIO, England. Both goat anti-mouse IgM secondary antibody, Horseradish peroxidase (HRP) conjugate (catalog #31440) and HRP substrate (SuperSignal™ West Femto Maximum Sensitivity, catalog # 34096) were from Thermo Fisher Scientific.

Phosphate buffered saline (PBS) buffer at pH 7.4 was used in all experiments; it was prepared by dissolving 1 PBS tablet (Medicago AB, Uppsala, Sweden) in 1 L milli-Q water (Millipore integral system, Molsheim, France) and filtered through 0.2 µm filters (Sarstedt, Germany).

2. Biotinylation of heparin and its derivatives

This study relies on commercially available heparin and three selectively desulfated heparins, whose properties are summarized in Table S1. The heparins were biotinylated via oxime ligation at their reducing end as described previously (1,2).

Table S1: GAGs used in this study

Name	Molecular weight (kDa)	n_{ds}§	Sulphate group per disaccharide#
Heparin	15	25	2.42
2-O-desulfated heparin (2-O-deS)	15	25	1.75
6-O desulfated heparin (6-O-deS)	15	25	1.48
N-desulfated heparin (N-deS)	15	25	1.55
Hyaluronan (HA)	23	57	0

§Values for number of disaccharide unit per chain were estimated from the average molecular weight of heparin.

#Average number of sulfate groups per disaccharide were calculated from disaccharides analysis provided by the supplier.

3. Measuring the lateral mobility of cross-linked streptavidin by FRAP

We tested the influence of streptavidin crosslinking with PFA, on the protein's lateral mobility by performing Fluorescence Recovery after Photobleaching (FRAP) measurements of fluorescently labelled streptavidin.

The detailed protocol used to immobilize GAGs on supported lipid bilayers though a streptavidin bridge is described in the main text. The concentrations and incubation times for each step for FRAP experiments were as following: SLBs of POPC:DOPE-Cap-B (90:10) and SLBs of Rhod PE:POPC with 1:99 molar ratio – 100 µg/mL, 30 mins; SAv-488 – 40 µg/mL, 60 mins; biotinylated chondroitin sulfate (bCSA, Aqua Biochem, Dessau, Germany) – 100 µg/mL, 30 mins; PFA – 2%, 15 mins. FRAP experiments were performed with an inverted Nikon Eclipse Ti2-E microscope using lasers with 488 nm and 565 nm wavelengths. Both lasers with 60% of total power were used for bleaching for 10 s. For recovery, a series of images consisting of 704 x 704 pixels with a 0.183 µm pixel width of was recorded at 0.5 fps with 10% of total laser power. The timelapse duration was 60 mins (30 mins for Rhod PE:POPC SLBs). The images were analyzed using ImageJ for intensity profiles. Our experiments indicate that the mobile fraction of labelled streptavidin is reduced from 57.5±3.5% (solid red line) to 5.5±0.3% (dashed black line) with PFA incubation (Fig. S1). Addition of the biotinylated GAG did not influence the streptavidin's diffusion behavior (Fig. S1, dashed red line. As a control, we used pure supported lipid bilayers composed of POPC:Rhod PE (99:1 molar ratio) whose lipids, as expected, exhibited nearly full recovery (96% mobile fraction, black line, Fig. S1). Two independent experiments were performed for each condition.

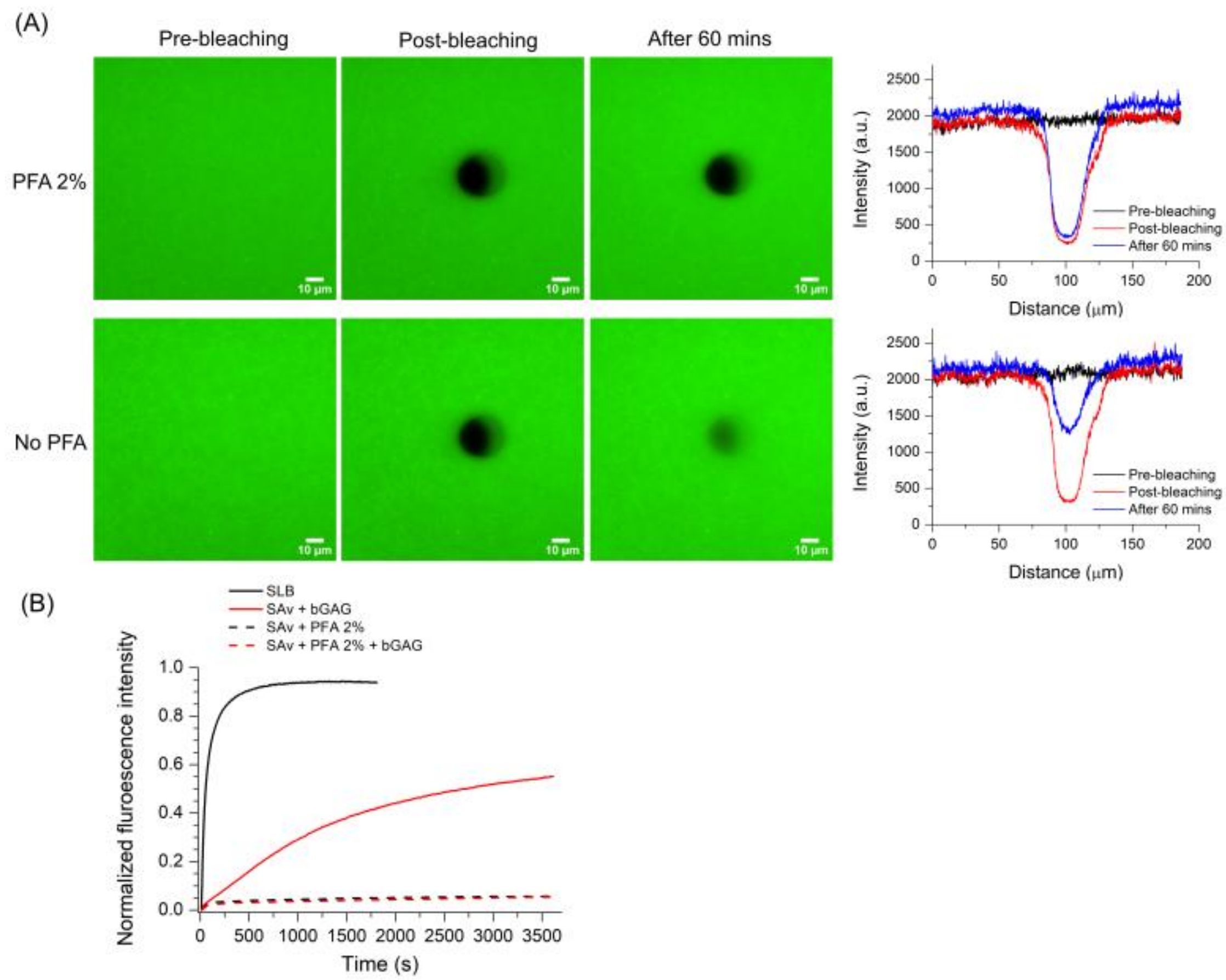

Figure S1. FRAP experiment to probe the lateral mobility of streptavidin before and after crosslinking with 2% PFA. (A) Representative images of fluorescently labelled streptavidin (SAv-488) on SLBs with 10% biotin with 2% PFA (top) or without. The images are taken before bleaching (pre-bleaching), right after bleaching (post-bleaching) and after 60 mins. The images are shown along with their intensity line profiles over the bleached area. Scale bar = 10 µm. (B) Intensity of the bleached area (taken from the line profiles) as a function of time are displayed to observe the recovery process after bleaching. Black solid line: control SLBs containing fluorescent lipids (Rhod PE); red solid line: SLBs containing 10% biotinylated lipids after exposing them with fluorescently labelled streptavidin (SAv-488) and addition of the biotinylated GAG (bGAG) chondroitin sulfate dashed lines: the SAv-488 was crosslinked with 2% PFA (dashed black line) following by addition of the bGAG (dashed red line).

4. Characterization of the glycosaminoglycan (GAG) films by Quartz Crystal Microbalance with Dissipation (QCM-D)

We used QCM-D to verify that the crosslinking of streptavidin with 2% paraformaldehyde (PFA) does not prevent the immobilization of biotinylated heparin (Fig. S2). QCM-D measures the shifts in resonance frequency (Δf), and dissipation (ΔD) of a sensor upon adsorption of molecules on its surface. The QCM-D signal is sensitive to the areal density

including the coupled water molecules and the mechanical properties of the surface-immobilized layer. Basically, a decrease in frequency corresponds to increased mass, while a high or low shift in dissipation corresponds to a soft or rigid layer, respectively.

QCM-D measurements were performed as previously described (3). Briefly, the QCM-D signal was collected using sensors with fundamental frequency of 5 kHz and silica coating (AWS SNS 000049A, Advanced Wave Sensors (AWSensors) S.L., Spain). Before use, the sensors were immersed in 2% (w/v) sodium dodecyl sulfate (SDS) for 1 hr, rinsed thoroughly with milli-Q water, blown dry with N_2, and treated in a UV/ozone cleaner (Bioforce Nanoscience, Ames, IA) for 30 min.

QCM-D data was collected with an X4 system (AWSensors, Spain) connected with four independent pumps. Buffers and biomolecule solutions (i.e., small unilamellar vesicles containing biotinylated lipids, streptavidin and biotinylated GAG e.g., heparin) were dispensed at a flow rate of 20 µL/min unless otherwise stated, at 22 ºC. The QCM-D response (frequency shift Δf and dissipation ΔD) was collected at six overtones (i = 3, 5, 7, 9, 11, 13) and data for f_i = 3 is presented, unless otherwise stated. Before use, PBS was degassed using a sonicator (Elmasonic, S40H, Singen, Germany).

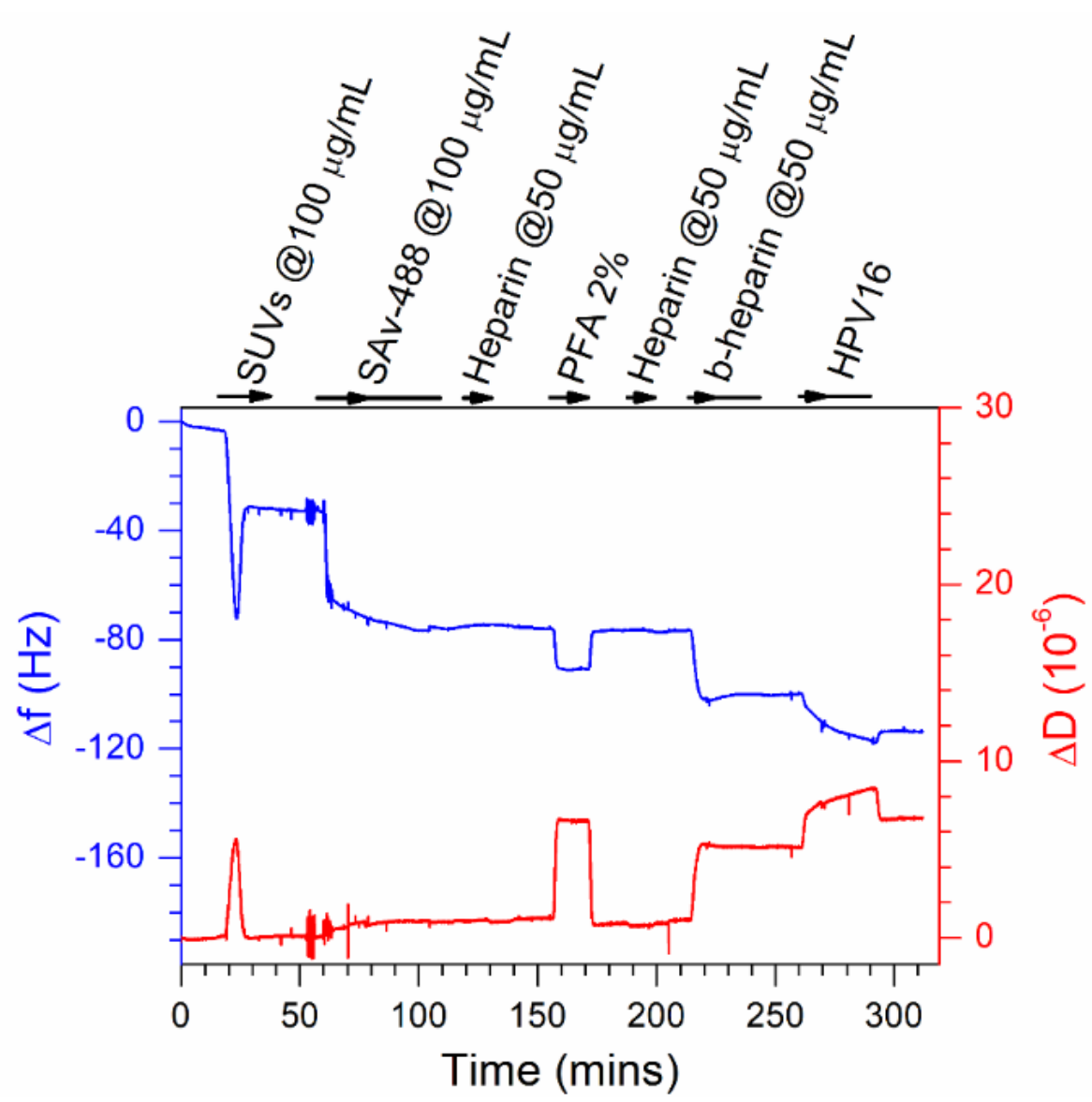

Figure S2. QCM-D experiments showing the build-up of a biotinylated heparin layer on a densely packed streptavidin layer. The shift in resonance frequency (blue) and the dissipation (red) associated with the binding of biotinylated heparin to a cross-linked densely packed streptavidin layer (Δf = -25.5 ± 2.8 Hz and ΔD = 4.4 ± 0.4 $\times 10^{-6}$) is close to reported values (3), supporting that the addition of 2% PFA did not have any effect on the immobilization of biotinylated heparin. Arrow indicates continuous flow of the

biomolecules. Line following an arrow indicates incubation with biomolecules without continuous flow (i.e., incubation in a still solution). Empty time slots represent rinsing with PBS under flow.

5. Quantification of the optical mass of heparin by SPR

To quantify the mass of adsorbed biomolecules per surface area or surface mass density (Γ) from the recorded SPR sensogram signal ($\Delta\theta$) presented in Figure 1B, we have used the reported expression (4):

$$\Gamma = \frac{\Delta\theta\delta}{S_0 dn/dc_m} \quad \text{(Eq. S1)}$$

Here dn/dc_m is the increase in refractive index per mass concentration of the binding species (see Table S1 for values of different biomolecules used in this study). S_0 is the bulk sensitivity of the instrument (angular shift per refractive index increment), which was estimated from Fresnel models at 670 nm to be 131 degrees (5). δ is the decay length of the evanescent field, which is calculated from the dispersion relation and the field distribution of the surface plasmons (6). Considering experimentally determined parameters for the Cr and Au thickness and permittivity, we have δ = 228 nm for our system.

Table S2: Refractive index increment per mass concentration (dn/dc_m) of biomolecules used in this study

	dn/dc_m (mL/g)
Heparin and its derivatives (2-O-deS, 6-O-deS and N-deS)	0.132[‡]
HA	0.16[‡]
PEG	0.134[§]

[‡]Values were taken from Altgärde et al. (7)
[§]Value was taken from Emilsson et al. (4)

Table S3: Summary of SPR angle shifts, surface mass densities, grafting coverages at saturation, and the average spacing between GAG chains for the different GAGs added at

a bulk concentration of 10 µg/mL. Values ± standard deviation were obtained from 2 independent experiments except for HA.

Name	SPR resonance shift at 670 nm (º)	Mass density (ng/cm^2)	Grafting coverage ($10^{11}/cm^2$)	Spacing between chains (nm)	Density of sulphate group ($10^{13}/cm^2$)
Heparin	0.051 ± 0.011	67.2 ± 14.9	27.0 ± 5.9	6.1 ± 0.7	16.3 ± 3.6
2-O-deS	0.056 ± 0.012	78.4 ± 15.8	31.5 ± 6.4	5.6 ± 0.6	13.8 ± 2.8
6-O-deS	0.070 ± 0.039	92.3 ± 52.2	37.1 ± 21	5.2 ± 1.5	13.7 ± 7.8
N-deS	0.072 ± 0.017	94.9 ± 23.4	38.1 ± 9.0	5.1 ± 0.6	14.8 ± 3.5
HA	0.113	122.9	32.2	5.6	/

6. Estimation of the grafting coverage and chain spacing of a mixed layer of heparin and PEG

We estimated the grafting coverage and chain spacing of a mixed layer of heparin and polyethylene glycol (PEG) using direct detection of labelled biotinylated GAGs (N-deS in this case) or an immunoassay.

Approach 1: Fluorescent labelled biotinylated GAG

Fluorescent labelling. bGAGs were fluorescently labelled by covalent conjugation to an amine-reactive dye. 50 µL of 2.5 mg/mL biotinylated N-deS heparin (biotinylated N-deS) heparin was first mixed with 7 µL of 1M $NaHCO_3$ (pH 9.0) buffer. Then 5 µL of Alexa Fluor™ 488 5-SDP Ester (Thermo Fisher Scientific) was added, mixed well, and incubated at room temperature for 1 hr, further mixing via vortex every 10 mins. The free dye was removed with 3 kDa molecular cut off spin column filters (Microcon YM-3, Millipore) by adding 100 µL of milli-Q water and centrifuging at 14,000 rcf for 40 mins until the flow through became clear (>7 times). We chose to label biotinylated N-deS as it maximizes the number of primary amines to react with the fluorescent dye. The concentration of labelled biotinylated N-deS was measured using the cetylpyridinium chloride (CPC) turbidity test as described elsewhere (3,8). Very briefly, 50 µL of CPC reagent (0.2% w/v of CPC and 133 mM of $MgCl_2$; both from Sigma) was mixed with 50 µL of GAGs solution in a 96-well cleared bottom plate (VWR). The solution was then carefully mixed to avoid bubbles and incubated at room temperature for 30 mins. The absorbance was measured at 405 nm using a spectrometer (Tecan, Infinite F200 PRO, Switzerland). At 405 nm, fluorophore

absorption was negligible. We also used QCM-D to validate the formation of a labelled biotinylated N-deS heparin layer on streptavidin. Despite a larger frequency shift and dissipation associated with the additional mass of the fluorophore, the data show that the softness of the heparin layer does not change after labelling, indicating that the mechanical properties of heparin are unchanged by the conjugation (Fig. S3A).

The use of fluorescently labelled GAGs to calculate the grafting density requires that fluorescence intensity of a single chain being independent from the grafting concentration, i.e. that no interchain quenching is present at high GAG concentration. To demonstrate that this is the case, we exposed surface-grafted FluoN-deS both at saturation and at 1:5 molar ratio bPEG:FluoN-deS, to focused 488 nm laser at 50% power for times between 0.1 and 60 seconds and recorded the difference in fluorescence signal before and after bleaching (Figs. S3C, D). At both concentrations, we observed a monotonic exponential decrease of the fluorescence intensity compatible with photobleaching of the illuminated area (Fig S3E). No dequenching, i.e., initial increase of the fluorescence signal, was observed indicating that no intra- or interchain quenching is present and the fluorophore emission is not influenced by the grafting density.

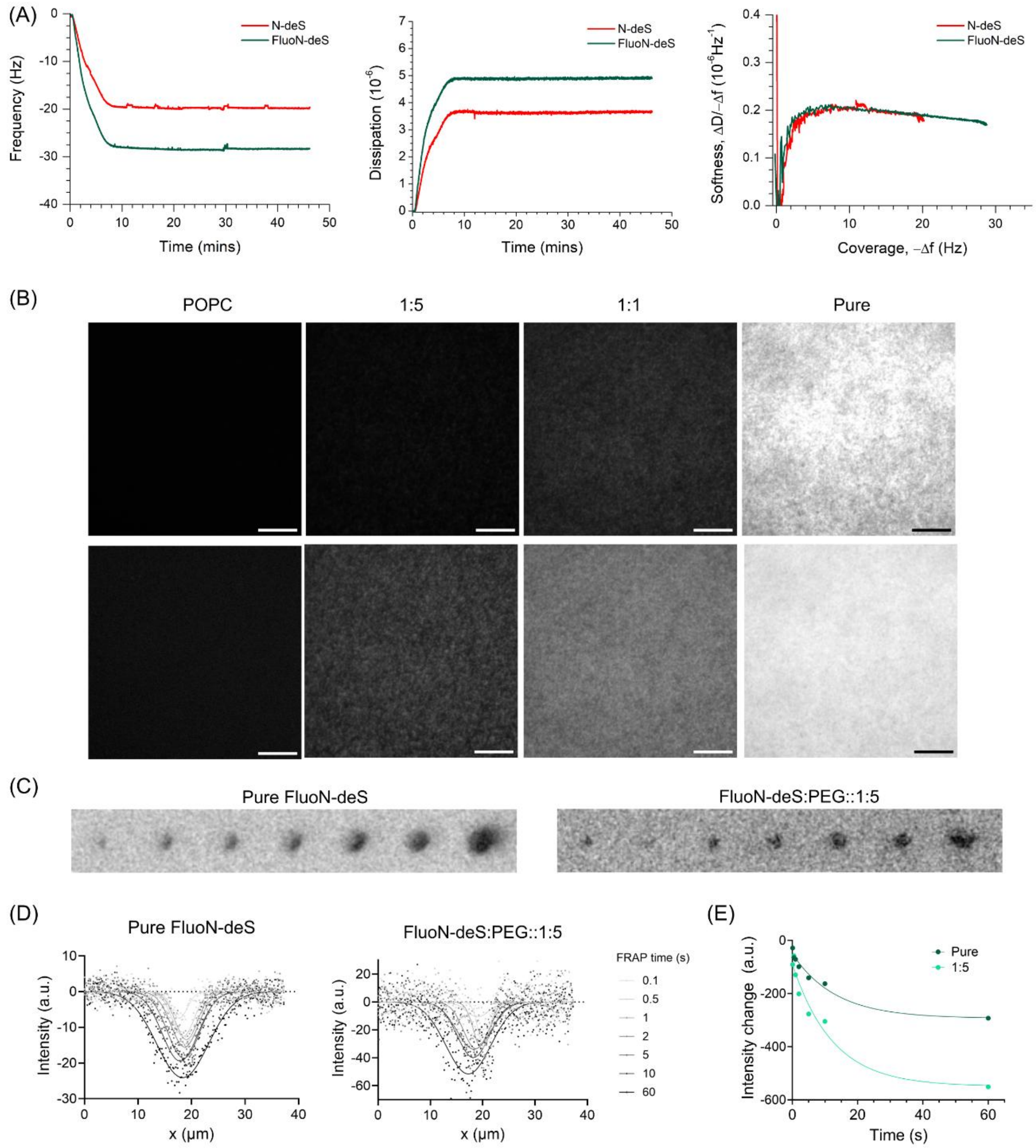

Figure S3. (A) QCM-D plots for labelled (dark yellow) and non-labelled (red) biotinylated N-deS that were immobilized on a streptavidin layer (20 µg/mL, 20 mins) on SLB containing 10% biotin lipids (100 µg/mL, 15 mins), indicating that labelling process did not disrupt bGAG adsorption. An increase in both the final frequency (left) and dissipation (middle) values for FluoN-deS is observed and ascribed to the additional mass of dye. The softness plot (right) displaying the overlap of both samples suggests that labelling does not affect the mechanical properties (softness) of the N-deS heparin chains. (B) Representative fluorescence image of four surfaces for the data presented in Fig. 1C. Top: Original image. Bottom: Same image as top with fluorescence intensity displayed using a logarithmic scale to enhance the difference between dim samples. Scale bar = 10 µm. (C) Bleaching experiments at increasing bleaching time on both pure fluorescent N-desulfated heparin

(FluoN-deS) and the 1:5 molar mixture with PEG (FluoN-deS:PEG::1:5). (D) Line profiles over the bleached regions shown in (C), after subtraction of the pre-bleach intensity (circles). Solid lines represent the best Gaussian fits to the profiles. (E) Maximum reduction of fluorescence intensity after bleaching, quantified as the area between the Gaussian fit and the baseline (y=0) in (D), as a function of bleaching time. Solid lines represent fits to a single exponential with a y-offset, consistent with photobleaching and showing no evidence of dequenching.

Approach 2: Immunoassay

Surface preparation for Immunoassay. Steps to prepare the surface until PFA incubation were identical to approach 1. To prepare heparin and the mixed heparin:PEG layer for this assay, the following concentration and exposure time were used: biotinylated parental hepain, bHeparin – 10µg/mL, 30 mins (Hep10); bHeparin – 1 µg/mL, 30 mins (Hep1); bHeparin and bPEGSH – molar ratio of 1:5, 30 mins (Hep:PEG::1:5); bPEGSH – 25 µg/mL, 30 mins (PEG). Following the rinsing with PBS, all surfaces were further rinsed with 0.2% BSA. The surfaces were then first incubated with a 5 µg/mL solution of antiHS antibody for 2 hr and then with anti-mouse HRP conjugate secondary antibody at final concentration of 0.4 µg/mL for 1 hr following an extensive rinsing with 0.2% BSA. After rinsing, surfaces were exposed to HRP substrate according to manufacturer's instructions and immediately (<1 min) imaged using Amersham Imager 680 (GE Healthcare) at exposure time of 0.5 s, Fig. S4A. Averages from three independent values with their standard deviation are summarized in Fig. S4B. This approach gives a factor of 19.7 ± 4.4 between the surface density of "Hep10" and "Hep:PEG::1:5".

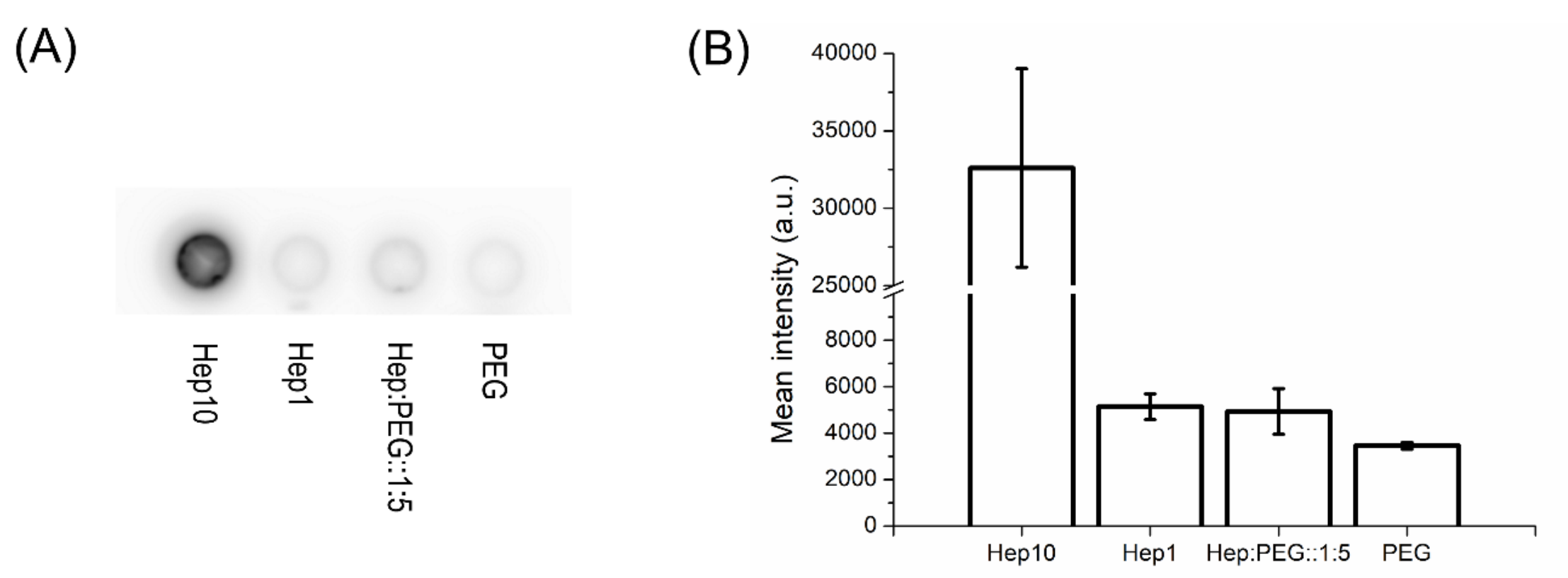

Figure S4. (A) Representative immunostaining assay reading of four surfaces prepared by exposing a streptavidin layer to heparin at final concentration of 10 µg/mL (Hep10) and 1

µg/mL (Hep1), with a mixture of bHeparin and bPEG at molar ratio of 1:5 (Hep:PEG::1:5), and PEG at 25 µg/mL for 30 mins. (B) Chemiluminescence signal from the immunostaining assay in (A). Mean and standard deviation were calculated from 3 separate wells per condition that were prepared on 2 independent days.

Estimation of spacing between GAG chains in mixed GAG:PEG layer. Using the SPR data for heparin or its derivatives films at equilibrium (Table 1) and the average intensity of N-deS films from the assay based on fluorescently labelled GAGs (Fig. S3) prepared at the same bulk concentration of N-deS as SPR (i.e., 10 µg/mL), we have estimated the distance between 2 heparin chains for 1:5 molar ratio of heparin:PEG to be 30 ± 4 nm (Table 1). To extract this distance, we divided the SPR resonance shift of each GAG reported in Table S3 with the reduction factor obtained from our fluorescent labelled GAG assay; this factor or the ratio of the fluorescent signal of FluoN-deS and FluoNdeS:PEG::1:5 (or 1:5) surfaces was 24.2 ± 2.6. Then the mass density and the grafting coverage were calculated as described in the main text. It should be noted that we selected data from approach 1 (i.e., fluorescent labelled GAG assay) to estimate the GAG grafting coverage in the main text, due to the lower uncertainty in the average value.

7. Additional Equilibrium Fluctuation Analysis data

Fig. S5 summarizes the equilibrium surface coverage of HPV16 on the different heparin derivatives.

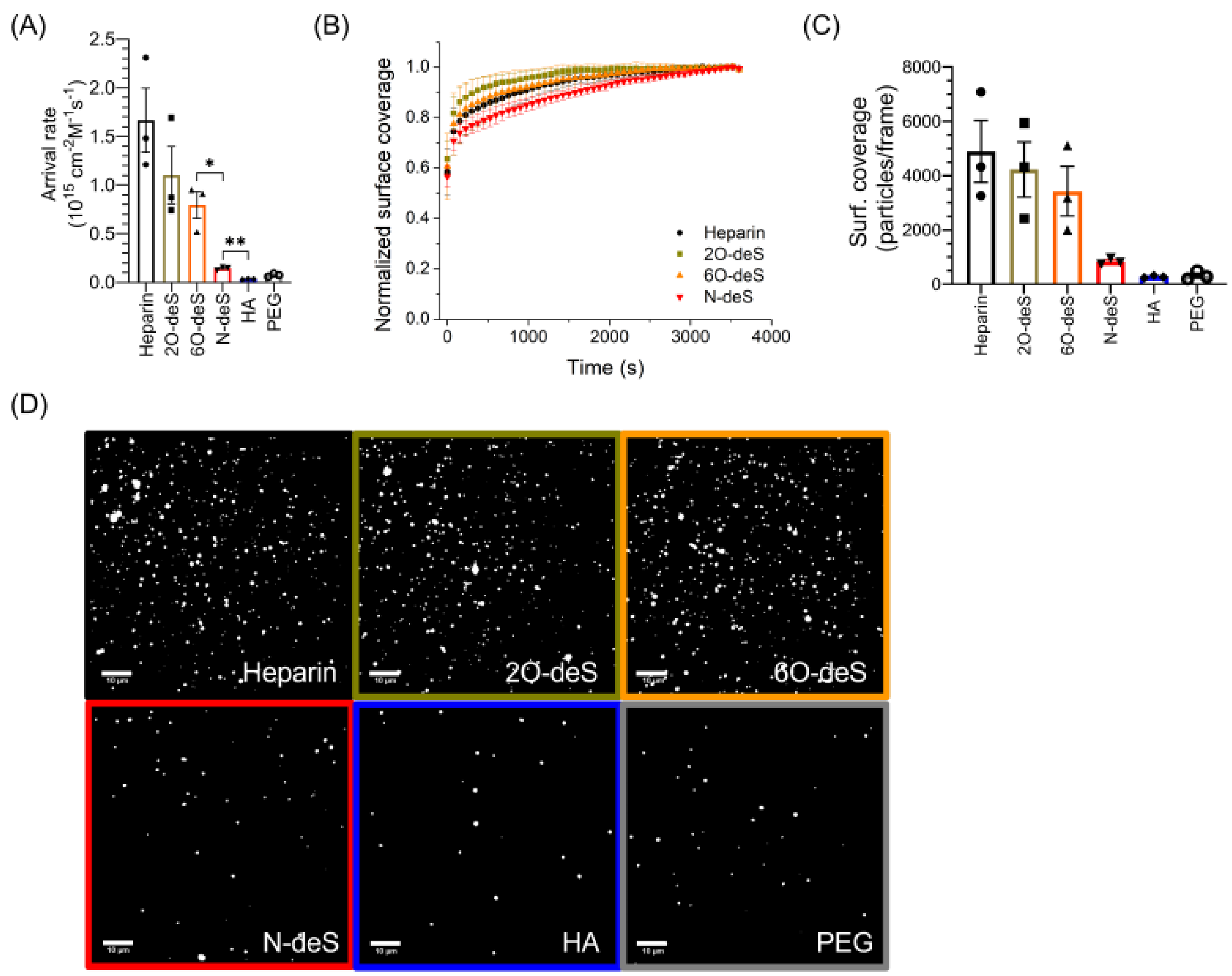

Figure S5. (A) Normalized arrival rate obtained from the linear fit of the data in Figure 2B for the indicated conditions. (B) Normalized surface coverage as a function of time. It is to be noted that the sharp increase observed in the first frames is an artifact associated with issues in recognizing all particles present in the initial frame. Accordingly, the first 150 s of the experiments are not considered in our analyses. Each data point represents average with error bars (i.e., standard deviation) from three independent experiments for four GAGs as indicated (C) Each point in the plot represents average surface coverage from five individual images with the size of 129×129 μm^2 of a single image and bar plots are average values ± S.D. from three independent experiments for tested conditions. (D) Representative images from the first frame of the movies, which were used for kinetics data analysis presented in Figure 2. A Gaussian Blur (=1 pixel) and all images were set to the same intensity threshold. Scale bar = 10 μm.

8. Diffusion-controlled particle flux calculation

To estimate the overall collision rate between the particles, present in well-mixed solution at uniform concentration of C_b with diffusion coefficient of D, and the surface, we can use the following expression derived from Fick's first and second laws:

$$J = C_b \sqrt{\frac{D}{\pi \Delta t}} \qquad \text{(Eq. S2)}$$

where Δt represent the observation time of the particle hitting and bouncing off the surface and is the time between each frame in videos recording for our kinetics experiments. Note that Eq. S2 is valid for the surface that is a reflective, implying that all the particles that will reach the surface bounce back and there is no adsorption. For full derivation of Eq. S2, see chapter 2 of reference (9). For HPV16 particles, using $D = 8.73 \times 10^{-8}\ \mathrm{cm^2 s^{-1}}$, $\mathrm{C_b} = 1.14\ \mathrm{pM}$, and $\Delta t = 15\mathrm{s}$, we obtain $J = 3.0 \times 10^{4}\ \mathrm{collisions.cm^{-2}.s^{-1}}$. This corresponds to an arrival rate of $r_J = 2.6 \times 10^{16}\ \mathrm{collisions.cm^{-2}.s^{-1}.M^{-1}}$, which is ~1 order of magnitude higher than the experimental values for heparin (Table 2).

9. Summary of the AFM-based SMFS data

The table below (Table S4) summarizes the AFM-based SMFS data obtained in reference (3).

Table S4*: Summary of association constant, interaction time, and associate rate for the interactions between HPV16 and four heparin surfaces obtained from fitting of dynamic force spectra data with Bell-Evans model and binding probability analysis.

Heparin surfaces	k_{on}^{SB} ($10^6\ \mathrm{M^{-1}s^{-1}}$)	τ^{SB} (s)	r_{on} ($\mathrm{s^{-1}}$)	$k_{\mathrm{off}}^{SB} = r_{\mathrm{off}}$ ($\mathrm{s^{-1}}$)
Heparin	2.0 ± 0.6	0.087 ± 0.027	3.7 ± 1.2	0.004 ± 0.003
2-O-deS	3.2 ± 0.8	0.055 ± 0.013	5.9 ± 2.5	0.015 ± 0.004
6-O-deS	4.6 ± 1.4	0.038 ± 0.011	8.5 ± 3.9	0.12 ± 0.04
N-deS	1.4 ± 0.3	0.124 ± 0.022	2.6 ± 1.2	12.2 ± 5.2

*Modified table from reference (3).

10. Markov-Model for multivalent binding

It is possible to use a continuous-time Markov chain to model the multivalent binding of a virion to the heparin surface, considering independent bonds and the possibility of rebinding if one or more bonds to the surface are present.

The probability of transition between states in the Markov model, i.e., changing the number of bonds between the viral particle and the heparin substrate, can be expressed in a matrix Q, in which each row represents the transition from a particular state to each other possible state. The off-diagonal element represents the rate of the transition between states, and the diagonal values are the negation of the total rate at which the state transitions to any other state. Considering that the number of bonds can only be increased or reduced by one, we can write the matrices describing the interaction varying the maximum number of bonds that the HPV16 particle can create with the surface. We limit ourselves to the cases between 1 and 5 bonds at the heparin grafting density considered in the study the probability for a particle to form more than 5 bonds is very small.

- For 2 bonds: $Q = \begin{bmatrix} 0 & 0 & 0 \\ r_{\text{off}} & -r_{\text{off}} - r_{\text{on}} & r_{\text{on}} \\ 0 & 2r_{\text{off}} & -2r_{\text{off}} \end{bmatrix}$;
- For 3 bonds: $Q = \begin{bmatrix} 0 & 0 & 0 & 0 \\ r_{\text{off}} & -r_{\text{off}} - 2r_{\text{on}} & 2r_{\text{on}} & 0 \\ 0 & 2r_{\text{off}} & -2r_{\text{off}} - r_{\text{on}} & r_{\text{on}} \\ 0 & 0 & 3r_{\text{off}} & -3r_{\text{off}} \end{bmatrix}$;
- For 4 bonds: $Q = \begin{bmatrix} 0 & 0 & 0 & 0 & 0 \\ r_{\text{off}} & -r_{\text{off}} - 3r_{\text{on}} & 3r_{\text{on}} & 0 & 0 \\ 0 & 2r_{\text{off}} & -2r_{\text{off}} - 2r_{\text{on}} & 2r_{\text{on}} & 0 \\ 0 & 0 & 3r_{\text{off}} & -3r_{\text{off}} - r_{\text{on}} & r_{\text{on}} \\ 0 & 0 & 0 & 4r_{\text{off}} & -4r_{\text{off}} \end{bmatrix}$;
- For 5 bonds:

$$Q = \begin{bmatrix} 0 & 0 & 0 & 0 & 0 & 0 \\ r_{\text{off}} & -r_{\text{off}} - 4r_{\text{on}} & 4r_{\text{on}} & 0 & 0 & 0 \\ 0 & 2r_{\text{off}} & -2r_{\text{off}} - 3r_{\text{on}} & 3r_{\text{on}} & 0 & 0 \\ 0 & 0 & 3r_{\text{off}} & -3r_{\text{off}} - 2r_{\text{on}} & 2r_{\text{on}} & 0 \\ 0 & 0 & 0 & 4r_{\text{off}} & -4r_{\text{off}} - r_{\text{on}} & r_{\text{on}} \\ 0 & 0 & 0 & 0 & 5r_{\text{off}} & -5r_{\text{off}} \end{bmatrix}.$$

Using the Markov theory, it is possible to calculate the average time to reach any j from a starting state i (t_{ij}), using the recursive formula (10):

$$t_{ij} = \begin{cases} 0 & \text{if } i = j \\ -\dfrac{1}{q_{ii}} + \displaystyle\sum_{k \neq j} -\dfrac{q_{ik}}{q_{ii}} t_{kj} & \text{if } i \neq j, \end{cases} \qquad \text{(Eq. S3)}$$

Where q_{xy} are the matrix element in row x and column y. In this formula, the time needed to transition from the state i to j is the sum of:

- The average time spent by the system in state *i* before transitioning to any other state: $\frac{1}{-q_{ii}}$.
- The time that it takes the system to reach state *j* from any other state the system can transition to (t_{kj}) weighted by the probability to make that transition ($\frac{q_{ik}}{-q_{ii}}$). This is the case because the transition matrix is constructed so that the sum of each row equals 0, i.e. $q_{ii} + \sum_{j \neq i} q_{ij} = 0$.

In our experiments, we only consider particles that bind the surface during the observation time and assume that at arrival only one bond is present. We thus limit our calculations to the average time to reach release (0 bonds) from a single bond, i.e., t_{10} and obtain equation 4 in the main text. This time depends on the maximum number of bonds possible (see section 11 "Probability of establishing multiple bonds with end-grafted heparin"), which in turn depends on the heparin concentration and possibly its conformation which may or may not allow engagement with the viral capsomers.

Solving the previous equation, we obtain:

- For 2 bonds: $t_1^{(2)} = {r_{\text{on}} + 2r_{\text{off}}}/{2r_{\text{off}}^2}$
- For 3 bonds: $t_1^{(3)} = {3r_{\text{off}}^2 + 3r_{\text{off}}r_{\text{on}} + r_{\text{on}}^2}/{3r_{\text{off}}^3}$
- For 4 bonds: $t_1^{(4)} = {4r_{\text{off}}^3 + 6r_{\text{off}}^2 r_{\text{on}} + 4r_{\text{off}}r_{\text{on}}^2 + r_{\text{on}}^3}/{4k_{\text{off}}^4}$
- For 5 bonds: $t_1^{(5)} = {5r_{\text{off}}^4 + 10r_{\text{off}}^3 r_{\text{on}} + 10r_{\text{off}}^2 r_{\text{on}}^2 + 5r_{\text{off}}r_{\text{on}}^3 + r_{\text{on}}^4}/{5r_{\text{off}}^5}$

Note that in the trivial case of only a single bond allowed, the transition matrix is:

$$\begin{bmatrix} 0 & 0 \\ r_{\text{off}} & -r_{\text{off}} \end{bmatrix}$$

Thus $t_1 = \frac{1}{r_{\text{off}}}$ or, as expected, the mean release time is reciprocal of the dissociation rate.

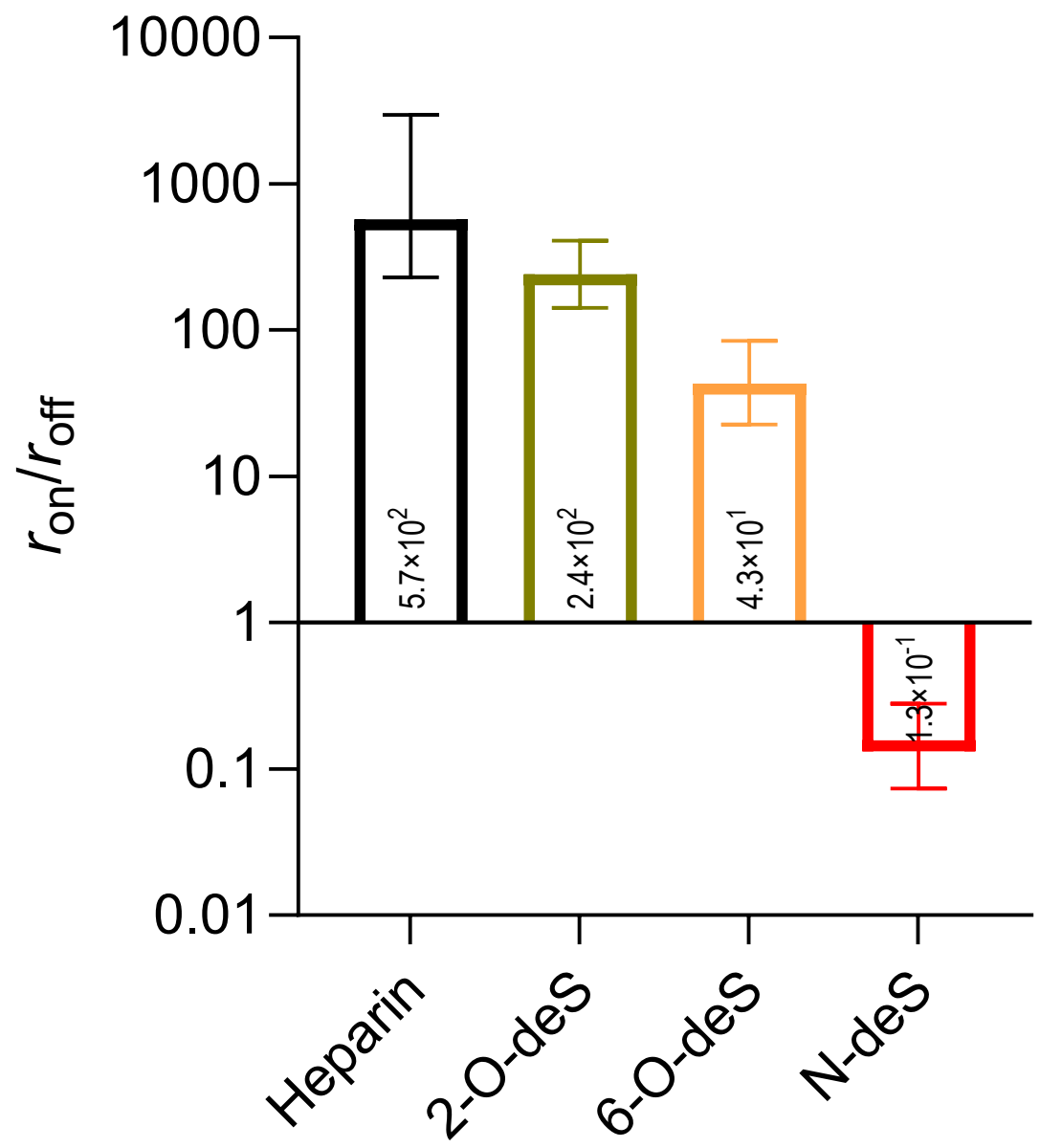

Figure S6. Ratio between the association and dissociation rates used in the Markov model for multivalent binding and derived from the AFM-SMFS reported in reference 1 and Table S4. The error bars indicate the maximum and minimum values obtained from the uncertainty interval of the AFM-SMFS data.

11. Probability of establishing multiple bonds with end-grafted heparin

The height of both the PEG and heparin layers depends on the polymer conformation and characteristics. We first consider PEG, as it is the most abundant molecule on the surface and as it acts as a spacer between the HPV16 particle and the surface.

The conformation assumed by a grafted polymer depends on the grafting coverage. If very sparse, i.e., if the polymer Flory radius (R_F) is much smaller than the separation between chain (d), it assumes a mushroom conformation. Otherwise, if $R_F >> d$, neighboring chains interact and extend away from the surface forming a brush.

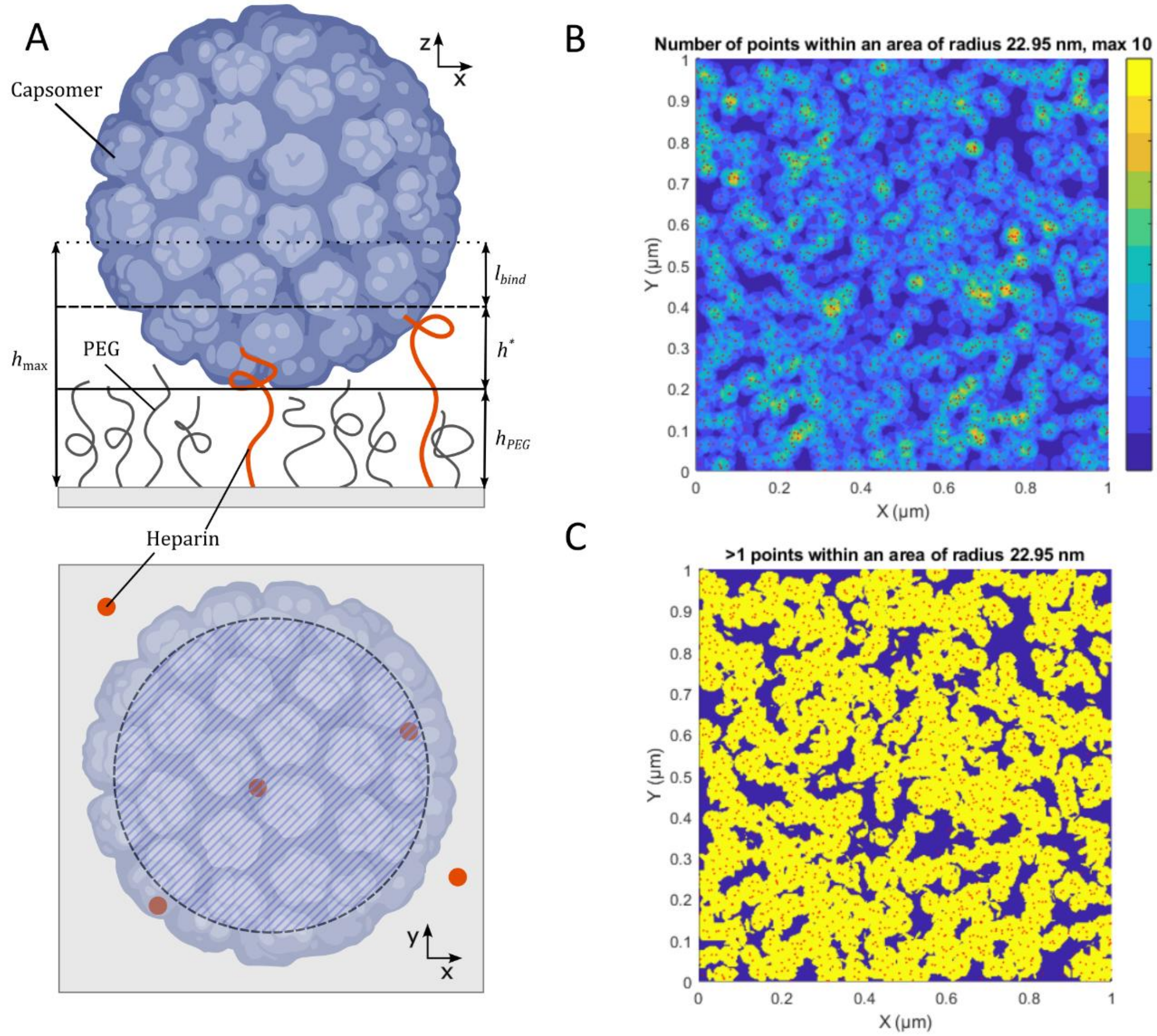

Figure S7. (A) Side (top) and top-down (bottom) representation of an HPV16 particle (in blue) bound to end-grafted heparin (in orange). The PEG brush prevents the virion to reach the surface (solid black line, h_{PEG}). The heparin chains can stretch up to h_{max} (dotted black line). However, binding requires at least 4 disaccharides, reducing the maximum binding height by l_{bind} (dashed black line). The shaded blue area indicates the area in which binding is possible, while the blue circles show the size of a virion. (B) Simulated distribution of heparin chains (red dots) superimposed to a heat map showing the maximum number of bonds a HPV16 particle can form. Simulation parameters: grafting coverage: $\sigma=1.7\times10^{11}$ cm^{-2}; virion contact area: 1655 nm^2, representative of N-deS. (C) Map indicating the portion of surface that can sustain bonds between HPV16 and 2 or more heparin chains (in yellow).

In our case, PEG is grafted onto a streptavidin layer bound to a biotinylated supported lipid bilayer. In these conditions, streptavidin arranges in lattices with footprints between 25 and 34 nm^2 (11). From the SPR experiment, we obtained this footprint to be 24.05 ± 2.25 nm^2

upon exposing SLBs containing 10% biotin to 50 µg/mL bulk concentration of streptavidin; this corresponds to a dense monolayer. At full coverage, each streptavidin has two biotinylated PEG molecules, and the grafting coverage is $\sigma_{PEG} = 0.07\ nm^{-2}$ or an interchain distance of ~4 nm. This is comparable with the Flory radius of 3.4k PEG used in our experiments ($\mathrm{R_F^{PEG}} = 4.7$ nm, as calculated below). The PEG is thus in a transition configuration between mushroom and brush. For simplicity, we consider the two extreme cases of mushroom and brush configuration.

For an isolated polymer, we consider the Flory theory for real polymer chain, i.e., we consider steric hindrance between monomers. The polymer conformation is determined by the balance between the interaction between single monomers that does not allow the chain to collapse on itself and the tendency of the polymer to assume a coiled low entropy configuration, which opposes polymer stretching. This can be expressed as:

$$E_{\text{chain}} = k_B T\left(v\frac{N^2}{R^3} + \frac{R^2}{Nb^2}\right) \qquad \text{(Eq. S6)}$$

where $v = b^2 d$ is the exclusion volume of a single polymer segment, b and d are the segment Kuhn length and diameter, respectively, $N = L_c/\mathrm{b}$ is the number of segments composing the polymer chain of total length L_c and R is the end-to-end distance of the polymer chain, which we take as the measure of the polymer size. In the case of PEG, this results in the free energy landscape shown in blue in Fig. S8, and an equilibrium R, which is the polymer Flory radius of 4.7 nm.

In the case of a brush configuration, the brush height is dependent on the grafting coverage, and the free energy landscape can be calculated using the Alexander-de Gennes model:

$$E_{\text{brush}} = k_B T\left(vN\frac{N\sigma}{R} + \frac{3R^2}{2Nb^2}\right) \qquad \text{(Eq. S7)}$$

where we still consider the equilibrium between interaction and entropic contribution but adapt the first term to consider intra-chain interactions. The result of this model in the case of PEG 3.4k is shown in green in Fig. S8A. We can observe that due to the low grafting coverage, the two models yield similar results indicating limited interaction between chains and thus we will consider the mushroom configuration for the rest of our calculation and consider R as the height of the PEG layer.

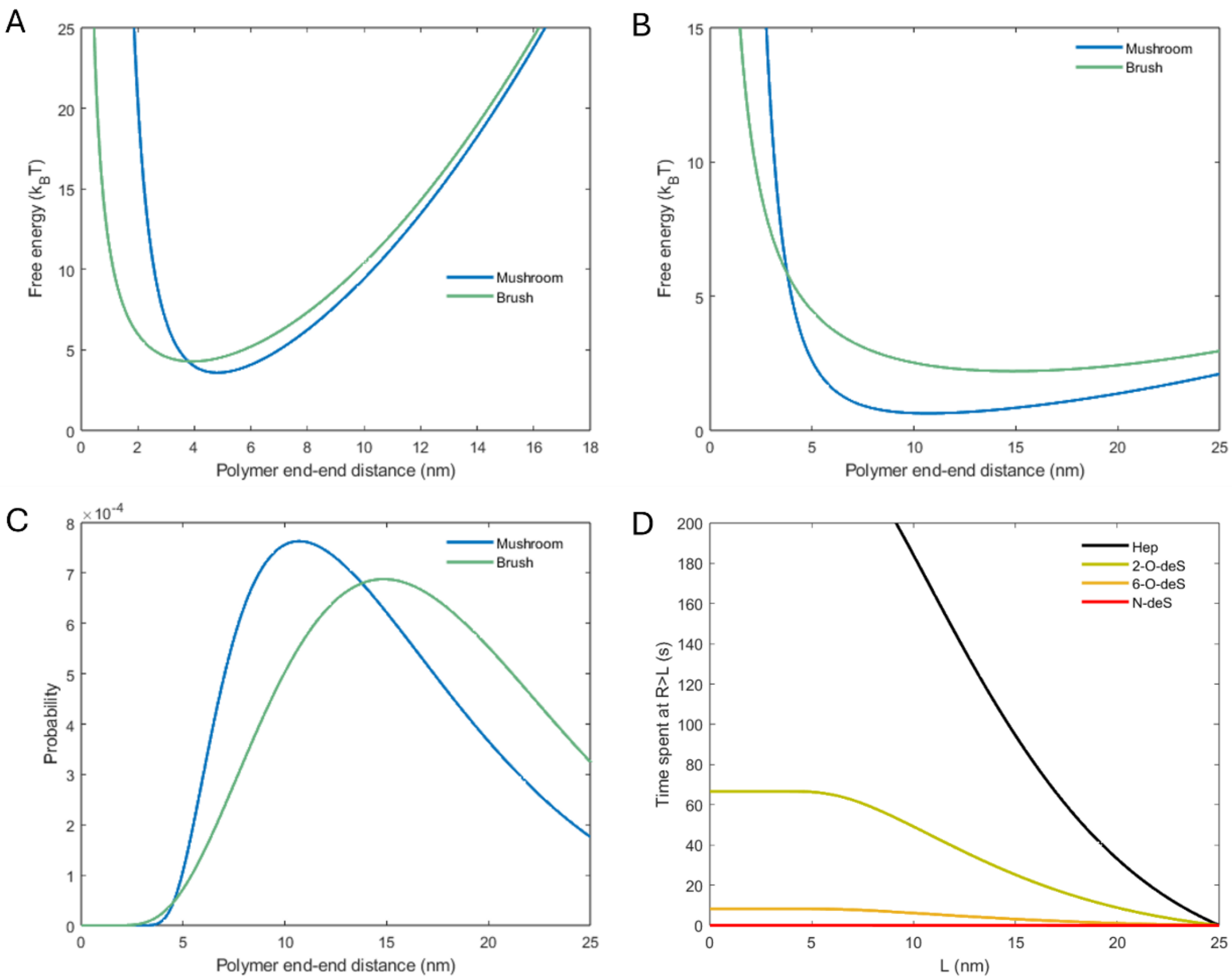

Figure S8. (A-B) Free energy landscape along the elongation path of a heparin chain in the case of a mushroom (blue) or brush (green) configuration for PEG 3.4k (A) and heparin (B). (C) Probability of finding a heparin chain at a given elongation calculated using Boltzmann statistics from B. Mushroom configuration in blue and brush in green. (D) Time spent on average at elongations larger than L_{ext} before the virion detaches from the surface, i.e. the integral of the elongation probability from L_{ext} to the contour length multiplied by $1/k_{off}$.

We can perform the same analysis for heparin (b = 9.0 nm (1,12), d = 0.5 nm, L_c = 25 nm). The results are shown in Fig. S8B. As for PEG, the two configurations result in very similar energy landscape and thus we consider the mushroom configuration in this case as well. Compared to PEG, the high charge carried by heparin results in a stiffer chain with equilibrium distance of 8 nm, and a much lower energy barrier towards extension.

Since a heparin chain will dynamically extend and contract due to thermal fluctuation, the chain will reach distances much larger than the equilibrium position. We thus consider as maximum extensions the length that the heparin chain assumes for a period comparable to $t_{\text{on}} = 1/r_{\text{on}}$, i.e., the time needed to establish a bond, over the time that the virion is present on the surface, which is $1/r_{\text{off}}$.

The probability of a chain to be found at a given elongation or larger can be calculated from the free energy landscape using Boltzmann's statistics:

$$P(L > h) \approx \int_h^{L_c} Ce^{-\frac{\Delta E(x)}{k_B T}} dx, \quad \text{(Eq. S8)}$$

where $C = 1/\int_0^{L_c} \exp\left(\frac{\Delta E(x)}{k_B T}\right) dx$ is a normalisation constant so that $P(L > 0) = 1$.

We can calculate the time spent at a given elongation or larger as the probability of the particle to be in that state multiplied by $1/k_{\text{off}}$, $\tau(L > h) = \frac{P(L>h)}{k_{\text{off}}}$. While this is not a measure of the continuous time spent in this configuration, we interpret it as the polymer fluctuating around this conformation and eventually resulting in the creation of a bond. Numerically inverting this formula results in the maximum length of a heparin chain, h_{MAX}.

In addition, it has been shown that HPV16 binding to HS requires at least 4 disaccharides monomers (13,14). In first approximation, we can incorporate this minimum binding motif in our model by reducing the maximum elongation of the heparin chain by the length of 4 monomers, 4 nm.

Thus, the maximum height at which the heparin can bind the particle on top of the PEG layer, h^*, is thus equal to the maximum heparin length (h_{MAX}) minus the height of the PEG layer and the minimum binding motif. The results depend on the kinetic parameters of the HPV16-heparin interaction and thus vary between heparin sulfation (Table S5).

Table S5: Dissociation constants determined by AFM-SMFS (3), maximum elongation, interaction area, GAG grafting coverage and probability for a virion to form less than 2 bonds with the surface, i.e., to land on an area in which less than two heparin chains are present. The values between square brackets indicate the maximum and minimum probability calculated according to the error in the determination of the grafting coverage.

	$\boldsymbol{k_{\text{off},1}}$ (s^{-1})	$\boldsymbol{h^*}$ (nm)	$\boldsymbol{A}$ (nm^2)	$\boldsymbol{\sigma_{\text{hep}}}$ (10^{-3} nm^{-2})	$\boldsymbol{P(n \leq 1)}$ (%)
**Heparin**	0.004 ± 0.003	16.3	1726	1.1 ± 0.3	43.4 [34.5, 59.9]
**2-O-deS**	0.015 ± 0.004	16.3	1726	1.3 ± 0.3	34.1 [23.8, 48.5]
**6-O-deS**	0.12 ± 0.04	16.2	1726	1.5 ± 0.9	27.0 [8.2, 72.3]

**N-deS**	12.2 ± 5.2	15.1	1655	1.6 ± 0.4	25.8 [15.7, 41.0]
**Fully stretched N-deS**	-	16.3	1726	1.6 ± 0.4	23.8 [14.1, 38.7]
**Flory radius N-deS**	-	0.45	70.05	1.6 ± 0.4	99.4 [99.1, 99.7]

In the case of N-deS, $h^* = 15.1$ nm. This results in a contact area of 1.6×10^3 nm^2, and, considering a grafting coverage of 1.6×10^3 nm^{-2}, a probability of a particle to attach in a spot with less than two heparins, and thus not being able to form multivalent bonds is 25.8%.

Notably, in this theory, the energy penalty does not diverge when the end-to-end length of the polymer approaches its contour length, the maximum length obtainable if the chain is completely stretched. Without appropriate boundary conditions, this could lead to unphysical stretching beyond the contour length and likely underestimates the energy cost for the polymer to reach highly stretched configurations. As a result, we might be overestimating the interaction volume of a virus particle bound to the surface, and ultimately the r_{on}.

Finally, we check the validity of the assumption that heparin binding sites are always accessible for HV16 binding. We can estimate the rate at which the heparin chain explores possible configurations around the equilibrium one to be of the order of the polymer relaxation time. In the experimental conditions, we consider the heparin as a non-tangled chain in a good solvent, being a hydrophilic molecule in water, and thus employ the Zimm model(15) for polymer dynamics:

$$\tau_1 \approx \frac{1}{2\sqrt{3\pi}} \frac{\eta b^3 N^{3/2}}{k_B T} = 133 \text{ ns}, \qquad \text{(Eq. S9)}$$

where $\eta = 1$ mPa $\cdot$ s is the dynamic viscosity of water. This value is several orders of magnitude lower than both t_{on} and $1/k_{\mathrm{off}}$, indicating that heparin binding epitopes are indeed available at the timescales of the interaction with HPV16.

12. Comparison between the Markov-multivalency model and experimental results

Figure S9 shows the dissociation curves and rates obtained by equilibrium fluctuation analysis when targeting short-lived initial interactions. Particles were imaged using the

same setup as in Fig. 2, but at 1 fps for 15 minutes to observe bond lifetimes between 10 seconds and 5 minutes. This approach allowed improved resolution of the detachment of HPV16 particles bound by a single bond to 2-O-deS and 6-O-deS heparin. The resulting dissociation curves (Fig. S9A) were compared with those predicted by the Markov model, showing excellent qualitative agreement. The curves were fitted with a two-phase exponential decay with vertical offset ($y = A + Bexp(-k_{\mathrm{s}}t) + Cexp(-k_{\mathrm{f}}t)$, with $A + B + C = 1$). This fit was chosen to distinguish between monovalent interactions that are expected to have a fast dissociation rate in the range of seconds to minutes for 2-O- and 6-O-deS (k_{f}), and the more stable divalent bonds (k_{s}). The vertical offset considers higher multivalency interactions, which, according to the proposed model, have dissociation rates much longer than the experimental observation time and thus appear as irreversibly attached to the surface.

All samples, including HA and N-deS (data not shown), show a fast initial dissociation within a few seconds, possibly due to weak non-specific interactions. This component was excluded from the analysis by considering only detachment times longer than 10 s. The fast components for 2-O-deS and 6-O-deS show good quantitative agreement with the detachment rates measured for single bonds using AFM-SMFS experiments (Fig. S9B), in line with the idea that the fast dissociation component can be ascribed to the monovalent interaction. Heparin also shows a fast-detaching population; however, this fraction is smaller than that observed for the desulfated variants and is likely due to residual non-specific interactions. The detachment rate of the slow components is much longer than the measurement time and is therefore poorly constrained by the fitting. However, in the case of parental heparin it is compatible with the value expected for monovalent bonds.

Figure S10 shows the effect of increasing heparin grafting density on the predicted detachment curves according to the Markov model presented in this work. As expected, the increased binder concentration results in a higher proportion of multivalent bonds that do not rupture within the experimental time window, thereby increasing the fraction of irreversibly bound particles.

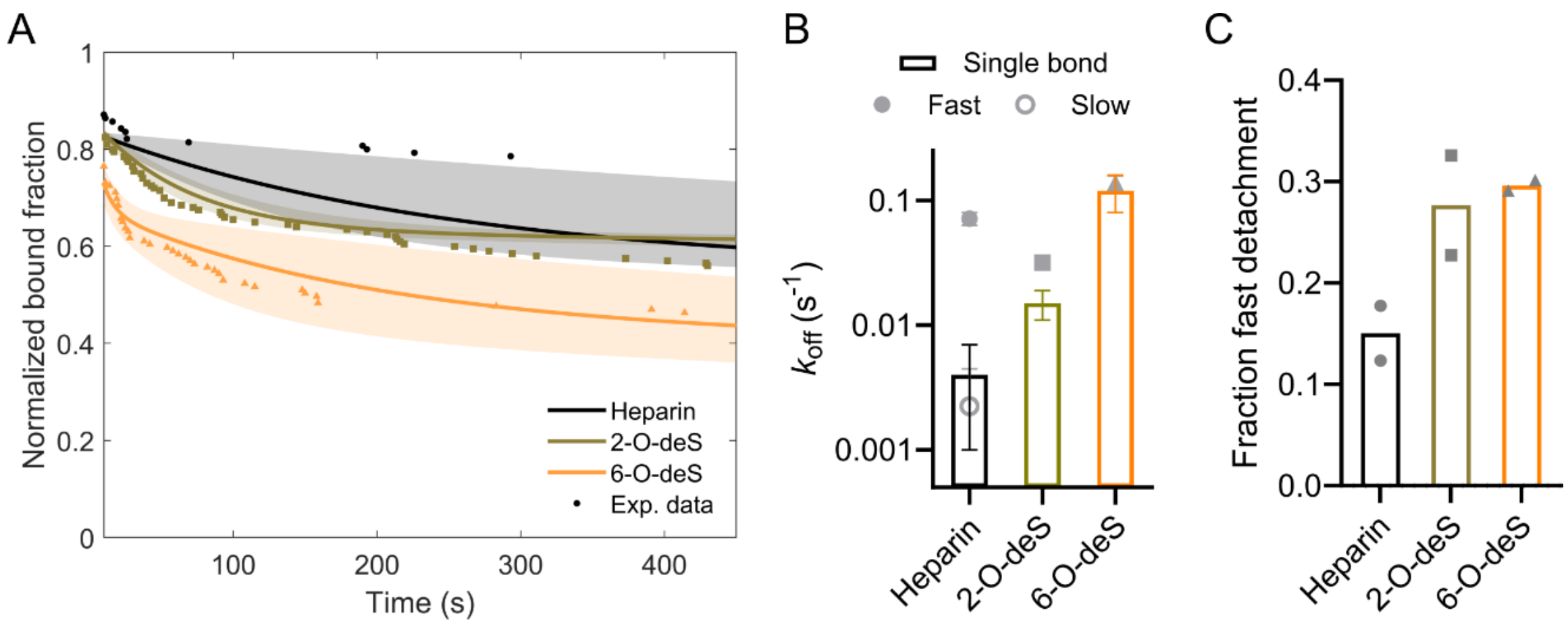

Figure S9. (A) Representative bond survival probability as a function of time obtained by equilibrium fluctuation analysis acquired at 1 fps for 15 minutes (symbols), compared to the Markov-multivalency model prediction in the same acquisition conditions (solid lines). The shaded area indicates the value obtained using the upper and lower limits of the bond lifetime as measured by AFM-SMFS (Table S4). (B) Mean dissociation rate constant of the short-lived particle population obtained by fitting the experimental data with a double exponential decay function plus offset (solid symbols), compared to the single bond dissociation rate measured by AFM-SMFS (Table S4). Slow dissociation rate constant for parental heparin is also included as a hollow circle. All obtained from 2 independent experiments and the error bars indicate the standard error of the mean. Error bars on the bar plot indicate the confidence interval of the AFM-SMFS measurements. (C) Fraction of particles that display the fast dissociation rate shown in B. All data were acquired from 2 independent experiments (grey symbols), the bar height indicated the mean.

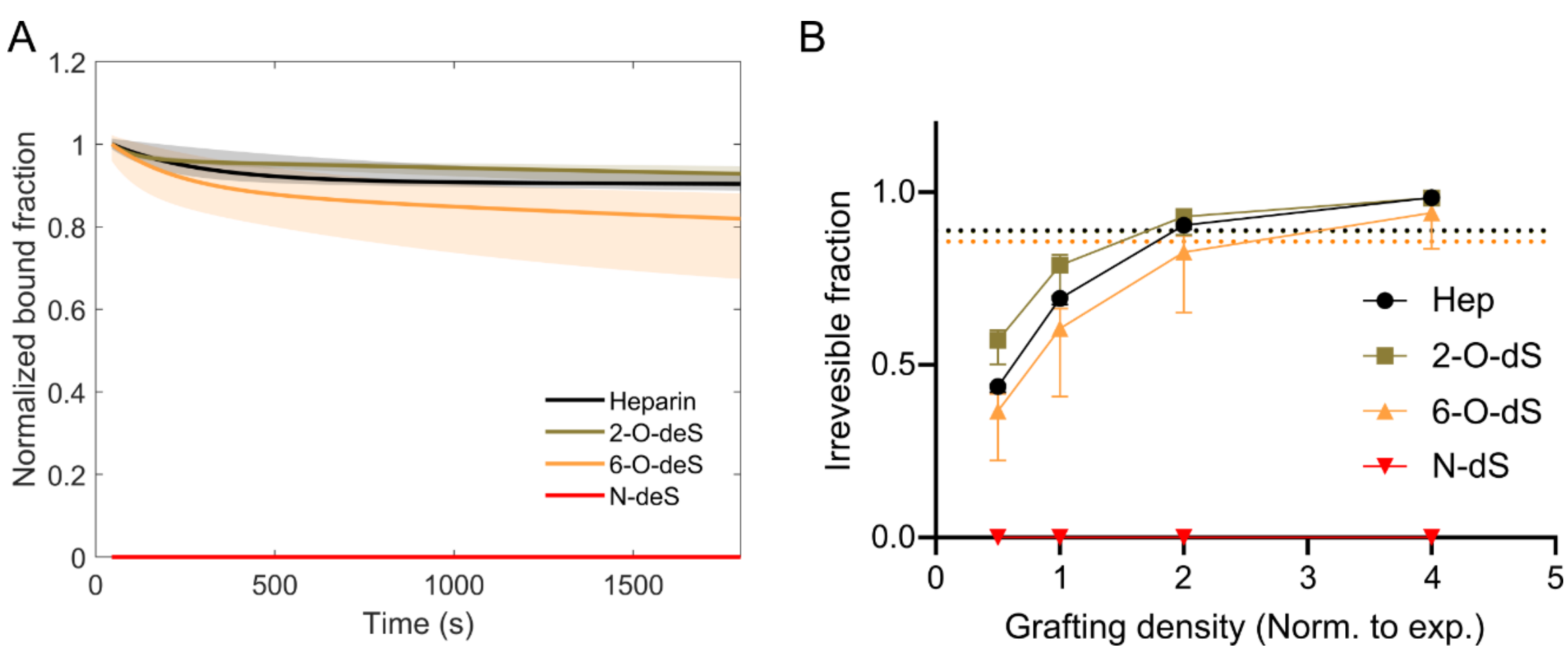

Figure S10. (A) Markov–multivalency model prediction of the bond survival probability as a function of time for a GAG grafting density twice that used experimentally (2.2-3.2×10^3 nm^{-2}). The solid line was obtained using the mean value of the bond lifetime, while shaded areas represent the upper and lower limits derived from AFM-SMFS measurements (Table S4). (B) Predicted irreversible fraction, defined as the fraction of the total number of bound particles that persist on the surface for times much longer than the experimental

observation period, as a function of GAG grafting density (normalized to the grafting density used experimentally). The dashed horizontal lines indicate the irreversible fraction measured experimentally using a single-exponential fit with offset. Error bars indicate the maximum and minimum values expected based on the confidence interval of the AFM-SMFS measurements (Table S4).

13. HPV16 diffusion on heparin film

Figures S9 and S10 illustrate the main results for tracking of HPV16 on parental heparin and N-deS heparin at two PEG:GAG densities. Fig. S13 provides the estimated diffusion coefficient for particles classified as confined, as a function of the signal-to-noise ratio, illustrating that no diffusion is observed in our tracking experiments.

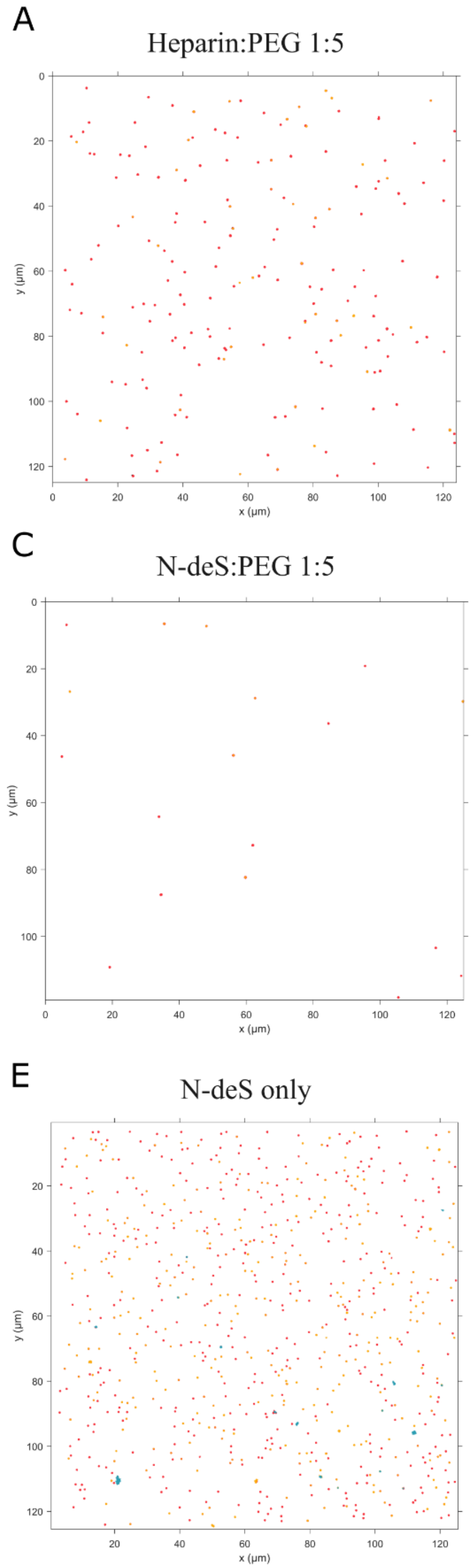

Figure S11. Representative tracks of HPV16 virus-like particles on heparin-grafted surfaces – (A) parental heparin and PEG in molar ratio 1:5, (B) parental heparin and PEG at a 1:1 molar ratio. (C) N-deS heparin and PEG in molar ratio 1:5, (D) N-deS heparin and

PEG at a 1:1 molar ratio. (E) Pure N-deS heparin. Tracks have been segmented according to the mobility characteristics. Red: immobile particle; yellow: Confined motion; blue: Free diffusion.

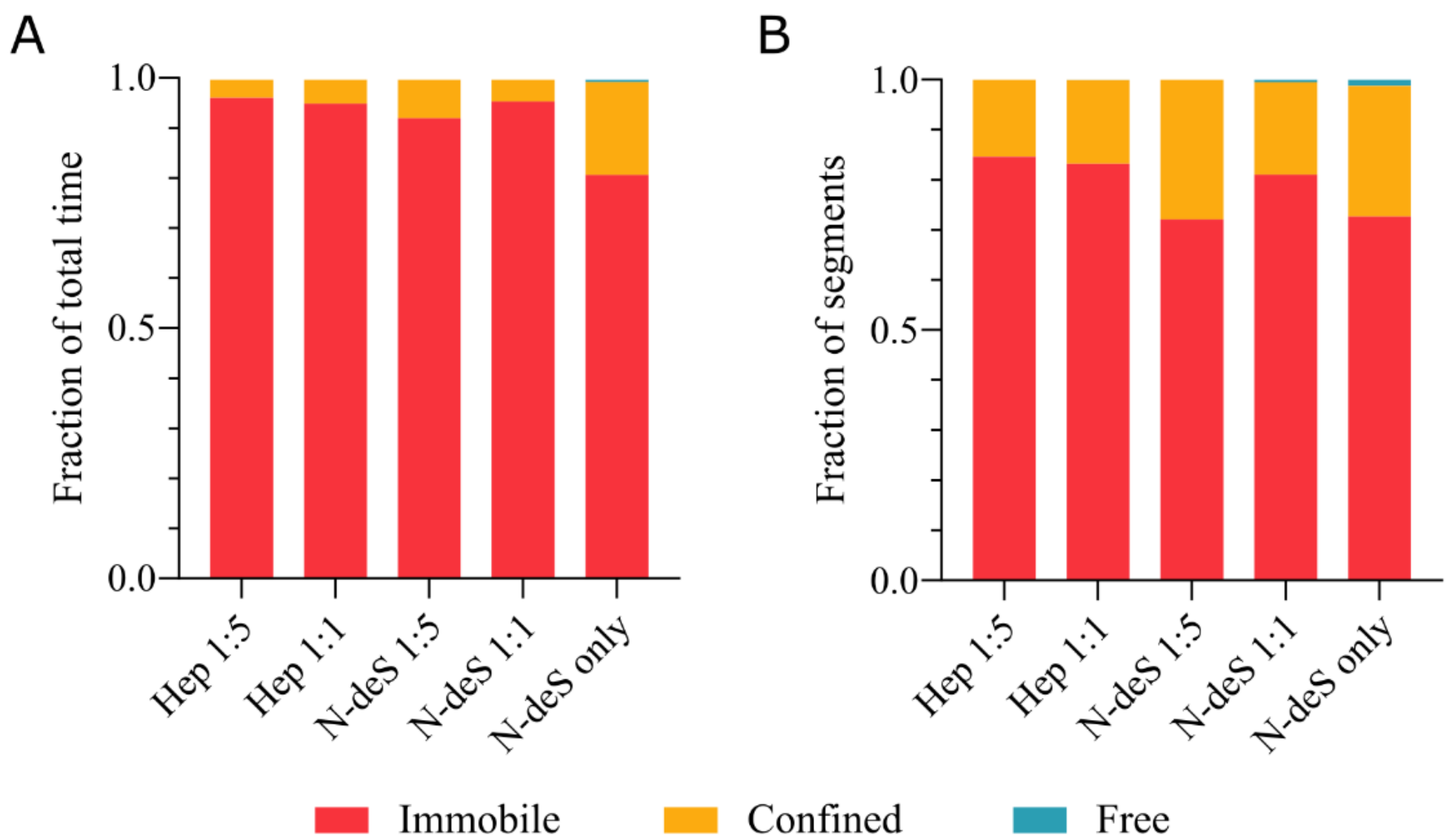

Figure S12. Fraction of the total cumulative time spent (A) or number of segments (B) in the three different motion regimes: immobile particles (red), confined motion (yellow), and free diffusion (blue).

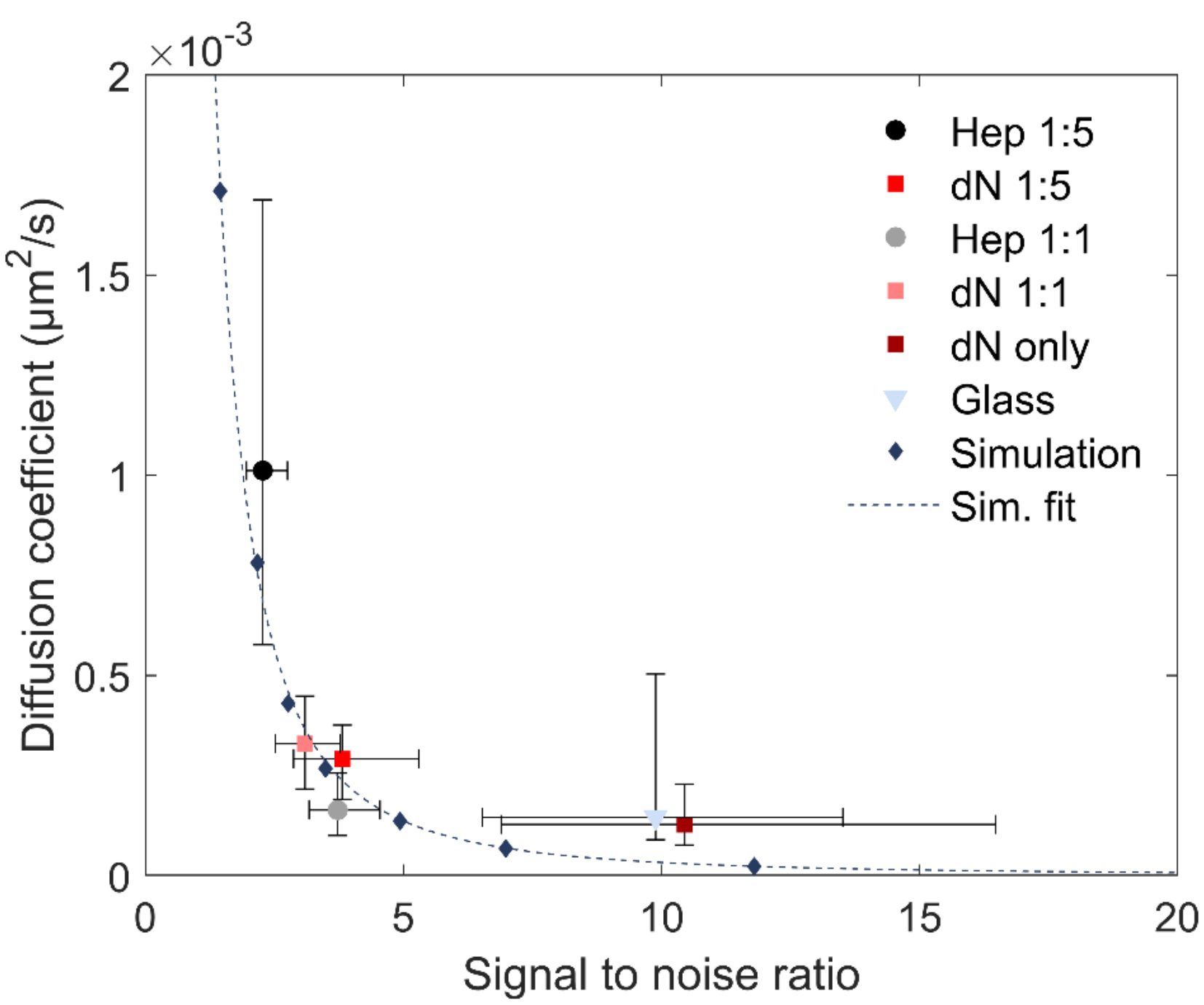

Figure S13. Diffusion coefficient (D) of HPV16 track segments classified as confined, as function of the signal to noise ratio of the images. The filled dots indicate the median value for each condition (Black: Hep:PEG::1:5; Grey: Hep:PEG::1:1; Red: N-deS:PEG::1:5; Pink: N-deS:PEG::1:1; Dark red: N-deS). Particles stuck on glass, in teal, were tracked as an immobile control. The error bars indicate the first and last quartile. The blue diamonds show the diffusion coefficient obtained using our algorithm for synthetic timelapses of immobile particles. The dashed line is the best power fit: $D = 0.0037 \cdot \mathrm{SNR}^{-2.05}$.

14. Simulation of TIRF images of immobile particles

We determined the localization precision limit of our tracking algorithm by generating synthetic timelapses of immobile particles with added acquisition noise. 150 particles were randomly positioned on a 130×130 μm^2 area and the Airy disk was approximated using a 2D Gaussians with standard deviation 220 nm, slightly larger than the of the resolution-limit: $\sigma_{\mathrm{spot}} = 0.45\lambda_{\mathrm{em}}/\mathrm{NA} = 181$ nm, where $\lambda_{\mathrm{em}} = 600$ nm is the emission wavelength and NA = 1.49 is the numerical aperture of the objective (16). The image was then discretized in 704x704 pixels to resemble the experimental images. The acquisition noise and background were matched to the experimental conditions by adding a uniform background of 145 and sum a normally distributed noise with standard deviation 8.3. The signal to noise ratio (SNR) was adjusted by changing the intensity of the spots to 20, 30, 40, 50, 70, 100, and 170. For each condition, 100 frames were generated maintaining the spots immobile and generating a new randomized noise pattern at each frame. The signal to noise ratio (SNR) was calculated as described in the Material and Methods in the main text for experimental time-lapses. Representative frames are shown in Fig. S14.

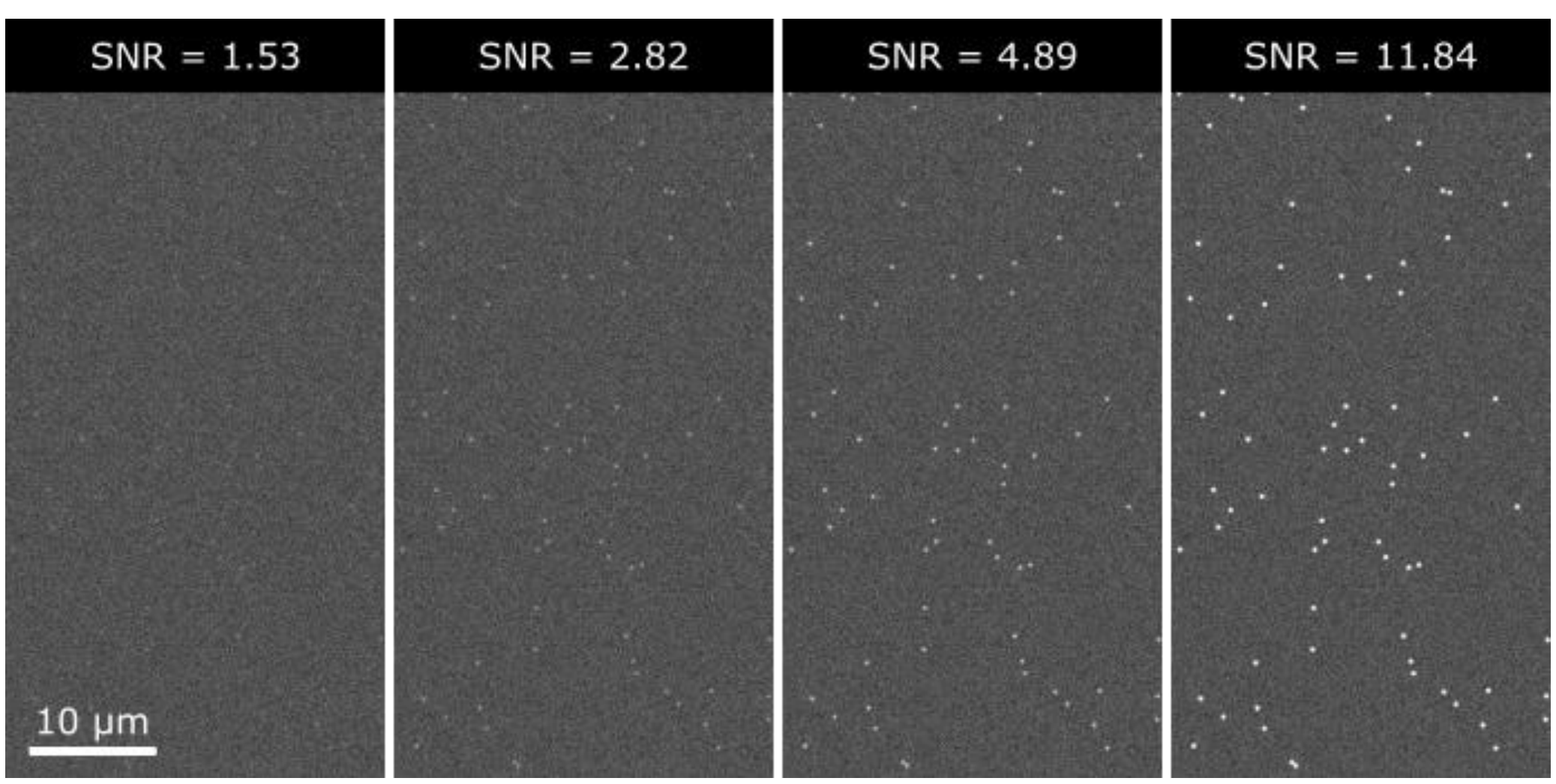

Figure S14. Example frames of the synthetic videos generated to test the localization precision of our particle tracking and analysis algorithm at signal to noise ratio (SNR) of 1.53, 2.82, 4.89, 11.84.

15. Model of multivalency aided diffusion

We modelled the diffusion of particles on surface-grafted binders originating from the establishment of transient multivalent bonds using in-house MATLAB scripts, as described in the Material and Methods in the main text (section Multivalency aided diffusion simulation).

The trajectories obtained from the Brownian dynamics simulations were analysed to compute several parameters:

- *Path length*: Computed as the sum of distances between consecutive particle positions, reflecting the cumulative distance travelled by the particle (Fig. S16A).
- *Hull area and confinement radius*: The area enclosed by the convex hull was calculated as a measure of the spatial extent of the particle's exploration, with an effective radius derived as half the square root of the area inscribed in the hull (Fig. S15A).
- *Maximum displacement*: Determined by calculating the largest distance between points on the convex hull enclosing all trajectory points, providing insight into the extent of particle movement within the simulation space (Fig. S16B).
- *Average number of bonds*: Evaluated by averaging the number of tether bonds formed between the particle and the surface across all recorded timesteps, indicating the extent of tether-particle interactions (Fig. S16C).
- *Track duration*: Calculated as the average time spent by the particle on the surface before detachment, breakage of the bond to all tethers (Fig. S16D).
- *Average particle speed*: The average speed of the particle between each iteration. The speed is calculated as the length of each particle step divided by dt (Fig. S16E).
- *Estimated diffusion coefficient*: Calculated as the hull area divided by the track duration (Fig. S15B). The true diffusion coefficient was also calculated by fitting of the mean square displacement. However, it produced highly noisy data due to the short length of tracks at low affinities and low binder concentration.

Qualitatively, both approaches resulted in similar diffusion coefficients (data not shown).

Additionally, each track was segmented into 4 possible motion types (immobile, confined, free diffusing and direct motion) as described for the analysis of the experimental tracks, and shown in Fig. S15A. The average motion type of the track was estimated as previously described (17). In brief, each motion type was scored by assigning a numerical value: 0: immobile, 1: confined, 2: free, 3: direct. The average motion type for each track was determined by calculating the average of the subtract scores weighted by their duration.
In the case of heparin, particles tethered to the surface can still move around the binding site due to the flexible nature of the heparin chain. In most cases however, the dynamic of the particle Brownian motion is much faster than the binding kinetics (μs to s) which makes the simultaneous simulation of both phenomena computationally challenging. To circumvent this problem, we performed a simulation of the Brownian motion of a particle tethered to the surface by a number of heparin chains between 1 and 100. The dynamics of a tethered spherical particle undergoing Brownian motion were simulated using an overdamped Brownian dynamics algorithm. The particle was modelled as a sphere of radius 25 nm initially suspended 10 nm above an impenetrable plane. The tether attachments were randomly distributed on both the lower hemisphere of the particle and the plane surface. The heparin chain was modelled as a polymer in mushroom configuration, as described in Eq. S6, with the addition of a vertical asymptote for stretching equal to the chain length to prevent unphysical overstretching of the chain: $F_{\mathrm{el}} = k_B T\left(-\frac{3\,N^2\nu}{\varepsilon^4}+\frac{3\,\varepsilon\,N}{b^2}+\frac{3\,N^2\nu}{(Nb-\varepsilon)^4}\right)$. Hydrodynamic interactions were included by considering translational and rotational drag, using drag coefficients derived from the Stokes relations: translational drag $\zeta_{trans} = 6\pi\eta R$ and rotational drag $\zeta_{trans} = 8\pi\eta R^3$, with viscosity $\eta =$ 1 mPa·s. Brownian motion was implemented by adding stochastic increments to particle displacement and rotation at each timestep as described in (18). The system evolved over discrete time steps of $dt = 1$ ns, for a total simulation time corresponding to 100,000 steps. Particle trajectories, tether attachment points, particle orientation, forces, and torques were recorded every 10 steps for analysis (Figs. S15 and S16A). The boundary condition was enforced by reflecting the particle off the plane surface, maintaining a minimum separation

corresponding to the particle radius plus the height of the PEG layer. The simulation was repeated 100 times for each condition, randomising the tether position. The distribution of the distance of the particle from the equilibrium position is shown in Fig. S18B. We then calculated the average distance from the equilibrium position as a function of the number of tethers and fitted it to the power law equation: $r_{\mathrm{avg}}(\mathrm{nm}) = 11.52 \times n_{\mathrm{teth}}^{-0.32}$, Fig. S18C. We incorporated the particle Brownian motion in the multivalent diffusion model by increasing the interaction area radius by the average distance from equilibrium according to the number of bounds between the particle and the surface at each simulation step. Summary of the results are presented in the heat maps in Fig. S19.

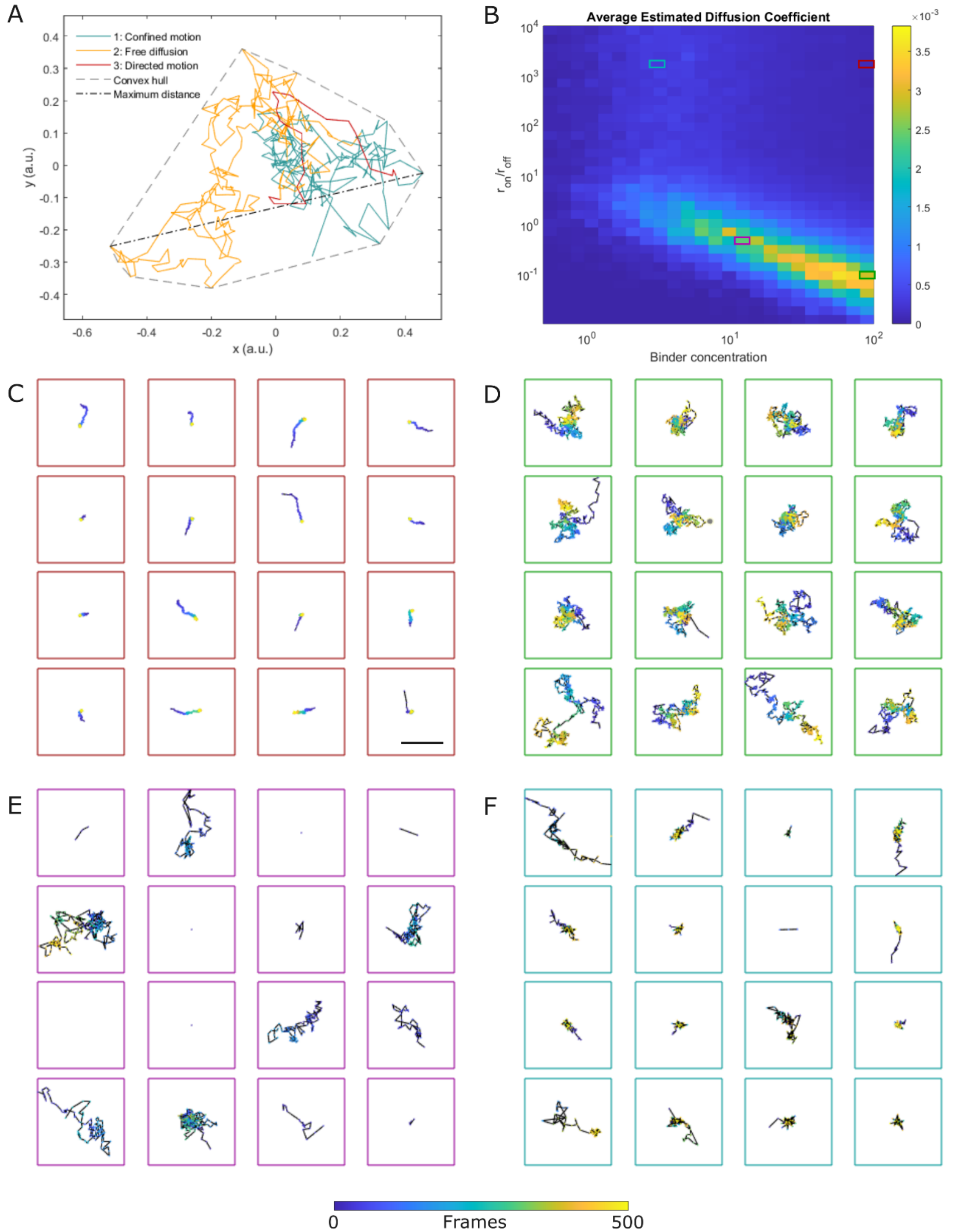

Figure S15. (A) Example of track segmentation showing sections identified as confined diffusion (teal), free diffusion (yellow) and directed motion (red). The track shown has an average motion score of 1.604, indicating mostly free diffusion. The convex hull containing all the track points and used to calculate the area explored by the track is shown as the gray dashed line and the maximum distance between points in the track as black dash dotted

line. (B) Heat map showing the estimated diffusion coefficient for the conditions described in the main text ($r_{\mathrm{on}}/r_{\mathrm{off}}$ = from 0.01 to 104 and binder within the interaction area, n_{bind}, between 0.5 and 100). (C-F) Example tracks using the conditions highlighted by the squares in B for $r_{\mathrm{on}}/r_{\mathrm{off}}$ = 1311, n_{bind}=100 (C); $r_{\mathrm{on}}/r_{\mathrm{off}}$ = 0.115, n_{bind}=100 (D); $r_{\mathrm{on}}/r_{\mathrm{off}}$ = 2.96, n_{bind}=11.0 (D); and $r_{\mathrm{on}}/r_{\mathrm{off}}$ = 1311, n_{bind}=3.65 (F). The scale bar indicates 1 normalized unit of space.

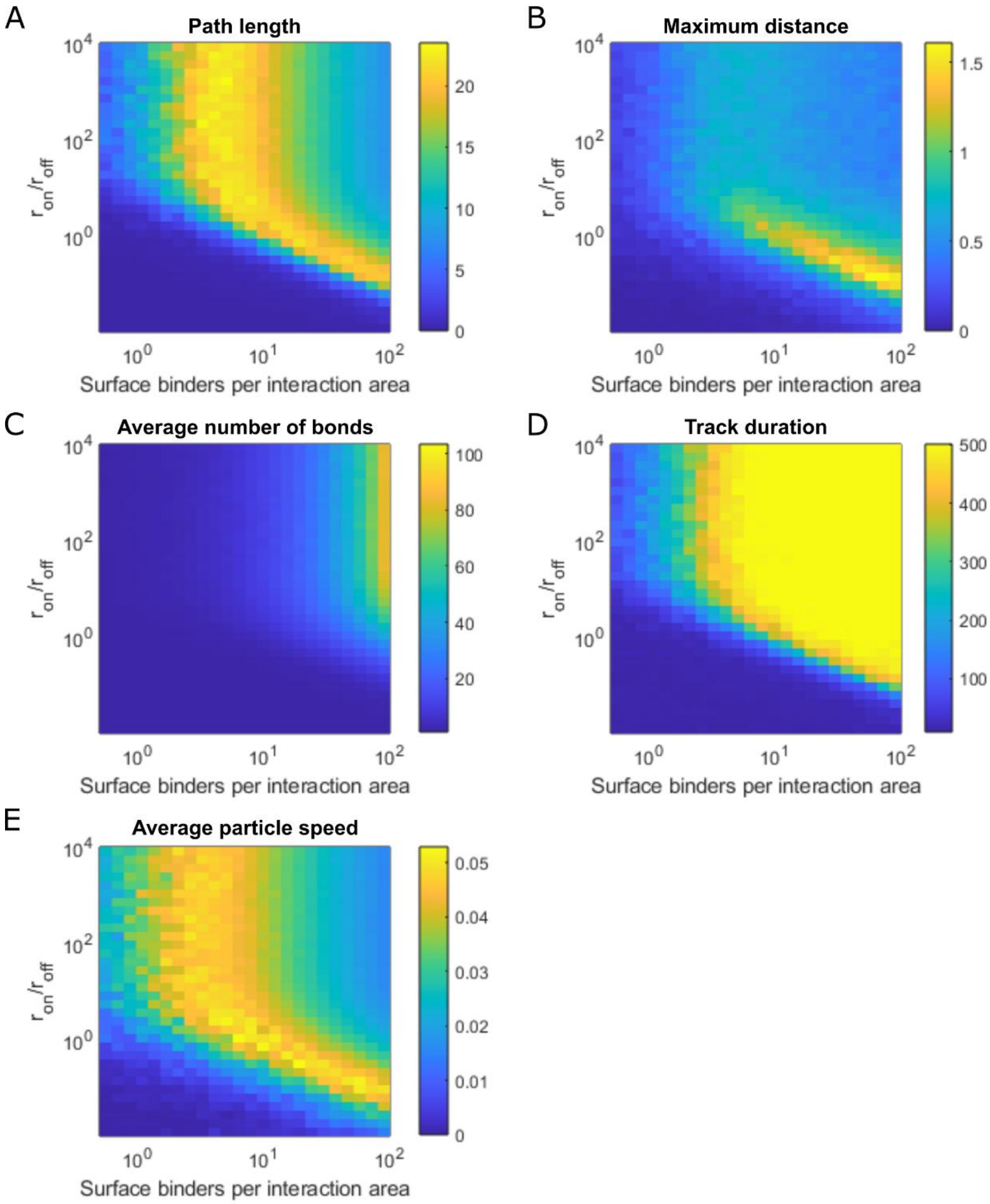

Figure S16. Heat maps showing mobility parameters of motion of a particle due to the creation and rupture of bonds with randomly distributed binders on the surface as a function of the ratio between association and dissociation rate and the average number of surface

binders in contact with the particle. (A) Length of the particle path; (B) Maximum distance between two points of the particle trajectory; (C) Average number of bonds established by the particle to the surface; (D) Average duration of the particle track in simulation steps ($dt = 0.1/k_{\text{off}}$); (E) Average instant speed of the particle. The instant speed is calculated as the length of each step divided by the simulation interval dt.

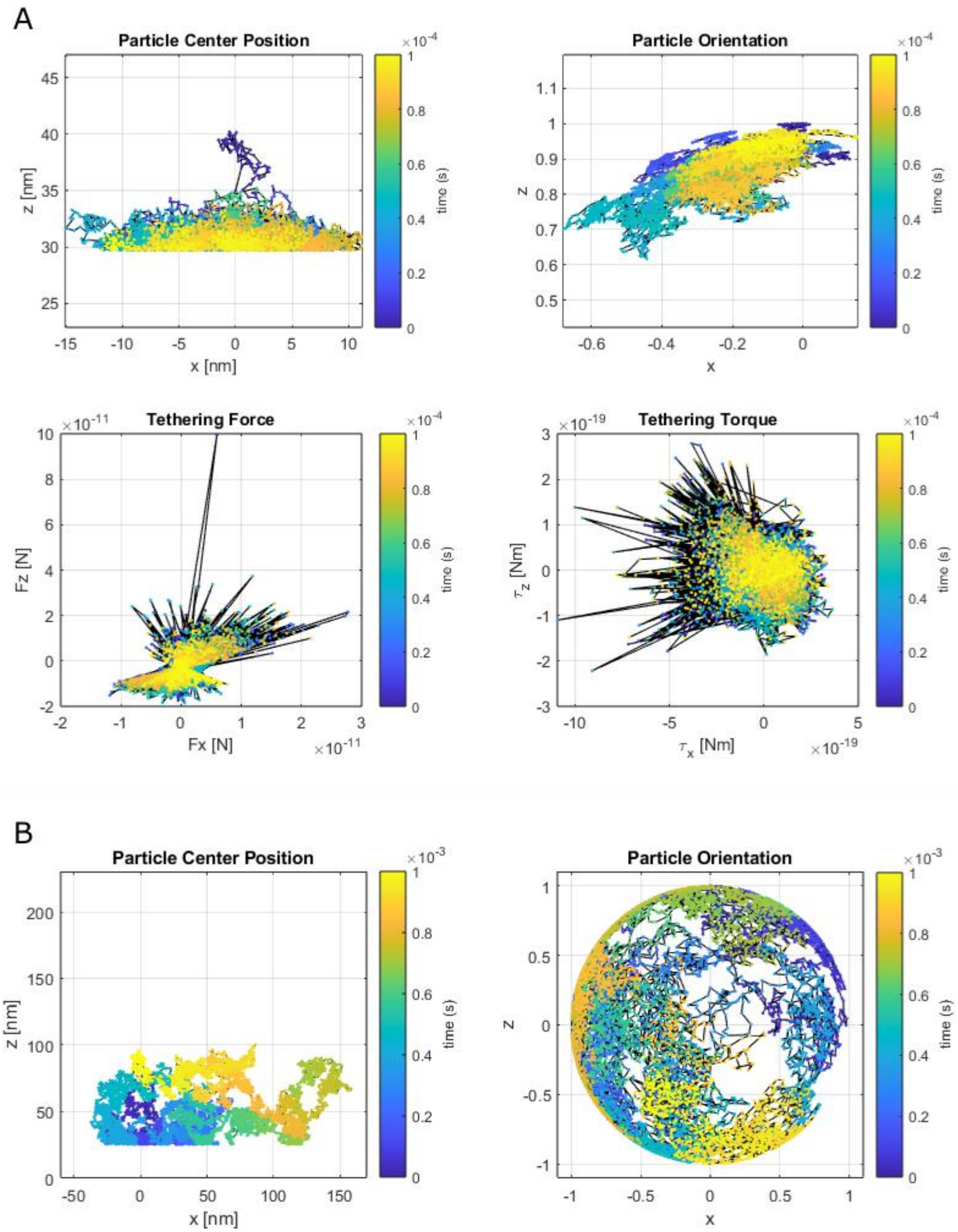

Figure S17. (A) *Top:* Position and orientation of a particle tethered to the surface by 5 heparin chains. *Bottom:* Cumulative force and torque exerted on the same particle by the heparin chains. (B) Position and orientation of a free particle simulated with the same algorithm as in (A). Initial position; $x = 0$ nm, $y = 0$ nm and $z = 40$ nm. The particle orientation is represented by a unit vector with an initial orientation at $t = 0$ s of [0,0,1].

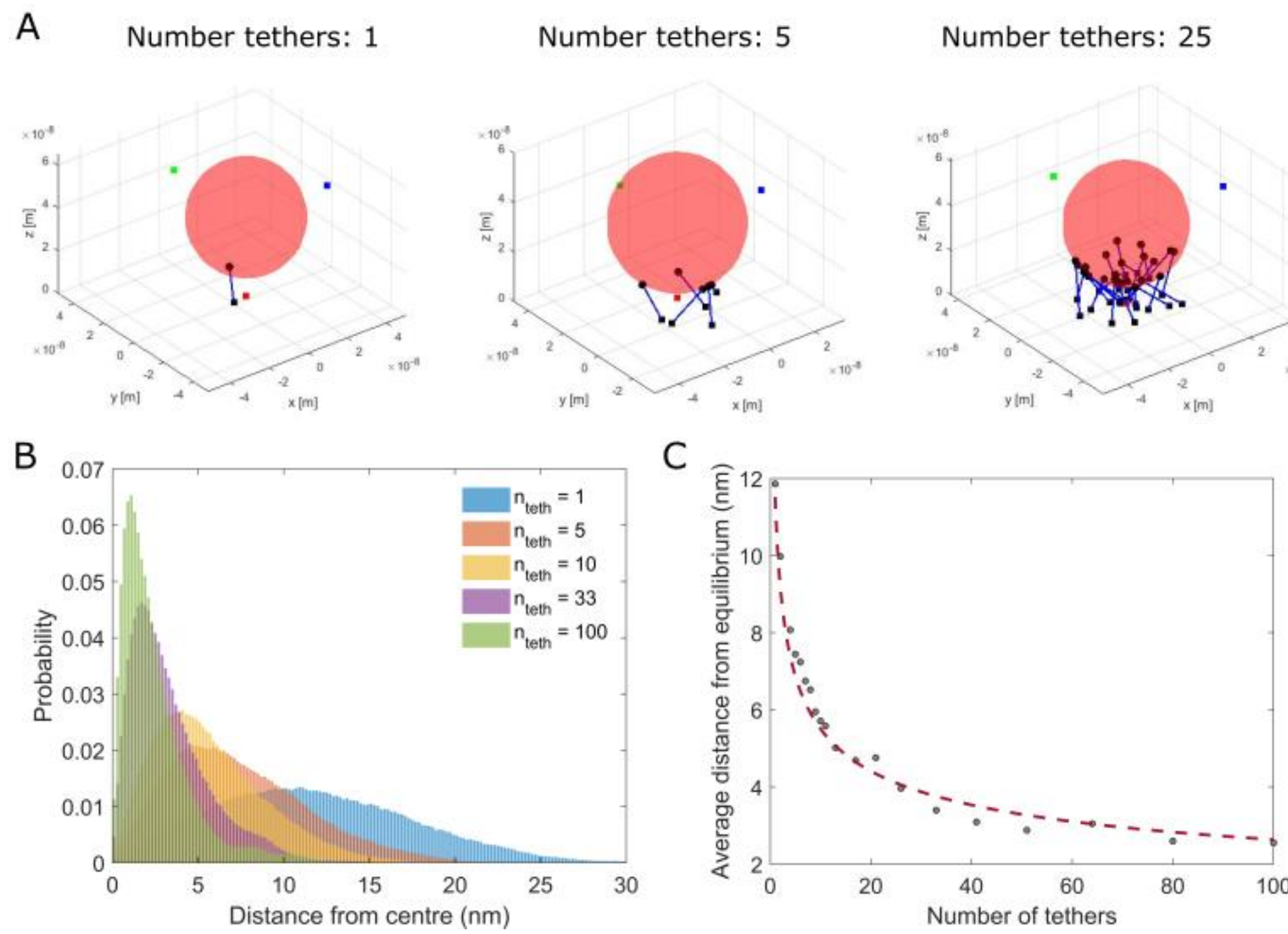

Figure S18. (A) Frames from the simulation of the Brownian motion of an HPV particle (red sphere) tethered to the surface by heparin chains (blue lines). The red, green and blue squares indicate the position of the particle along the three planes. The black dots, the attachment points of the heparin chains to the surface (squares) and the particle (circles). (B) Histogram showing the distribution of the distance from the equilibrium assumed by the HPV particle for several number of tethers (n_{teth}). (C) Mean distance from equilibrium calculated from the distribution in B as a function of the number of tethers (grey dots). Power law fit (red dashed line): $r_{\mathrm{avg}}(\mathrm{nm}) = 11.52 \cdot n_{\mathrm{teth}}^{-0.32}$.

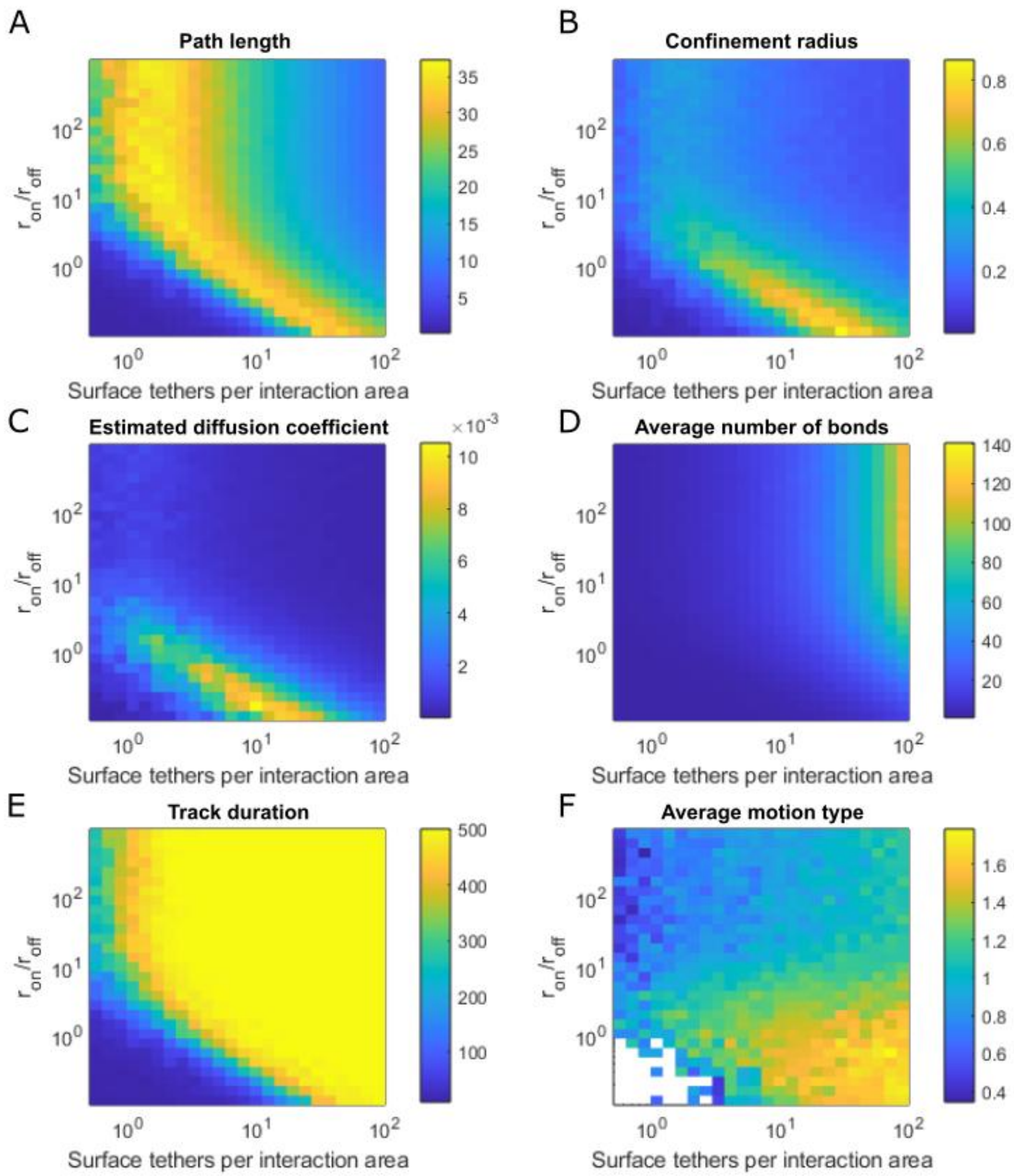

Figure S19. Heat maps showing mobility parameters of motion of a particle due to the creation and rupture of bonds with randomly distributed binders on the surface as a function of the ratio between association and dissociation rate and the average number of surface binders in contact with the particle. The Brownian motion of the tethered particle is considered by increasing the interaction radius by the average radius of the tethered Brownian motion experienced by the particle (Fig. S18B-C). (A) Length of the particle path; (B) Confinement radius calculated as half of the square root of the area of the convex hull calculated from the particle trajectory; (C) Estimated diffusion coefficient calculated as the area of the convex hull divided by the track length; (D) Average number of bonds established by the particle to the surface; (E) Duration of the particle track in simulation steps ($dt = 0.1/k_{\mathrm{off}}$); (F) Average motion type after sub/track sectioning (0: immobile, 1: confined, 2: free motion). White pixels indicate areas with less than 10 tracks successfully analyzed.

16. Effects of residual sulfation on particle binding and mobility

The parental heparin used in the study has on average 2.5 sulfation groups per disaccharide unit. Given that 3O sulfation is rare and most likely does not significantly contribute to the chain sulfation, and assuming that the occupation of the remaining sulfation sites is equally likely, we consider an occupation probability for each sulfation site of 80%. The enzymatic desulfation used to produce the heparin derivatives specifically targets single sulfation sites with an efficiency >90%. In particular, for N-deS, N-sulfation is reduced by 92% (according to supplier, Iduron, UK) on average, resulting in an occupation probability of N-sulfation sites of 6.4%. While drastically reduced, this is not negligible, with an 80% probability of having at least one N-sulfation on a 25-units chain (Table S6). Previous studies have proposed that the binding between HPV16 and heparan sulfate involves several binding pockets on the virus capsomer and at least 4 disaccharide units. It is unknown how many of these units are required to be sulfated to obtain a stable bond. However, we can determine the probability of observing 1 to 4 consecutive N-sulfates using a simple Markov model (see section 17 "Discrete Markov model for heparin sulfation"). In the case of parental heparin, virtually all chains possess two consecutive N-sulfation sites and 99.4% four. In the case of N-deS, this number is reduced to only 8.9% to 0.035%, respectively. Considering the grafting coverage of N-deS heparin (3.8×10^{12} cm^{-2}, Table 1), we can determine the surface coverage of binding motifs to be 0.34×10^{12} cm^{-2} to 1.33×10^{9} cm^{-2}, respectively. Despite the steep reduction, in the most conservative case of 4 consecutive N-sulfated units needed for stable binding, 2.25×10^{5} binders are present per field of view. Particles binding to this residual sulfation pockets will be stably bound to the surface and might contribute to the number of irreversibly bound particles (Fig. S20). In the case of the more diluted N-deS:PEG::1:5 used also in kinetic studies, we estimate a reduction of the GAG grafting coverage of around 25 times (see section 17) to as low as 10^{4} possible binders per field of view, strongly decreasing but not eliminating possible interactions mediated by N-sulfation.

17. Discrete Markov model for heparin sulfation

We model a heparin chain composed by M monomers as a series of an equivalent number of independent Bernoulli trials, where each monomer is either sulfated with probability p or not sulfated with probability $q = 1 - p$. We seek the probability that the chain contains at least one run of N consecutive sulfated monomers at a given sulfation site type(19,20). Reading monomers sequentially, if the next monomer is sulfated, we increase the current count of consecutive sulfated monomers by one; if it is not sulfated, the count resets to zero. We can thus build a Markov chain with states $S_0, S_1, \ldots, S_{N-1}, S_N^*$ where S_k (for $\mathrm{k} = 0, \ldots, \mathrm{N} - 1$) means that no run of N has occurred yet and the current run length is k, and S_N^* is an absorbing state indicating that a run of N has occurred, regardless of the current count.

With these assumptions, the one-step transition matrix T of dimension N+1×N+1 is:

$$T = \begin{bmatrix} q & p & 0 & \cdots & 0 & 0 \\ q & 0 & p & \cdots & 0 & 0 \\ \vdots & \vdots & \ddots & \ddots & \vdots & \vdots \\ q & 0 & \cdots & 0 & p & 0 \\ q & 0 & \cdots & 0 & 0 & p \\ 0 & 0 & \cdots & 0 & 0 & 1 \end{bmatrix},$$

i.e., from S_k (for $k \leq N - 1$) a sulfated monomer moves to S_{k+1} with probability p and a non-sulfated monomer moves to S_0 with probability q. S_N^* is absorbing, hence once in that state, the system will remain there.

We start before the first monomer in state S_0, which can be expressed with a row vector $v_0 = [1, 0, \cdots, 0]$ (dimension 1×N+1). After reading the m-th monomer the distribution of the probability of being in each state is:

$$v_m = v_{m-1}T = v_{m-2}TT = v_0T^m.$$

For a full chain, we obtain $v_M = v_0T^M$, and the probability $P_{N,M}$ of having encountered at least one run of at least N sulfated monomers is equivalent to the probability of being in state S_N^* after M transitions, i.e., the last element of v_M: $P_{N,M} = (v_M)_{N+1} = (v_0T^M)_{N+1}$.

Table S6: Summary of residual N-sulfation in N-deS substrates as a function of the number of consecutive N-sulfation sites required for stable HPV16 binding and grafting coverage, assuming random distribution of GAG chain on the SLB. Grafting coverage reflecting N-deS only, N-deS:PEG::1:1, N-deS:PEG::1:5, 38×10^{11} cm^{-2}, 6.4×10^{11} cm^{-2}, 1.6×10^{11} cm^{-2} (Table 1), respectively; particle contact area: 1800 nm^2.

Consecutive N-sulf. sites for binding	% Chains with binding sites	% Surface allowing multivalent binding		
		N-deS only	N-deS:PEG 1:1	N-deS:PEG 1:5
1	80.86	100	99.9	68.1
2	8.89	98.5	27.2	2.82
3	0.56	5.84	0.20	1.31×10^{-2}
4	3.5×10^{-2}	2.9×10^{-2}	8.3×10^{-4}	5.2×10^{-5}

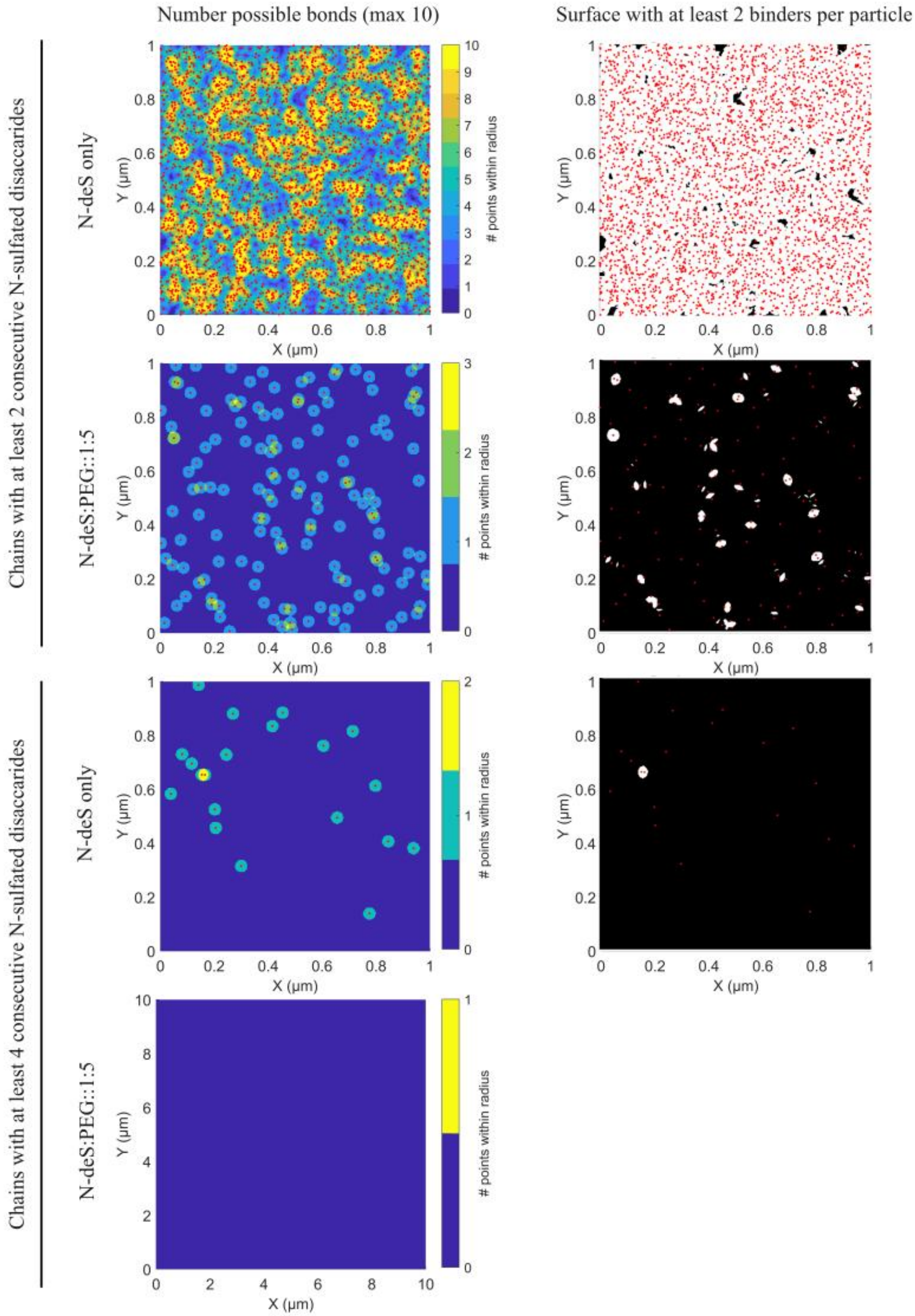

Figure S20. Simulated distribution of N-deS heparin chains maintaining 2 (*top four panels*) or 4 (*bottom 3 panels*) consecutive N-sulfated disaccharides (red dots) superimposed to a

heat map showing the maximum number of N-sulfation assisted bonds a HPV16 particle could form, *left*, and the fraction of surface that allows multivalent bonds resulting in immobile and irreversibly bound particle (white area), *right*. Grafting coverage reflecting N-deS only and N-deS:PEG::1:5, 38×10^{11} cm^{-2} and 1.6×10^{11} cm^{-2}, respectively; particle contact area: 1800 nm^2.

References

1. Bano, F., S. Banerji, M. Howarth, D. G. Jackson, and R. P. Richter. 2016. A single molecule assay to probe monovalent and multivalent bonds between hyaluronan and its key leukocyte receptor CD44 under force. *Sci. Rep.* 6(1):34176, doi: 10.1038/srep34176.
2. Thakar, D., E. Migliorini, L. Coche-Guerente, R. Sadir, H. Lortat-Jacob, D. Boturyn, O. Renaudet, P. Labbe, and R. P. Richter. 2014. A quartz crystal microbalance method to study the terminal functionalization of glycosaminoglycans. *Chem. Commun*. 50(96):15148-15151, doi: 10.1039/c4cc06905f.
3. Bano, F., L. Soria-Martinez, D. van Bodegraven, K. Thorsteinsson, A. M. Brown, I. Fels, N. L. Snyder, M. Bally, and M. Schelhaas. 2024. Site-specific sulfations regulate the physicochemical properties of papillomavirus-heparan sulfate interactions for entry. *Sci. Adv*. 10(40):eado8540, doi: 10.1126/sciadv.ado8540.
4. Emilsson, G., R. L. Schoch, L. Feuz, F. Hook, R. Y. Lim, and A. B. Dahlin. 2015. Strongly stretched protein resistant poly(ethylene glycol) brushes prepared by grafting-to. *ACS Appl. Mater. Interfaces*. 7(14):7505-7515, doi: 10.1021/acsami.5b01590.
5. del Castillo, G. F. D., G. Emilsson, and A. Dahlin. 2018. Quantitative Analysis of Thickness and pH Actuation of Weak Polyelectrolyte Brushes. *J. Phys. Chem. C*. 122(48):27516-27527, doi: 10.1021/acs.jpcc.8b09171.
6. Dahlin, A. B., M. Mapar, K. L. Xiong, F. Mazzotta, F. Höök, and T. Sannomiya. 2014. Plasmonic Nanopores in Metal-Insulator-Metal Films. *Adv. Opt. Mater*. 2(6):556-564, doi: 10.1002/adom.201300510.
7. Altgärde, N., E. Nilebäck, L. de Battice, I. Pashkuleva, R. L. Reis, J. Becher, S. Möller, M. Schnabelrauch, and S. Svedhem. 2013. Probing the biofunctionality of biotinylated hyaluronan and chondroitin sulfate by hyaluronidase degradation and aggrecan interaction. *Acta Biomater.* 9(9):8158-8166, doi: 10.1016/j.actbio.2013.05.031.
8. Lin, R., J. C. Kwok, D. Crespo, and J. W. Fawcett. 2008. Chondroitinase ABC has a long-lasting effect on chondroitin sulphate glycosaminoglycan content in the injured rat brain. *J. Neurochem*. 104(2):400-408, doi: 10.1111/j.1471-4159.2007.05066.x.
9. Berg, H. C. 1984. Random Walks in Biology. Princeton University Press.
10. Walker, D. M. 1998. The expected time until absorption when absorption is not certain. *J. Appl. Probab.* 35(4):812-823, doi: 10.1239/jap/1032438377.
11. Migliorini, E., D. Thakar, R. Sadir, T. Pleiner, F. Baleux, H. Lortat-Jacob, L. Coche-Guerente, and R. P. Richter. 2014. Well-defined biomimetic surfaces to characterize glycosaminoglycan-mediated interactions on the molecular,

supramolecular and cellular levels. *Biomaterials*. 35(32):8903-8915, doi: 10.1016/j.biomaterials.2014.07.017.

12. Pavlov, G., S. Finet, K. Tatarenko, E. Korneeva, and C. Ebel. 2003. Conformation of heparin studied with macromolecular hydrodynamic methods and X-ray scattering. *Eur. Biophys. J. Biophy*. 32(5):437-449, doi: 10.1007/s00249-003-0316-9.
13. Knappe, M., S. Bodevin, H. C. Selinka, D. Spillmann, R. E. Streeck, X. S. Chen, U. Lindahl, and M. Sapp. 2007. Surface-exposed amino acid residues of HPV16 L1 protein mediating interaction with cell surface heparan sulfate. *J. Biol. Chem*. 282(38):27913-27922, doi: 10.1074/jbc.M705127200.
14. Sapp, M., and M. Bienkowska-Haba. 2009. Viral entry mechanisms: human papillomavirus and a long journey from extracellular matrix to the nucleus. *FEBS J*. 276(24):7206-7216, doi: 10.1111/j.1742-4658.2009.07400.x.
15. Rubinstein, M., and R. H. Colby. 2003. Polymer Physics. Oxford University Press.
16. Zhang, B., J. Zerubia, and J.-C. Olivo-Marin. 2007. Gaussian approximations of fluorescence microscope point-spread function models. *Appl. Opt.* 46(10):1819-1829, doi: 10.1364/AO.46.001819.
17. Becker, M., D. V. Conca, N. Dorma, N. Mistry, E. Hahlin, L. Frangsmyr, M. Bally, N. Arnberg, and G. Gerold. 2023. Efficient clathrin-mediated entry of enteric adenoviruses in human duodenal cells. *J. Virol*. 97(10):e0077023, doi: 10.1128/jvi.00770-23.
18. Volpe, G., S. Gigan, and G. Volpe. 2014. Simulation of the active Brownian motion of a microswimmer. *Am. J. Phys*. 82(7):659-664, doi: 10.1119/1.4870398.
19. Moshkovitz, I., and Y. Barron (2025). The Waiting Time Distribution of Competing Patterns in Markov-Dependent Bernoulli Trials. *Axioms*. 14(3):221, doi: 10.3390/axioms14030221.
20. Coleman, B. D., and T. G. Fox. 1963. General theory of stationary random sequences with applications to the tacticity of polymers. *J. Polym. Sci. Part A: Gen. Pap*. 1(10):3183-3197, doi: 10.1002/pol.1963.100011013.
