# Supplementary material for: Multivalency influences virus dynamics at the glycocalyx: A study of glycosaminoglycan binding in HPV16 entry
Source: Biophys J. 2026 May 12;125(14):3498–515. doi: 10.1016/j.bpj.2026.05.014 (PMC13507399; doi:10.1016/j.bpj.2026.05.014)
Supplement: Supplementary file 2 [file mmc2.pdf]

Biophysical Journal
Article

Biophysical Society

Multivalency influences virus dynamics at the glycocalyx: A study of glycosaminoglycan binding in HPV16 entry

Dario Valter Conca,[1,5] Fouzia Bano,[1,5] Yara Abidine,[1] Laura Soria Martinez,[2] Kerstin Seier,[1] Justas Svirelis,[3] Andreas Dahlin,[3] Mario Schelhaas,[2] and Marta Bally[1,4,*]

[1]Department of Clinical Microbiology, Wallenberg Centre for Molecular Medicine and Umeå Centre for Microbial Research, Umeå University, Umeå, Sweden; [2]Institute of Cellular Virology, ZMBE, University of Münster, Münster, Germany; [3]Department of Chemistry and Chemical Engineering, Chalmers University of Technology, 412 96 Gothenburg, Sweden; and [4]Science for Life Laboratory (SciLifeLab), Umeå University, Umeå, Sweden

[5]These authors contributed equally

ABSTRACT Virus attachment at the cell surface often involves the establishment of multiple ligand-receptor interactions between viral capsid proteins and glycans in the glycocalyx. Multivalency likely contributes to strengthening and fine-tuning virus dynamics at the cell surface to optimize entry. Here, we present experimental and theoretical frameworks to describe how virus attachment, detachment, and diffusion at the cell surface are modulated by multivalency. We focus on human papillomavirus 16 (HPV16), a leading cause of cervical cancer and its multivalent interactions with heparan sulfate (HS), a ubiquitous cell-surface glycan. Using single-particle tracking microscopy, we investigate the dynamic behavior of HPV16 particles interacting with multiple HS chains through a glycocalyx mimic consisting of end-tethered heparin chains that were selectively desulfated. We further establish a theoretical framework to predict the dynamic behavior of individual virus particles interacting with multiple cellular receptors and apply it to the HPV16-HS binding system using previously published association and dissociation rates of the individual capsomer-glycan bonds. Our experiments reveal that N-sulfation is essential to ensure HPV16 association and validate our theoretical prediction that at biologically relevant timescales, the lifetime of monovalent interaction primarily influences the apparent particle attachment behavior. Conversely, the nature of the sulfate group had a marginal effect on particle dissociation in the experiments, due to the great influence of multivalency on the particle's interaction lifetime. Experiments and simulations also indicate that the mobility of HPV16 particles on heparin surfaces is highly restricted due to the relatively high affinity of the monovalent interactions, suggesting that particle motion due to the creation and rupturing of individual bonds is unlikely key in HPV16 recruitment. Together, these findings provide quantitative mechanistic insights into how virus-glycan interactions are modulated. They highlight the importance of HS chemistry in modulating early HPV16 engagement and suggest new targets against viral binding.

SIGNIFICANCE To optimize entry, viruses modulate their dynamic behavior at the cell surface through multivalent interactions with glycans. In this work, we establish a theoretical framework describing how individual ligand-receptor interactions culminated into a fine-tuned virus attachment, detachment, and diffusion behavior in the glycocalyx. Theory and simulations are validated by experimental investigations of the multivalent interaction of individual HPV16 particles with heparin, which complement previous investigations of individual capsomer-heparin bonds. The presented theoretical frameworks represent a mechanistic basis for molecular-level understanding of the early stages of viral attachment and can be readily applied to other viruses. Our experimental findings contribute to our understanding of HPV16's biology and will facilitate the design of therapeutic strategies targeting HS sulfation to block HPV16 infection.

INTRODUCTION

The first step of viral infection requires the attachment of a virus to the cell surface, a process eventually triggering its uptake by the host cell. The viral attachment process is

Submitted December 3, 2025, and accepted for publication May 8, 2026.

*Correspondence: marta.bally@umu.se

Editor: Guy Genin.

https://doi.org/10.1016/j.bpj.2026.05.014

© 2026 The Authors. Published by Elsevier Inc. on behalf of Biophysical Society.
This is an open access article under the CC BY license (http://creativecommons.org/licenses/by/4.0/).

mediated by specific biomolecular recognition events occurring between viral proteins and cellular biomolecules. Given that viruses are often highly symmetric and carry multiple copies of a binding protein, they interact by establishing multiple bonds with molecular species at the cell surface. In this context, the term bond is intended as the ensemble of atomic-scale interactions that results in a transient association between individual viral binding proteins and individual viral receptors on the cell surface. Here, multivalency is likely to offer exquisite possibilities to fine-tune the dynamic behavior of a virus at the cell surface and to optimize the entry process. Many viruses initially bind carbohydrates in the glycocalyx, the sugar coat covering the cell surface, before transferring to more specific entry-receptors that trigger virus uptake.[1,2] Such protein-carbohydrate interactions are often relatively weak but can be significantly strengthened through multivalency, thereby modulating the dynamic characteristics of the initial contact of a virus approaching a target cell.[3–5] While the importance of multivalency in tuning viral interactions at the cell surface is being increasingly acknowledged,[5–7] theoretical and experimental frameworks quantitatively relating the characteristics of individual ligand-receptor interactions to the dynamic behavior of a virus particle interacting through multivalent interactions have remained limited. In this context, *in vitro* glycocalyx models such as surface-immobilized glycosaminoglycan (GAG)[8–10] and synthetic glycopolymers[11,12] have shown a great potential; they have been previously used to study how glycan density and spatial organization can modulate viral binding kinetics.

Heparan sulfate (HS), a ubiquitous GAG found in the glycocalyx and extracellular matrix, mediates a variety of cellular processes involving multivalent interactions with proteins at the cell surface, for example cell signaling, cell adhesion, or the regulation of immune responses.[13,14] Conversely, many viral pathogens exploit them to initiate infection. Viruses binding to HS include human papillomaviruses (HPVs), herpes simplex virus (HSV), human immunodeficiency virus, SARS-COV-2, and many more.[8,15,16] HS is a linear polysaccharide composed of a repeating disaccharide backbone, made of glucuronic or iduronic (IdoA) acid and N-acetylglycosamine (GlcNAc) units. This periodic structure is often modified by the addition of sulfation groups in well-defined positions, such as N-sulfated glucosamine, and sulfation of the C6 and C3 positions of GlcNAc and the C2 position of IdoA (6-O, 3-O, and 2-O sulfation, respectively) (Figure 1A).[17] HS is synthesized and sulfated enzymatically, resulting in highly heterogeneous glycan chains and sulfation patterns in terms of the level and type of modifications, whose nature and distribution are likely cell and tissue specific. These structural and spatial heterogeneities of HS chains on mammalian cells are likely key in defining the host specificity, tissue tropism, and severity of disease caused by many GAG-binding viruses.[18,19]

HPVs, a large family of small (~50 nm in diameter) nonenveloped DNA viruses, use HS for initial attachment at the cell surface.[20–23] These viruses are of major public health concern, as they can be etiological agents for several anogenital and oropharyngeal cancers, with HPVs detected in more than 90% of invasive cervical cancer cases.[24,25] HPV type 16 (HPV16), in particular, represents the highest-risk genotype and is responsible for approximately 60% of cervical cancer cases worldwide, across cancer subtypes and geographic regions.[26] HPV has an icosahedral capsid composed of 72 pentamers of the major capsid proteins L1 and up to an equal number of minor capsid protein L2.[27,28] Binding to HS occurs through the engagement of several binding pockets located on the HPV16 capsomer.[20,27] This interaction is of particular importance to the infection process as it triggers a conformational change in the capsid, thereby structurally activating it for further steps of infections.[29,30] Specifically, structural activation promotes a cascade of enzymatic reactions that further modify the capsid, leading to a loss of the virus' ability to bind to HS and its transfer to an entry receptor whose identity remains elusive.[31] In this process, specific sulfation groups and patterns of HS have been suggested to play a key role in mediating functional binding and structural activation of HPV16.[23,29,30] The entry process of HPV16 is uniquely slow. While enzymatic processing of the capsid likely starts within minutes after attachment, the whole entry process is known to take several hours.[31,32]

In a recent study, we have used atomic force microscopy (AFM)-based single-molecule force spectroscopy (SMFS) to quantify the association (k_{on}) and dissociation rate constants (k_{off}) of individual ligand-receptor bonds occurring between a single HPV16 virus particle and single heparin chains. Using either heparin, a secreted highly sulfated structural analog of HS, or various desulfated heparin derivatives, we showed that all types of sulfation contribute similarly to the initial engagement of the capsomer with a heparin chain, while N-sulfation is crucially needed to increase the interaction's lifetime.[30] Here, we focus on the binding kinetics and diffusion of HPV16 particles in the context of their multivalent interaction with heparin. Using a glycocalyx-mimetic platform, produced by end-grafting GAG chains on a surface, we quantify HPV16 particle interaction kinetics and diffusion on this surface using single-particle tracking (SPT) of the particles bound to the surface by total internal reflection fluorescence (TIRF) microscopy. Additionally, we present a theoretical framework that links the properties of single capsomer-glycan interactions to the characteristics of a multivalent particle-glycan interaction. We confirm that N-sulfates play a key role in mediating the interaction between HPV16 and heparin. However, at timescales of relevance to the biological processes under investigations, i.e., minutes to hours, the lifetime of the monovalent interaction primarily influences the apparent attachment behavior of the multivalent particle, resulting in a nearly 1 order of magnitude increase in particle binding rates for HPV16 when N-sulfation is present. Conversely, the nature of the sulfate group had a minimal effect on the

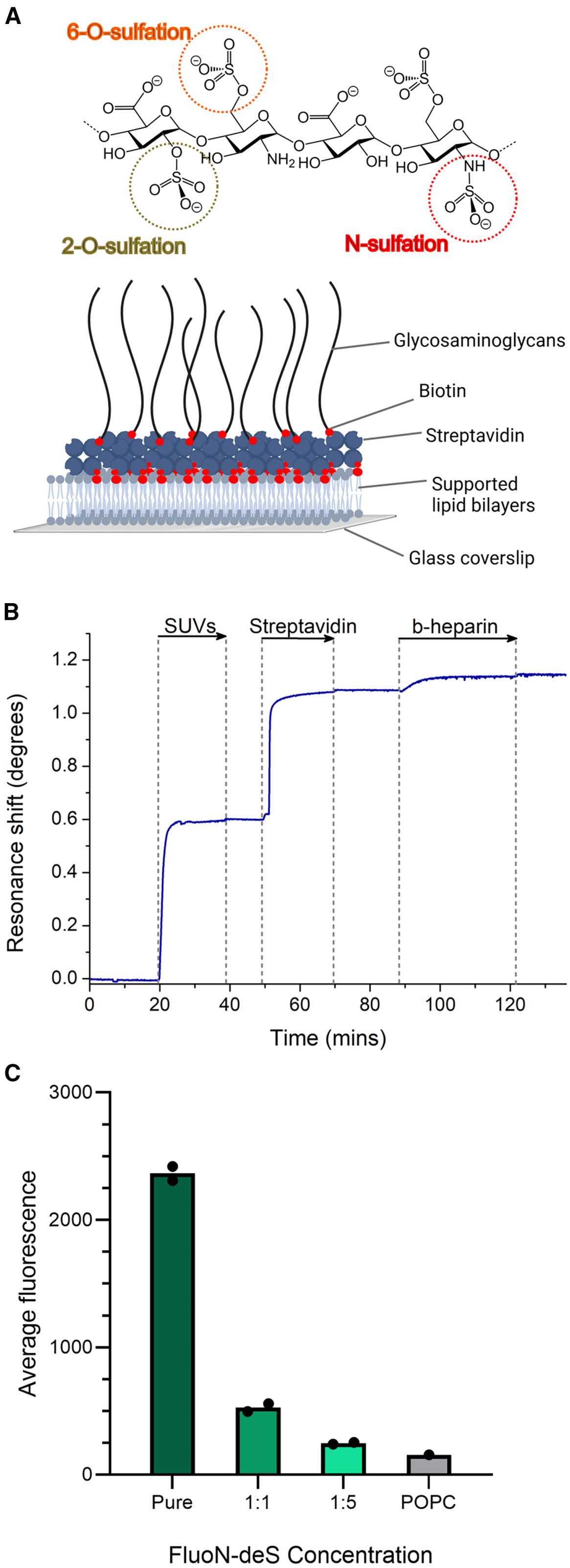

Figure 1. Characterization of GAG platform. (A) Chemical structure of two disaccharide units of heparin with sulfation groups (top) and illustration of the design of a surface displaying end-grafting immobilization of glycosaminoglycans via biotin on a monolayer of streptavidin formed on a supported lipid bilayer (not to scale, bottom). (B) A representative SPR sensogram (670 nm) for the step-by-step assembly of heparin films. Stability of each immobilized biomolecule is demonstrated by no further shift during rinsing with PBS buffer. (C) Mean of the fluorescence images (Figure S3B) from 2 identically prepared wells for each condition except POPC (black dots). Conditions from left to right: full layer of FluoN-deS heparin (pure); 1:1 (molar ratio) mixture of biotinylated FluoN-deS and biotinylated PEG (1:1); 1:5 (molar ratio) mixture of biotinylated FluoN-deS and biotinylated PEG (1:5); POPC surfaces exposed to biotinylated-fluorescent N-deS heparin (POPC) as a negative control.

dissociation, with particle dissociation rate constants in the same order of magnitude for all cases. In addition to this, theoretical modeling and experimental investigations of virus motion on surfaces presenting immobile heparin ligands reveal that in all cases investigated here, the monovalent capsomer-heparin interaction is too strong to permit multivalency-assisted diffusion of the particle on the surface, i.e., motion of the particles on the surface presenting immobile receptors due to the creation and rupturing of individual capsomer-heparin bonds.

MATERIALS AND METHODS

Buffer and proteins

All experiments were performed in phosphate-buffered saline (PBS) buffer at pH 7.4, which was prepared by dissolving 1 PBS tablet (Medicago AB, Uppsala, Sweden) in 1 L Milli-Q water (Millipore integral system, Molsheim, France) and filtering with 0.2 μm filters (Sarstedt, Germany) before use.

Lyophilized streptavidin (Sigma-Aldrich, St. Louis, MO) was dissolved in Milli-Q water at 5 mg/mL and stored at −80°C. Thawed aliquots were used within a few days. Alexa Fluor 488 succinimidylester was from Thermo Fisher Scientific.

Virus production and labeling

HPV16 pseudoviruses (PsVs) containing a GFP reporter plasmid were produced using 293TT cells as previously described.[30] In brief, 293TT cells were transfected with p16SheLL and pCIneo. After 48 h, cells were harvested and lysed. For optimal maturation, lysates were incubated for a further 24 h with 25 mM ammonium sulfate (pH 9.0) at 37°C. PsVs were purified on a 25%–39% linear Optiprep gradient (Sigma-Aldrich).

HPV16 PsVs were covalently labeled with Alexa Fluor dyes as previously described.[30,33] In short, HPV16 PsVs were incubated for 1 h with NHS ester of the fluorescent dye Alexa Fluor 488 at a 1:8 molar ratio (L1/dye). Labeled HPV16 PsVs were separated from free dye by an Optiprep step gradient (5%/39%). Stock concentration of WT HPV16 was 130 μg/mL, which was determined after SDS-PAGE and Coomassie staining by densitometry relative to a standard of BSA. It should be noted that throughout this

study, all mentions of particles or viruses specifically refer to HPV16 PsVs.

GAGs

All GAGs used in this study are listed in Table S1 together with their biochemical characteristics. Lyophilized heparin (HEP001) and 3 selectively desulfated heparins (N-desulfated, DSH003/N; 2-O-desulfated, DSH001/2; 6-O-desulfated, DSH002/6) were purchased from Iduron, UK. According to the supplier's product data sheet, there was ∼92% reduction in N-sulfates for DSH003/N, ∼90% reduction in 6-O sulfates with 25% loss of 2-O sulfates for DSH002/6, and ∼95% reduction in 2-O sulfates for DSH001/2 as compared with parental heparin. Lyophilized end-biotinylated hyaluronan (HA) was purchased from INNOVENT e.V. Technologieentwicklung, Jena, Germany (KS855). All GAGs were dissolved in Milli-Q water at the desired concentration and gently shaken overnight at 4°C before aliquoting. Heparin and the selectively desulfated variants were biotinylated via oxime ligation at their reducing end as described previously.[30,34] Stock solutions of all GAGs were aliquoted and stored at −20°C after biotinylation. Thawed aliquots were used within a few weeks.

Lipid vesicle preparation

1-palmitoyl-2-oleoyl-sn-glycero-3-phosphocholine (POPC), cat. no. 850457P, and 18:1 Biotinyl Cap PE (DOPE-Cap-B), cat. no. 870273P, were purchased from Avanti Polar Lipids (Alabaster, AL, USA). Small unilamellar vesicles (SUVs) were prepared from a mixture of POPC:DOPE-Cap-B (90:10, molar ratio) in PBS at a stock concentration of 2 mg/mL by extrusion through a 50 nm polycarbonate membrane at least 21 times using a mini extruder equipped with a 1 mL syringe (Avanti Polar Lipids; 610020 and 610017) as previously described.[9] Stocks were stored at 4°C under N_2 and used within a few weeks.

Surface plasmon resonance

Surface plasmon resonance (SPR) chips with ∼2 nm Cr and 50 nm Au were prepared by electron beam heated physical vapor deposition (Lesker PVD 225) on glass substrates (20 × 12 mm; Bionavis), which were cleaned beforehand with RCA-2 (1:1:5 volume of conc. HCl:H_2O_2 [30%]:H_2O [ultrafiltered at 18.6 MΩs] at 80°C) and 50 W O_2 plasma at 250 mTorr. Moreover, a thin silica layer (∼11 nm) was deposited on top of the gold layer with atomic layer deposition (Oxford FlexAL) at 300°C by using a bis(t-butylamino)silane (BTBAS) as a precursor and oxygen as processing gas. Sensor surfaces were incubated in a UV O_3 chamber for at least 30 min before use.

SPR Navi 220 A instrument (BioNavis) is equipped with three lasers (wavelengths of 785 nm, 670 nm, and 980 nm), which induce different decay lengths of sensing fields. The SPR spectra can be acquired in parallel from 2 flow channels in a polyether ether ketone flow cell. The running buffer was PBS (Merck), which was degassed using a combined setup of vacuum pump (DIVAC 1.4HV3C) and sonicator bath (35 kHz). The flow rate was between 5 and 20 μL/min, and the temperature was kept at 25°C. The quantification of mass coverage on silica-coated gold sensors was done in a similar manner as reported before[35] (see supplemental information, Section S2). In brief, the SPR signals were used to calculate the surface molecular grafting coverage, i.e., the number of GAG chains per unit area, by employing independently determined parameters (molar refractivity, bulk sensitivity, and plasmonic field decay length). The grafting coverage was calculated based on the mass density and the molecular weight of biomolecules. The average spacing between GAG chains was calculated as the inverse of the square root of the grafting coverage, thus assuming a square arrangement.

Condition for self-assembling GAGs films in SPR experiments was as follows: (1) the formation of supported lipid bilayer (SLB) was done by the injection of SUVs containing 10 mol % biotin functionalized lipids at concentration of 100 μg/mL with flow rate of 20 μL/min; (2) next the streptavidin monolayer was formed by exposing SLBs at concentration of 50 μg/mL with flow rate of 12.5 μL/min; and (3) finally the binding of biotinylated GAGs or biotinylated polyethylene glycol (PEG) was performed at concentration of 10 μg/mL with flow rate of 5 μL/min.

Fluorescently labeled biotinylated GAG film

In our system, we control the grafting coverage of GAGs by premixing them with biotinylated PEG (biotin-PEG-SH; biotinylated PEG with thiol end group; molecular weight of 3.4 kDa; RAPP polymere, Germany). The surface coverage of GAG in the GAG-PEG mixture was deduced from SPR measurements (Table S3) and the fluorescence intensity of surface-immobilized fluorescently labeled N-desulfated (FluoN-deS). For details on how the N-desulfated heparin was fluorescently labeled with an Alexa Fluor 488 derivative (FluoN-deS), see supplemental information, Section S6. To prepare pure FluoN-deS and mixed FluoN-deS:PEG layers, 8 wells were created on cleaned glass coverslips and prepared with a streptavidin layer as described below in TIRF microscopy. The biotinylated heparin derivatives, either alone or mixed with biotin-PEG-SH, were immobilized on the surface by incubating them with the solution for 30 min at the following concentrations: biotinylated FluoN-deS at 10 μg/mL (FluoN-deS in Figure 1C) or biotinylated FluoN-deS (1 μg/mL) mixed with biotin-PEG-SH at the concentration of either 0.227 μg/mL or 1.13 μg/mL to have the molar ratios of 1:1 and 1:5, respectively. All experiments were performed in duplicate. Pure POPC SLBs with and without incubation with biotinylated FluoN-deS (10 μg/mL for 30 min) were used as negative controls.

Each sample was imaged at 12 random positions on the surface using an inverted Nikon Eclipse Ti2-E microscope. Assuming linearity between the GAG density at the surface and the fluorescent signal, we can calculate the surface coverage (SC) of the GAG-PEG mixture from the SC of immobilized pure GAG, as measured with SPR, using the following formula: $SC_{\text{FluoN-deS:PEG::1:}x} = \frac{I_{\text{FluoN-deS}} - I_{\text{POPC}}}{I_{\text{FluoN-deS:PEG::1:}x} - I_{\text{POPC}}} SC_{\text{FluoN-deS}}$, where SC_{surface} and I_{surface} are the surface coverage and intensity of the corresponding surface, respectively, and X represents either 1 or 5.

TIRF microscopy

The surfaces for TIRF microscopy were prepared using a mixture of biotinylated GAGs and PEG. For TIRF microscopy-based kinetic and diffusion experiments, glass coverslips were cleaned and UV treated as described previously.[30] Briefly, round microscope glass coverslips (24 mm diameter, Deckglaser, Germany) were clened with 1:6 (v/v) solution of 7x detergent (MP Biomedicals, CA) and Milli-Q water at near boiling temperature for 2 h. The slides were then thoroughly rinsed and stored in Milli-Q water. Immediately prior to use, the slides were rinsed again with Milli-Q water, dried with N_2, and treated with UV (UV Ozone Cleaner - ProCleaner Plus, BioForce Nanosciences, IL) for 30 min. Then, two slides were glued to a custom-designed Teflon holder using Twinsil glue (Picodent, Germany) to create 6 separated wells that can hold a maximum volume of 250 μL. SLBs were formed by spontaneous rupture of liposomes composed of 90:10 (molar ratio) POPC and DOPE-Cap-B at a concentration of 100 μg/mL. SLBs were then rinsed extensively and incubated for 30 min with POPC vesicles at 50 μg/mL to fill up any potential gaps in the SLBs. After removing excess unbound vesicles, each well was incubated with streptavidin in PBS at a final concentration of 50 μg/mL for 30 min. After rinsing, the wells were incubated with 2% v/w paraformaldehyde (PFA) from VWR for 15 min. Following a rinsing step with PBS, a solution mixture of desired biotinylated GAGs and biotin-PEG-SH was added to each well at molar ratio of 1:5 for 30 min. One well was incubated with biotin-PEG-SH only at final concentration of 25 μg/mL for 30 min. This well was used as negative control. After rinsing with PBS, all wells were incubated with 50 μg/mL pure POPC vesicles for ~1 h. The HPV16 particles were diluted (~5,714× stock) and added to the wells in 50 μg/mL pure POPC vesicles in PBS, to further minimize nonspecific adsorption. Rinsing was performed by dispensing 150 μL of PBS into each well while maintaining a residual volume of 50 μL to prevent the wells from drying out. This procedure was repeated 14 times for each rinsing step. All reagents were prepared at twice the working concentration, and 50 μL of reagent was added to the 50 μL residual volume already present in each well. For N-deS and HA films, we used 3 times higher virus concentration than the rest of the conditions. A high density of biotin within the SLBs was used to ensure the formation of a densely packed and immobile layer of streptavidin molecules after PFA fixation. This prevents clustering of HS around the particles as well as particle motion arising from HS diffusion.

TIRF imaging of single virus particles was as follows: an inverted Nikon Eclipse Ti2-E microscope (Nikon, Japan) equipped with an Apo TIRF 60× oil-immersion objective (numerical aperture: 1.49) together with a Lumencor SPECTRA III light source, a multi-band filter cube 86012v2 DAPI/FITC/TxRed/Cy5 (Nikon), a Ti2-LAPP laser system, and a Prime 95 B sCMOS camera (Photometrics Teledyne, USA) was used to perform all TIRF experiments.

Single-particle kinetics and equilibrium fluctuation analysis (EFA) was as follows: for binding kinetic data, time-lapse videos (129 × 129 μm^2 field of view) at a frame every 15 s for 1 h were recorded with labeled HPV16 particles after 75 min preincubation to reach equilibrium unless otherwise stated. This experimental time frame was deemed to be a good compromise between time resolution and photobleaching of the particles, while allowing observation of virus binding events at timescales of relevance to the infection process.

The time-lapse movies were automatically analyzed using an in-house MATLAB script.[8] Briefly, particles were detected in all frames using a 2D peak detection algorithm. Only particles exceeding a user-defined prominence threshold were considered. Particles were then linked between frames using a nearest-neighbor approach with a maximum linking distance of 2 pixels. According to their fluorescence intensity and permanence on the surface, particles were categorized as either "firmly bound" (residence time more than 3 frames), "loosely bound" (residence time less than 3 frames), or "bleached" (slow and steady decrease in intensity below the detection threshold with no sudden drop suggesting detachment).

Loosely bound and bleached particles were disregarded in the analysis, and only detachment of particles that landed onto the surface in the first half of each video was considered. To improve statistics, we measured the cumulative value of 5 individual movies corresponding to 5 randomized locations.

The particle association kinetics to the GAG surface can be determined by considering the cumulative sum of the detected arrival events over time. In particular, under equilibrium conditions, i.e., in absence of global diffusion limitations, the slope of the cumulative sum can be related to the particles' association rate constant (k_{on}) using the following expression:

$$\frac{dN^+}{dt} = k_{\text{on}} C_b \theta \tag{1}$$

Here, $N^+[\text{cm}^{-2}]$ is the cumulative number of particles landed since the beginning of the observation period, C_b [M] is the virus bulk concentration, and $\theta[\text{cm}^{-2}]$ is the surface density of free receptors on the surface. It is important to note that determination of a value for k_{on} in the complex case of an HPV16 particle binding to GAGs is complicated by the fact

Table 1. Surface grafting densities and average spacing between GAG chains for different GAG surfaces used in the study

GAG:PEG surface molar ratio	Mass density[a] (ng/cm^2)	Grafting coverage ($10^{11}/cm^2$)	Spacing between chains (nm)
Heparin:PEG:1:5	2.8 ± 0.7	1.1 ± 0.3	29.9 ± 3.7
2-O-deS:PEG:1:5	3.2 ± 0.7	1.3 ± 0.3	27.7 ± 3.2
6-O-deS:PEG:1:5	3.8 ± 2.2	1.5 ± 0.9	25.6 ± 7.4
N-deS:PEG:1:5	3.9 ± 1.0	1.6 ± 0.4	25.2 ± 3.3
Heparin:PEG:1:1	11.3 ± 3.1	4.5 ± 1.2	14.8 ± 2.0
N-deS:PEG:1:1	16.0 ± 4.5	6.4 ± 1.8	12.5 ± 1.8
N-deS	94.9 ± 23.4	38.1 ± 9.0	5.1 ± 0.6

Values ± standard deviation were obtained from 2 independent experiments except HA.

[a]Grafting density values were calculated from SPR data (Table S3) with the factor of 24 ± 3 for 1:5 and 6 ± 1 for 1:1 obtained from fluorescently labeled biotinylated GAG assay.

that the receptor cannot be unambiguously identified. Here, we assume that each GAG chain is a single receptor. Additionally, in our experiments, the number of grafted heparin chains exceeds bound particles by $>10^4$ ($>2 \times 10^7$ heparin chains and $<10^3$ bound particles per field of view). The number of occupied receptors is thus negligible, and we can ignore saturation effects and approximate θ to the total GAG grafting coverage (Table 1). Furthermore, because of a well-recognized artifact associated with difficulties in accurately identifying all particles initially present on a crowded surface, the first 150 s were discarded from the linear fit.

In addition, the particle residence time distribution was used to calculate the cumulative survival probability of the bond to the surface, i.e., the percentage of particles still bound to the surface at any time from attachment. This was then fitted to a single exponential function to determine the particle's dissociation rate constant (k_{off}):

$$f(\text{t}) = \text{A}\left[(1 - \text{B})e^{-k_{\text{off}}t} + \text{B}\right] \tag{2}$$

where A is the initial value, and B represents the fraction of particles that appeared to be irreversibly bound to the substrate in the time frame of the experiment**.** Both A and B are fitted parameters.

Single-particle tracking of virus particles

To image and track landing HPV16, diluted HPV16 particles were added at concentration of 1.14 pM (or 0.0228 μg/mL) on the GAG surfaces. Timelapses of the landing particles were recorded at room temperature without rinsing and immediately after virus addition to capture the possible motion of the particle while still in the process of establishing bonds. Movies were recorded either at an acquisition rate of 1 s per frame for a total duration of 20 min, or at 10 s per frame for a total duration of 25 min. Recorded movies were processed to remove spatial drift and analyzed using Trackmate[36] and MATLAB DC-MSS (Divide-and-Conquer Moment Scaling Spectrum)[37] transient diffusion analysis as described in Abidine et al., 2022.[38] First, sample drift was removed using an in-house MATLAB script that performs fast Fourier transform-based frame registration[39] and generates drift-corrected movies. The resulting movies were then analyzed using TrackMate. The virus particles were detected with sub-pixel localization in each frame, and tracks were reconstructed using a maximum frame-to-frame displacement of 0.8 μm and maximum gap of 0.5 μm and 3 frames. The resulting tracks were further processed in MATLAB to correct for residual sample drift by identifying and removing collective motions common to all tracks, discarding trajectories that switched between two particles, and filtering out single-point outliers within individual tracks.

To have a better understanding of the transient diffusion of HPV16 on GAG surfaces, the trajectories were further segmented and classified in MATLAB according to the DC-MSS algorithm, as detailed in Abidine et al., 2022.[38] In brief, for each trajectory, a rolling-window of 21 frames was used to calculate the time-dependent moment scaling spectrum (MSS) and tracks were segmented according to the MSS slope: a slope <0 represented immobile particles, while a slope between 0 and 0.5 implied a confined motion, a slope of 0.5 indicating free diffusion, and a slope >0.5 directed motion. After the first classification, adjecent segments with the same motion type were pooled together, and classification was calculated once more on the new segments. Finally, diffusion properties, such as the diffusion coefficient (D) and confinement radius (R_c), were calculated for each segment, as well as the time spent in one motion type. The signal to noise ratio (SNR) in all movies was calculated as follows. First, the background was isolated from the particle signal by manual thresholding in each frame. The resulting mask was then eroded by three pixels to remove possible remaining signal from the particles, and the background mean intensity (I_{bkg}) and standard deviation (σ_{bkg}) were calculated for each frame and averaged over the whole movie. The particle intensity (I_p) was calculated by applying a Gaussian blur ($\sigma_{\text{Gaussian}} = 1$ pixel) to each frame and measuring the maximum value in a 5×5 pixel area around the detected position of each particle at each frame. The intensity was then averaged for each particle over the whole trajectory. The SNR was calculated for each particle as $SNR = \left(I_p - I_{\text{bkg}}\right)/\sigma_{\text{bkg}}$.

Estimation of the maximum number of bonds per virion

In our platform, the concentration of heparin on the surface is maintained at $\sigma = 1.1$ to 1.6×10^{11} molecules/cm^2 for all heparin species (Table 1) and is ~24 times lower than saturated coverage for experimental conditions tested here (Table S3). For this reason, heparin does not form a continuous layer; instead the chains are distributed randomly with an average distance of ~27 nm. This results in areas of high concentration and locally depleted areas. The number of molecules available for binding for a single virion thus varies depending on the landing position. The probability distribution of the number n of available heparin chain in a contact area, A, follows a Poisson distribution:

$$P(n, \sigma A) = \frac{(\sigma A)^n e^{-\sigma A}}{n!} \tag{3}$$

In particular, the probability that the area contains at most n_{max} molecules is

$$P(n \leq n_{\max}) = \sum_{i=0}^{n_{\max}} P(i, \lambda A) \tag{4}$$

The contact area depends on the radius of the virion, but also on the height of heparin chain above the PEG layer and the length of the minimal binding motif, which, based on previous studies,[29,40] we consider to be four disaccharides (Figure S7A). The Flory theory for real polymer chains was used to calculate the extension of heparin and PEG. The following parameters were used for heparin: Kuhn length (b): 9 nm, diameter exclusion volume (d): 0.5 nm, contour length (L_c): 25 nm, and $\sigma = 1.1$–16×10^{-3} nm^{-2}. For PEG, b = 1.4 nm, d: 0.3, $L_c = 23$ nm, and $\sigma = 0.07$ nm^{-2}. A detailed description of the model and calculation performed is presented in Section S11 of the supplemental information. The resulting heparin height was calculated to be between 15.1 and 16.3 nm, depending on the sulfation. Approximating the virion to a sphere of radius 25 nm, this results in an interaction area between 1,650 nm^2 and 1,730 nm^2, respectively.

Multivalency-aided diffusion simulations

We modeled the diffusion of particles on surface-grafted binders originating from the establishment of transient multivalent bonds using in-house MATLAB scripts. We consider randomly distributed binders with a defined concentration and initialize the system with a single particle bound to a single binder on the surface. The particle is then allowed to create new bonds with any of the binders within its interaction area and break existing ones with probability $p_r = 1 - e^{-r/dt}$, where r is either the attachment or detachment rates (r_{on} and r_{off}). The position of the particle is determined at each iteration as the center of mass of all the engaged binders. If the particle has no bonds with the surface, it is interpreted as detached from the surface, and the simulation is ended. We normalized the binder concentration to the number of binders in the interaction area and the r_{on} to the r_{off}, in order to generalize the results to all particle-surface interactions. We considered 35 logarithmically distributed values of $r_{\text{on}}/r_{\text{off}}$ between 0.02 and 10,000 and 25 binder concentrations between 0.1 and 100. All conditions were simulated 100 times for 500 timesteps, with a time interval $dt = 0.1/r_{\text{off}}$. Example tracks are shown in Figures S15C–S15F. We analyze the resulting tracks by considering the total path length, maximum displacement, average number of bonds, average track duration, average particle speed, hull area and radius of the trajectory, and estimated diffusion coefficient. Additionally, we segmented the tracks in 4 possible motion types (immobile, confined, free diffusion, and direct motion) as described for the analysis of the experimental tracks and shown in Figure S15A. The average motion time was determined by assigning a score from 0 (immobile) to 3 (directed) to each motion time and averaging the score over the track, weighted by the time spent in each mode, as described in Becker et al.[41] Additional details on the track analysis and the incorporation of the particle Brownian motion are described in Section S15 of the supplemental information.

RESULTS

Heparin and its derivatives have the same grafting density on the glycocalyx mimic platform

In this study, we implemented a glycocalyx mimic platform, which allowed us to study the interactions between viruses and GAGs in a well-controlled experimental environment. This platform is based on the immobilization of GAG chains by end-grafting through a biotin-tag at their reducing end, as illustrated in Figure 1A. Moreover, streptavidin was crosslinked using paraformaldehyde. This prevented GAG chain diffusion laterally on the bilayer (Figure S1; Section S3 in supplemental information) while preserving streptavidin's ability to bind biotinylated GAGs (Figure S2; Section S4 in supplemental information).

In this study, we use heparin as a representative of the sulfated domain of HS, as it is commonly used instead of HS. Moreover, sulfation patterns of heparin are more predictable and lack the highly sulfated or nearly desulfated macrodomains characteristic of HS. This reduced structural heterogeneity, along with ease of production and high purity,[42] facilitates clearer interpretation of the role of individual sulfation types, which is the focus of this work. More specifically, we used parental heparin as well as its chemically desulfated derivatives missing either 2-O, 6-O, or N-sulfations (2-O-deS, 6-O-deS, and N-deS, respectively). HA, a naturally occurring negatively charged nonsulfated GAG, was used as a negative control (Table S1). Where required, we used PEG as an inert spacer to control the GAG chains' grafting coverage.

SPR was used to follow the step-by-step assembly of GAG films and to quantify the amount of surface-bound GAG molecules on the streptavidin monolayers. A representative

time-resolved SPR sensogram for a heparin film is shown in Figure 1B, while Table S3 provides the shift in SPR angle at saturated binding for all 5 GAG films, together with the corresponding calculated grafting density and average spacing between adjacent GAGs. Further details on the calculation are provided in the supplemental information (Section S5). These data indicate that heparin and all its derivatives yield similar SCs on the streptavidin layer.

For TIRF experiments in this study, we used a mixture of GAGs and PEG at molar ratio of 1:1 or 1:5 to alter the grafting coverage of the GAG chains. However, the presence of two biotinylated species complicates the SPR analysis as only the total adsorbed mass is detected, not the relative amounts. We estimate the reduction in GAG density from the saturation values by measuring the fluorescent intensity of fluorescently labeled N-deS heparin immobilized on SLBs (FluoN-deS, Figure 1C; S3B; Section S6 of supplemental information). We determined a reduction of 6 ± 1 and 24 ± 3 for 1:1 and 1:5 FluoN-deS:PEG molar ratio, respectively. The estimated mass density, grafting coverage, and spacing between chains for all heparin derivatives and GAG:PEG mixtures employed in this work are summarized in Table 1. Overall, we observed a similar reduction by immunostaining of immobilized parental heparin (Figure S4), indicating that the grafting coverage is independent of the heparin sulfation.

N-sulfation of heparin primarily mediates the attachment of HPV16 particles to the glycocalyx mimic

To assess the influence of the different sulfate groups on the overall interaction kinetics of individual HPV16 particles with biomimetic heparin films, we used TIRF-based SPT and EFA.[8,9,43,44] Here, time-lapse movies of fluorescently labeled HPV16 binding to and detaching from the model GAG surfaces are recorded, as schematically shown in

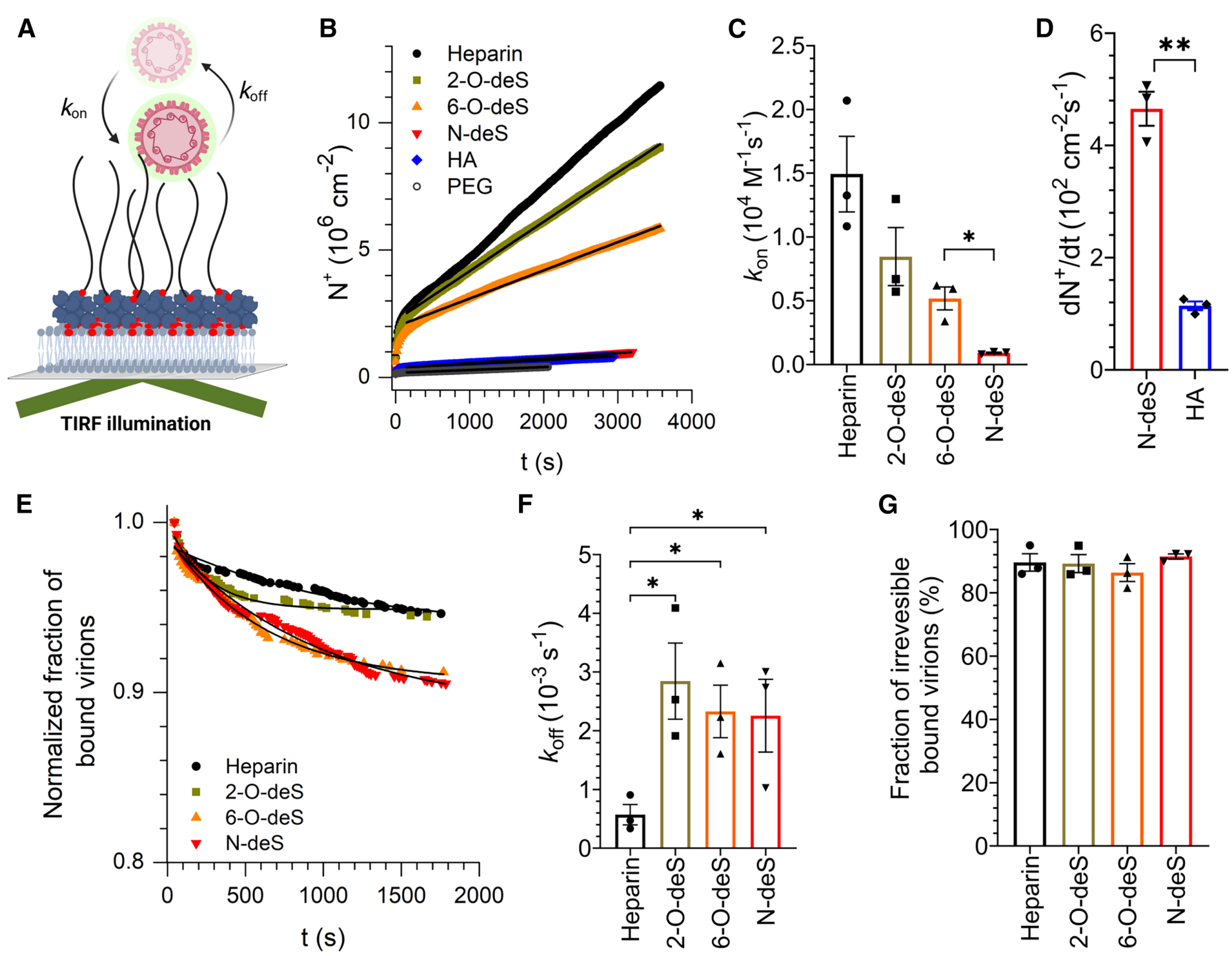

Figure 2. Kinetics of binding between HPV16 particles and GAG. (A) Schematic representation of TIRF microscopy assay to probe the interactions between HPV16 on immobilized GAG:PEG (1:5) surface. Surfaces consisting of a mixture of GAG and PEG were prepared on an SLB-bound streptavidin layer. The binding of HPV16 particles labeled with Alexa Fluor 488 was detected using TIRF microscopy. (B) Representative curves showing the cumulated number of newly bound (since t = 0) particles per unit area (N^+) as a function of time (t). The black lines represent fits to a linear function, where the first 150 s are discarded from the fit. (C) Association rate constant (k_{on}). (D) New particle arrival rate (N^+/dt) for N-deS and HA showing significant increase in binding over HA control. (E) Fraction of HPV16 remaining bound as a function of time. The black lines are best fits to a single exponential decaying function with an offset. The curves are normalized to 1 at t = 45 s (F and G) Dissociation rate constants (k_{off},F) and fraction of irreversibly bound particles (G), obtained from the decaying function fit of the data in (C) for each condition. Column bars represent means, and error bars represent SEM. Each black symbol represents an independent experiment. Statistical significance is calculated using Welch's *t* test. * = 0.02, ** = 0.006, ns = nonsignificant.

Figure 2A. The selected experimental time window for the EFA experiment allowed the observation of particles with a residence time ranging from 45 s to 30 min, i.e., in the order of the expected timescale of the HPV16-HS interaction at the cell surface during binding and the initial stages of structural activation.[31,32] Under steady-state conditions, i.e., in absence of global diffusion limitations, the arrival rate of new particles is proportional to the association rate constant of the virus particle to the GAG surface (k_{on}) (Equation 1). Additionally, the residence time of the viruses on the surface yields the dissociation rate constant of the particle (k_{off}) (Equation 2). The model GAG surfaces used for EFA were prepared with a mixture of GAG:PEG chains at a molar ratio of 1:5 as described earlier. In these conditions, the distance between two adjacent GAG chains was estimated to be between 25 and 30 nm (Table 1). This distance is about half the diameter of a single virus (~50 nm), which allows, in principle, the formation of multivalent bonds with neighboring GAG chains (see supplemental information, Section S11).

Figure 2B shows the cumulative number of newly bound HPV16 particles per surface area (N^+) over time on four differently sulfated HSs and the two negative control model surfaces (HA and PEG), while Figure S5A and Table 2 present the values of the slope obtained by linear fit. The linear increment of N^+ versus time (Figure 2B) and the limited time-dependence of the virus SC (Figure S5B) are consistent with equilibrium conditions. It must be noted that in the case where the ligand of interest is immobilized on a larger particle, as in the case of the binding of HPV16 capsomers to heparin, the slow diffusion of the whole particle might limit the arrival rate of the ligand to the cell-surface receptor. In this case, even though an equilibrium without net mass transfer is established, the k_{on} of the ligand-receptor interaction is not experimentally probed. Here, it is thus important to compare the experimental arrival rate to what is expected by sole diffusion of the particles in solution to ascertain that we are indeed probing the characteristics of the capsomer-heparin interaction. In our case, we calculated that the arrival rate in all conditions is at least one order of magnitude lower than was expected by diffusion (supplemental information, Section S7), confirming that the experiment probes the HPV16-GAG bonds in reaction limit conditions.

Table 2. Summary of association rates and association rates per GAG chain on GAG:PEG model surfaces

GAG surfaces	Arrival rate (N^+) (10^{15} cm^{-2}M^{-1}s^{-1})	k_{on} (10^4 M^{-1}s^{-1})	k_{off} (10^{-3} s^{-1})
Heparin:PEG	1.67 ± 0.60	1.49 ± 0.5	0.57 ± 0.3
2-O-deS:PEG	1.10 ± 0.51	0.85 ± 0.4	2.8 ± 1.1
6-O-deS:PEG	0.79 ± 0.24	0.52 ± 0.15	2.3 ± 0.77
N-deS:PEG[a]	0.14 ± 0.02	0.09 ± 0.01	2.3 ± 1.1
HA:PEG[a]	0.035 ± 0.004	/	/
PEG	0.07 ± 0.02	/	/

Arrival rate; k_{on}= association rate constant, assuming that each GAG chain is an individual receptor; and k_{off} = dissociation rate constant.
[a]At 3 times more virus concentration in bulk solution.

The notably low arrival rate of particles to N-deS indicates that HPV16 mostly exploits N-sulfation to bind to heparin, although it appears that HPV16 is still able, to a limited extent, to interact with N-deS heparin (Figure 2D). This interaction is presumably mediated by other sulfate groups, or by the ~8% residual N-sulfates, which are still present after the chemical de-sulfation process (see materials and methods and Section S16 of supplemental information for a discussion on the potential effect of residual sulfation on particle binding).

Under steady-state conditions, and when the number of occupied receptors is low, the arrival rate is proportional to k_{on} and to the number of receptors found on the cell surface (Equation 1). In the case of a virus binding to heparin, the estimation of k_{on} is complicated by the fact that the interaction is multivalent and that the receptor is not unambiguously identified. Since it has previously been reported that at least four disaccharide units are needed to establish binding,[32,40] one GAG chain may in principle carry several virus-binding domains. Nevertheless, we estimate the association rate constant for the virus-GAG interaction (k_{on}) from the measured arrival rate (Figure S5A), assuming that each GAG chain is acting as a single receptor (Figure 2C; Table 2). Our results show that removal of N-sulfates leads to a decrease in k_{on}, by a factor ~16 as compared with heparin. Removal of 6-O sulfates leads to a decrease in k_{on}, by a factor ~2, while removal of the 2-O sulfates leads to a decrease in k_{on}, by a factor ~1.5 as compared with heparin. Overall, these suggest that HPV16 favors the binding to sulfated GAGs, and that N-sulfates, in particular, play a key role in mediating a stable attachment of the virus to cell-surface HS, in agreement with our previous studies.[23,30] This clear dependence of HPV16 binding on sulfate groups is also supported by data showing the surface coverage of particles bound to the respective surfaces (Figures S5C and S5D).

To gain insight into how the different sulfation groups influence the overall multivalent dissociation behavior of a single particle, we consider the residence time of individual HPV16 particles and report the fraction of particles still bound over time (Figure 2E). All plots showed a slow dissociation. Fitting the dissociation data with a single exponential decay with offset function (Equation 2; black solid lines in Figure 2E), as an approximation for the dissociation behavior, revealed that about 90% of the HPV16 particles were irreversibly bound (see materials and methods) in all cases (Figure 2G), meaning that they do not dissociate from the surface within the time frame of the experiment (30 min). For those particles that exhibited dissociation, we extracted the dissociation rate constants for the multivalent virus-GAG interaction (k_{off}) from the fits. As presented in Figure 2F, no statistically significant differences in k_{off} values were found for the different desulfated heparins; however, parental heparin showed a significant lower k_{off} value in comparison to other desulfated heparin, indicating that all sulfates have a stabilizing effect on heparin binding. Comparing these results with our previous reports on monovalent capsomer-heparin bonds using AFM-SMFS,[30] we observe much larger differences in k_{on} between heparin derivatives (a

factor >15 instead of two to three for single bonds), while we fail to observe significant differences in the detachment rate, which instead spanned more than two orders of magnitude in the single-molecule case (see Table S4; Figure S6).

Multivalent interactions stabilize the virus-receptor interaction: A theoretical framework

To investigate the origin of the dynamic behavior of individual particles establishing multiple interactions with a receptor-presenting surface, we propose a simplified model as a theoretical framework to relate the single-molecule and multivalent interaction kinetics. In this model, we assume that each heparin chain acts as a single binding site, i.e., a single chain can only bind one capsomer of the HPV16 particle. Similarly, an individual capsomer can only interact with a single heparin chain. Thus, the multivalent interaction between a particle and the substrate requires the binding of multiple heparin chains by an equal number of capsomers on the virus capsid (see Figure S7A). This assumption is reasonable, given that in the AFM-SMFS experiments published previously,[30] the reported interactions were ascribed to a single HPV16 particle interacting with a single heparin chain, without observing, in most cases, multiple rupture events. Since we focus our interest on the binding behavior at the particle level, we abstract from the nature of the molecular interaction and refer to each interaction between a capsomer and a heparin chain as a single bond. Further, we consider that all heparin chains immobilized on the biomimetic surface are always accessible for binding. This aligns with the notion that the heparin chain moves much faster (~100 ns) than the timescale for detachment, so the binding sites are accessible during the capsomer-heparin interaction (see supplemental information, Section S11) In this scenario, the number of possible bonds, i.e., the number of possible capsomer-heparin interactions, a single particle can form with the surface is thus limited by the number of heparin chains in its vicinity or the number of available capsomers. In the model, we further assume that all bonds are identical, that the individual bonds do not interact with each other, and that they can be independently formed and broken; i.e., they should be modeled as independent contributors rather than cooperatively producing a single energy barrier between the bound and unbound states. In such a case, the bonds are formed one at a time in a sequential manner. Consequentially, when a particle first attaches to the surface, only one bond is formed. Under such conditions, the interaction can be modeled as a continuous-time Markov process where states are solely defined by the number of bonds, and we only consider transitions between states that differ by a single bond, as depicted in Figure 3A. The association rate (r_{on}), the rate at which a new single bond is created, can be readily calculated from the average binding time τ^{SB} previously measured for AFM-SMFS (Table S4). In the case of a particle bound to the surface, the interaction volume can be estimated as a cylinder having the height of the expected elongation of the grafted heparin during the particle residence time (h ~16 nm, Table S5) and the radius of the base surface of a spherical cap of height h and radius of a virus particle ($R \sim 25$ nm), $V_{int} = \pi h^2(2R - h) = 28 \times 10^3$ nm^3 (Figure S7A and supplemental information; Section S11). Note that in this case, the particle is not allowed to rotate freely, and the interaction volume only includes ~23 capsomers (n_c) on average. This contrasts to the AFM-SMFS case, where the particle is held in contact with the surface by the cantilever tip, and the estimated interaction volume includes the whole virus particle, with any of the 72 capsomers (N_c) available for binding. To account for differences in number capsomers available for binding in the different experimental geometries, we adjusted the association behavior for the heparin derivatives as $r_{\text{on}} = n_c/N_c\tau^{\text{SB}} = 2.6$ to 8.5 s^{-1} (Table S4). In addition, the association rate is proportional to the number of heparin chains available to the virus. For this reason, the transition between a state with M bonds to one with M + 1 bonds can be calculated as $r_{\text{on}}^{M\to M+1} = (M_{max} - M)r_{\text{on}}$, where M_{max} is the maximum number of bonds that can be formed by a particle. In our experiments, given the heparin low grafting density, this corresponds to the number of heparin chains in the interaction volume (Figure S7).

As for the dissociation behavior, the transition rate between a state with M bonds and one with M − 1 bonds is proportional to M, since there are M bonds available for dissociation, and to the single-molecule dissociation rate ($k_{\text{off}}^{\text{SB}} = r_{\text{off}} = 4 \times 10^{-3}$ to 12 s^{-1}) determined by AFM-SMFS (Table S4). Thus, $r_{\text{off}}^{M\to M-1} = Mr_{\text{off}}$. The rate of the possible transitions in the model system can be described with the following matrix:

$$Q = \begin{array}{c} \\ \\ \\ \\ \\ \end{array}\overset{\begin{matrix}0 & \quad 1 & \quad\quad 2 & \quad\cdots & M+1\end{matrix}}{\begin{bmatrix} 0 & 0 & 0 & \cdots & 0 \\ r_{\text{off}} & -\left(r_{\text{off}} + (M-1)r_{\text{on}}\right) & (M-1)r_{\text{on}} & \cdots & 0 \\ 0 & 2r_{\text{off}} & -\left(2r_{\text{off}} + (M-2)r_{\text{on}}\right) & \cdots & \vdots \\ \vdots & \vdots & \vdots & \ddots & r_{\text{on}} \\ 0 & 0 & \cdots & Mr_{\text{off}} & -Mr_{\text{off}} \end{bmatrix}}\begin{matrix}0 \\ 1 \\ \vdots \\ M \\ M+1\end{matrix} \tag{5}$$

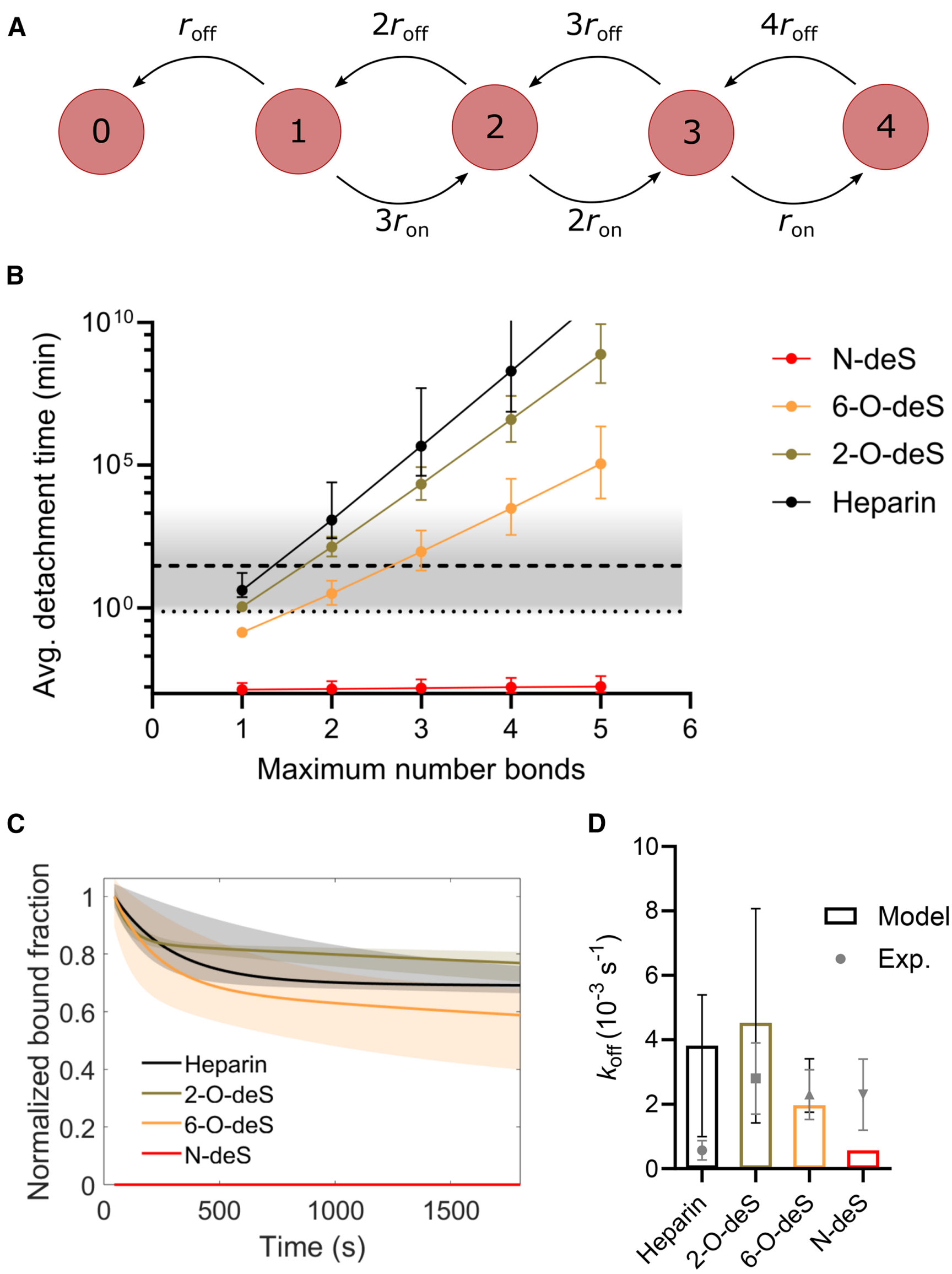

Figure 3. Markov theory to estimate the average dissociation rate. (A) Diagram of the continuous-time Markov chain that describes the multivalent binding of a particle considering possible creation of new bonds, at the example of a case with a maximum number of bonds of 4. The states are named after the number of bonds established with the surface, and the arrows indicate the possible transitions with their expected rate. (B) Average detachment time for HPV16 as a function of the maximum number of bonds they can establish with the surface-immobilized heparin. The dotted line indicates the minimum time a particle must stay on the surface to be detected as bound, and the dashed line indicates the duration of an experiment. The error bars indicate the values obtained using the confidence interval obtained in the AFM-SMFS measurements. The area between the two lines is the time frame in which our system can detect particle detachment, while the shaded gray area indicates the timescale of HPV16 internalization from first structural modifications upon HS binding (∼minutes) to entry (4–16 h). (C) Curves obtained by calculating the percentage of bonds remaining as a function of time using the values in (B) and the heparin grafting density. The solid line was obtained using the mean value and the shaded areas using the upper and lower limits of the bond lifetime. (D) Dissociation rate constants obtained by fitting the curves in (C) with the same function used for the experimental data. Error bars indicate the values obtained using the curves describing the edges of the shaded areas in (C). The gray symbols indicate the experimental results.

From this matrix, we can use Markov theory to estimate the average time needed to transition between the different states, as further detailed in the supplemental information (Section S10). In particular, we can calculate the time between attachment (1 bond) to particle release (0 bonds), using the following formula:

$$t_{10} = -\frac{1}{q_{11}} + \sum_{j \neq 0} \frac{q_{1j}}{q_{11}} t_{j0} \quad (6)$$

where q_{ij} is the (i,j) element of the Q-matrix in Equation 5, and t_{j0} is the average time for the particle to transition from a state with j bonds to release.

In our AFM-SMFS experiments, the attachment rate was found to be between 1 and 3 orders of magnitude faster than the detachment rate for all heparin variants but N-deS[30] (Figure S6). This means that after attaching, the particles will quickly occupy all the available binding sites and that all bonds must break almost simultaneously to prevent reattachment. The residence time of the particle thus strongly depends on the number of available binding sites for all heparin variants, as shown in Figure 3B. For parental and 2-O-deS heparin, the high affinity between heparin and HPV16 results in dissociation times for the particle longer than the duration of the experiment (dashed line in Figure 3B), suggesting that the detachment observed in the TIRF-based kinetic experiment is due to particles that only formed a single bond to the surface. In the case of 6-O-deS, instead, the average lifetime of a single bond is less than 10 s. This is more than 4 times shorter than the minimum residence time used in our experiment to define a bound particle (3 consecutive frames at 15 s per frame: 45 s, dotted line in

Figure 3B) and indicates that particles bound through single bonds are typically not captured in our experimental setup. In contrast, particles interacting with two bonds exhibit an average residence time of ∼200 s compatible with experimental parameters and similar to what was observed for single bonds of parental and 2-O-deS heparin. This multivalent effect can explain the similar dissociation kinetics observed in TIRF experiments and provides an example of the stabilizing effect of multivalency in HPV16-cell interaction.

Combining the bond lifetime as a function of the number of bonds formed with the probability that a particle lands in a region where multiple bonds are possible, we calculated the survival probability of particles at the surface as a function of time and the dissociation rates predicted by the model (Figures 3C and 3D). The resulting curves qualitatively resemble those observed experimentally (Figure 2E), and the dissociation rate constants show good quantitative agreement with the experiments for both 2-O-deS and 6-O-deS. The model overestimates the dissociation rate for heparin, although the predicted value remains within the same order of magnitude as the experimental measurement. In addition, the predicted presence of a fast-dissociating population for 2-O-deS and 6-O-deS was tested by EFA at a higher time resolution of 1 fps, with the observation period limited to 15 min. As shown in Figure S9A, the experimental data are in very good agreement with the model predictions, with dissociation times comparable to those expected for the detachment of single bonds. However, the presence of short-lived, possibly nonspecific events limits the accuracy of the measurement (Figures S9B and S9C).

In the case of N-deS, much faster dissociation ($r_{on}/r_{off} \sim 0.13$) causes the lifetime of all particles, even those bound via multiple bonds, to be much shorter than what can be detected in our experiment. Indeed, the average lifetime of a particle bound through 5 attachment points is only ∼0.1 s, indicating that in our TIRF-based kinetic experiments, no particle binding should be detected. We speculate that the low, yet specific residual binding to N-deS (binding is still significantly higher than what observed for the non-sulfated HA) is due to residual N-sulfation in our sample, in which sulfation is reduced by 92% and not eliminated, as further discussed in Section S16 of the supplemental information.

In all cases, the model underestimates the irreversible fraction, i.e., the number of particles that remain on the surface for longer than the experimental observation time. The number of irreversibly bound particles is primarily determined by the number of available binding partners, as bond multiplicity rapidly stabilizes the interaction, as shown in Figure S10. We therefore consider the possibility that the model underestimates either the interaction area between the particles and GAGs or the GAG grafting density in the GAG:PEG complex mixture. Doubling the grafting density in the model indeed greatly improves the agreement between the model and the experimental data (Figure S10B).

Diffusion of HPV16 on heparin surfaces is restricted

Diffusion of individual virus particles across GAG chains through exchange of individual ligand-receptor interactions[9] may be an important mechanism by which viruses can move through the glycocalyx and on the cell surface, thereby playing an important role in virus accessibility to the cell surface for entry.[6] Next, we thus asked whether the presence or absence of sulfate groups could influence the lateral diffusion of the HPV16 on the GAG layers, and in particular whether areas of low N-sulfation in the glycocalyx, while being ineffective at structurally activate the virus, could allow it to search the glycocalyx for areas of higher affinity, while maintaining contact with the cell surface. To address this question, we characterized the mobility of individual HPV16 particles using SPT on parental heparin and N-deS derivatives, the heparin derivatives that exhibit the highest and lowest affinity, respectively[30] (Figure 2). Time-lapse movies were recorded immediately after adding fluorescently labeled HPV16 to the GAG surfaces, and single-particle tracks were extracted from the resulting movies[36] (Figure 4A). The influence of the GAG concentration on the diffusive behavior was first assessed by measuring the diffusion of HPV16 particles on surfaces prepared at saturation coverage with pure GAGs as well as surfaces prepared from a mixture of GAG:PEG chains at molar ratios of 1:1 and 1:5. For these conditions, the spacing between individual GAG chains was estimated to be 5.1 ± 0.6, 13 ± 1, and 27 ± 2 nm, respectively. We analyzed the collected tracks using a segmentation algorithm[41] to distinguish free diffusion from confined or immobile particles. In all conditions, we observed limited mobility with particles spending more than 99% of the total time tightly confined within an area comparable to a single pixel (Figures 4A and 4C and S12). The small differences in the values of the confinement radius (Figure 4B) and diffusion coefficient (Figure S13) observed between experimental conditions were found to be mostly a consequence of tracking noise, as shown by experiments tracking particles stuck on glass, and assumed to be immobile, as well as by simulations of immobile particles at SNRs comparable to the range observed in the experiments (see supplemental information, Sections S12 and S13). These controls confirm that, in our experiments, particle motion mostly falls below the resolution of our tracking techniques and is to be considered largely immobile. Similar results with low mobility were obtained with the other desulfated derivatives (data not shown).

In the absence of PEG dilution, i.e., in case of a saturated N-deS surface, we observed a notable increase in particles undergoing free diffusion compared with all other conditions with instances of free diffusion lasting for more than 10 min (Figure 4A). However, free diffusing events remain below 1% of the total recoded track duration (Figure 4C) and are too rare to confidently measure the diffusion coefficient or other mobility parameters. No free motion was

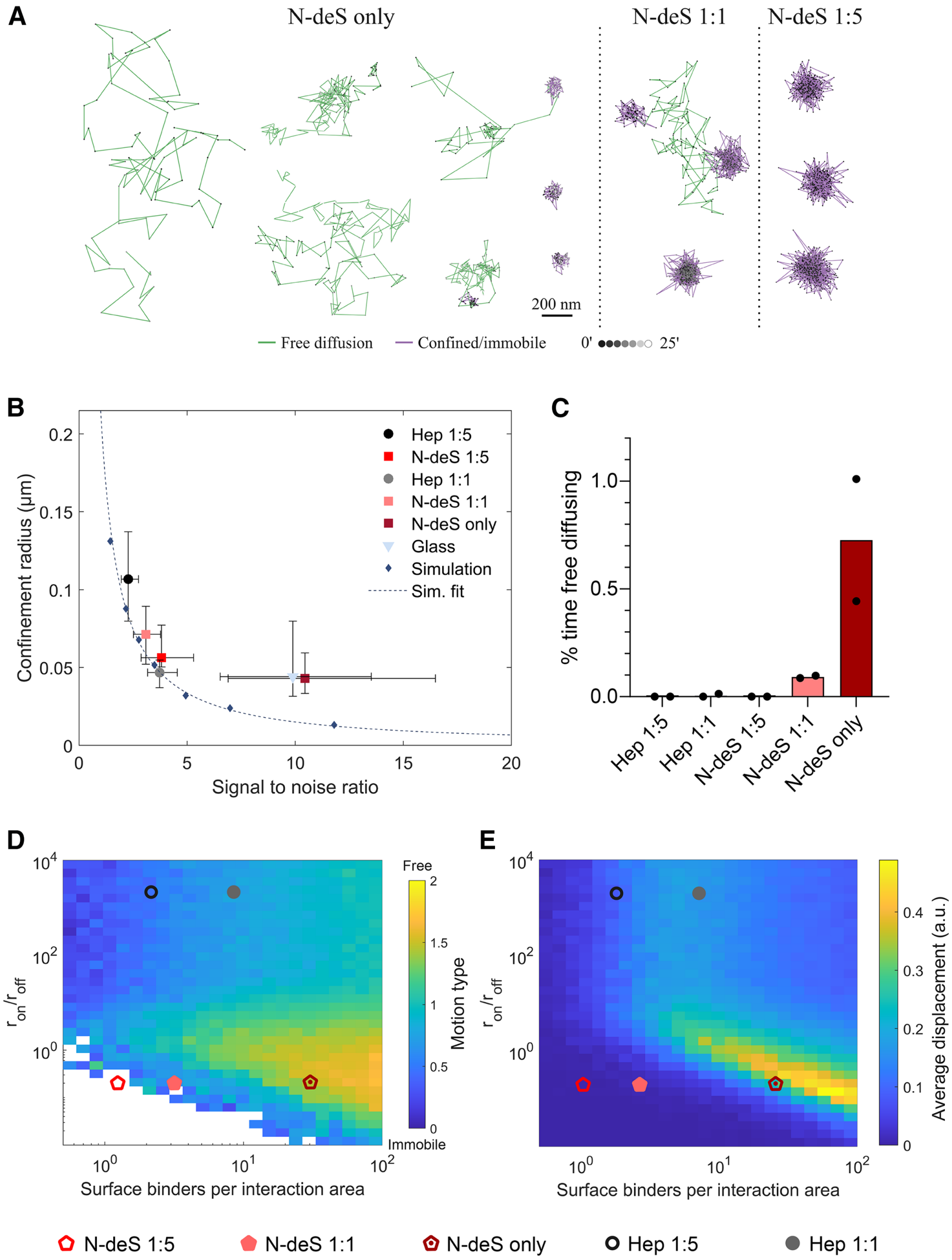

Figure 4. HPV16 diffusion on the GAG surface. (A) Example of experimental tracks of HPV16 particles diffusing on surfaces presenting different grafting coverage of N-deS heparin; from left to right: N-deS only: $\sim 38 \times 10^{11}$ cm^{-2}; N-deS:PEG 1:1: $\sim 6 \times 10^{11}$ cm^{-2}; N-deS:PEG 1:5: $\sim 1.6 \times 10^{11}$ cm^{-2} (Table 1). Tracks are colored to distinguish between segments displaying free diffusion (green) and tightly confined/immobile segments (purple). Grayscale dots indicate the position of the particle at each frame and are colored to indicate the passage of time from black (0 min) to white (25 min). (B) Confinement radius of the detected particles as a function of the signal to noise ratio of the images used for single-particle tracking. The filled dots indicate the median value for each experimental condition (black: Hep:PEG1:5; gray: Hep:PEG1:1; red: N-deS:PEG1:5; pink: N-deS:PEG1:1; dark red: N-deS). Particles stuck on glass, in teal, were an immobile control. The error bars indicate the first and last quartile. The blue diamonds show the confinement radius obtained using our algorithm for synthetic timelapses of immobile particles. The dashed line is the best power fit: $R_C = 0.215 \cdot \mathrm{SNR}^{-1.15}$. (C) Percentage of time collectively spent by the HPV16 particles in free diffusion for parental heparin and N-deS heparin at different grafting densities. (D and E) Results of our multivalency-aided diffusion model of viruses on immobile randomly distributed surface binders. The heat maps, show (D) the average motion type (0: immobile, 1: confined, 2: free motion; see Becker et al.[41]) and (E) the average confinement radius as a function of the ratio between attachment and detachment rates (r_{on}/r_{off}) and the number of binders available to the virus on average estimated through our simulations. White area indicates fewer than 10 tracks available for analysis. The circles and pentamers represent the various experimental conditions investigated in this work for N-deS and parental heparin, as indicated in the legend.

observed for saturated parental heparin, probably due to the extremely stable bond established by HPV16, which prevents mobility.

In summary, HPV16 particles have limited capability to diffuse on heparin, even when most of the N-sulfates are removed, despite the profound effect of this modification on the association rate of the virus particle and on the dissociation rate constants of the single virus-heparin interactions.[30]

Model of multivalency-aided diffusion

To further complement our experimental tracking results, we explored the condition needed for multivalency-assisted diffusion of the particle on the surface, i.e., motion of the particles on the surface presenting only immobile receptors, due to the creation and rupturing of bonds. We modeled the system considering that heparin chains are randomly distributed on the surface and immobile. In this simulation, the virus particle is initially bound to a single heparin chain and allowed to interact with neighboring chains within its interaction area. Upon the formation of new bonds, the particle moves to the center of mass of the bound chains, resulting in a particle displacement on the surface (see supplemental information, Section S15). We initially consider the bond as rigid and neglect the Brownian motion of the tethered particle. Since the motion of the particle only depends on how fast bonds are created and broken and on the number of binders available for binding, we generalize our model by normalizing for the ratio of the attachment and detachment rates (r_{on}/r_{off}) and the number of chains within the interaction volume. In this case, time is also

expressed as a function of the detachment rate, while space coordinates are rescaled into interaction area (A_i) units, i.e., $t = 1/r_{\text{off}}$ and $x = 1/A_i^2$.

We varied the ratio between the attachment and detachment rate ($r_{\text{on}}/r_{\text{off}}$) from 0.01 to 1,000 and the binder number per unit area between 0.5, for which the binding sites are so sparse that almost completely prevent multivalency, and 100 binders. The results of the simulations are shown as heat maps in Figures 4C and 4D, as well as in Figures S13 and S14. We observe the largest mobility in the presence of multiple weak bonds (bottom right corner in Figures 4C, 4D; and S13B), both in the type of motion (free diffusion versus confined or immobile particles), diffusion coefficient, and the area explored by the particle. This is expected as weak bonds allow the particle to move on the surface, while a large number of binders prevents detachment from the surface (Figure S15D). Strong bonds at low binder density (1–10 binders) results in long tracks and high average speeds, comparable to the high-multiplicity, low-affinity case; however, in this condition, the particle motion is strongly confined in a small region due to the inability to efficiently break already established bonds (Figure S15C). Mobility becomes extremely limited and largely insensitive to the bond affinity for $r_{\text{on}}/r_{\text{off}}$ larger than 10. This is due to the high binding affinity between individual ligand receptors, which encourages rebinding in case of bond rupture, effectively resulting in particle immobilization at the landing site (Figure S16).

The model does not make assumption on the particle size or absolute value of the binding and unbinding rates and can thus be easily applied to other systems and experimental conditions. However, it does not consider the Brownian motion of the particles tethered to the surface. For this reason, we tested if the addition of Brownian motion has a significant impact on the overall particle diffusion. Given that Brownian motion is much faster than the binding kinetics (ns to μs versus ms to minutes), we can assume that a particle tethered to the surface by one or multiple heparin chains will explore the space around the binding site. Effectively, the particle Brownian motion increases the interaction area beyond the particle radius. The average displacement of a particle is dependent on the number of tethering heparin chains, and it was estimated through simulations, as further detailed in the supplemental information, Section S15. This displacement was then added to the interaction radius to correct the interaction area used in the multivalency-aided diffusion simulation. The larger interaction area and thus availability of possible receptors results in an increased mobility at low binder concentration (Figure S19). This inclusion, however, did not modify the overall dependency of mobility on $r_{\text{on}}/r_{\text{off}}$ or receptor density, and, thus, any major conclusion is from the simpler, Brownian-free, model.

Our model indicates that surface diffusion on immobilized binders driven solely by the binding and unbinding of individual bonds in a multivalent interaction is only expected for low-affinity bonds, r_{on} of similar magnitude to r_{off}, at high binder concentrations (bottom right corner in Figures 4D and 4E).

The model is in good agreement with our experimental results obtained with parental heparin. In this case, the highly stable single-molecule interaction ($r_{\text{on}}/r_{\text{off}} \sim 2 \times 10^3$) strongly restricts the mobility of particles attached to the surface through multiple bonds. This results in a strongly confined motion around the initial binding site with a confinement radius of only few nanometers (considering an $\sim$1,700 nm^2 interaction area) at any surface binder density. This motion is of the same order as the Brownian motion experienced by tethered particles and around an order of magnitude smaller than the localization precision in our experiments, explaining the absence of free diffusion observed for parental heparin. In the N-deS case, due to the lower affinity ($r_{\text{on}}/r_{\text{off}} \sim 0.13$), the model predicts a sharp change in behavior at increasing binder concentrations (red symbols in Figures 4D and 4E) from short-lived confined motion to long-range mostly free diffusion. While an increase in mobility is observed in the experiments at high binder concentrations, and free diffusion is only present at GAG interchain distance of $\sim$5.1 nm, immobile or strongly confined particles remain the dominant population in all conditions (Figure S11). We hypothesize that this discrepancy is due to the imperfect chemical removal of N-sulfation from N-deS heparin. Indeed, the presence of low percentage of N-sulfates is statistically sufficient to lead to the formation of localized binding pockets capable of stably pinning the virus to the surface (see Figure S20, Table S6, and Section 17 in the supplemental information for further details).

DISCUSSION

In this study, we adopted an approach based on a glycocalyx mimic platform to study the interaction dynamics of individual HPV16 particles binding to multiple heparin chains, using TIRF-based SPT and EFA. The role of individual sulfate types was assessed using desulfated variants of heparin. The study, which tagetted multivalent interactions at the particle level, reveals that N-sulfates are essential to ensure initial association of an HPV16 virus particle to a layer of heparin molecules, with only marginal effects from other sulfate types. Our results further show that the type of sulfation has little influence on both the dissociation of particles from and their diffusion on the biomimetic heparin-presenting surfaces.

The kinetic studies of HPV16-heparin interactions on a single-particle level presented here expand and complement our previous study of HPV16-heparin interaction using AFM-SMFS. The AFM-SMFS study focused on probing the characteristics of “monovalent” heparin-HPV16 interactions, presumably involving the interaction between an individual capsomer and an individual heparin chain. Here,

we employ TIRF microscopy and SPT to observe the binding behavior of whole virus and consider the effect of the creation of multiple bonds by the same virus.[30] Together, both studies provide a more comprehensive understanding of the role of sulfation groups in modulating the multivalent binding of HPV16 to HS. Both studies agree that N-sulfation is crucial for HPV16 binding to cell-surface GAGs. However, for the case of a particle interacting through multivalent interactions, the type of sulfation primarily influences the arrival rate and k_{on}, with a limited effect on k_{off}. This stands in contrast with the single-molecule (monovalent interactions) investigations (Table S4) that reveal only a marginal effect of the type of sulfation on the association rate constant $\left(k_{on}^{SB}\right)$ and rather point toward a key role of the N-sulfates in stabilizing the interaction by increasing the lifetime of the capsomer-heparin bond (decreasing k_{off}^{SB}). These differences reflect the formation of multiple bonds between the HPV16 particle and heparin chains while being also associated with the different experimental timescales of both techniques. Indeed, AFM is able to resolve transient and fast interactions, while TIRF-based kinetics focus on bonds lasting for minutes to hours, closer to what is observed during viral infection.[31,32] Bridging this gap thus requires adequate modeling. To address this, we employed a continuous-time Markov model, in which bonds form and dissociate independently while the particle is in contact with the surface, and where the state of the particle is fully described by the number of bonds formed. The model reveals that for parental and 2-O-deS heparin, it is only possible to monitor the detachment of particles interacting with the surface through a single bond. Particles capable of forming multiple bonds with the surface have detachment times that are orders of magnitude larger than the experimental observation time and thus appear as irreversibly bound to the surface (Figure 3B). Accordingly, the relative difference in k_{off} between heparin and 2-O-deS is in very good qualitative agreement with the values of the k_{off}^{SB} values reported by AFM-SMFS[30] (Table S4). For 6-O-deS, the model predicts that particles forming only a single bond have a residence time around 5 times shorter than the experimental time resolution, making them undetectable in our experiments and suggesting that only particles that form at least two bonds can be observed attaching in the assay. Conversely, particles forming more than 3 bonds have an expected detachment rate lower than what can effectively measure in our experiments and will appear as irreversibly bound. Thus, the slow detachment observed for 6-O-deS in TIRF-based experiments can be attributed to the stabilization effect of multivalency, which makes the detachment rate comparable to those of monovalent bonds formed by HPV16 with the parental heparin. Additionally, our model predicts that the experimental window of the TIRF-based kinetic experiments does not allow us to visualize the binding of HPV16 to N-deS heparin, which aligns with the low attachment rates reported here. A second consequence of the high affinity of the HPV16-heparin interaction, except for the N-deS case, is that the particles, once the first bond is established, quickly form additional bonds to all heparin chains accessible. Thus, the number of bonds formed to the surface depends mainly on the number of heparin chains that can be reached by an already bound particle. While the heparin grafting coverage in our system guarantees an average of ~2 heparins per particle, the random distribution of accessible surface-grafted heparin leads to areas of high heparin density and areas with one or no heparin chains available for particle binding (Figures S7B and S7C). By modeling the heparin chain as a semi-flexible polymer embedded in a PEG brush (see Section S11 in supplemental information for the full mathematical model), we determine that most particles reaching the surface (>70%) will land in vicinity of multiple heparin chains and are able to form more than one bond (Table S5). This contributes to the large portion (~90%) of irreversibly bound particles observed for all heparin variants. This modeling also reveals that ~30% of particles will form a single bond for the 6-O-deS case, which are undetectable in our TIRF-based kinetic experiments due to their short residence time (Figure 3B). We speculate that this could be the reason for a 65% ± 41% reduction in the k_{on} value observed between parental and 6-O-deS heparin (Figure 2C).

When it comes to the determination of the multivalent particle's lifetime with our Markov model, it should be noted that this value is very sensitive to the input binding rate (r_{on}) value. Indeed, r_{on} determines the rebinding probability of individual bonds while the particle is still attached to the surface, i.e., the time frame in which all bonds need to break for the particle to be released. An accurate determination of r_{on} is particularly challenging here, as it requires knowledge of the interaction volume, which is hard to predict.[45,46] For example, the extension of the heparin chains in solution, the temporal availability of binding sites, as well as the mobility of the tethered particle all influence r_{on}. Additionally, these parameters could slightly vary between the sulfation level and grafting densities of the different heparin derivatives. While such inaccuracies potentially limit our ability to resolve small differences, expected for example when comparing 2-O-desulfation to heparin, our analysis remains very helpful in the interpretation of the interaction and resolving the apparent mismatch between single and multiple bond behavior observed experimentally. Although the model reproduces our experimental data well at both short (1 fps for 15 min) and long (15 s per frame for 1 h) timescales (Figures 3C, 3D, and S9), its validity cannot be fully tested in the system investigated here. This is primarily due to the high affinity of the HPV16-heparin interaction, which remains highly stable even at a low number of bonds. For example, the model predicts an average detachment time of ~7,000 h for a triple bond, which is experimentally inaccessible and of limited

biological relevance. In addition, the strong dependence on N-sulfation, where the monovalent affinity of N-deS is more than three orders of magnitude lower than that of parental heparin, makes the system highly sensitive to residual N-sulfates in the N-deS sample. As a result, even small amounts of remaining N-sulfation can dominate binding and prevent reliable determination of multivalent kinetic parameters. Accurate validation of the model will require its application to systems with lower affinity and higher multivalency, which is beyond the scope of this work.

The kinetic investigations presented here further revealed that the k_{on} values determined in TIRF-based experiments are two orders of magnitude lower, as compared with the ones determined by AFM-SMFS, k_{on}^{SB}. This discrepancy is particularly surprising considering that, in most cases, we determined that the detachment events observed in TIRF experiments are from particles monovalently bound to the surface, and thus the same bond probed with AFM-SMFS. The determination of k_{on} via AFM-SMFS is strongly dependent on the determination of the interaction volume, which, as discussed above, is difficult to estimate as it depends on the specific configuration of the particle immobilized on the tip.[45,46] Conventionally, this is estimated as the half sphere having the sum of the particle diameter and the polymer chain linking it to the AFM tip as a radius. However, this can lead to an overestimation of the volume, particularly in the case of a short linker, such as the one used in our previous AFM-SMFS study where the linker is estimated to be $\sim$2 nm in length (unstretched). Indeed, such a short linker will most likely greatly restrict diffusion and rotational freedom to the particle. Accordingly, a contact volume between the particle and the heparin layer, as the one used in the Markov analysis (Figure S7A), may lead to a more realistic estimation. In such a more conservative representation, the interaction volume is decreased by a factor $\sim$24, with an equivalent reduction of the measured k_{on}^{SB}. This illustrates that the discrepancies in experimental k_{on} values reported for the TIRF and AFM-SMFS assays may be explained by assumptions and limitations of the used models. Altogether, the theoretical framework developed in this paper emphasizes how the time window and multiplicity of virus-receptor bonds can drastically influence their binding kinetic characteristics. For example, we show that a substantial change in bond stability (i.e., bond lifetime) can manifest as an apparent variation in association kinetics when examined at the spatial and temporal scales of the virus attachment, rather than at the level of individual bond formation.

A second aspect of this study focuses on the two-dimensional diffusive properties of heparin-bound HPV16 by tracking the movement of fluorescently labeled particles after they landed on the heparin film, using TIRF-based SPT. We previously reported that HSV1 viruses bound to GAG films similar to those used here undergo lateral diffusion once recruited to the surface, presumably through exchange of individual bonds between GAG chains and viral glycoproteins.[9] This observation inspired us to investigate whether similar behavior exists for HPV16. In this study, however, HPV16 diffusion was extremely limited. For parental heparin, only immobile particles were observed, independently of the heparin grafting density. On N-deS heparin, particle mobility was partially recovered with a small percentage of particles displaying slow but free diffusion at high N-deS grafting coverage (Figure 4). This trend agrees with the numerical model we propose to describe multivalency-assisted mobility on surface-immobilized binders. The model, however, predicts nearly free diffusion for most particles in the case of high SC of N-deS heparin. This discrepancy is probably due, as in the kinetic assay, to residual N-sulfation present in our samples. Altogether, we conclude that passive diffusion of HPV16 aided by multivalent bond is unlikely at the cell surface, due to the long bond lifetimes (small k_{off}^{SB} values) reported for the interaction with parental heparin.[30] Previous studies showed that HPV16 binds quickly and mostly irreversibly to cells via interaction with HSPGs in line with our model.[33,47] HPV16 exhibits lateral mobility on the plasma membrane predominantly by constricted diffusion and to a minor extent by ballistic motion types.[33] The relatively fast diffusive motion is likely the result of lateral diffusion of HSPGs or clusters thereof within the membrane, whereas ballistic motion is driven by actin-retrograde flow. Hence, this is in line with the conclusion of a lack of passive diffusion by binding and unbinding events. The current model of structural activation by glycan engagement indicates a preference for certain sulfation types on HS.[29,48] On cells, HS instead of heparin is present. The highly dispersed HS is variably sulfated and exhibits sulfated domains interspersed with unsulfated domains. Hence, it is possible that while not contributing to passive diffusion, binding and unbinding events of single HS chains may help sampling of the sulfated domains for a best fit to achieve structural activation. Finally, it has been reported that structural rearrangements and further enzymatic processing of the capsid weaken the affinity with cellular HS.[49–51] We hypothesized that these processes could, in a second phase, improve the local mobility of the virus at the cell surface and thereby contribute viral entry.

In conclusion, the theoretical framework presented here allows us to predict the dynamic behavior of individual virus particles interacting with multiple cellular receptors from the association and dissociation rates of the individual ligand-receptor bonds involved in the interaction. In this study, this framework was used to convey fundamental understanding of the attachment, detachment, and diffusion behavior of HPV16 particles in an HS-rich glycocalyx and of its dependence on the presence of specific sulfation types. Beyond confirming the key role of N-sulfation in mediating the interaction of HPV16 particle with HS, we were able to show that at timescales of relevance to the HPV16 entry, the attachment rate of the virus particle is primarily determined by the lifetime of the individual virus-glycan bond; the

influence of the association rate constant of the single bond (k_{on}^{SB}) becomes negligible. We further explored the possibility that areas of low sulfation and high receptor density could allow for motion of particles on the surface presenting immobile cellular receptors, due to the creation and rupturing of individual bonds with cellular carbohydrates. We concluded that while such behavior is possible in the presence of abundant and weakly interacting binders, it could not be observed experimentally for the N-deS case, presumably due to the presence of residual N-sulfates on the N-deS heparin. This indicates that multivalency-aided diffusive behavior is unlikely to play an important role in initiating the recruitment of HPV16 to the cell surface.

DATA AVAILABILITY

All the data are available upon request to authors. Codes are available at https://github.com/darioconcaUMU/HPV_HSsulfation.

ACKNOWLEDGMENTS

We acknowledge the Biochemical Imaging Center at Umeå University and the National Microscopy Infrastructure, NMI (VR-*RFI* 2019-00217) for providing access to AFM and QCM-D. D.C. thanks the European Union's Horizon 2020 research and innovation program under the Marie Skłodowska-Curie grant agreement no. 101027987 for the funding received. M.B. thanks the Knut and Alice Wallenberg Foundation, the Swedish Research Council (VR grants 2017-04029; 2020-06242; 2024-04375; 2024-02928), the Kempe Foundations, and the Wenner-Gren Foundations (UPD2018-0193) for financial support. M.S. acknowledges the German Research Foundation (DFG, grants SCHE 1552/6-1, SCHE 1552/3-2, INST 211/1029-1, SFB1348/2 A09 to M.S.) for funding. Hudson Pace is acknowledged for the help with fluorescent labeling of GAG for the quantification of the adlayer's GAG surface density.

AUTHOR CONTRIBUTIONS

M.B. and M.S. conceived and supervised the project. F.B. performed the experiments and analyzed the data. D.V.C. developed the theoretical framework and wrote MATLAB code for analysis. Y.A., K.S., and D.V.C. analyzed the SPT data. L.S.M. prepared and labeled the virus samples. J.S. and A.D. assisted with SPR data acquisition and analysis. F.B., D.V.C., and M.B. wrote the original draft. All the authors reviewed and edited the manuscript.

DECLARATION OF INTERESTS

The authors declare that they do not have any competing interests.

SUPPLEMENTAL INFORMATION

Supplemental information can be found online at https://doi.org/10.1016/j.bpj.2026.05.014.

REFERENCES

1. Domma, A. J., L. A. Henderson, …, J. P. Kamil. 2024. Chapter Three - Uncloaking the viral glycocalyx: How do viruses exploit glycoimmune checkpoints? *In* Advances in Virus Research. R. MacDiarmid, B. Lee, and M. Beer, eds Academic Press, pp. 63–110.
2. Olofsson, S., and T. Bergström. 2005. Glycoconjugate glycans as viral receptors. *Ann. Med.* 37:154–172. https://doi.org/10.1080/07853890510007340.
3. Jurriaan, H., P. J. Leonard, …, R. B. Jan. 2017. Multivalency: Concepts, Research & Applications. John Wiley & Sons Ltd.
4. Lu, W., and R. J. Pieters. 2019. Carbohydrate–protein interactions and multivalency: implications for the inhibition of influenza A virus infections. *Expert Opin. Drug Discov.* 14:387–395. https://doi.org/10.1080/17460441.2019.1573813.
5. Mammen, M., S. K. Choi, and G. M. Whitesides. 1998. Polyvalent Interactions in Biological Systems: Implications for Design and Use of Multivalent Ligands and Inhibitors. *Angew Chem. Int. Ed. Engl.* 37:2754–2794. https://doi.org/10.1002/(SICI)1521-3773(19981102)37:20<2754::AID-ANIE2754>3.0.CO;2-3.
6. Boulant, S., M. Stanifer, and P. Y. Lozach. 2015. Dynamics of virus-receptor interactions in virus binding, signaling, and endocytosis. *Viruses.* 7:2794–2815. https://doi.org/10.3390/v7062747.
7. Müller, M., D. Lauster, …, S. Block. 2019. Mobility-Based Quantification of Multivalent Virus-Receptor Interactions: New Insights Into Influenza A Virus Binding Mode. *Nano Lett.* 19:1875–1882. https://doi.org/10.1021/acs.nanolett.8b04969.
8. Conca, D. V., F. Bano, …, M. Bally. 2025. Variant-Specific Interactions at the Plasma Membrane: Heparan Sulfate's Impact on SARS-CoV-2 Binding Kinetics. *Anal. Chem.* 97:4318–4328. https://doi.org/10.1021/acs.analchem.4c04283.
9. Peerboom, N., S. Block, …, M. Bally. 2017. Binding Kinetics and Lateral Mobility of HSV-1 on End-Grafted Sulfated Glycosaminoglycans. *Biophys. J.* 113:1223–1234. https://doi.org/10.1016/j.bpj.2017.06.028.
10. Bally, M., S. Block, …, G. E. Rydell. 2021. Physicochemical tools for studying virus interactions with targeted cell membranes in a molecular and spatiotemporally resolved context. *Anal. Bioanal. Chem.* 413:7157–7178. https://doi.org/10.1007/s00216-021-03510-5.
11. Delaveris, C. S., E. R. Webster, …, C. R. Bertozzi. 2020. Membrane-tethered mucin-like polypeptides sterically inhibit binding and slow fusion kinetics of influenza A virus. *Proc. Natl. Acad. Sci. USA.* 117:12643–12650. https://doi.org/10.1073/pnas.1921962117.
12. Honigfort, D. J., M. O. Altman, …, K. Godula. 2021. Glycocalyx crowding with mucin mimetics strengthens binding of soluble and virus-associated lectins to host cell glycan receptors. *Proc. Natl. Acad. Sci. USA.* 118:e2107896118. https://doi.org/10.1073/pnas.2107896118.
13. Sarrazin, S., W. C. Lamanna, and J. D. Esko. 2011. Heparan sulfate proteoglycans. *Cold Spring Harbor Perspect. Biol.* 3:a004952. https://doi.org/10.1101/cshperspect.a004952.
14. Tumova, S., A. Woods, and J. R. Couchman. 2000. Heparan sulfate proteoglycans on the cell surface: versatile coordinators of cellular functions. *Int. J. Biochem. Cell Biol.* 32:269–288. https://doi.org/10.1016/s1357-2725(99)00116-8.
15. Joyce, J. G., J. S. Tung, …, P. M. Keller. 1999. The L1 major capsid protein of human papillomavirus type 11 recombinant virus-like particles interacts with heparin and cell-surface glycosaminoglycans on human keratinocytes. *J. Biol. Chem.* 274:5810–5822. https://doi.org/10.1074/jbc.274.9.5810.
16. Paiardi, G., S. Richter, …, R. C. Wade. 2022. The binding of heparin to spike glycoprotein inhibits SARS-CoV-2 infection by three mechanisms. *J. Biol. Chem.* 298:101507. https://doi.org/10.1016/j.jbc.2021.101507.
17. Esko, J. D., and S. B. Selleck. 2002. Order out of chaos: assembly of ligand binding sites in heparan sulfate. *Annu. Rev. Biochem.* 71:435–471. https://doi.org/10.1146/annurev.biochem.71.110601.135458.
18. Spillmann, D. 2001. Heparan sulfate: Anchor for viral intruders? *Biochimie.* 83:811–817. https://doi.org/10.1016/s0300-9084(01)01290-1.

19. Ströh, L. J., and T. Stehle. 2014. Glycan Engagement by Viruses: Receptor Switches and Specificity. *Annu. Rev. Virol.* 1:285–306. https://doi.org/10.1146/annurev-virology-031413-085417.

20. Richards, K. F., M. Bienkowska-Haba, …, M. Sapp. 2013. Multiple heparan sulfate binding site engagements are required for the infectious entry of human papillomavirus type 16. *J. Virol.* 87:11426–11437. https://doi.org/10.1128/JVI.01721-13.

21. Rommel, O., J. Dillner, …, M. Sapp. 2005. Heparan sulfate proteoglycans interact exclusively with conformationally intact HPV L1 assemblies: basis for a virus-like particle ELISA. *J. Med. Virol.* 75:114–121. https://doi.org/10.1002/jmv.20245.

22. Shafti-Keramat, S., A. Handisurya, …, R. Kirnbauer. 2003. Different heparan sulfate proteoglycans serve as cellular receptors for human papillomaviruses. *J. Virol.* 77:13125–13135. https://doi.org/10.1128/jvi.77.24.13125-13135.2003.

23. Cerqueira, C., Y. Liu, …, M. Schelhaas. 2013. Heparin increases the infectivity of Human Papillomavirus type 16 independent of cell surface proteoglycans and induces L1 epitope exposure. *Cell. Microbiol.* 15:1818–1836. https://doi.org/10.1111/cmi.12150.

24. Galati, L., P. Di Bonito, …, T. Gheit. 2024. HPV16 Phylogenetic Variants in Anogenital and Head and Neck Cancers: State of the Art and Perspectives. *Viruses.* 16:904. https://doi.org/10.3390/v16060904.

25. de Sanjose, S., W. G. Quint; …, Retrospective International Survey and HPV Time Trends Study Group. 2010. Human papillomavirus genotype attribution in invasive cervical cancer: a retrospective cross-sectional worldwide study. *Lancet Oncol.* 11:1048–1056. https://doi.org/10.1016/S1470-2045(10)70230-8.

26. Wei, F., D. Georges, …, G. M. Clifford. 2024. Causal attribution of human papillomavirus genotypes to invasive cervical cancer worldwide: a systematic analysis of the global literature. *Lancet.* 404:435–444. https://doi.org/10.1016/S0140-6736(24)01097-3.

27. Dasgupta, J., M. Bienkowska-Haba, …, X. S. Chen. 2011. Structural basis of oligosaccharide receptor recognition by human papillomavirus. *J. Biol. Chem.* 286:2617–2624. https://doi.org/10.1074/jbc.M110.160184.

28. Guan, J., S. M. Bywaters, …, S. Hafenstein. 2017. Cryoelectron Microscopy Maps of Human Papillomavirus 16 Reveal L2 Densities and Heparin Binding Site. *Structure.* 25:253–263. https://doi.org/10.1016/j.str.2016.12.001.

29. Feng, Y., D. van Bodegraven, …, W. H. Roos. 2024. Glycan-induced structural activation softens the human papillomavirus capsid for entry through reduction of intercapsomere flexibility. *Nat. Commun.* 15:10076. https://doi.org/10.1038/s41467-024-54373-0.

30. Bano, F., L. Soria-Martinez, …, M. Schelhaas. 2024. Site-specific sulfations regulate the physicochemical properties of papillomavirus-heparan sulfate interactions for entry. *Sci. Adv.* 10:eado8540. https://doi.org/10.1126/sciadv.ado8540.

31. Becker, M., L. Greune, …, M. Schelhaas. 2018. Extracellular Conformational Changes in the Capsid of Human Papillomaviruses Contribute to Asynchronous Uptake into Host Cells. *J. Virol.* 92:e02106-17. https://doi.org/10.1128/JVI.02106-17.

32. Sapp, M., and M. Bienkowska-Haba. 2009. Viral entry mechanisms: human papillomavirus and a long journey from extracellular matrix to the nucleus. *FEBS J.* 276:7206–7216. https://doi.org/10.1111/j.1742-4658.2009.07400.x.

33. Schelhaas, M., H. Ewers, …, A. Helenius. 2008. Human papillomavirus type 16 entry: retrograde cell surface transport along actin-rich protrusions. *PLoS Pathog.* 4:e1000148. https://doi.org/10.1371/journal.ppat.1000148.

34. Thakar, D., E. Migliorini, …, R. P. Richter. 2014. A quartz crystal microbalance method to study the terminal functionalization of glycosaminoglycans. *Chem. Commun.* 50:15148–15151. https://doi.org/10.1039/c4cc06905f.

35. Emilsson, G., R. L. Schoch, …, A. B. Dahlin. 2015. Strongly stretched protein resistant poly(ethylene glycol) brushes prepared by grafting-to. *ACS Appl. Mater. Interfaces.* 7:7505–7515. https://doi.org/10.1021/acsami.5b01590.

36. Tinevez, J. Y., N. Perry, …, K. W. Eliceiri. 2017. TrackMate: An open and extensible platform for single-particle tracking. *Methods.* 115:80–90. https://doi.org/10.1016/j.ymeth.2016.09.016.

37. Vega, A. R., S. A. Freeman, …, K. Jaqaman. 2018. Multistep Track Segmentation and Motion Classification for Transient Mobility Analysis. *Biophys. J.* 114:1018–1025. https://doi.org/10.1016/j.bpj.2018.01.012.

38. Abidine, Y., L. Liu, …, M. Bally. 2022. Cellular Chondroitin Sulfate and the Mucin-like Domain of Viral Glycoprotein C Promote Diffusion of Herpes Simplex Virus 1 While Heparan Sulfate Restricts Mobility. *Viruses.* 14:1836. https://doi.org/10.3390/v14081836.

39. Tzimiropoulos, G., V. Argyriou, …, T. Stathaki. 2010. Robust FFT-based scale-invariant image registration with image gradients. *IEEE Trans. Pattern Anal. Mach. Intell.* 32:1899–1906. https://doi.org/10.1109/TPAMI.2010.107.

40. Knappe, M., S. Bodevin, …, M. Sapp. 2007. Surface-exposed amino acid residues of HPV16 L1 protein mediating interaction with cell surface heparan sulfate. *J. Biol. Chem.* 282:27913–27922. https://doi.org/10.1074/jbc.M705127200.

41. Becker, M., D. V. Conca, …, G. Gerold. 2023. Efficient clathrin-mediated entry of enteric adenoviruses in human duodenal cells. *J. Virol.* 97:e0077023. https://doi.org/10.1128/jvi.00770-23.

42. Birkmann, A., K. Mahr, …, F. Neipel. 2001. Cell surface heparan sulfate is a receptor for human herpesvirus 8 and interacts with envelope glycoprotein K8.1. *J. Virol.* 75:11583–11593. https://doi.org/10.1128/JVI.75.23.11583-11593.2001.

43. Gunnarsson, A., P. Jönsson, …, F. Höök. 2008. Single-molecule detection and mismatch discrimination of unlabeled DNA targets. *Nano Lett.* 8:183–188. https://doi.org/10.1021/nl072401j.

44. Bally, M., A. Gunnarsson, …, F. Höök. 2011. Interaction of single viruslike particles with vesicles containing glycosphingolipids. *Phys. Rev. Lett.* 107:188103. https://doi.org/10.1103/PhysRevLett.107.188103.

45. Rankl, C., F. Kienberger, …, P. Hinterdorfer. 2008. Multiple receptors involved in human rhinovirus attachment to live cells. *Proc. Natl. Acad. Sci. USA.* 105:17778–17783. https://doi.org/10.1073/pnas.0806451105.

46. Reiter-Scherer, V., J. L. Cuellar-Camacho, …, J. P. Rabe. 2019. Force Spectroscopy Shows Dynamic Binding of Influenza Hemagglutinin and Neuraminidase to Sialic Acid. *Biophys. J.* 116:1577. https://doi.org/10.1016/j.bpj.2019.03.032.

47. Schelhaas, M., B. Shah, …, A. Helenius. 2012. Entry of human papillomavirus type 16 by actin-dependent, clathrin- and lipid raft-independent endocytosis. *PLoS Pathog.* 8:e1002657. https://doi.org/10.1371/journal.ppat.1002657.

48. Bano, F., M. I. Tammi, …, R. P. Richter. 2018. Single-Molecule Unbinding Forces between the Polysaccharide Hyaluronan and Its Binding Proteins. *Biophys. J.* 114:2910–2922. https://doi.org/10.1016/j.bpj.2018.05.014.

49. Day, P. M., and M. Schelhaas. 2014. Concepts of papillomavirus entry into host cells. *Curr. Opin. Virol.* 4:24–31. https://doi.org/10.1016/j.coviro.2013.11.002.

50. Day, P. M., and J. T. Schiller. 2009. The role of furin in papillomavirus infection. *Future Microbiol.* 4:1255–1262. https://doi.org/10.2217/fmb.09.86.

51. Raff, A. B., A. W. Woodham, …, W. M. Kast. 2013. The evolving field of human papillomavirus receptor research: a review of binding and entry. *J. Virol.* 87:6062–6072. https://doi.org/10.1128/JVI.00330-13.

Biophysical Journal, Volume *125*

Supplemental information

Multivalency influences virus dynamics at the glycocalyx: A study of glycosaminoglycan binding in HPV16 entry

Dario Valter Conca, Fouzia Bano, Yara Abidine, Laura Soria Martinez, Kerstin Seier, Justas Svirelis, Andreas Dahlin, Mario Schelhaas, and Marta Bally

Supporting material

Multivalency influences virus dynamics at the glycocalyx: a study of glycosaminoglycan binding in HPV16 entry

Dario Valter Conca[a*], Fouzia Bano[a*], Yara Abidine[a], Laura Soria Martinez[b], Kerstin Seier[a], Justas Svirelis[c], Andreas Dahlin[c], Mario Schelhaas[b], Marta Bally[a,d†]

[a]*Department of Clinical Microbiology, Wallenberg Centre for Molecular Medicine and Umeå Centre for Microbial Research, Umeå University, Umeå, Sweden*
[b]*Institute of Cellular Virology, ZMBE, University of Münster, Münster, Germany*
[c]*Department of Chemistry and Chemical Engineering, Chalmers University of Technology, Gothenburg 412 96, Sweden*
[d]*Science for Life Laboratory (SciLifeLab), Umeå University, Sweden*

*Contributed equally to this work
†Corresponding author

Contents

1. Supplementary reagents
2. Biotinylation of heparin and its derivatives
3. Measuring the lateral mobility of cross-linked streptavidin by FRAP
4. Characterization of the glycosaminoglycan (GAG) films by Quartz Crystal Microbalance with Dissipation (QCM-D)
5. Quantification of the optical mass of heparin by SPR
6. Estimation of the grafting coverage and chain spacing of a mixed layer of heparin and PEG
7. Additional Equilibrium Fluctuation Analysis data
8. Diffusion-controlled particle flux calculation
9. Summary of the AFM-based SMFS data
10. Markov-Model for multivalent binding
11. Probability of establishing multiple bonds with end-grafted heparin
12. Comparison between the Markov-multivalency model and experimental results
13. HPV16 diffusion on heparin film
14. Simulation of TIRF images of immobile particles

15. Model of multivalency aided diffusion
16. Effects of residual sulfation on particle binding and mobility
17. Discrete Markov model for heparin sulfation

1. Supplementary reagents

Reagents, proteins, lipids and buffer: Lyophilized fluorescently streptavidin with atto-488 (SAv-488; Sigma Aldrich, St. Louis, MO) was dissolved in milli-Q water at 1 mg/mL and stored at -20 ºC. Labelled lipids (1,2-dioleoyl-sn-glycero-3-phosphoethanolamine-N-(lissamine rhodamine B sulfonyl, 18:1 Liss Rhod PE; 810150, avanti polar lipids) were purchased from Sigma Aldrich. Lyophilized Bovine Serum Albumin (BSA) Fraction V was obtained from Roche, Germany, dissolved in milli-Q water at concentration of 10% (weight/volume, w/v) and stored at -20ºC. Anti-Heparan Sulphate (antiHS, clone F58-10E4) was obtained from AMSBIO, England. Both goat anti-mouse IgM secondary antibody, Horseradish peroxidase (HRP) conjugate (catalog #31440) and HRP substrate (SuperSignal™ West Femto Maximum Sensitivity, catalog # 34096) were from Thermo Fisher Scientific.

Phosphate buffered saline (PBS) buffer at pH 7.4 was used in all experiments; it was prepared by dissolving 1 PBS tablet (Medicago AB, Uppsala, Sweden) in 1 L milli-Q water (Millipore integral system, Molsheim, France) and filtered through 0.2 µm filters (Sarstedt, Germany).

2. Biotinylation of heparin and its derivatives

This study relies on commercially available heparin and three selectively desulfated heparins, whose properties are summarized in Table S1. The heparins were biotinylated via oxime ligation at their reducing end as described previously (1,2).

Table S1: GAGs used in this study

Name	Molecular weight (kDa)	n_{ds}§	Sulphate group per disaccharide#
Heparin	15	25	2.42
2-O-desulfated heparin (2-O-deS)	15	25	1.75
6-O desulfated heparin (6-O-deS)	15	25	1.48
N-desulfated heparin (N-deS)	15	25	1.55
Hyaluronan (HA)	23	57	0

§Values for number of disaccharide unit per chain were estimated from the average molecular weight of heparin.

[#]Average number of sulfate groups per disaccharide were calculated from disaccharides analysis provided by the supplier.

3. Measuring the lateral mobility of cross-linked streptavidin by FRAP

We tested the influence of streptavidin crosslinking with PFA, on the protein's lateral mobility by performing Fluorescence Recovery after Photobleaching (FRAP) measurements of fluorescently labelled streptavidin.

The detailed protocol used to immobilize GAGs on supported lipid bilayers though a streptavidin bridge is described in the main text. The concentrations and incubation times for each step for FRAP experiments were as following: SLBs of POPC:DOPE-Cap-B (90:10) and SLBs of Rhod PE:POPC with 1:99 molar ratio – 100 µg/mL, 30 mins; SAv-488 – 40 µg/mL, 60 mins; biotinylated chondroitin sulfate (bCSA, Aqua Biochem, Dessau, Germany) – 100 µg/mL, 30 mins; PFA – 2%, 15 mins. FRAP experiments were performed with an inverted Nikon Eclipse Ti2-E microscope using lasers with 488 nm and 565 nm wavelengths. Both lasers with 60% of total power were used for bleaching for 10 s. For recovery, a series of images consisting of 704 x 704 pixels with a 0.183 µm pixel width of was recorded at 0.5 fps with 10% of total laser power. The timelapse duration was 60 mins (30 mins for Rhod PE:POPC SLBs). The images were analyzed using ImageJ for intensity profiles. Our experiments indicate that the mobile fraction of labelled streptavidin is reduced from 57.5±3.5% (solid red line) to 5.5±0.3% (dashed black line) with PFA incubation (Fig. S1). Addition of the biotinylated GAG did not influence the streptavidin's diffusion behavior (Fig. S1, dashed red line. As a control, we used pure supported lipid bilayers composed of POPC:Rhod PE (99:1 molar ratio) whose lipids, as expected, exhibited nearly full recovery (96% mobile fraction, black line, Fig. S1). Two independent experiments were performed for each condition.

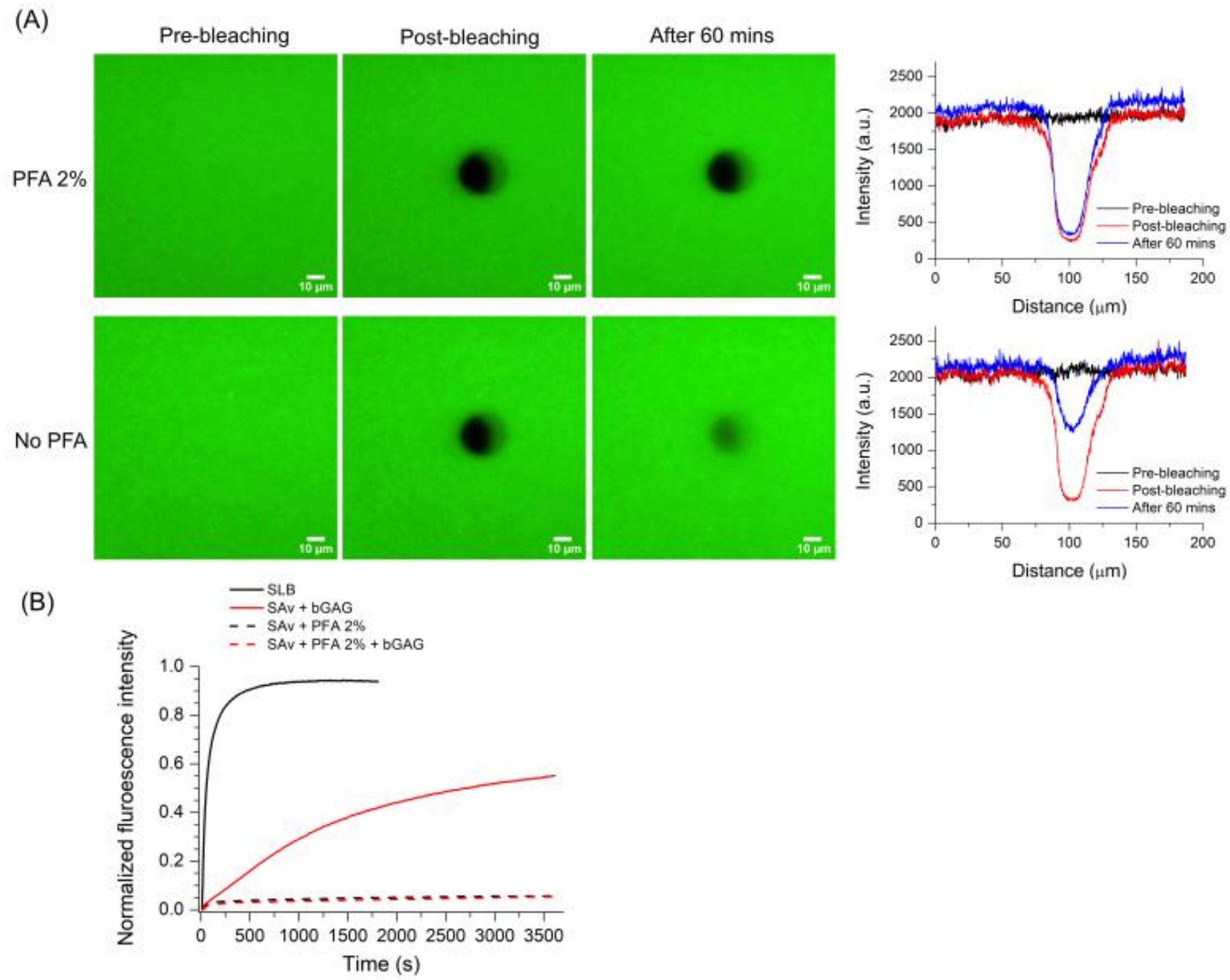

Figure S1. FRAP experiment to probe the lateral mobility of streptavidin before and after crosslinking with 2% PFA. (A) Representative images of fluorescently labelled streptavidin (SAv-488) on SLBs with 10% biotin with 2% PFA (top) or without. The images are taken before bleaching (pre-bleaching), right after bleaching (post-bleaching) and after 60 mins. The images are shown along with their intensity line profiles over the bleached area. Scale bar = 10 µm. (B) Intensity of the bleached area (taken from the line profiles) as a function of time are displayed to observe the recovery process after bleaching. Black solid line: control SLBs containing fluorescent lipids (Rhod PE); red solid line: SLBs containing 10% biotinylated lipids after exposing them with fluorescently labelled streptavidin (SAv-488) and addition of the biotinylated GAG (bGAG) chondroitin sulfate dashed lines: the SAv-488 was crosslinked with 2% PFA (dashed black line) following by addition of the bGAG (dashed red line).

4. Characterization of the glycosaminoglycan (GAG) films by Quartz Crystal Microbalance with Dissipation (QCM-D)

We used QCM-D to verify that the crosslinking of streptavidin with 2% paraformaldehyde (PFA) does not prevent the immobilization of biotinylated heparin (Fig. S2). QCM-D measures the shifts in resonance frequency (Δf), and dissipation (ΔD) of a sensor upon adsorption of molecules on its surface. The QCM-D signal is sensitive to the areal density

including the coupled water molecules and the mechanical properties of the surface-immobilized layer. Basically, a decrease in frequency corresponds to increased mass, while a high or low shift in dissipation corresponds to a soft or rigid layer, respectively.

QCM-D measurements were performed as previously described (3). Briefly, the QCM-D signal was collected using sensors with fundamental frequency of 5 kHz and silica coating (AWS SNS 000049A, Advanced Wave Sensors (AWSensors) S.L., Spain). Before use, the sensors were immersed in 2% (w/v) sodium dodecyl sulfate (SDS) for 1 hr, rinsed thoroughly with milli-Q water, blown dry with N_2, and treated in a UV/ozone cleaner (Bioforce Nanoscience, Ames, IA) for 30 min.

QCM-D data was collected with an X4 system (AWSensors, Spain) connected with four independent pumps. Buffers and biomolecule solutions (i.e., small unilamellar vesicles containing biotinylated lipids, streptavidin and biotinylated GAG e.g., heparin) were dispensed at a flow rate of 20 µL/min unless otherwise stated, at 22 ºC. The QCM-D response (frequency shift Δf and dissipation ΔD) was collected at six overtones (i = 3, 5, 7, 9, 11, 13) and data for f_i = 3 is presented, unless otherwise stated. Before use, PBS was degassed using a sonicator (Elmasonic, S40H, Singen, Germany).

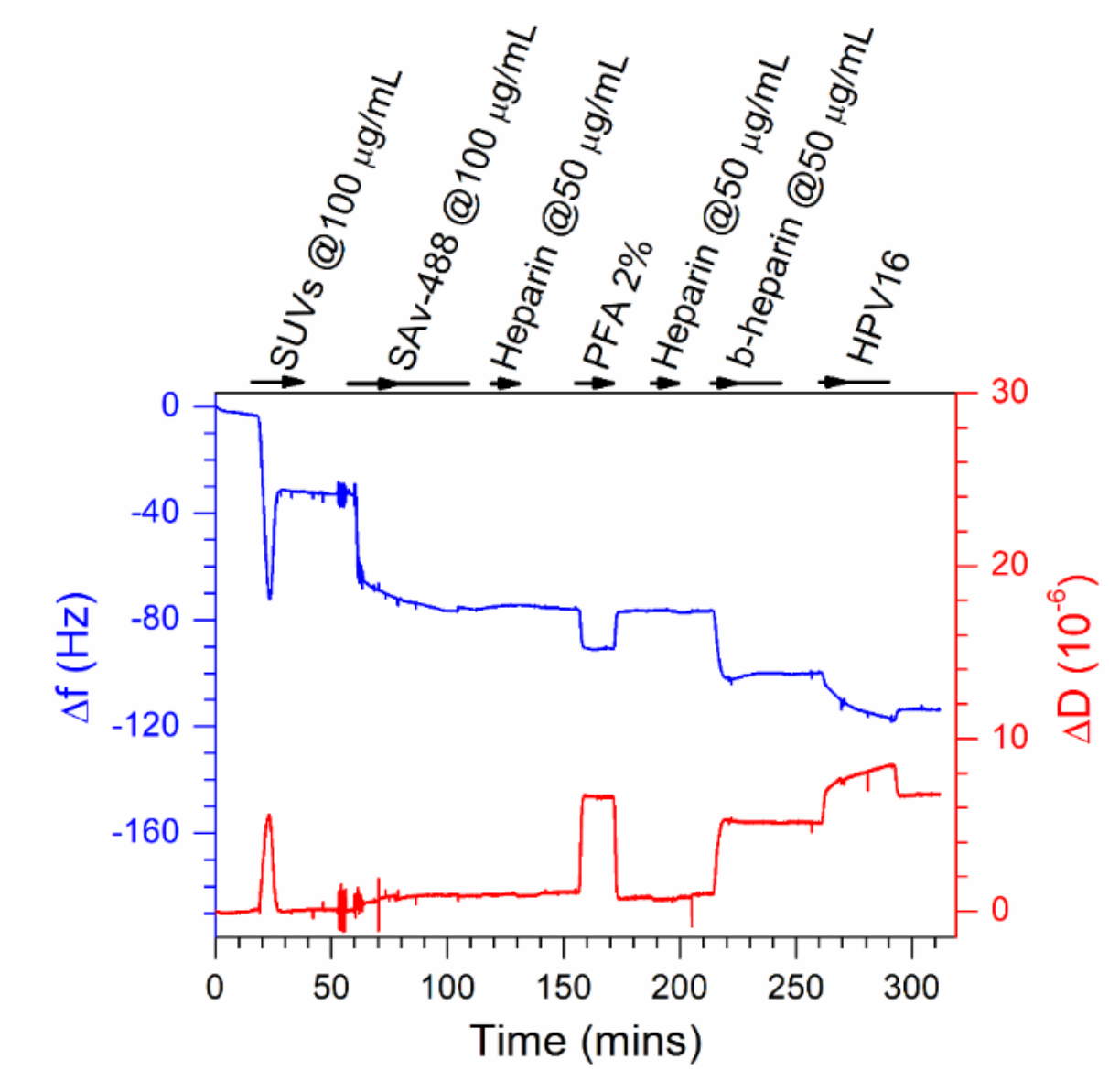

Figure S2. QCM-D experiments showing the build-up of a biotinylated heparin layer on a densely packed streptavidin layer. The shift in resonance frequency (blue) and the dissipation (red) associated with the binding of biotinylated heparin to a cross-linked densely packed streptavidin layer (Δf = -25.5 ± 2.8 Hz and ΔD = 4.4 ± 0.4 $\times 10^{-6}$) is close to reported values (3), supporting that the addition of 2% PFA did not have any effect on the immobilization of biotinylated heparin. Arrow indicates continuous flow of the

biomolecules. Line following an arrow indicates incubation with biomolecules without continuous flow (i.e., incubation in a still solution). Empty time slots represent rinsing with PBS under flow.

5. Quantification of the optical mass of heparin by SPR

To quantify the mass of adsorbed biomolecules per surface area or surface mass density (Γ) from the recorded SPR sensogram signal ($\Delta\theta$) presented in Figure 1B, we have used the reported expression (4):

$$\Gamma = \frac{\Delta\theta\delta}{S_0 dn/dc_m} \qquad \text{(Eq. S1)}$$

Here dn/dc_m is the increase in refractive index per mass concentration of the binding species (see Table S1 for values of different biomolecules used in this study). S_0 is the bulk sensitivity of the instrument (angular shift per refractive index increment), which was estimated from Fresnel models at 670 nm to be 131 degrees (5). δ is the decay length of the evanescent field, which is calculated from the dispersion relation and the field distribution of the surface plasmons (6). Considering experimentally determined parameters for the Cr and Au thickness and permittivity, we have δ = 228 nm for our system.

Table S2: Refractive index increment per mass concentration (dn/dc_m) of biomolecules used in this study

	**dn/dc_m (mL/g)**
Heparin and its derivatives (2-O-deS, 6-O-deS and N-deS)	0.132[ǂ]
HA	0.16[ǂ]
PEG	0.134[§]

[ǂ]Values were taken from Altgärde et al. (7)
[§]Value was taken from Emilsson et al. (4)

Table S3: Summary of SPR angle shifts, surface mass densities, grafting coverages at saturation, and the average spacing between GAG chains for the different GAGs added at

a bulk concentration of 10 µg/mL. Values ± standard deviation were obtained from 2 independent experiments except for HA.

Name	SPR resonance shift at 670 nm (º)	Mass density (ng/cm^2)	Grafting coverage ($10^{11}/cm^2$)	Spacing between chains (nm)	Density of sulphate group ($10^{13}/cm^2$)
Heparin	0.051 ± 0.011	67.2 ± 14.9	27.0 ± 5.9	6.1 ± 0.7	16.3 ± 3.6
2-O-deS	0.056 ± 0.012	78.4 ± 15.8	31.5 ± 6.4	5.6 ± 0.6	13.8 ± 2.8
6-O-deS	0.070 ± 0.039	92.3 ± 52.2	37.1 ± 21	5.2 ± 1.5	13.7 ± 7.8
N-deS	0.072 ± 0.017	94.9 ± 23.4	38.1 ± 9.0	5.1 ± 0.6	14.8 ± 3.5
HA	0.113	122.9	32.2	5.6	/

6. Estimation of the grafting coverage and chain spacing of a mixed layer of heparin and PEG

We estimated the grafting coverage and chain spacing of a mixed layer of heparin and polyethylene glycol (PEG) using direct detection of labelled biotinylated GAGs (N-deS in this case) or an immunoassay.

Approach 1: Fluorescent labelled biotinylated GAG

Fluorescent labelling. bGAGs were fluorescently labelled by covalent conjugation to an amine-reactive dye. 50 µL of 2.5 mg/mL biotinylated N-deS heparin (biotinylated N-deS) heparin was first mixed with 7 µL of 1M $NaHCO_3$ (pH 9.0) buffer. Then 5 µL of Alexa Fluor™ 488 5-SDP Ester (Thermo Fisher Scientific) was added, mixed well, and incubated at room temperature for 1 hr, further mixing via vortex every 10 mins. The free dye was removed with 3 kDa molecular cut off spin column filters (Microcon YM-3, Millipore) by adding 100 µL of milli-Q water and centrifuging at 14,000 rcf for 40 mins until the flow through became clear (>7 times). We chose to label biotinylated N-deS as it maximizes the number of primary amines to react with the fluorescent dye. The concentration of labelled biotinylated N-deS was measured using the cetylpyridinium chloride (CPC) turbidity test as described elsewhere (3,8). Very briefly, 50 µL of CPC reagent (0.2% w/v of CPC and 133 mM of $MgCl_2$; both from Sigma) was mixed with 50 µL of GAGs solution in a 96-well cleared bottom plate (VWR). The solution was then carefully mixed to avoid bubbles and incubated at room temperature for 30 mins. The absorbance was measured at 405 nm using a spectrometer (Tecan, Infinite F200 PRO, Switzerland). At 405 nm, fluorophore

absorption was negligible. We also used QCM-D to validate the formation of a labelled biotinylated N-deS heparin layer on streptavidin. Despite a larger frequency shift and dissipation associated with the additional mass of the fluorophore, the data show that the softness of the heparin layer does not change after labelling, indicating that the mechanical properties of heparin are unchanged by the conjugation (Fig. S3A).

The use of fluorescently labelled GAGs to calculate the grafting density requires that fluorescence intensity of a single chain being independent from the grafting concentration, i.e. that no interchain quenching is present at high GAG concentration. To demonstrate that this is the case, we exposed surface-grafted FluoN-deS both at saturation and at 1:5 molar ratio bPEG:FluoN-deS, to focused 488 nm laser at 50% power for times between 0.1 and 60 seconds and recorded the difference in fluorescence signal before and after bleaching (Figs. S3C, D). At both concentrations, we observed a monotonic exponential decrease of the fluorescence intensity compatible with photobleaching of the illuminated area (Fig S3E). No dequenching, i.e., initial increase of the fluorescence signal, was observed indicating that no intra- or interchain quenching is present and the fluorophore emission is not influenced by the grafting density.

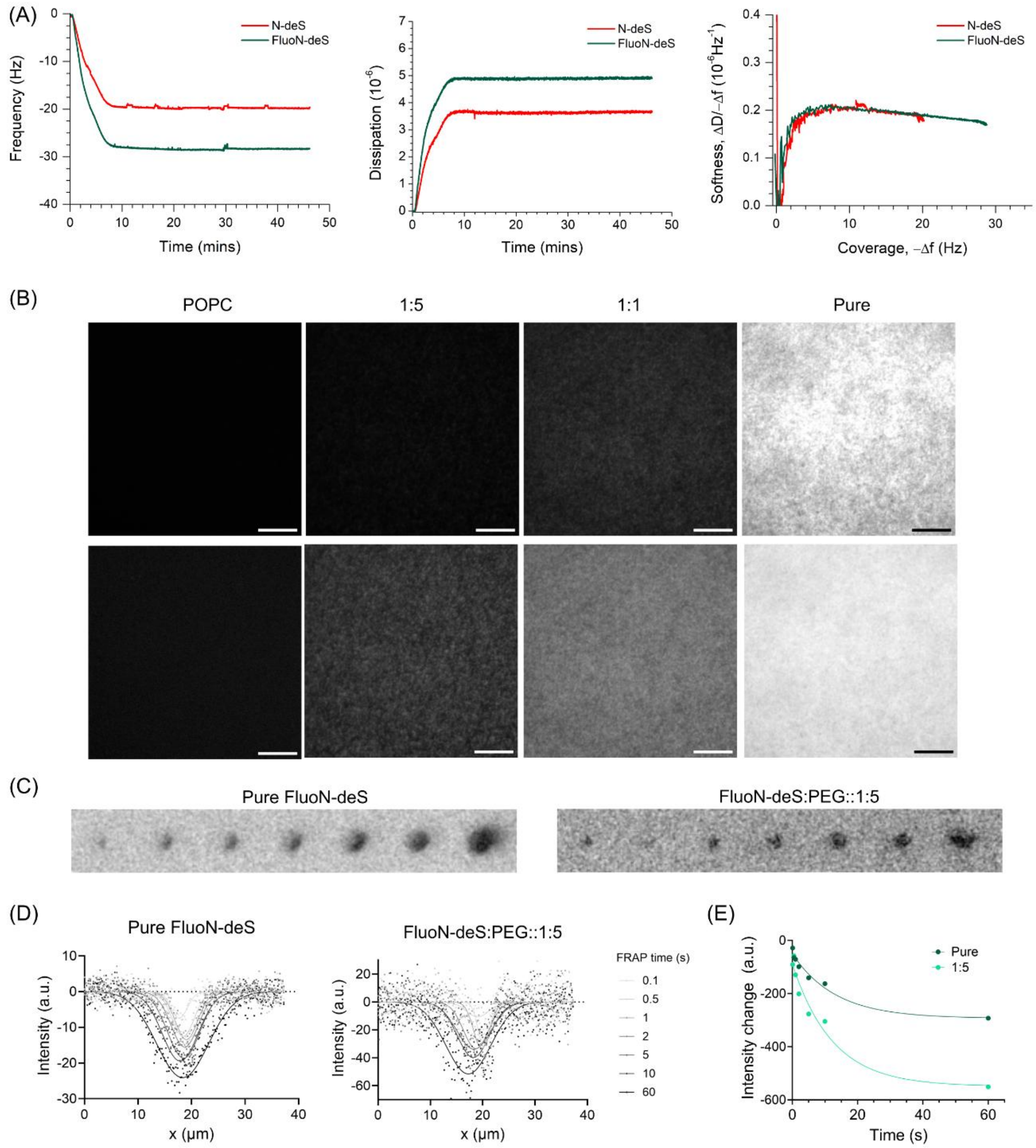

Figure S3. (A) QCM-D plots for labelled (dark yellow) and non-labelled (red) biotinylated N-deS that were immobilized on a streptavidin layer (20 µg/mL, 20 mins) on SLB containing 10% biotin lipids (100 µg/mL, 15 mins), indicating that labelling process did not disrupt bGAG adsorption. An increase in both the final frequency (left) and dissipation (middle) values for FluoN-deS is observed and ascribed to the additional mass of dye. The softness plot (right) displaying the overlap of both samples suggests that labelling does not affect the mechanical properties (softness) of the N-deS heparin chains. (B) Representative fluorescence image of four surfaces for the data presented in Fig. 1C. Top: Original image. Bottom: Same image as top with fluorescence intensity displayed using a logarithmic scale to enhance the difference between dim samples. Scale bar = 10 µm. (C) Bleaching experiments at increasing bleaching time on both pure fluorescent N-desulfated heparin

(FluoN-deS) and the 1:5 molar mixture with PEG (FluoN-deS:PEG::1:5). (D) Line profiles over the bleached regions shown in (C), after subtraction of the pre-bleach intensity (circles). Solid lines represent the best Gaussian fits to the profiles. (E) Maximum reduction of fluorescence intensity after bleaching, quantified as the area between the Gaussian fit and the baseline (y=0) in (D), as a function of bleaching time. Solid lines represent fits to a single exponential with a y-offset, consistent with photobleaching and showing no evidence of dequenching.

Approach 2: Immunoassay

Surface preparation for Immunoassay. Steps to prepare the surface until PFA incubation were identical to approach 1. To prepare heparin and the mixed heparin:PEG layer for this assay, the following concentration and exposure time were used: biotinylated parental hepain, bHeparin – 10µg/mL, 30 mins (Hep10); bHeparin – 1 µg/mL, 30 mins (Hep1); bHeparin and bPEGSH – molar ratio of 1:5, 30 mins (Hep:PEG::1:5); bPEGSH – 25 µg/mL, 30 mins (PEG). Following the rinsing with PBS, all surfaces were further rinsed with 0.2% BSA. The surfaces were then first incubated with a 5 µg/mL solution of antiHS antibody for 2 hr and then with anti-mouse HRP conjugate secondary antibody at final concentration of 0.4 µg/mL for 1 hr following an extensive rinsing with 0.2% BSA. After rinsing, surfaces were exposed to HRP substrate according to manufacturer's instructions and immediately (<1 min) imaged using Amersham Imager 680 (GE Healthcare) at exposure time of 0.5 s, Fig. S4A. Averages from three independent values with their standard deviation are summarized in Fig. S4B. This approach gives a factor of 19.7 ± 4.4 between the surface density of "Hep10" and "Hep:PEG::1:5".

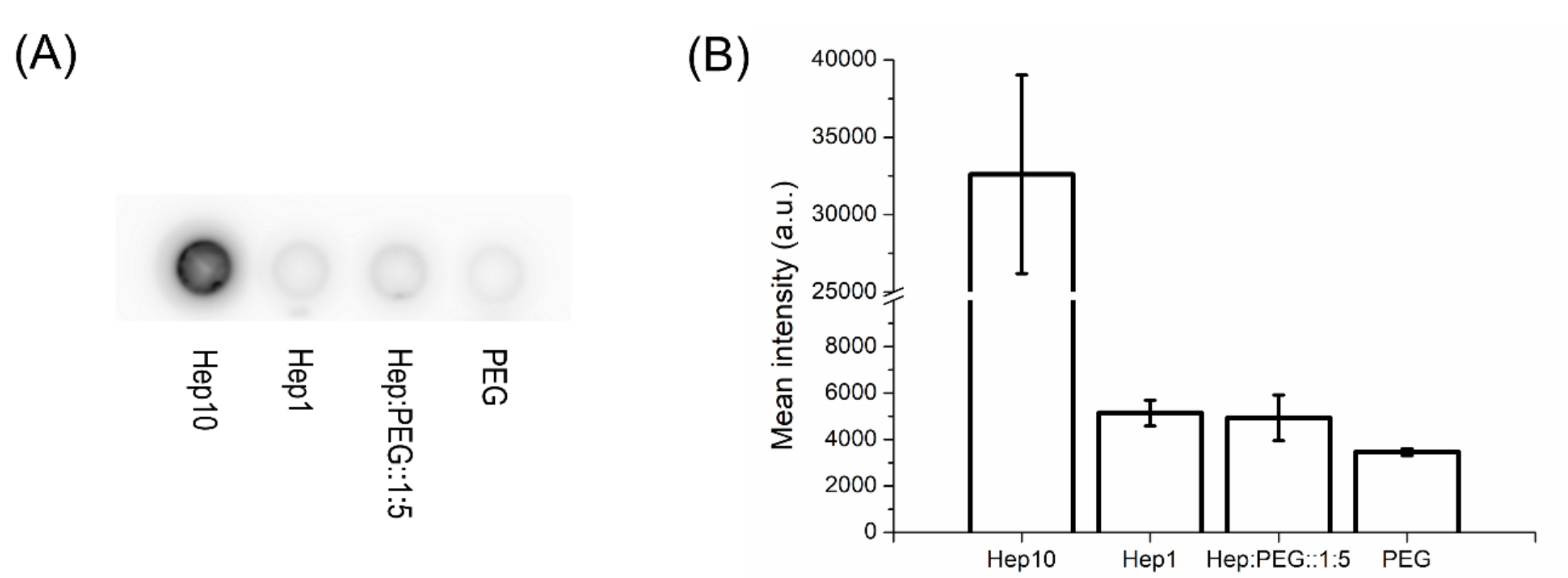

Figure S4. (A) Representative immunostaining assay reading of four surfaces prepared by exposing a streptavidin layer to heparin at final concentration of 10 µg/mL (Hep10) and 1

μg/mL (Hep1), with a mixture of bHeparin and bPEG at molar ratio of 1:5 (Hep:PEG::1:5), and PEG at 25 μg/mL for 30 mins. (B) Chemiluminescence signal from the immunostaining assay in (A). Mean and standard deviation were calculated from 3 separate wells per condition that were prepared on 2 independent days.

Estimation of spacing between GAG chains in mixed GAG:PEG layer. Using the SPR data for heparin or its derivatives films at equilibrium (Table 1) and the average intensity of N-deS films from the assay based on fluorescently labelled GAGs (Fig. S3) prepared at the same bulk concentration of N-deS as SPR (i.e., 10 μg/mL), we have estimated the distance between 2 heparin chains for 1:5 molar ratio of heparin:PEG to be 30 ± 4 nm (Table 1). To extract this distance, we divided the SPR resonance shift of each GAG reported in Table S3 with the reduction factor obtained from our fluorescent labelled GAG assay; this factor or the ratio of the fluorescent signal of FluoN-deS and FluoNdeS:PEG::1:5 (or 1:5) surfaces was 24.2 ± 2.6. Then the mass density and the grafting coverage were calculated as described in the main text. It should be noted that we selected data from approach 1 (i.e., fluorescent labelled GAG assay) to estimate the GAG grafting coverage in the main text, due to the lower uncertainty in the average value.

7. Additional Equilibrium Fluctuation Analysis data

Fig. S5 summarizes the equilibrium surface coverage of HPV16 on the different heparin derivatives.

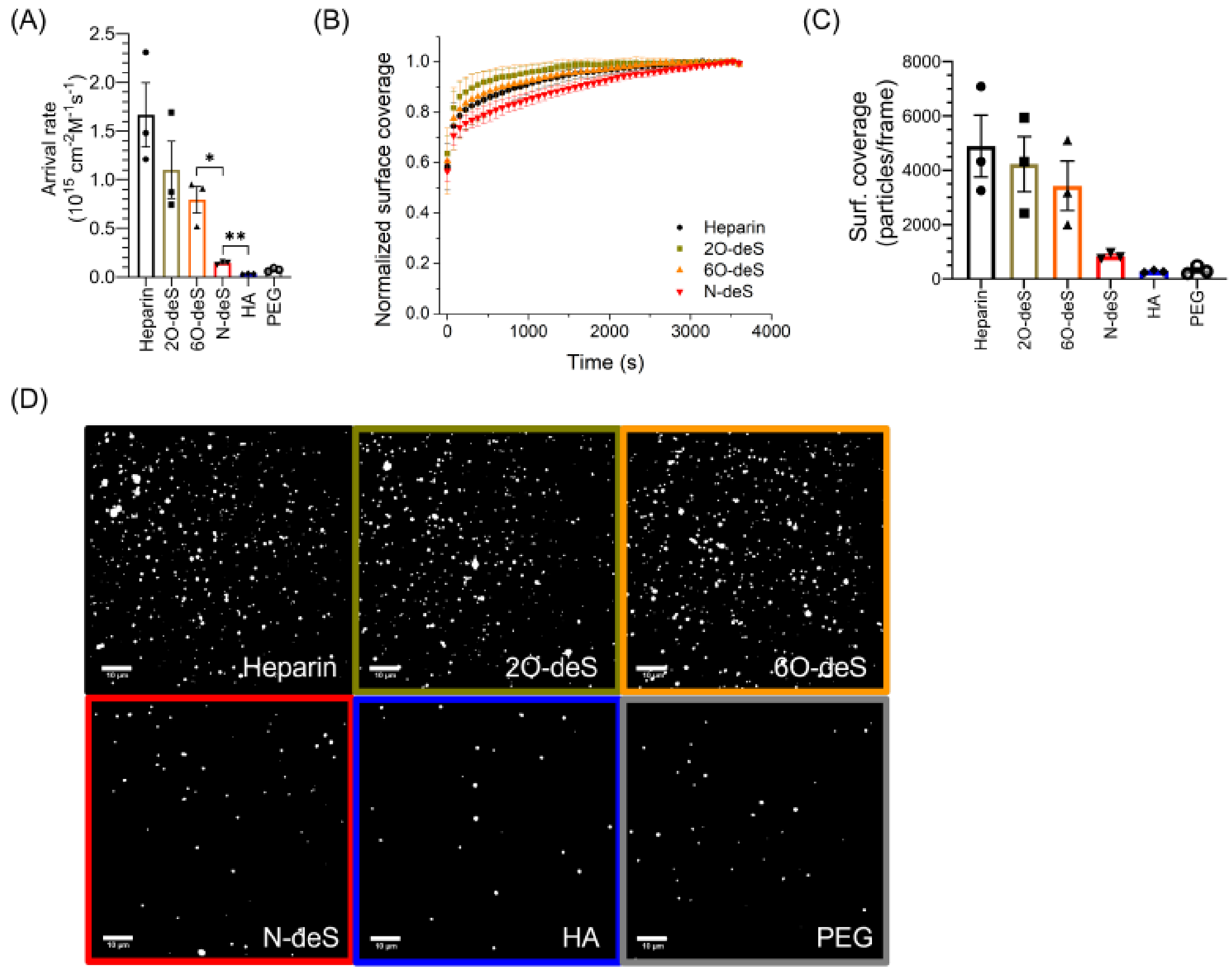

Figure S5. (A) Normalized arrival rate obtained from the linear fit of the data in Figure 2B for the indicated conditions. (B) Normalized surface coverage as a function of time. It is to be noted that the sharp increase observed in the first frames is an artifact associated with issues in recognizing all particles present in the initial frame. Accordingly, the first 150 s of the experiments are not considered in our analyses. Each data point represents average with error bars (i.e., standard deviation) from three independent experiments for four GAGs as indicated (C) Each point in the plot represents average surface coverage from five individual images with the size of 129×129 μm^2 of a single image and bar plots are average values ± S.D. from three independent experiments for tested conditions. (D) Representative images from the first frame of the movies, which were used for kinetics data analysis presented in Figure 2. A Gaussian Blur (=1 pixel) and all images were set to the same intensity threshold. Scale bar = 10 μm.

8. Diffusion-controlled particle flux calculation

To estimate the overall collision rate between the particles, present in well-mixed solution at uniform concentration of C_b with diffusion coefficient of D, and the surface, we can use the following expression derived from Fick's first and second laws:

$$J = C_b \sqrt{\frac{D}{\pi \Delta t}} \quad \text{(Eq. S2)}$$

where Δt represent the observation time of the particle hitting and bouncing off the surface and is the time between each frame in videos recording for our kinetics experiments. Note that Eq. S2 is valid for the surface that is a reflective, implying that all the particles that will reach the surface bounce back and there is no adsorption. For full derivation of Eq. S2, see chapter 2 of reference (9). For HPV16 particles, using $D = 8.73 \times 10^{-8}\ \mathrm{cm^2 s^{-1}}$, $\mathrm{C_b} = 1.14\ \mathrm{pM}$, and $\Delta t = 15\mathrm{s}$, we obtain $J = 3.0 \times 10^4\ \mathrm{collisions.\,cm^{-2}.\,s^{-1}}$. This corresponds to an arrival rate of $r_J = 2.6 \times 10^{16}\ \mathrm{collisions.\,cm^{-2}.\,s^{-1}.\,M^{-1}}$, which is ~1 order of magnitude higher than the experimental values for heparin (Table 2).

9. Summary of the AFM-based SMFS data

The table below (Table S4) summarizes the AFM-based SMFS data obtained in reference (3).

Table S4*: Summary of association constant, interaction time, and associate rate for the interactions between HPV16 and four heparin surfaces obtained from fitting of dynamic force spectra data with Bell-Evans model and binding probability analysis.

**Heparin surfaces**	k_{on}^{SB} **(10⁶ M⁻¹s⁻¹)**	τ^{SB} **(s)**	r_{on} **(s⁻¹)**	$k_{\mathrm{off}}^{SB} = r_{\mathrm{off}}$ (s⁻¹)
Heparin	2.0 ± 0.6	0.087 ± 0.027	3.7 ± 1.2	0.004 ± 0.003
2-O-deS	3.2 ± 0.8	0.055 ± 0.013	5.9 ± 2.5	0.015 ± 0.004
6-O-deS	4.6 ± 1.4	0.038 ± 0.011	8.5 ± 3.9	0.12 ± 0.04
N-deS	1.4 ± 0.3	0.124 ± 0.022	2.6 ± 1.2	12.2 ± 5.2

*Modified table from reference (3).

10. Markov-Model for multivalent binding

It is possible to use a continuous-time Markov chain to model the multivalent binding of a virion to the heparin surface, considering independent bonds and the possibility of rebinding if one or more bonds to the surface are present.

The probability of transition between states in the Markov model, i.e., changing the number of bonds between the viral particle and the heparin substrate, can be expressed in a matrix Q, in which each row represents the transition from a particular state to each other possible state. The off-diagonal element represents the rate of the transition between states, and the diagonal values are the negation of the total rate at which the state transitions to any other state. Considering that the number of bonds can only be increased or reduced by one, we can write the matrices describing the interaction varying the maximum number of bonds that the HPV16 particle can create with the surface. We limit ourselves to the cases between 1 and 5 bonds at the heparin grafting density considered in the study the probability for a particle to form more than 5 bonds is very small.

- For 2 bonds: $Q = \begin{bmatrix} 0 & 0 & 0 \\ r_{\text{off}} & -r_{\text{off}} - r_{\text{on}} & r_{\text{on}} \\ 0 & 2r_{\text{off}} & -2r_{\text{off}} \end{bmatrix}$;
- For 3 bonds: $Q = \begin{bmatrix} 0 & 0 & 0 & 0 \\ r_{\text{off}} & -r_{\text{off}} - 2r_{\text{on}} & 2r_{\text{on}} & 0 \\ 0 & 2r_{\text{off}} & -2r_{\text{off}} - r_{\text{on}} & r_{\text{on}} \\ 0 & 0 & 3r_{\text{off}} & -3r_{\text{off}} \end{bmatrix}$;
- For 4 bonds: $Q = \begin{bmatrix} 0 & 0 & 0 & 0 & 0 \\ r_{\text{off}} & -r_{\text{off}} - 3r_{\text{on}} & 3r_{\text{on}} & 0 & 0 \\ 0 & 2r_{\text{off}} & -2r_{\text{off}} - 2r_{\text{on}} & 2r_{\text{on}} & 0 \\ 0 & 0 & 3r_{\text{off}} & -3r_{\text{off}} - r_{\text{on}} & r_{\text{on}} \\ 0 & 0 & 0 & 4r_{\text{off}} & -4r_{\text{off}} \end{bmatrix}$;
- For 5 bonds:

$$Q = \begin{bmatrix} 0 & 0 & 0 & 0 & 0 & 0 \\ r_{\text{off}} & -r_{\text{off}} - 4r_{\text{on}} & 4r_{\text{on}} & 0 & 0 & 0 \\ 0 & 2r_{\text{off}} & -2r_{\text{off}} - 3r_{\text{on}} & 3r_{\text{on}} & 0 & 0 \\ 0 & 0 & 3r_{\text{off}} & -3r_{\text{off}} - 2r_{\text{on}} & 2r_{\text{on}} & 0 \\ 0 & 0 & 0 & 4r_{\text{off}} & -4r_{\text{off}} - r_{\text{on}} & r_{\text{on}} \\ 0 & 0 & 0 & 0 & 5r_{\text{off}} & -5r_{\text{off}} \end{bmatrix}.$$

Using the Markov theory, it is possible to calculate the average time to reach any j from a starting state i (t_{ij}), using the recursive formula (10):

$$t_{ij} = \begin{cases} 0 & \text{if } i = j \\ -\dfrac{1}{q_{ii}} + \sum\limits_{k \neq j} -\dfrac{q_{ik}}{q_{ii}} t_{kj} & \text{if } i \neq j, \end{cases} \quad \text{(Eq. S3)}$$

Where q_{xy} are the matrix element in row x and column y. In this formula, the time needed to transition from the state i to j is the sum of:

- The average time spent by the system in state *i* before transitioning to any other state: $\frac{1}{-q_{ii}}$.
- The time that it takes the system to reach state *j* from any other state the system can transition to (t_{kj}) weighted by the probability to make that transition ($\frac{q_{ik}}{-q_{ii}}$). This is the case because the transition matrix is constructed so that the sum of each row equals 0, i.e. $q_{ii} + \sum_{j \neq i} q_{ij} = 0$.

In our experiments, we only consider particles that bind the surface during the observation time and assume that at arrival only one bond is present. We thus limit our calculations to the average time to reach release (0 bonds) from a single bond, i.e., t_{10} and obtain equation 4 in the main text. This time depends on the maximum number of bonds possible (see section 11 "Probability of establishing multiple bonds with end-grafted heparin"), which in turn depends on the heparin concentration and possibly its conformation which may or may not allow engagement with the viral capsomers.
Solving the previous equation, we obtain:

- For 2 bonds: $t_1^{(2)} = {r_{\text{on}} + 2r_{\text{off}}}\big/{2r_{\text{off}}^2}$
- For 3 bonds: $t_1^{(3)} = {3r_{\text{off}}^2 + 3r_{\text{off}}r_{\text{on}} + r_{\text{on}}^2}\big/{3r_{\text{off}}^3}$
- For 4 bonds: $t_1^{(4)} = {4r_{\text{off}}^3 + 6r_{\text{off}}^2 r_{\text{on}} + 4r_{\text{off}}r_{\text{on}}^2 + r_{\text{on}}^3}\big/{4k_{\text{off}}^4}$
- For 5 bonds: $t_1^{(5)} = {5r_{\text{off}}^4 + 10r_{\text{off}}^3 r_{\text{on}} + 10r_{\text{off}}^2 r_{\text{on}}^2 + 5r_{\text{off}}r_{\text{on}}^3 + r_{\text{on}}^4}\big/{5r_{\text{off}}^5}$

Note that in the trivial case of only a single bond allowed, the transition matrix is:

$$\begin{bmatrix} 0 & 0 \\ r_{\text{off}} & -r_{\text{off}} \end{bmatrix}$$

Thus $t_1 = \frac{1}{r_{\text{off}}}$ or, as expected, the mean release time is reciprocal of the dissociation rate.

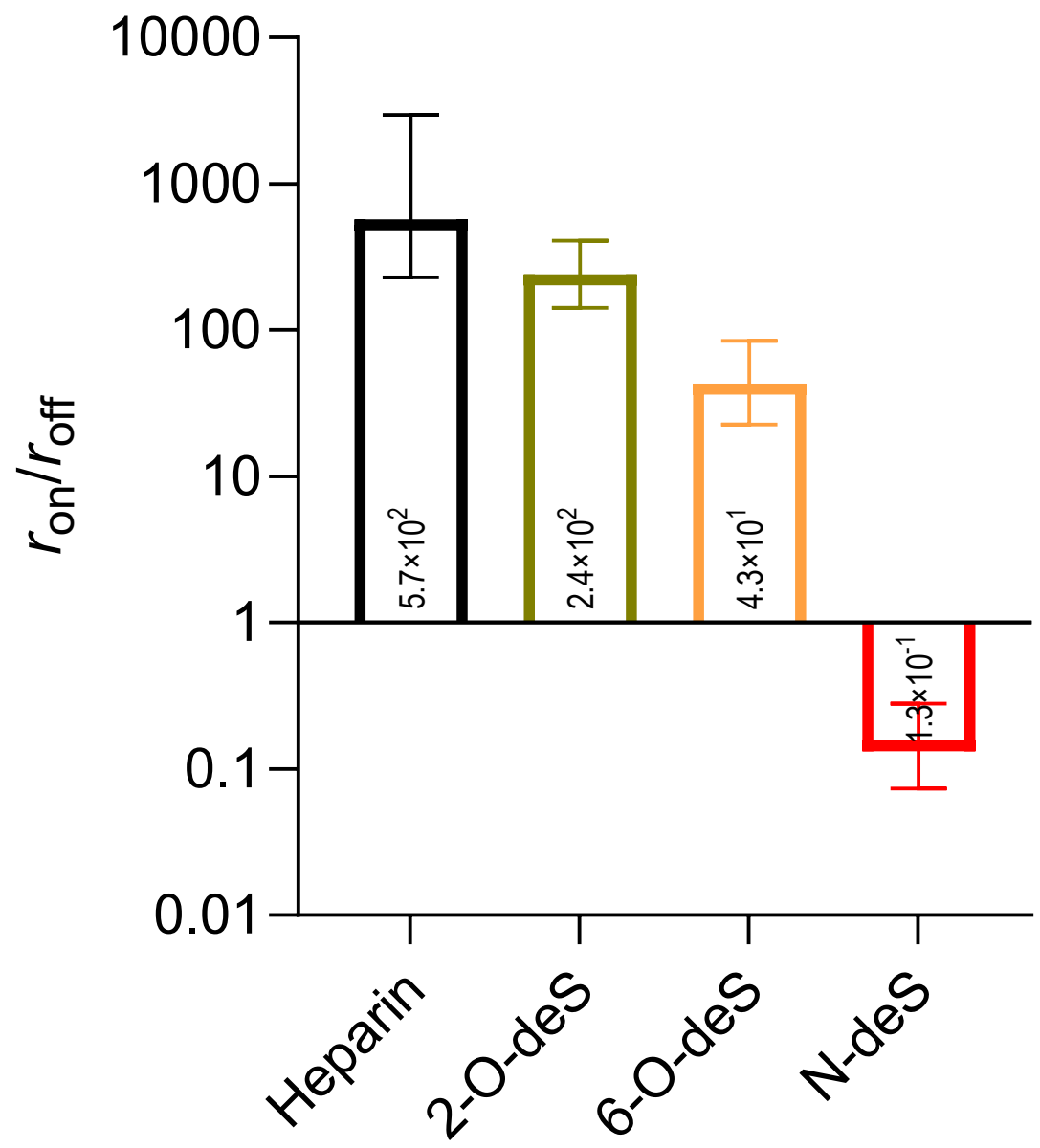

Figure S6. Ratio between the association and dissociation rates used in the Markov model for multivalent binding and derived from the AFM-SMFS reported in reference 1 and Table S4. The error bars indicate the maximum and minimum values obtained from the uncertainty interval of the AFM-SMFS data.

11. Probability of establishing multiple bonds with end-grafted heparin

The height of both the PEG and heparin layers depends on the polymer conformation and characteristics. We first consider PEG, as it is the most abundant molecule on the surface and as it acts as a spacer between the HPV16 particle and the surface.

The conformation assumed by a grafted polymer depends on the grafting coverage. If very sparse, i.e., if the polymer Flory radius (R_F) is much smaller than the separation between chain (d), it assumes a mushroom conformation. Otherwise, if $R_F >> d$, neighboring chains interact and extend away from the surface forming a brush.

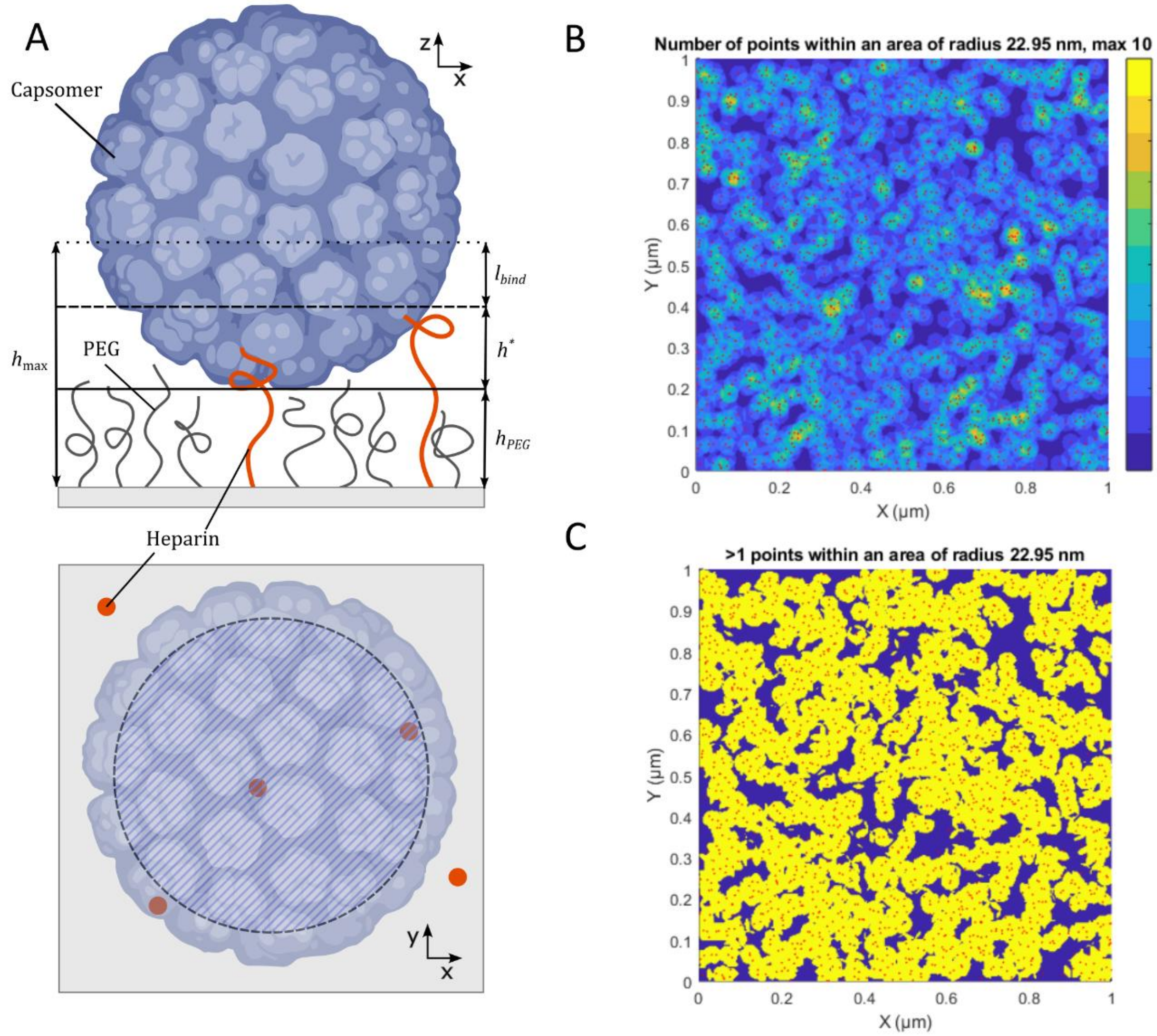

Figure S7. (A) Side (top) and top-down (bottom) representation of an HPV16 particle (in blue) bound to end-grafted heparin (in orange). The PEG brush prevents the virion to reach the surface (solid black line, h_{PEG}). The heparin chains can stretch up to h_{max} (dotted black line). However, binding requires at least 4 disaccharides, reducing the maximum binding height by l_{bind} (dashed black line). The shaded blue area indicates the area in which binding is possible, while the blue circles show the size of a virion. (B) Simulated distribution of heparin chains (red dots) superimposed to a heat map showing the maximum number of bonds a HPV16 particle can form. Simulation parameters: grafting coverage: $\sigma=1.7\times10^{11}$ cm^{-2}; virion contact area: 1655 nm^2, representative of N-deS. (C) Map indicating the portion of surface that can sustain bonds between HPV16 and 2 or more heparin chains (in yellow).

In our case, PEG is grafted onto a streptavidin layer bound to a biotinylated supported lipid bilayer. In these conditions, streptavidin arranges in lattices with footprints between 25 and 34 nm^2 (11). From the SPR experiment, we obtained this footprint to be 24.05 ± 2.25 nm^2

upon exposing SLBs containing 10% biotin to 50 µg/mL bulk concentration of streptavidin; this corresponds to a dense monolayer. At full coverage, each streptavidin has two biotinylated PEG molecules, and the grafting coverage is $\sigma_{PEG} = 0.07\, nm^{-2}$ or an interchain distance of ~4 nm. This is comparable with the Flory radius of 3.4k PEG used in our experiments ($\mathrm{R_F^{PEG}} = 4.7$ nm, as calculated below). The PEG is thus in a transition configuration between mushroom and brush. For simplicity, we consider the two extreme cases of mushroom and brush configuration.

For an isolated polymer, we consider the Flory theory for real polymer chain, i.e., we consider steric hindrance between monomers. The polymer conformation is determined by the balance between the interaction between single monomers that does not allow the chain to collapse on itself and the tendency of the polymer to assume a coiled low entropy configuration, which opposes polymer stretching. This can be expressed as:

$$E_{\mathrm{chain}} = k_B T \left(v \frac{N^2}{R^3} + \frac{R^2}{N b^2} \right) \qquad \text{(Eq. S6)}$$

where $v = b^2 d$ is the exclusion volume of a single polymer segment, b and d are the segment Kuhn length and diameter, respectively, $N = L_{\mathrm{c}}/\mathrm{b}$ is the number of segments composing the polymer chain of total length L_{c} and R is the end-to-end distance of the polymer chain, which we take as the measure of the polymer size. In the case of PEG, this results in the free energy landscape shown in blue in Fig. S8, and an equilibrium R, which is the polymer Flory radius of 4.7 nm.

In the case of a brush configuration, the brush height is dependent on the grafting coverage, and the free energy landscape can be calculated using the Alexander-de Gennes model:

$$E_{\mathrm{brush}} = k_B T \left(v N \frac{N\sigma}{R} + \frac{3R^2}{2N b^2} \right) \qquad \text{(Eq. S7)}$$

where we still consider the equilibrium between interaction and entropic contribution but adapt the first term to consider intra-chain interactions. The result of this model in the case of PEG 3.4k is shown in green in Fig. S8A. We can observe that due to the low grafting coverage, the two models yield similar results indicating limited interaction between chains and thus we will consider the mushroom configuration for the rest of our calculation and consider R as the height of the PEG layer.

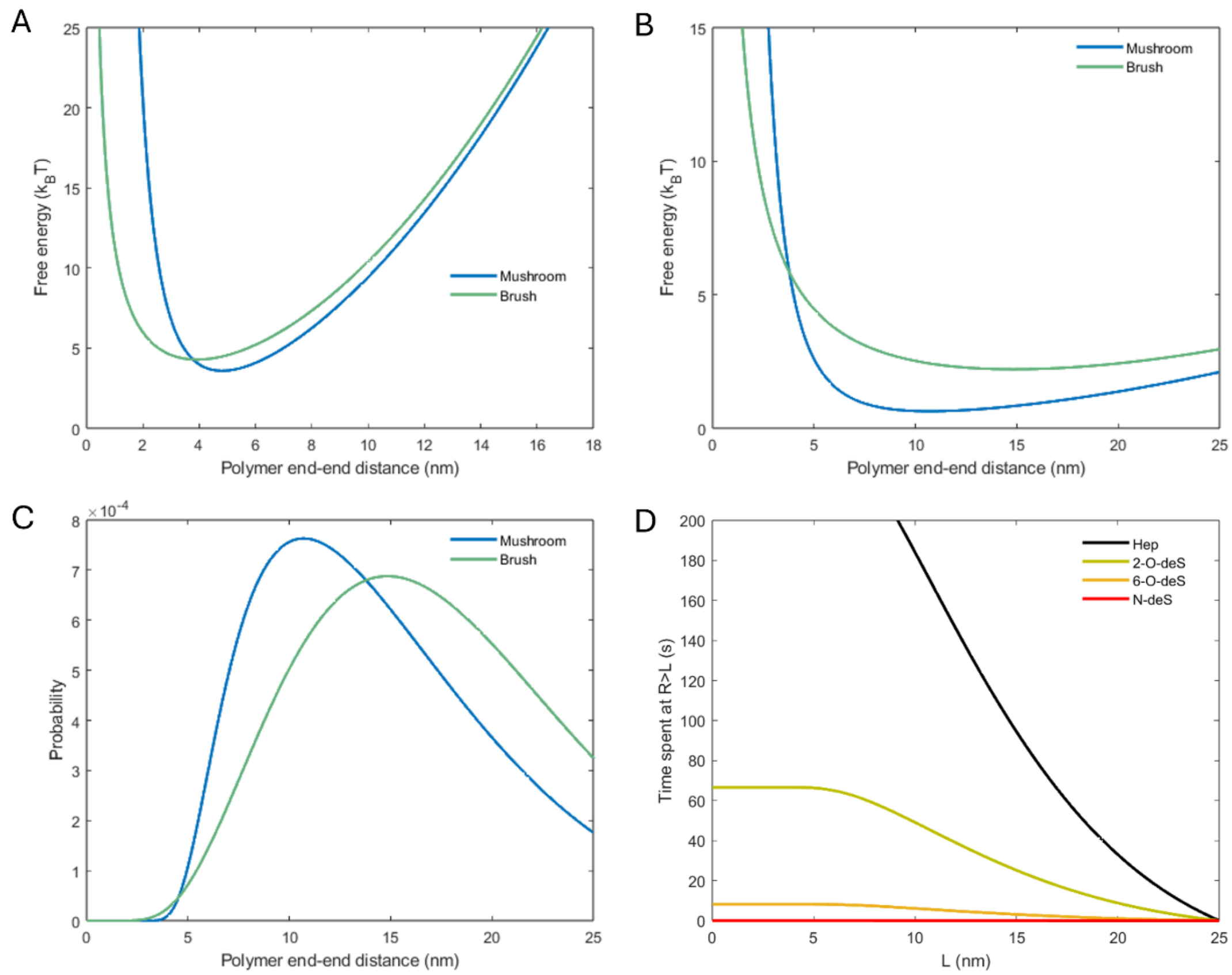

Figure S8. (A-B) Free energy landscape along the elongation path of a heparin chain in the case of a mushroom (blue) or brush (green) configuration for PEG 3.4k (A) and heparin (B). (C) Probability of finding a heparin chain at a given elongation calculated using Boltzmann statistics from B. Mushroom configuration in blue and brush in green. (D) Time spent on average at elongations larger than L_{ext} before the virion detaches from the surface, i.e. the integral of the elongation probability from L_{ext} to the contour length multiplied by $1/k_{off}$.

We can perform the same analysis for heparin (b = 9.0 nm (1,12), d = 0.5 nm, L_c = 25 nm). The results are shown in Fig. S8B. As for PEG, the two configurations result in very similar energy landscape and thus we consider the mushroom configuration in this case as well. Compared to PEG, the high charge carried by heparin results in a stiffer chain with equilibrium distance of 8 nm, and a much lower energy barrier towards extension.

Since a heparin chain will dynamically extend and contract due to thermal fluctuation, the chain will reach distances much larger than the equilibrium position. We thus consider as maximum extensions the length that the heparin chain assumes for a period comparable to $t_{on} = 1/r_{on}$, i.e., the time needed to establish a bond, over the time that the virion is present on the surface, which is $1/r_{off}$.

The probability of a chain to be found at a given elongation or larger can be calculated from the free energy landscape using Boltzmann's statistics:

$$P(L > h) \approx \int_{h}^{L_c} Ce^{-\frac{\Delta E(\mathrm{x})}{k_B T}} dx, \qquad \text{(Eq. S8)}$$

where $C = 1/\int_0^{L_c} \exp\left(\frac{\Delta E(x)}{k_B T}\right) dx$ is a normalisation constant so that $P(L > 0) = 1$.

We can calculate the time spent at a given elongation or larger as the probability of the particle to be in that state multiplied by $1/k_{\mathrm{off}}$, $\tau(L > h) = \frac{P(L>h)}{k_{\mathrm{off}}}$. While this is not a measure of the continuous time spent in this configuration, we interpret it as the polymer fluctuating around this conformation and eventually resulting in the creation of a bond. Numerically inverting this formula results in the maximum length of a heparin chain, h_{MAX}.

In addition, it has been shown that HPV16 binding to HS requires at least 4 disaccharides monomers (13,14). In first approximation, we can incorporate this minimum binding motif in our model by reducing the maximum elongation of the heparin chain by the length of 4 monomers, 4 nm.

Thus, the maximum height at which the heparin can bind the particle on top of the PEG layer, h^*, is thus equal to the maximum heparin length (h_{MAX}) minus the height of the PEG layer and the minimum binding motif. The results depend on the kinetic parameters of the HPV16-heparin interaction and thus vary between heparin sulfation (Table S5).

Table S5: Dissociation constants determined by AFM-SMFS (3), maximum elongation, interaction area, GAG grafting coverage and probability for a virion to form less than 2 bonds with the surface, i.e., to land on an area in which less than two heparin chains are present. The values between square brackets indicate the maximum and minimum probability calculated according to the error in the determination of the grafting coverage.

	$\boldsymbol{k_{\mathrm{off,1}}}$ ($\mathrm{s^{-1}}$)	$\boldsymbol{h^*}$ (nm)	$\boldsymbol{A}$ ($\mathrm{nm^2}$)	$\boldsymbol{\sigma_{\mathrm{hep}}}$ ($10^{-3}\ \mathrm{nm^{-2}}$)	$\boldsymbol{P(n \le 1)}$ (%)
**Heparin**	0.004 ± 0.003	16.3	1726	1.1 ± 0.3	43.4 [34.5, 59.9]
**2-O-deS**	0.015 ± 0.004	16.3	1726	1.3 ± 0.3	34.1 [23.8, 48.5]
**6-O-deS**	0.12 ± 0.04	16.2	1726	1.5 ± 0.9	27.0 [8.2, 72.3]

**N-deS**	12.2 ± 5.2	15.1	1655	1.6 ± 0.4	25.8 [15.7, 41.0]
**Fully stretched N-deS**	-	16.3	1726	1.6 ± 0.4	23.8 [14.1, 38.7]
**Flory radius N-deS**	-	0.45	70.05	1.6 ± 0.4	99.4 [99.1, 99.7]

In the case of N-deS, $h^{*} = 15.1$ nm. This results in a contact area of 1.6×10^3 nm^2, and, considering a grafting coverage of 1.6×10^3 nm^{-2}, a probability of a particle to attach in a spot with less than two heparins, and thus not being able to form multivalent bonds is 25.8%.

Notably, in this theory, the energy penalty does not diverge when the end-to-end length of the polymer approaches its contour length, the maximum length obtainable if the chain is completely stretched. Without appropriate boundary conditions, this could lead to unphysical stretching beyond the contour length and likely underestimates the energy cost for the polymer to reach highly stretched configurations. As a result, we might be overestimating the interaction volume of a virus particle bound to the surface, and ultimately the r_{on}.

Finally, we check the validity of the assumption that heparin binding sites are always accessible for HV16 binding. We can estimate the rate at which the heparin chain explores possible configurations around the equilibrium one to be of the order of the polymer relaxation time. In the experimental conditions, we consider the heparin as a non-tangled chain in a good solvent, being a hydrophilic molecule in water, and thus employ the Zimm model(15) for polymer dynamics:

$$\tau_1 \approx \frac{1}{2\sqrt{3\pi}} \frac{\eta b^3 N^{3/2}}{k_B T} = 133 \text{ ns}, \qquad \text{(Eq. S9)}$$

where $\eta = 1$ mPa $\cdot$ s is the dynamic viscosity of water. This value is several orders of magnitude lower than both t_{on} and $1/k_{\text{off}}$, indicating that heparin binding epitopes are indeed available at the timescales of the interaction with HPV16.

12. Comparison between the Markov-multivalency model and experimental results

Figure S9 shows the dissociation curves and rates obtained by equilibrium fluctuation analysis when targeting short-lived initial interactions. Particles were imaged using the

same setup as in Fig. 2, but at 1 fps for 15 minutes to observe bond lifetimes between 10 seconds and 5 minutes. This approach allowed improved resolution of the detachment of HPV16 particles bound by a single bond to 2-O-deS and 6-O-deS heparin. The resulting dissociation curves (Fig. S9A) were compared with those predicted by the Markov model, showing excellent qualitative agreement. The curves were fitted with a two-phase exponential decay with vertical offset ($y = A + Bexp(-k_s t) + Cexp(-k_f t)$, with $A + B + C = 1$). This fit was chosen to distinguish between monovalent interactions that are expected to have a fast dissociation rate in the range of seconds to minutes for 2-O- and 6-O-deS (k_f), and the more stable divalent bonds (k_s). The vertical offset considers higher multivalency interactions, which, according to the proposed model, have dissociation rates much longer than the experimental observation time and thus appear as irreversibly attached to the surface.

All samples, including HA and N-deS (data not shown), show a fast initial dissociation within a few seconds, possibly due to weak non-specific interactions. This component was excluded from the analysis by considering only detachment times longer than 10 s. The fast components for 2-O-deS and 6-O-deS show good quantitative agreement with the detachment rates measured for single bonds using AFM-SMFS experiments (Fig. S9B), in line with the idea that the fast dissociation component can be ascribed to the monovalent interaction. Heparin also shows a fast-detaching population; however, this fraction is smaller than that observed for the desulfated variants and is likely due to residual non-specific interactions. The detachment rate of the slow components is much longer than the measurement time and is therefore poorly constrained by the fitting. However, in the case of parental heparin it is compatible with the value expected for monovalent bonds.

Figure S10 shows the effect of increasing heparin grafting density on the predicted detachment curves according to the Markov model presented in this work. As expected, the increased binder concentration results in a higher proportion of multivalent bonds that do not rupture within the experimental time window, thereby increasing the fraction of irreversibly bound particles.

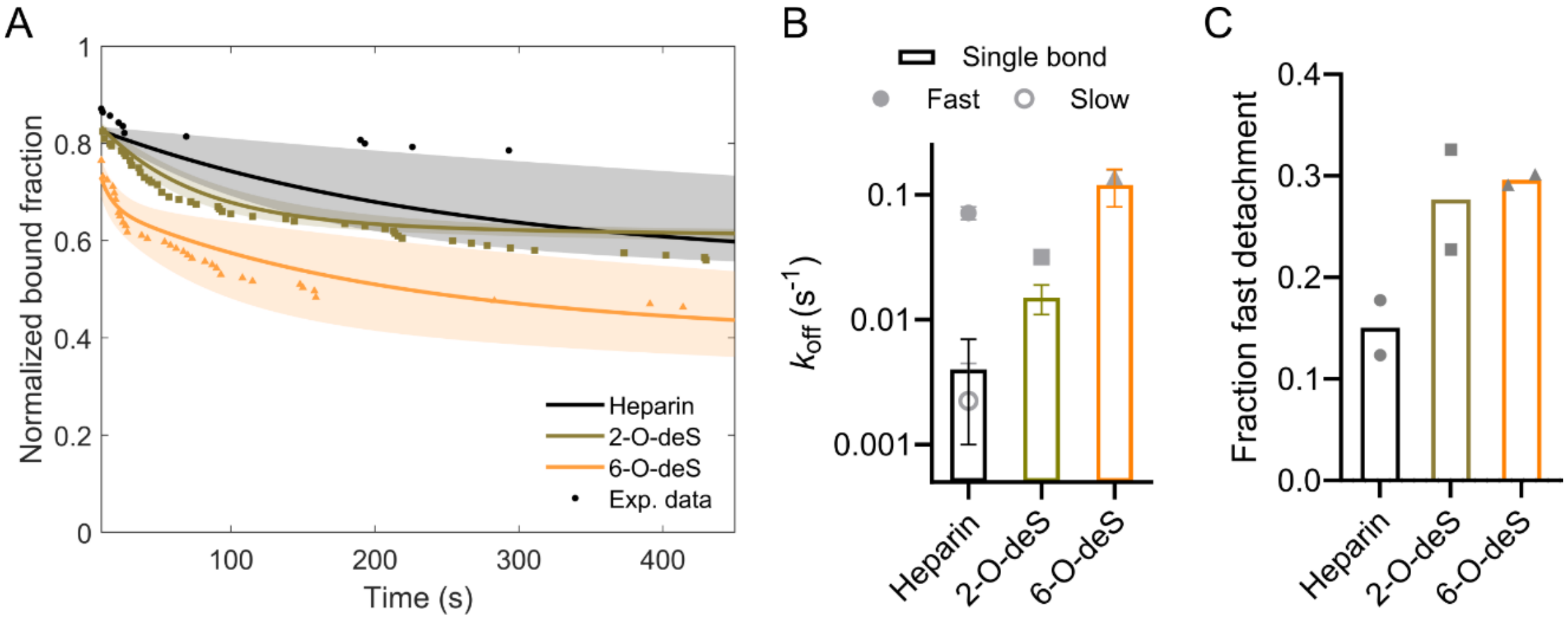

Figure S9. (A) Representative bond survival probability as a function of time obtained by equilibrium fluctuation analysis acquired at 1 fps for 15 minutes (symbols), compared to the Markov-multivalency model prediction in the same acquisition conditions (solid lines). The shaded area indicates the value obtained using the upper and lower limits of the bond lifetime as measured by AFM-SMFS (Table S4). (B) Mean dissociation rate constant of the short-lived particle population obtained by fitting the experimental data with a double exponential decay function plus offset (solid symbols), compared to the single bond dissociation rate measured by AFM-SMFS (Table S4). Slow dissociation rate constant for parental heparin is also included as a hollow circle. All obtained from 2 independent experiments and the error bars indicate the standard error of the mean. Error bars on the bar plot indicate the confidence interval of the AFM-SMFS measurements. (C) Fraction of particles that display the fast dissociation rate shown in B. All data were acquired from 2 independent experiments (grey symbols), the bar height indicated the mean.

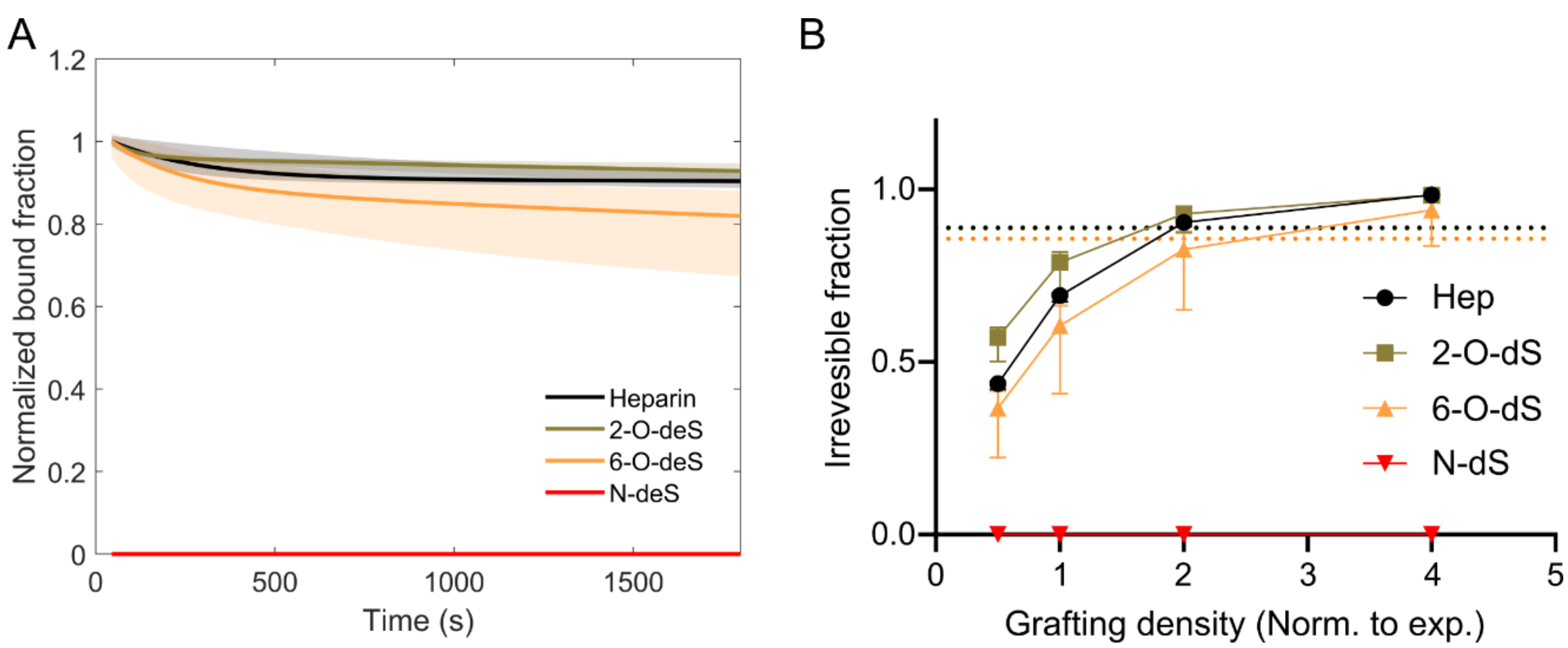

Figure S10. (A) Markov–multivalency model prediction of the bond survival probability as a function of time for a GAG grafting density twice that used experimentally (2.2-3.2×10^{3} nm^{-2}). The solid line was obtained using the mean value of the bond lifetime, while shaded areas represent the upper and lower limits derived from AFM-SMFS measurements (Table S4). (B) Predicted irreversible fraction, defined as the fraction of the total number of bound particles that persist on the surface for times much longer than the experimental

observation period, as a function of GAG grafting density (normalized to the grafting density used experimentally). The dashed horizontal lines indicate the irreversible fraction measured experimentally using a single-exponential fit with offset. Error bars indicate the maximum and minimum values expected based on the confidence interval of the AFM-SMFS measurements (Table S4).

13. HPV16 diffusion on heparin film

Figures S9 and S10 illustrate the main results for tracking of HPV16 on parental heparin and N-deS heparin at two PEG:GAG densities. Fig. S13 provides the estimated diffusion coefficient for particles classified as confined, as a function of the signal-to-noise ratio, illustrating that no diffusion is observed in our tracking experiments.

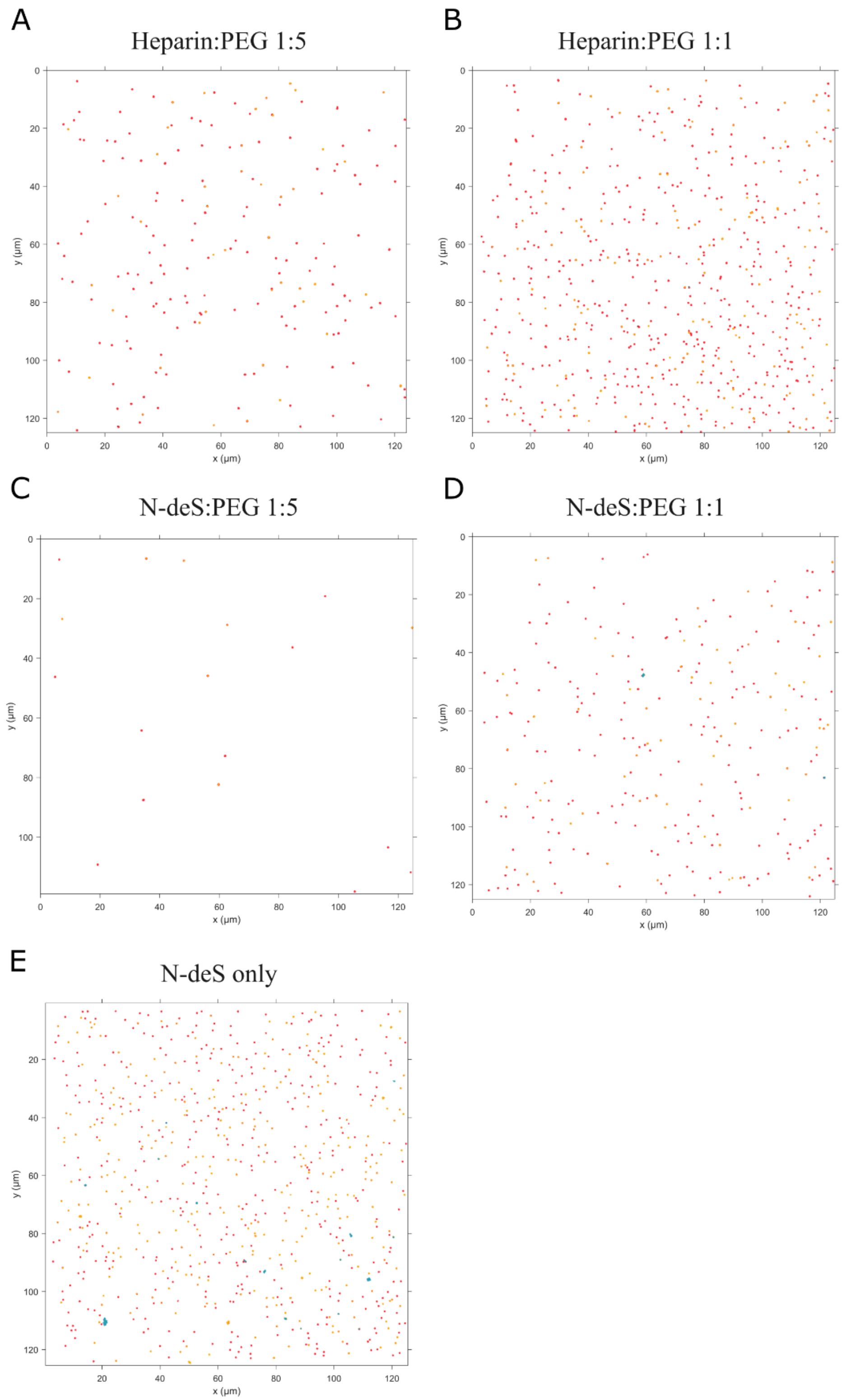

Figure S11. Representative tracks of HPV16 virus-like particles on heparin-grafted surfaces – (A) parental heparin and PEG in molar ratio 1:5, (B) parental heparin and PEG at a 1:1 molar ratio. (C) N-deS heparin and PEG in molar ratio 1:5, (D) N-deS heparin and

PEG at a 1:1 molar ratio. (E) Pure N-deS heparin. Tracks have been segmented according to the mobility characteristics. Red: immobile particle; yellow: Confined motion; blue: Free diffusion.

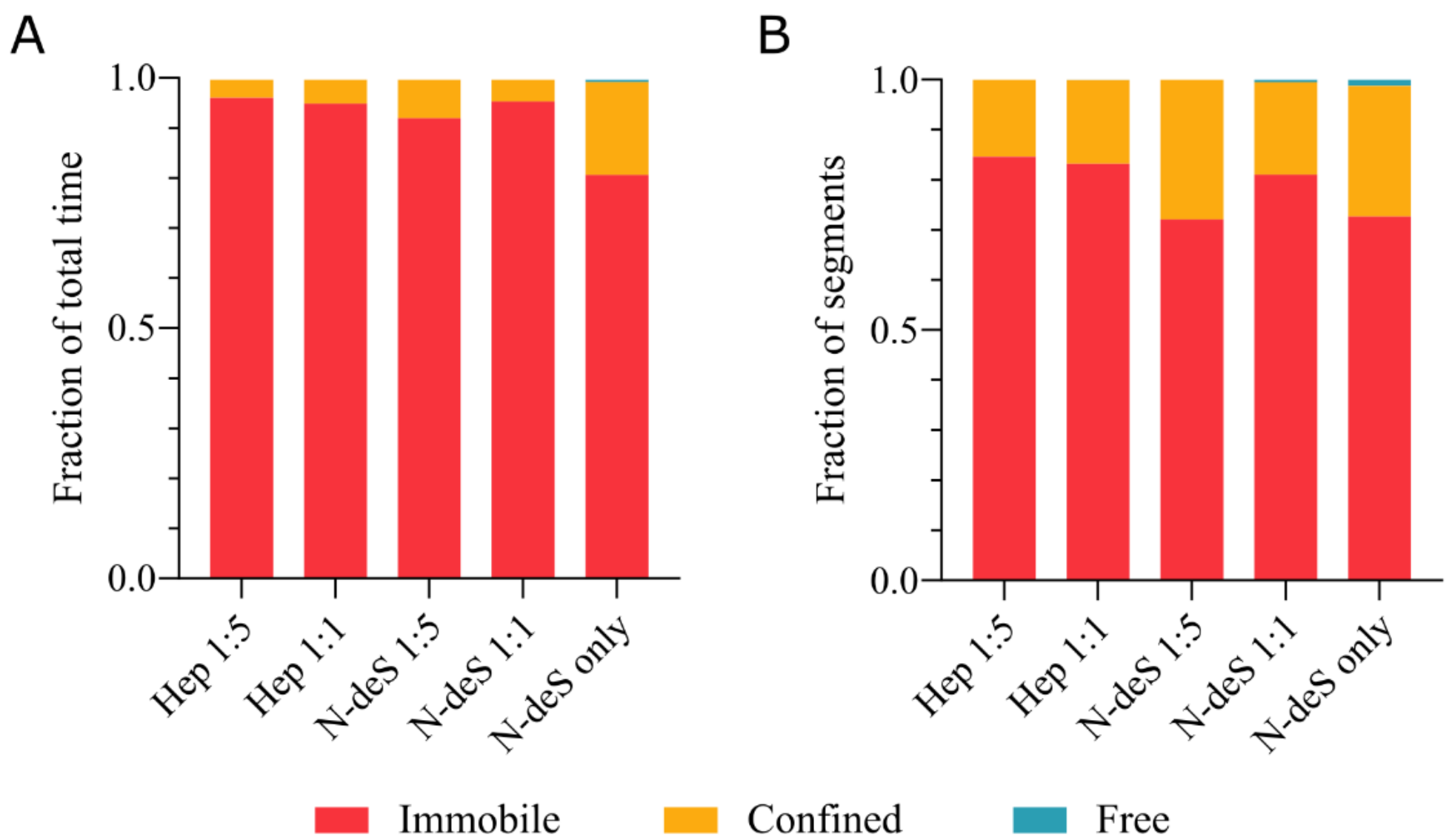

Figure S12. Fraction of the total cumulative time spent (A) or number of segments (B) in the three different motion regimes: immobile particles (red), confined motion (yellow), and free diffusion (blue).

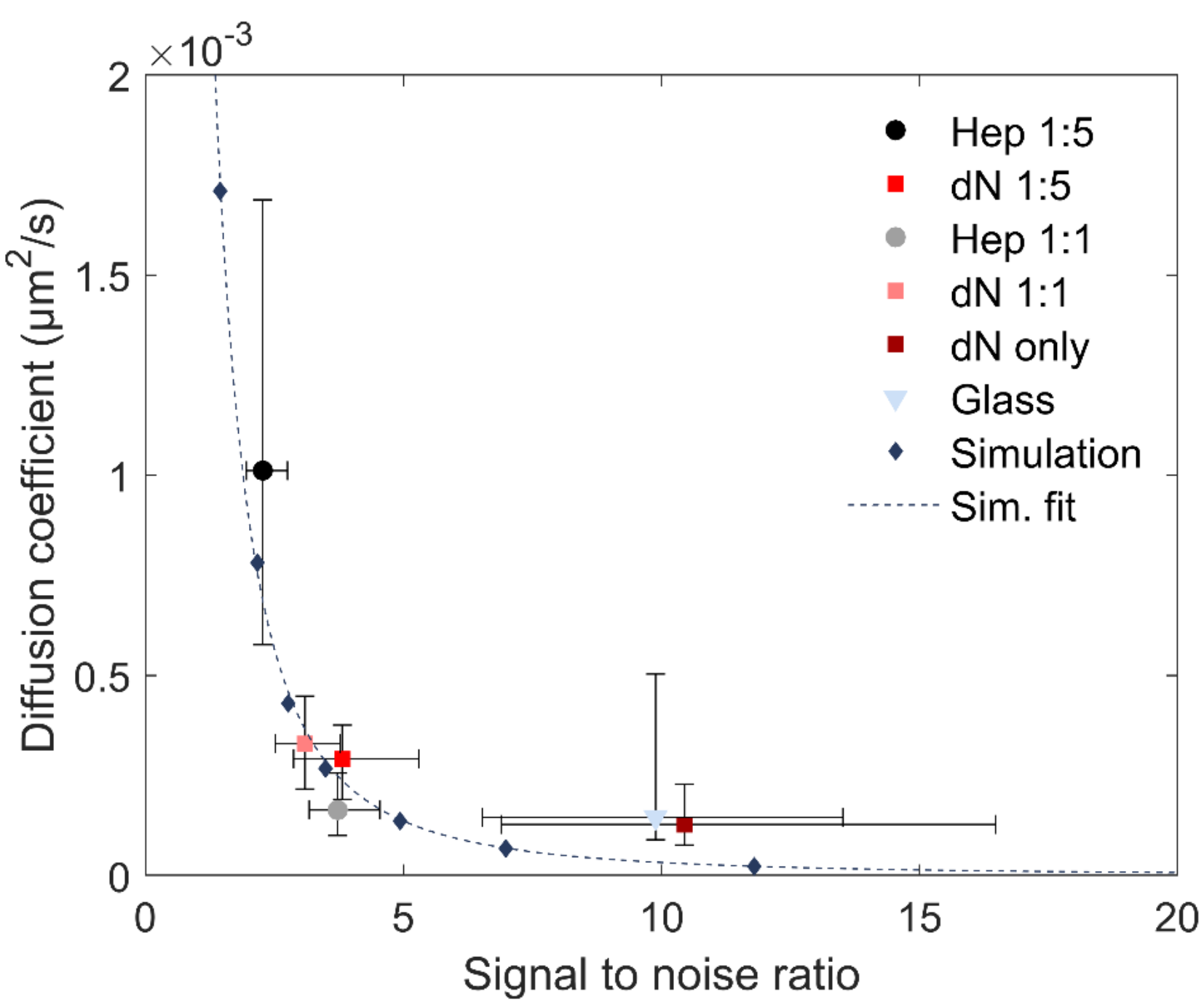

Figure S13. Diffusion coefficient (D) of HPV16 track segments classified as confined, as function of the signal to noise ratio of the images. The filled dots indicate the median value for each condition (Black: Hep:PEG::1:5; Grey: Hep:PEG::1:1; Red: N-deS:PEG::1:5; Pink: N-deS:PEG::1:1; Dark red: N-deS). Particles stuck on glass, in teal, were tracked as an immobile control. The error bars indicate the first and last quartile. The blue diamonds show the diffusion coefficient obtained using our algorithm for synthetic timelapses of immobile particles. The dashed line is the best power fit: $D = 0.0037 \cdot \mathrm{SNR}^{-2.05}$.

14. Simulation of TIRF images of immobile particles

We determined the localization precision limit of our tracking algorithm by generating synthetic timelapses of immobile particles with added acquisition noise. 150 particles were randomly positioned on a 130×130 μm^2 area and the Airy disk was approximated using a 2D Gaussians with standard deviation 220 nm, slightly larger than the of the resolution-limit: $\sigma_{\mathrm{spot}} = 0.45\lambda_{\mathrm{em}}/\mathrm{NA} = 181$ nm, where $\lambda_{\mathrm{em}} = 600$ nm is the emission wavelength and NA = 1.49 is the numerical aperture of the objective (16). The image was then discretized in 704x704 pixels to resemble the experimental images. The acquisition noise and background were matched to the experimental conditions by adding a uniform background of 145 and sum a normally distributed noise with standard deviation 8.3. The signal to noise ratio (SNR) was adjusted by changing the intensity of the spots to 20, 30, 40, 50, 70, 100, and 170. For each condition, 100 frames were generated maintaining the spots immobile and generating a new randomized noise pattern at each frame. The signal to noise ratio (SNR) was calculated as described in the Material and Methods in the main text for experimental time-lapses. Representative frames are shown in Fig. S14.

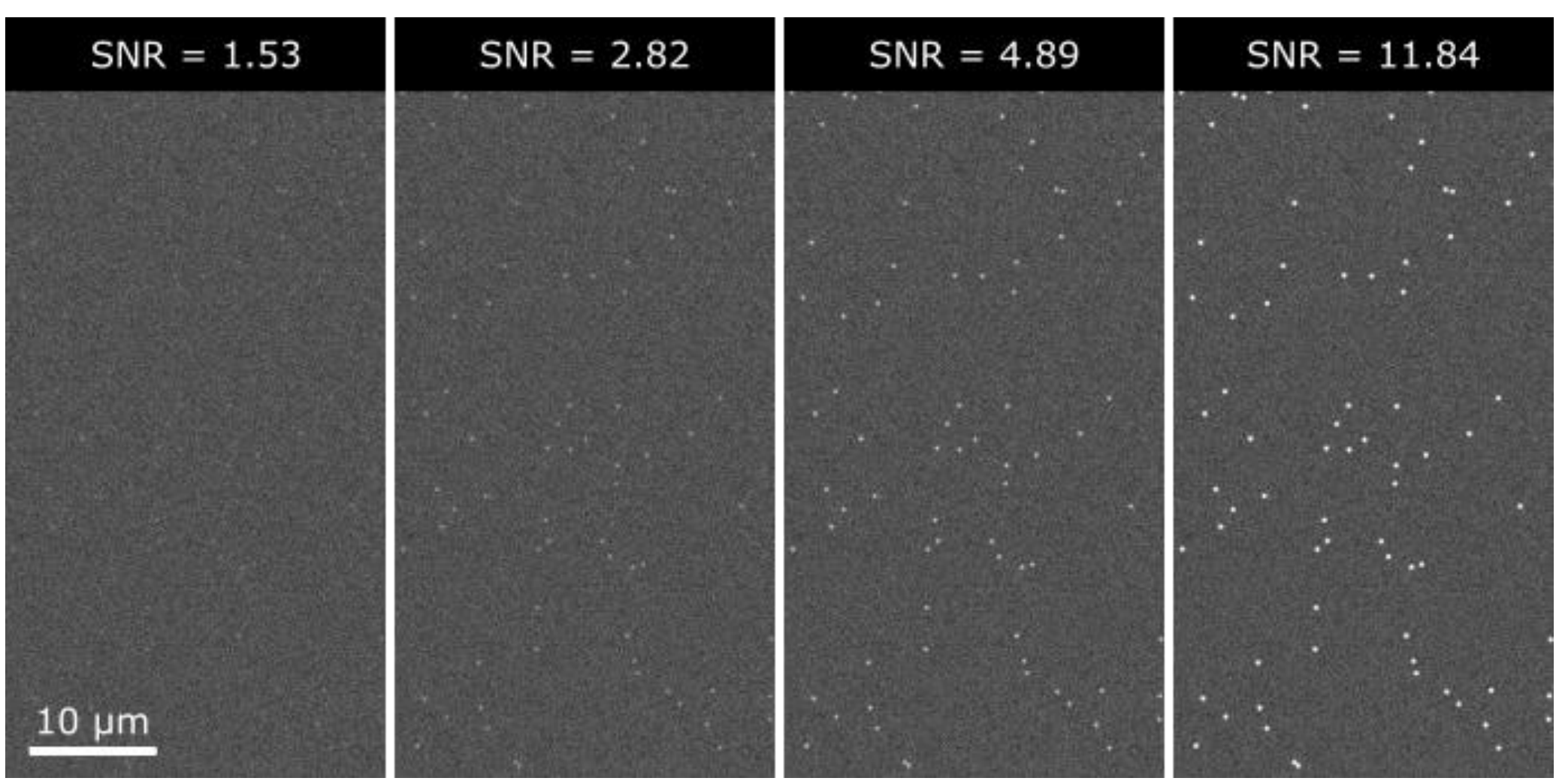

Figure S14. Example frames of the synthetic videos generated to test the localization precision of our particle tracking and analysis algorithm at signal to noise ratio (SNR) of 1.53, 2.82, 4.89, 11.84.

15. Model of multivalency aided diffusion

We modelled the diffusion of particles on surface-grafted binders originating from the establishment of transient multivalent bonds using in-house MATLAB scripts, as described in the Material and Methods in the main text (section Multivalency aided diffusion simulation).

The trajectories obtained from the Brownian dynamics simulations were analysed to compute several parameters:

- *Path length*: Computed as the sum of distances between consecutive particle positions, reflecting the cumulative distance travelled by the particle (Fig. S16A).
- *Hull area and confinement radius*: The area enclosed by the convex hull was calculated as a measure of the spatial extent of the particle's exploration, with an effective radius derived as half the square root of the area inscribed in the hull (Fig. S15A).
- *Maximum displacement*: Determined by calculating the largest distance between points on the convex hull enclosing all trajectory points, providing insight into the extent of particle movement within the simulation space (Fig. S16B).
- *Average number of bonds*: Evaluated by averaging the number of tether bonds formed between the particle and the surface across all recorded timesteps, indicating the extent of tether-particle interactions (Fig. S16C).
- *Track duration*: Calculated as the average time spent by the particle on the surface before detachment, breakage of the bond to all tethers (Fig. S16D).
- *Average particle speed*: The average speed of the particle between each iteration. The speed is calculated as the length of each particle step divided by dt (Fig. S16E).
- *Estimated diffusion coefficient*: Calculated as the hull area divided by the track duration (Fig. S15B). The true diffusion coefficient was also calculated by fitting of the mean square displacement. However, it produced highly noisy data due to the short length of tracks at low affinities and low binder concentration.

Qualitatively, both approaches resulted in similar diffusion coefficients (data not shown).

Additionally, each track was segmented into 4 possible motion types (immobile, confined, free diffusing and direct motion) as described for the analysis of the experimental tracks, and shown in Fig. S15A. The average motion type of the track was estimated as previously described (17). In brief, each motion type was scored by assigning a numerical value: 0: immobile, 1: confined, 2: free, 3: direct. The average motion type for each track was determined by calculating the average of the subtract scores weighted by their duration.

In the case of heparin, particles tethered to the surface can still move around the binding site due to the flexible nature of the heparin chain. In most cases however, the dynamic of the particle Brownian motion is much faster than the binding kinetics (µs to s) which makes the simultaneous simulation of both phenomena computationally challenging. To circumvent this problem, we performed a simulation of the Brownian motion of a particle tethered to the surface by a number of heparin chains between 1 and 100. The dynamics of a tethered spherical particle undergoing Brownian motion were simulated using an overdamped Brownian dynamics algorithm. The particle was modelled as a sphere of radius 25 nm initially suspended 10 nm above an impenetrable plane. The tether attachments were randomly distributed on both the lower hemisphere of the particle and the plane surface. The heparin chain was modelled as a polymer in mushroom configuration, as described in Eq. S6, with the addition of a vertical asymptote for stretching equal to the chain length to prevent unphysical overstretching of the chain: $F_{\mathrm{el}} = k_B T\left(-\frac{3\,N^2 \nu}{\varepsilon^4} + \frac{3\,\varepsilon\,N}{b^2} + \frac{3\,N^2 \nu}{(Nb-\varepsilon)^4}\right)$. Hydrodynamic interactions were included by considering translational and rotational drag, using drag coefficients derived from the Stokes relations: translational drag $\zeta_{trans} = 6\pi\eta R$ and rotational drag $\zeta_{trans} = 8\pi\eta R^3$, with viscosity $\eta =$ 1 mPa·s. Brownian motion was implemented by adding stochastic increments to particle displacement and rotation at each timestep as described in (18). The system evolved over discrete time steps of $dt = 1$ ns, for a total simulation time corresponding to 100,000 steps. Particle trajectories, tether attachment points, particle orientation, forces, and torques were recorded every 10 steps for analysis (Figs. S15 and S16A). The boundary condition was enforced by reflecting the particle off the plane surface, maintaining a minimum separation

corresponding to the particle radius plus the height of the PEG layer. The simulation was repeated 100 times for each condition, randomising the tether position. The distribution of the distance of the particle from the equilibrium position is shown in Fig. S18B. We then calculated the average distance from the equilibrium position as a function of the number of tethers and fitted it to the power law equation: $r_{\mathrm{avg}}(\mathrm{nm}) = 11.52 \times n_{\mathrm{teth}}^{-0.32}$, Fig. S18C. We incorporated the particle Brownian motion in the multivalent diffusion model by increasing the interaction area radius by the average distance from equilibrium according to the number of bounds between the particle and the surface at each simulation step. Summary of the results are presented in the heat maps in Fig. S19.

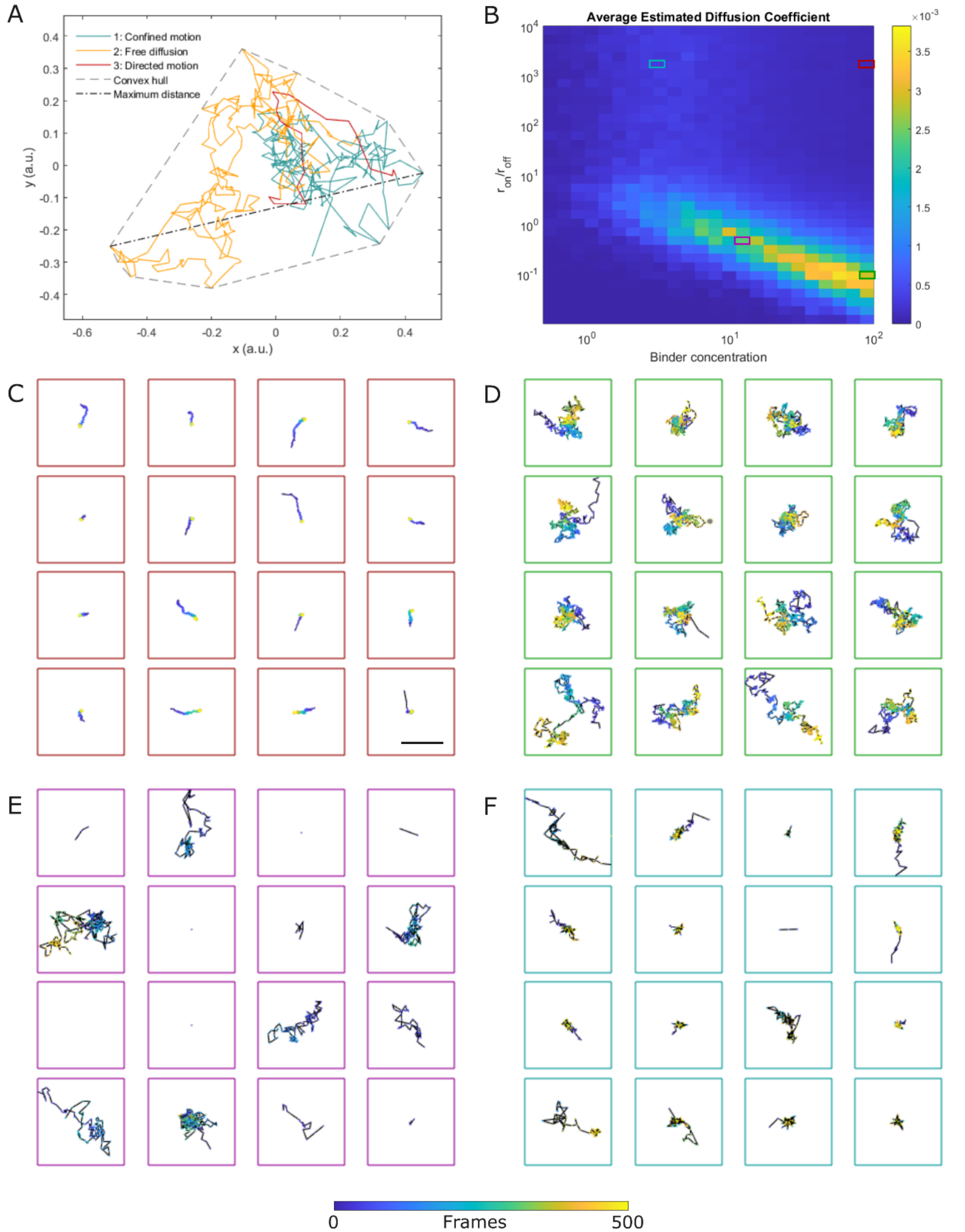

Figure S15. (A) Example of track segmentation showing sections identified as confined diffusion (teal), free diffusion (yellow) and directed motion (red). The track shown has an average motion score of 1.604, indicating mostly free diffusion. The convex hull containing all the track points and used to calculate the area explored by the track is shown as the gray dashed line and the maximum distance between points in the track as black dash dotted

line. (B) Heat map showing the estimated diffusion coefficient for the conditions described in the main text ($r_{\mathrm{on}}/r_{\mathrm{off}}$ = from 0.01 to 104 and binder within the interaction area, n_{bind}, between 0.5 and 100). (C-F) Example tracks using the conditions highlighted by the squares in B for $r_{\mathrm{on}}/r_{\mathrm{off}}$ = 1311, n_{bind}=100 (C); $r_{\mathrm{on}}/r_{\mathrm{off}}$ = 0.115, n_{bind}=100 (D); $r_{\mathrm{on}}/r_{\mathrm{off}}$ = 2.96, n_{bind}=11.0 (D); and $r_{\mathrm{on}}/r_{\mathrm{off}}$ = 1311, n_{bind}=3.65 (F). The scale bar indicates 1 normalized unit of space.

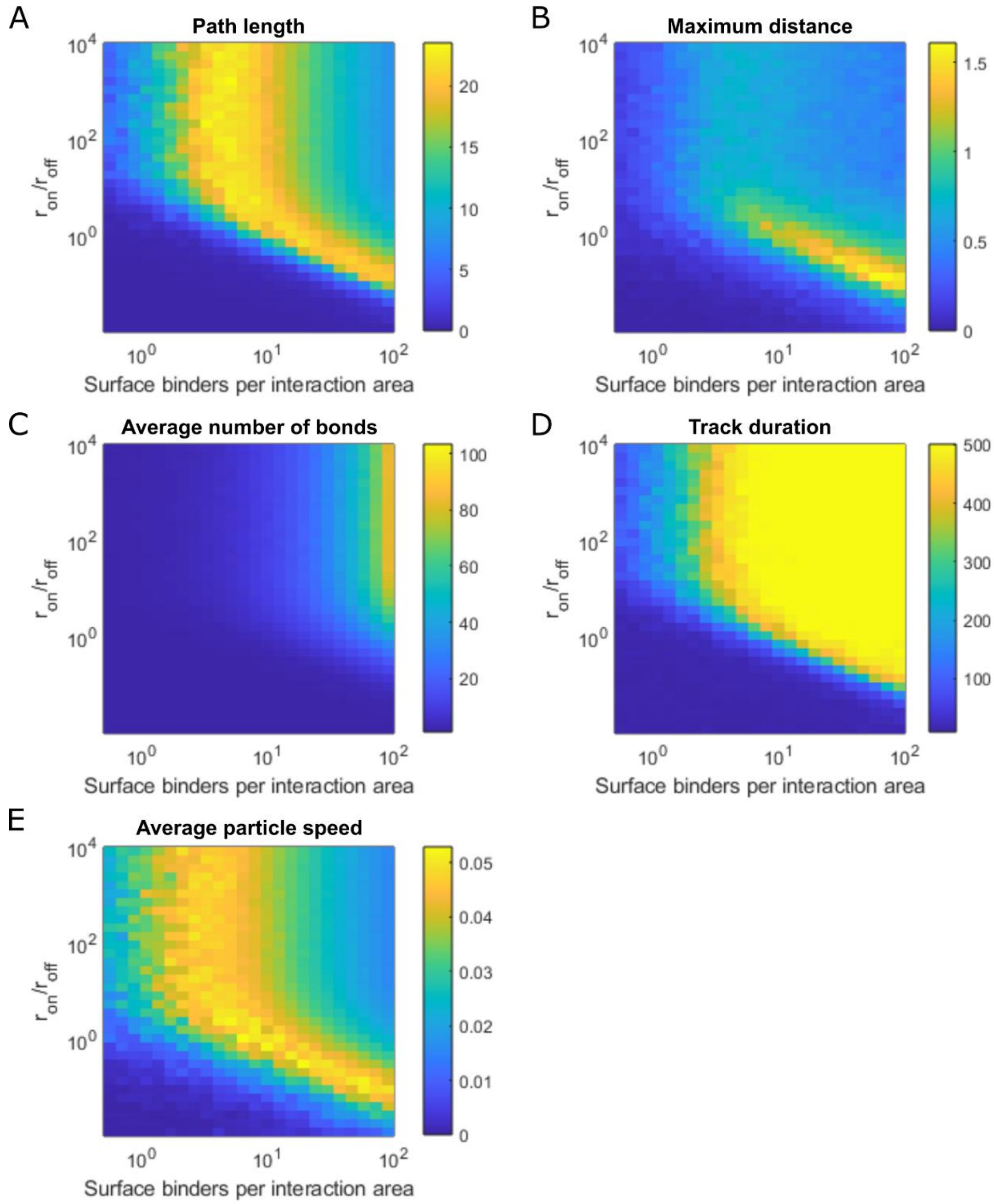

Figure S16. Heat maps showing mobility parameters of motion of a particle due to the creation and rupture of bonds with randomly distributed binders on the surface as a function of the ratio between association and dissociation rate and the average number of surface

binders in contact with the particle. (A) Length of the particle path; (B) Maximum distance between two points of the particle trajectory; (C) Average number of bonds established by the particle to the surface; (D) Average duration of the particle track in simulation steps ($dt = 0.1/k_{\mathrm{off}}$); (E) Average instant speed of the particle. The instant speed is calculated as the length of each step divided by the simulation interval dt.

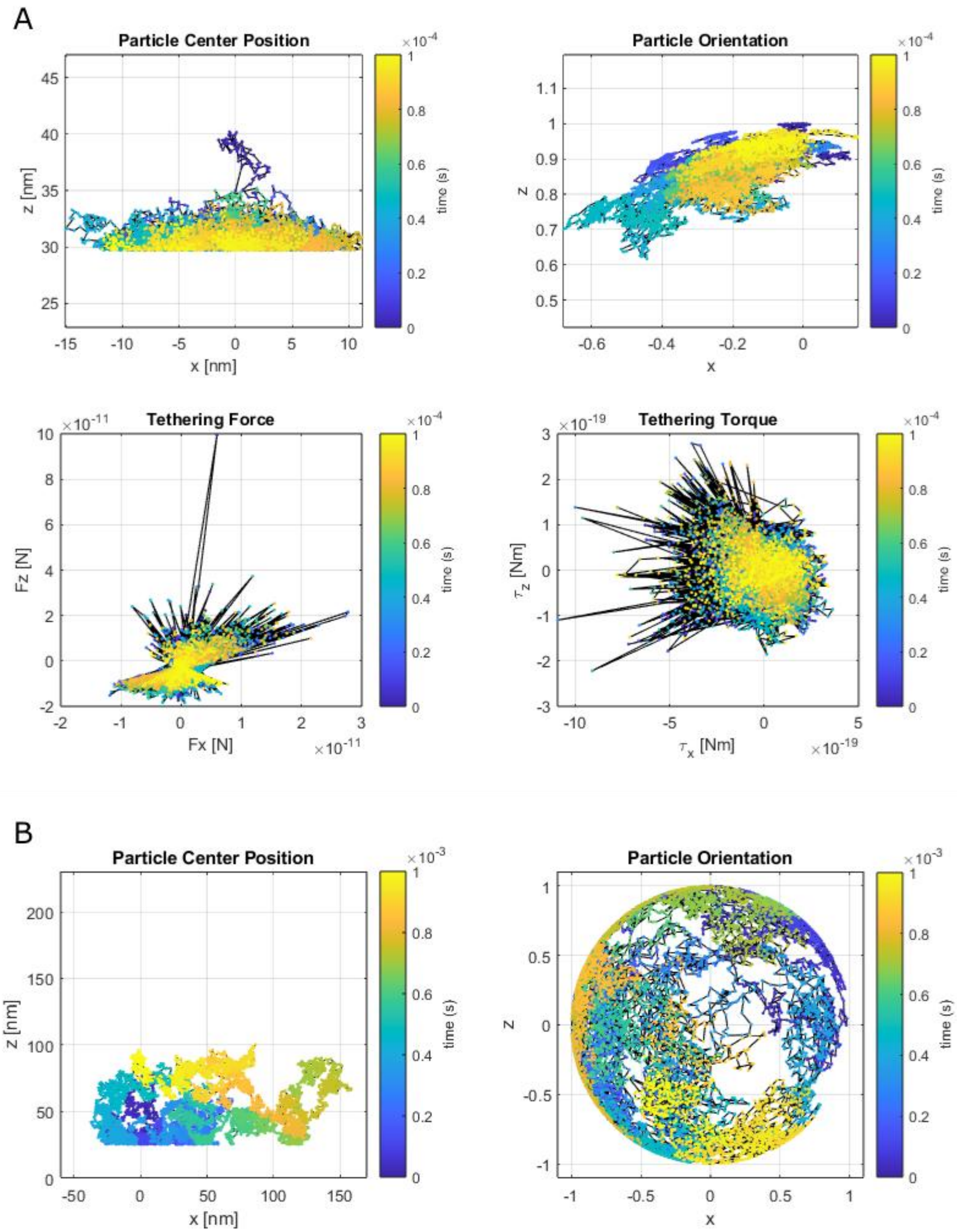

Figure S17. (A) *Top:* Position and orientation of a particle tethered to the surface by 5 heparin chains. *Bottom:* Cumulative force and torque exerted on the same particle by the heparin chains. (B) Position and orientation of a free particle simulated with the same algorithm as in (A). Initial position; $x = 0$ nm, $y = 0$ nm and $z = 40$ nm. The particle orientation is represented by a unit vector with an initial orientation at $t = 0$ s of [0,0,1].

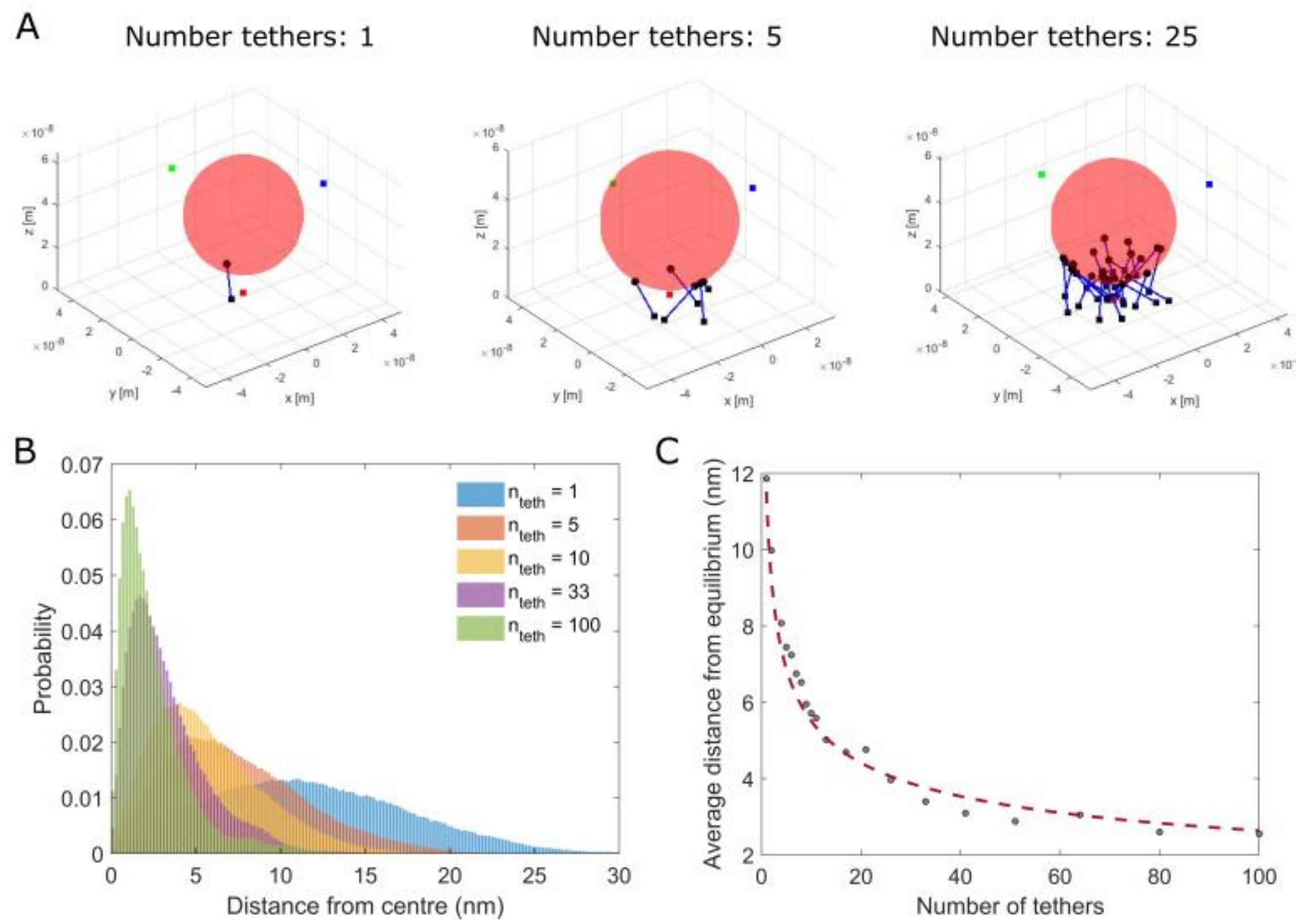

Figure S18. (A) Frames from the simulation of the Brownian motion of an HPV particle (red sphere) tethered to the surface by heparin chains (blue lines). The red, green and blue squares indicate the position of the particle along the three planes. The black dots, the attachment points of the heparin chains to the surface (squares) and the particle (circles). (B) Histogram showing the distribution of the distance from the equilibrium assumed by the HPV particle for several number of tethers (n_{teth}). (C) Mean distance from equilibrium calculated from the distribution in B as a function of the number of tethers (grey dots). Power law fit (red dashed line): $r_{\text{avg}}(\text{nm}) = 11.52 \cdot n_{\text{teth}}^{-0.32}$.

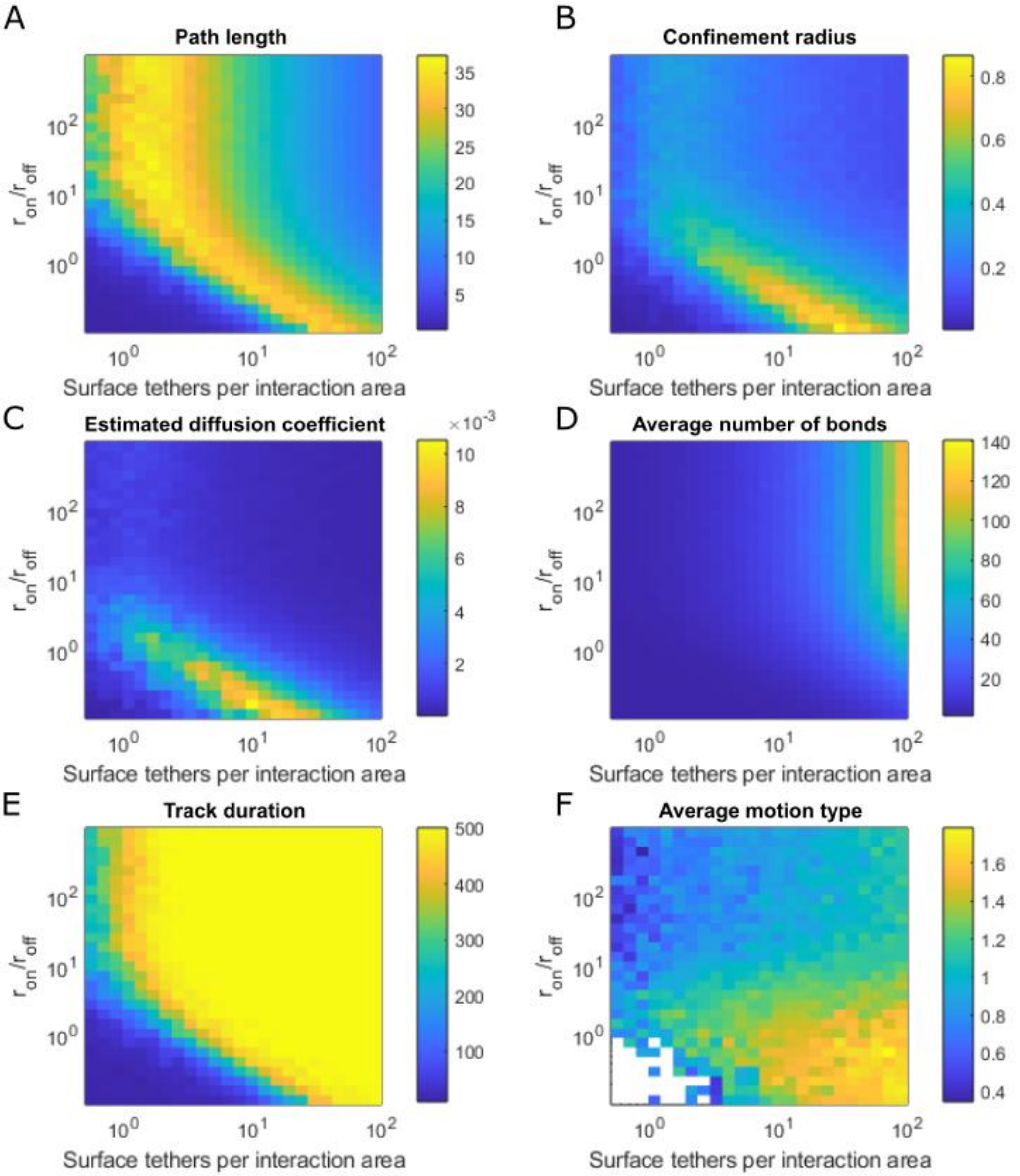

Figure S19. Heat maps showing mobility parameters of motion of a particle due to the creation and rupture of bonds with randomly distributed binders on the surface as a function of the ratio between association and dissociation rate and the average number of surface binders in contact with the particle. The Brownian motion of the tethered particle is considered by increasing the interaction radius by the average radius of the tethered Brownian motion experienced by the particle (Fig. S18B-C). (A) Length of the particle path; (B) Confinement radius calculated as half of the square root of the area of the convex hull calculated from the particle trajectory; (C) Estimated diffusion coefficient calculated as the area of the convex hull divided by the track length; (D) Average number of bonds established by the particle to the surface; (E) Duration of the particle track in simulation steps ($dt = 0.1/k_{\text{off}}$); (F) Average motion type after sub/track sectioning (0: immobile, 1: confined, 2: free motion). White pixels indicate areas with less than 10 tracks successfully analyzed.

16. Effects of residual sulfation on particle binding and mobility

The parental heparin used in the study has on average 2.5 sulfation groups per disaccharide unit. Given that 3O sulfation is rare and most likely does not significantly contribute to the chain sulfation, and assuming that the occupation of the remaining sulfation sites is equally likely, we consider an occupation probability for each sulfation site of 80%. The enzymatic desulfation used to produce the heparin derivatives specifically targets single sulfation sites with an efficiency >90%. In particular, for N-deS, N-sulfation is reduced by 92% (according to supplier, Iduron, UK) on average, resulting in an occupation probability of N-sulfation sites of 6.4%. While drastically reduced, this is not negligible, with an 80% probability of having at least one N-sulfation on a 25-units chain (Table S6). Previous studies have proposed that the binding between HPV16 and heparan sulfate involves several binding pockets on the virus capsomer and at least 4 disaccharide units. It is unknown how many of these units are required to be sulfated to obtain a stable bond. However, we can determine the probability of observing 1 to 4 consecutive N-sulfates using a simple Markov model (see section 17 "Discrete Markov model for heparin sulfation"). In the case of parental heparin, virtually all chains possess two consecutive N-sulfation sites and 99.4% four. In the case of N-deS, this number is reduced to only 8.9% to 0.035%, respectively. Considering the grafting coverage of N-deS heparin (3.8×10^{12} cm^{-2}, Table 1), we can determine the surface coverage of binding motifs to be 0.34×10^{12} cm^{-2} to 1.33×10^{9} cm^{-2}, respectively. Despite the steep reduction, in the most conservative case of 4 consecutive N-sulfated units needed for stable binding, 2.25×10^{5} binders are present per field of view. Particles binding to this residual sulfation pockets will be stably bound to the surface and might contribute to the number of irreversibly bound particles (Fig. S20). In the case of the more diluted N-deS:PEG::1:5 used also in kinetic studies, we estimate a reduction of the GAG grafting coverage of around 25 times (see section 17) to as low as 10^{4} possible binders per field of view, strongly decreasing but not eliminating possible interactions mediated by N-sulfation.

17. Discrete Markov model for heparin sulfation

We model a heparin chain composed by M monomers as a series of an equivalent number of independent Bernoulli trials, where each monomer is either sulfated with probability p or not sulfated with probability $q = 1 - p$. We seek the probability that the chain contains at least one run of N consecutive sulfated monomers at a given sulfation site type(19,20). Reading monomers sequentially, if the next monomer is sulfated, we increase the current count of consecutive sulfated monomers by one; if it is not sulfated, the count resets to zero. We can thus build a Markov chain with states $S_0, S_1, \ldots, S_{N-1}, S_N^*$ where S_k (for $\mathrm{k} = 0, \ldots, \mathrm{N} - 1$) means that no run of N has occurred yet and the current run length is k, and S_N^* is an absorbing state indicating that a run of N has occurred, regardless of the current count.

With these assumptions, the one-step transition matrix T of dimension N+1×N+1 is:

$$T = \begin{bmatrix} q & p & 0 & \cdots & 0 & 0 \\ q & 0 & p & \cdots & 0 & 0 \\ \vdots & \vdots & \ddots & \ddots & \vdots & \vdots \\ q & 0 & \cdots & 0 & p & 0 \\ q & 0 & \cdots & 0 & 0 & p \\ 0 & 0 & \cdots & 0 & 0 & 1 \end{bmatrix},$$

i.e., from S_k (for $k \leq N - 1$) a sulfated monomer moves to S_{k+1} with probability p and a non-sulfated monomer moves to S_0 with probability q. S_N^* is absorbing, hence once in that state, the system will remain there.

We start before the first monomer in state S_0, which can be expressed with a row vector $v_0 = [1, 0, \cdots, 0]$ (dimension 1×N+1). After reading the m-th monomer the distribution of the probability of being in each state is:

$$v_m = v_{m-1}T = v_{m-2}TT = v_0T^m.$$

For a full chain, we obtain $v_M = v_0T^M$, and the probability $P_{N,M}$ of having encountered at least one run of at least N sulfated monomers is equivalent to the probability of being in state S_N^* after M transitions, i.e., the last element of v_M: $P_{N,M} = (v_M)_{N+1} = (v_0T^M)_{N+1}$.

Table S6: Summary of residual N-sulfation in N-deS substrates as a function of the number of consecutive N-sulfation sites required for stable HPV16 binding and grafting coverage, assuming random distribution of GAG chain on the SLB. Grafting coverage reflecting N-deS only, N-deS:PEG::1:1, N-deS:PEG::1:5, 38×10^{11} cm^{-2}, 6.4×10^{11} cm^{-2}, 1.6×10^{11} cm^{-2} (Table 1), respectively; particle contact area: 1800 nm^2.

Consecutive N-sulf. sites for binding	% Chains with binding sites	% Surface allowing multivalent binding		
		N-deS only	N-deS:PEG 1:1	N-deS:PEG 1:5
1	80.86	100	99.9	68.1
2	8.89	98.5	27.2	2.82
3	0.56	5.84	0.20	1.31×10^{-2}
4	3.5×10^{-2}	2.9×10^{-2}	8.3×10^{-4}	5.2×10^{-5}

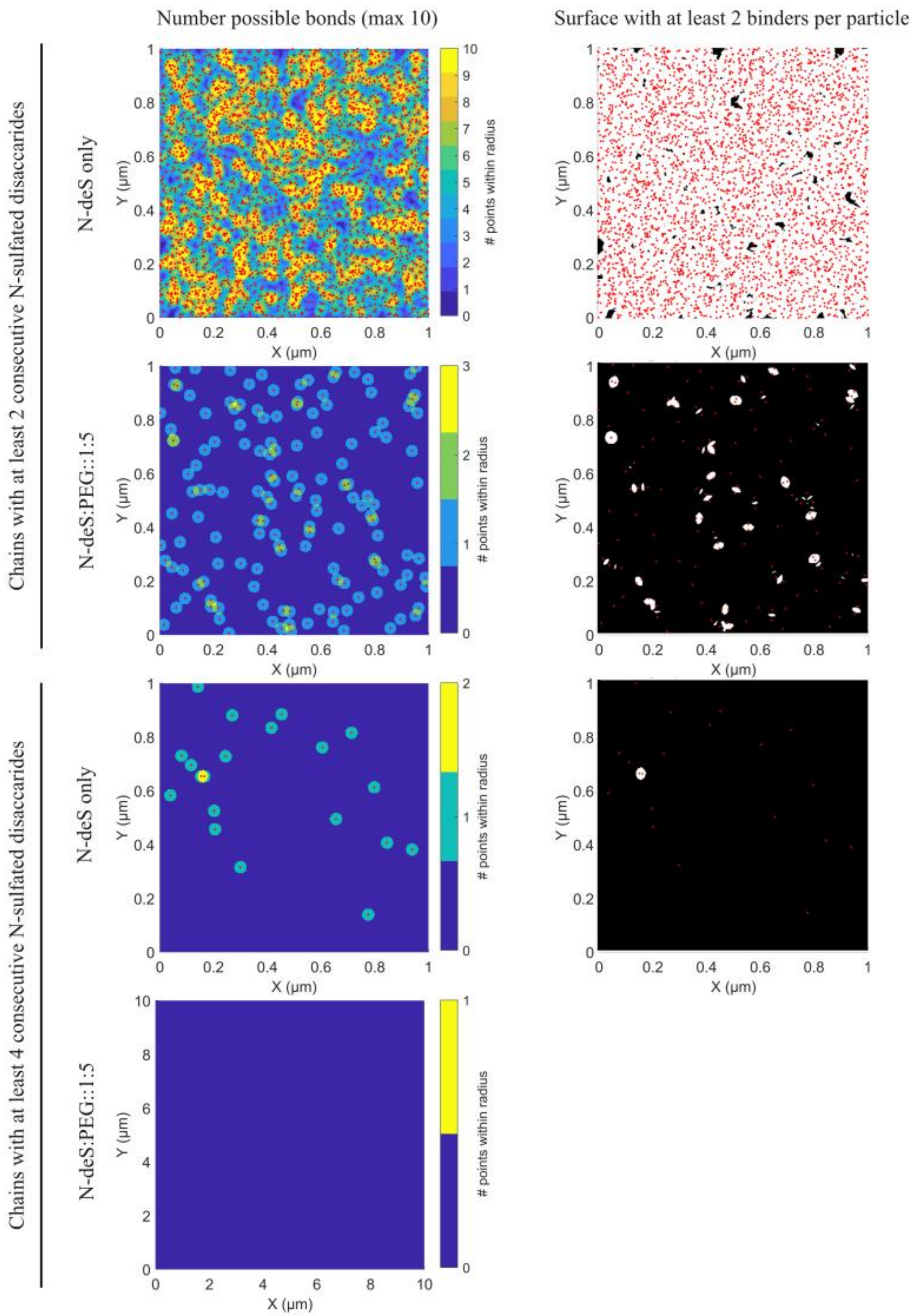

Figure S20. Simulated distribution of N-deS heparin chains maintaining 2 (*top four panels*) or 4 (*bottom 3 panels*) consecutive N-sulfated disaccharides (red dots) superimposed to a

heat map showing the maximum number of N-sulfation assisted bonds a HPV16 particle could form, *left*, and the fraction of surface that allows multivalent bonds resulting in immobile and irreversibly bound particle (white area), *right*. Grafting coverage reflecting N-deS only and N-deS:PEG::1:5, 38×10^{11} cm^{-2} and 1.6×10^{11} cm^{-2}, respectively; particle contact area: 1800 nm^2.

References

1. Bano, F., S. Banerji, M. Howarth, D. G. Jackson, and R. P. Richter. 2016. A single molecule assay to probe monovalent and multivalent bonds between hyaluronan and its key leukocyte receptor CD44 under force. *Sci. Rep.* 6(1):34176, doi: 10.1038/srep34176.
2. Thakar, D., E. Migliorini, L. Coche-Guerente, R. Sadir, H. Lortat-Jacob, D. Boturyn, O. Renaudet, P. Labbe, and R. P. Richter. 2014. A quartz crystal microbalance method to study the terminal functionalization of glycosaminoglycans. *Chem. Commun*. 50(96):15148-15151, doi: 10.1039/c4cc06905f.
3. Bano, F., L. Soria-Martinez, D. van Bodegraven, K. Thorsteinsson, A. M. Brown, I. Fels, N. L. Snyder, M. Bally, and M. Schelhaas. 2024. Site-specific sulfations regulate the physicochemical properties of papillomavirus-heparan sulfate interactions for entry. *Sci. Adv*. 10(40):eado8540, doi: 10.1126/sciadv.ado8540.
4. Emilsson, G., R. L. Schoch, L. Feuz, F. Hook, R. Y. Lim, and A. B. Dahlin. 2015. Strongly stretched protein resistant poly(ethylene glycol) brushes prepared by grafting-to. *ACS Appl. Mater. Interfaces*. 7(14):7505-7515, doi: 10.1021/acsami.5b01590.
5. del Castillo, G. F. D., G. Emilsson, and A. Dahlin. 2018. Quantitative Analysis of Thickness and pH Actuation of Weak Polyelectrolyte Brushes. *J. Phys. Chem. C*. 122(48):27516-27527, doi: 10.1021/acs.jpcc.8b09171.
6. Dahlin, A. B., M. Mapar, K. L. Xiong, F. Mazzotta, F. Höök, and T. Sannomiya. 2014. Plasmonic Nanopores in Metal-Insulator-Metal Films. *Adv. Opt. Mater*. 2(6):556-564, doi: 10.1002/adom.201300510.
7. Altgärde, N., E. Nilebäck, L. de Battice, I. Pashkuleva, R. L. Reis, J. Becher, S. Möller, M. Schnabelrauch, and S. Svedhem. 2013. Probing the biofunctionality of biotinylated hyaluronan and chondroitin sulfate by hyaluronidase degradation and aggrecan interaction. *Acta Biomater.* 9(9):8158-8166, doi: 10.1016/j.actbio.2013.05.031.
8. Lin, R., J. C. Kwok, D. Crespo, and J. W. Fawcett. 2008. Chondroitinase ABC has a long-lasting effect on chondroitin sulphate glycosaminoglycan content in the injured rat brain. *J. Neurochem*. 104(2):400-408, doi: 10.1111/j.1471-4159.2007.05066.x.
9. Berg, H. C. 1984. Random Walks in Biology. Princeton University Press.
10. Walker, D. M. 1998. The expected time until absorption when absorption is not certain. *J. Appl. Probab.* 35(4):812-823, doi: 10.1239/jap/1032438377.
11. Migliorini, E., D. Thakar, R. Sadir, T. Pleiner, F. Baleux, H. Lortat-Jacob, L. Coche-Guerente, and R. P. Richter. 2014. Well-defined biomimetic surfaces to characterize glycosaminoglycan-mediated interactions on the molecular,

supramolecular and cellular levels. *Biomaterials*. 35(32):8903-8915, doi: 10.1016/j.biomaterials.2014.07.017.

12. Pavlov, G., S. Finet, K. Tatarenko, E. Korneeva, and C. Ebel. 2003. Conformation of heparin studied with macromolecular hydrodynamic methods and X-ray scattering. *Eur. Biophys. J. Biophy*. 32(5):437-449, doi: 10.1007/s00249-003-0316-9.
13. Knappe, M., S. Bodevin, H. C. Selinka, D. Spillmann, R. E. Streeck, X. S. Chen, U. Lindahl, and M. Sapp. 2007. Surface-exposed amino acid residues of HPV16 L1 protein mediating interaction with cell surface heparan sulfate. *J. Biol. Chem*. 282(38):27913-27922, doi: 10.1074/jbc.M705127200.
14. Sapp, M., and M. Bienkowska-Haba. 2009. Viral entry mechanisms: human papillomavirus and a long journey from extracellular matrix to the nucleus. *FEBS J*. 276(24):7206-7216, doi: 10.1111/j.1742-4658.2009.07400.x.
15. Rubinstein, M., and R. H. Colby. 2003. Polymer Physics. Oxford University Press.
16. Zhang, B., J. Zerubia, and J.-C. Olivo-Marin. 2007. Gaussian approximations of fluorescence microscope point-spread function models. *Appl. Opt.* 46(10):1819-1829, doi: 10.1364/AO.46.001819.
17. Becker, M., D. V. Conca, N. Dorma, N. Mistry, E. Hahlin, L. Frangsmyr, M. Bally, N. Arnberg, and G. Gerold. 2023. Efficient clathrin-mediated entry of enteric adenoviruses in human duodenal cells. *J. Virol*. 97(10):e0077023, doi: 10.1128/jvi.00770-23.
18. Volpe, G., S. Gigan, and G. Volpe. 2014. Simulation of the active Brownian motion of a microswimmer. *Am. J. Phys*. 82(7):659-664, doi: 10.1119/1.4870398.
19. Moshkovitz, I., and Y. Barron (2025). The Waiting Time Distribution of Competing Patterns in Markov-Dependent Bernoulli Trials. *Axioms*. 14(3):221, doi: 10.3390/axioms14030221.
20. Coleman, B. D., and T. G. Fox. 1963. General theory of stationary random sequences with applications to the tacticity of polymers. *J. Polym. Sci. Part A: Gen. Pap*. 1(10):3183-3197, doi: 10.1002/pol.1963.100011013.
